# Access to enantioenriched 2,3- and 2,5-dihydrofurans with a fully substituted C2 stereocenter by Pd-catalyzed asymmetric intermolecular Heck reaction†
†Electronic supplementary information (ESI) available: Experimental procedures, characterization of all new compounds, VCD studies and spectral data. See DOI: 10.1039/c5sc01460c



**DOI:** 10.1039/c5sc01460c

**Published:** 2015-06-03

**Authors:** Gustavo M. Borrajo-Calleja, Vincent Bizet, Thomas Bürgi, Clément Mazet

**Affiliations:** a Department of Organic Chemistry , University of Geneva , 30 quai Ernest Ansermet , 1211 Geneva-4 , Switzerland . Email: clement.mazet@unige.ch; b Department of Physical Chemistry , University of Geneva , 30 quai Ernest Ansermet , 1211 Geneva-4 , Switzerland

## Abstract

A palladium-catalyzed intermolecular asymmetric Heck reaction with dihydrofurans with a trisubstituted double bond is reported. The use of two different chiral ligands provides access to valuable 2,3- and 2,5-dihydrofurans with a fully substituted C2 stereocenter with high levels of regio- and enantiocontrol.

## Abstract 2

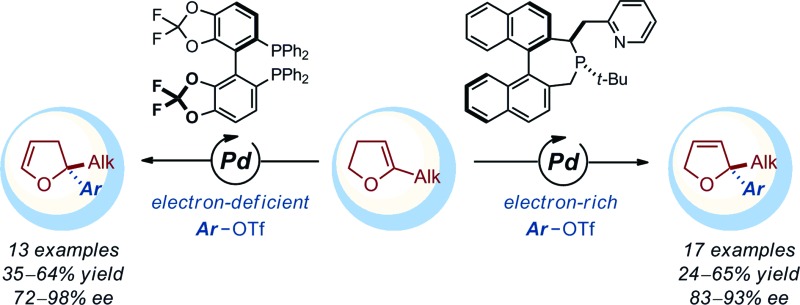
A palladium catalyzed intermolecular asymmetric Heck reaction provides access to valuable 2,3- and 2,5-dihydrofurans with a fully substituted C2 stereocenter with high levels of regio- and enantiocontrol.

## Introduction

**Fig. 1:**
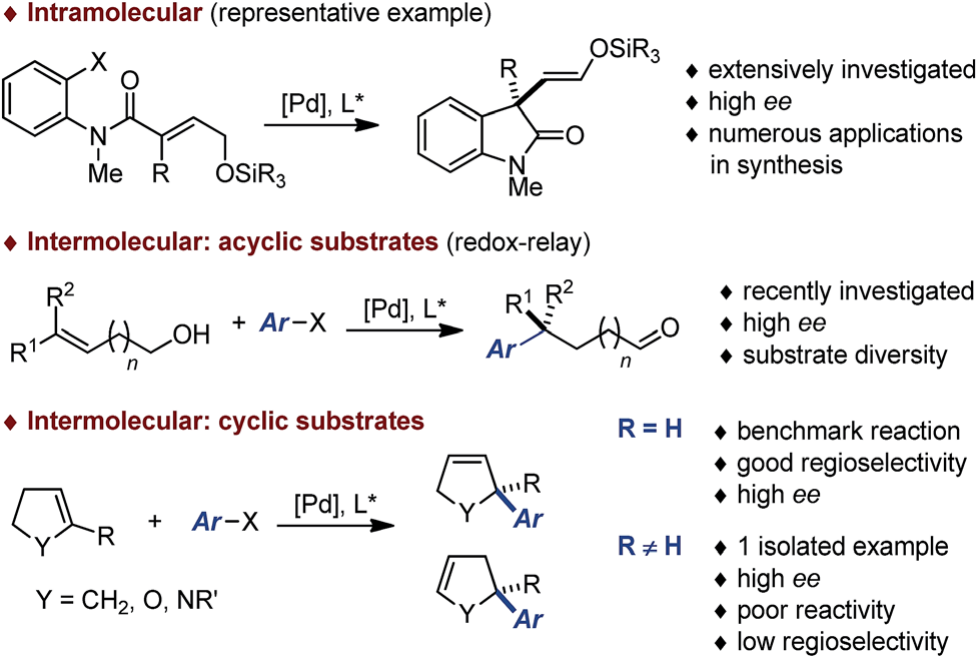
Access to quaternary and related fully saturated stereocenters by asymmetric Heck reactions.

During the past decades, building on the seminal contributions of Overman and Shibasaki, the asymmetric intramolecular Heck reaction has been successfully applied in natural product synthesis to install tertiary and quaternary stereocenters.^1,2^ In stark contrast, the *intermolecular* version of this asymmetric transformation has not reached the same level of development and its synthetic utility remains limited (Fig. 1).^3^ A survey of the literature following Hayashi's pioneering contribution indicates that this transformation has been used essentially as a benchmark reaction to validate the design of novel homotopic and heterotopic chiral ligands.^4,5^ While variations of the electronic and steric nature of the aryl halides and pseudo-halides has been investigated in detail, most of these studies have been focused on the use of dihydrofurans or N-protected dihydropyrroles to afford the corresponding coupling products with a C2 tertiary center. Systematic studies where emphasis has been placed on exploring the diversity of the olefinic coupling partner are scarce, in particular for the construction of congested quaternary centers or related fully saturated centers. Very recently, Sigman has reported a series of elegant contributions for the redox-relay Heck arylations of acyclic alkenyl alcohols enabling the installation of remote tertiary and quaternary stereogenic centers.^6,7^ Despite these recent breakthroughs, it is apparent that the development of an intermolecular asymmetric Heck reaction to enable the construction of *fully saturated stereocenters* in *cyclic* systems constitutes an important synthetic challenge.^8^

In this report, we disclose a methodology that gives access to chiral 2,3- and 2,5-dihydrofurans (dhfs) with a fully substituted C2 stereocenter. Our study highlights the complementarity in product selectivity between two distinct chiral ligands while it also delineates the scope of trisubstituted dihydrofurans compatible with the catalytic systems.

## Results and discussion

The choice of our model reaction was inspired by a report from Pregosin and co-workers where 5-methyl-2,3-dihydrofuran **1a** was cross-coupled with phenyl triflate **2a** using (*R*)-DTB-MeO-BIPHEP (*i.e.* (*R*)-(+)-2,2′-bis[di(3,5-di-*tert*-butylphenyl)phosphino]-6,6′-dimethoxy-1,1′-biphenyl).^9^ To the best of our knowledge this represents the only precedent of an asymmetric intermolecular Heck reaction employing a cyclic substrate with a trisubstituted olefinic moiety. Despite an excellent enantioselectivity (**4aa**: 98% ee), the low yield (38%), the moderate regioselectivity (1.0 : 6.0) and the impractical reaction time (7 days) certainly precluded further developments. We began our investigations by evaluating an array of chiral (P,N) and (P,P) ligands as to their complementarity in providing access to the product of direct cross-coupling **3** and the product of further isomerization **4**, respectively, as is well documented for unsubstituted cyclic substrates.^4,5,10^

With an initial set of reaction conditions (Pd(OAc)_2_, toluene, *i*-Pr_2_NEt, 110 °C, 62 h), only traces of products **3aa** and **4aa** were observed in the coupling between **1a** and **2a** using **L1** (Table 1, entry 1). Evaluation of two of our home-made chiral (P,N) ligands did not significantly enhance the reactivity, even though **3aa** was the only detectable product of cross-coupling.^11^ While **3aa** was obtained in 76% ee with **L3**, **L4** gave an excellent 94% ee value (entry 2 and 3). A slightly improved reactivity along with similar regio- and enantioselectivities were obtained with the electron-rich 4-methoxyphenyl triflate **2b** (entry 4). An extensive optimization of all reaction parameters was then conducted. Multiple combinations of palladium sources, solvents and bases along with adjustment of the temperature, stoichiometry, concentration and reaction time led to the identification of a very efficient catalytic system (Pd_2_(dba)_3_, 2-Me-THF, *i*-Pr_2_NEt, 100 °C, 48 h; see ESI† for details) affording **3ab** in 64% and 93% ee with **L4** (entry 5).

**Table 1:** Reaction optimization^*a*^

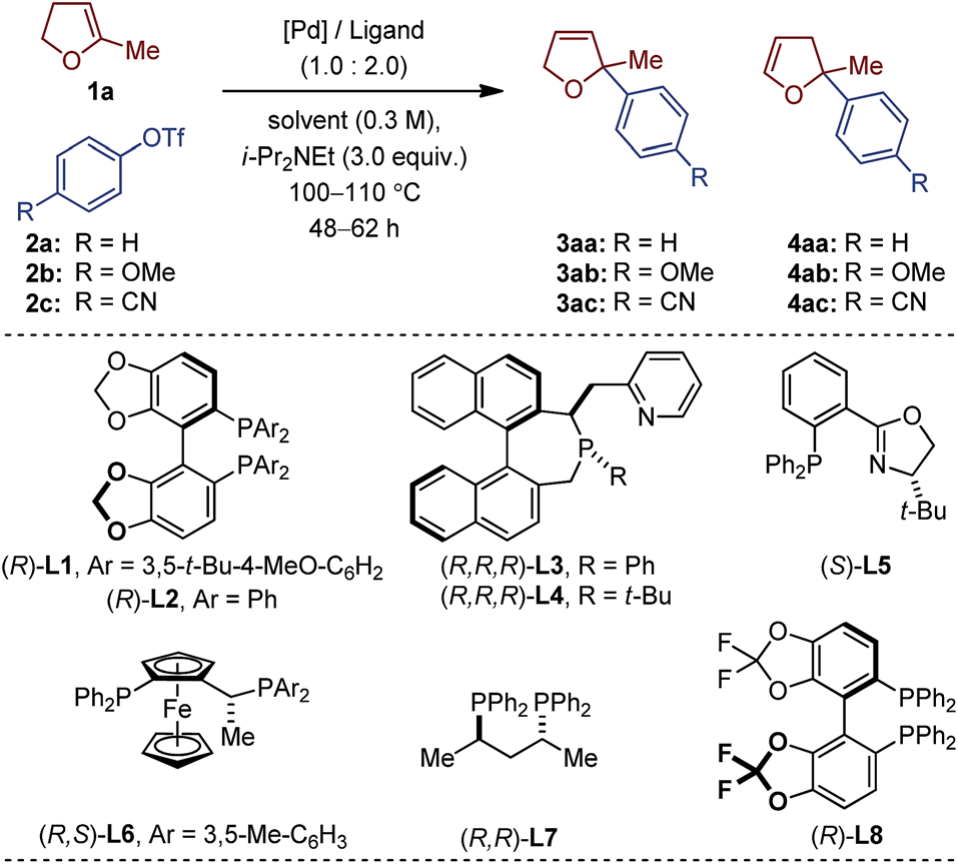							
Entry^*b*^	**2**	Ligand	**3** : **4** ^*c*^	**3**		**4**	
				Yield^*d*^ (%)	ee^*e*^ (%)	Yield^*d*^ (%)	ee^*e*^ (%)
1	**2a**	**L1**	22 : 78	<5	nd^*f*^	5	9
2	**2a**	**L3**	99 : 1	12	76	—	—
3	**2a**	**L4**	99 : 1	16	94	—	—
4	**2b**	**L4**	99 : 1	11	92	—	—
5	**2b**	**L4**	99 : 1	64	93	—	—
6	**2c**	**L4**	—	nr^*g*^	—	nr	—
7	**2b**	**L5**	98 : 2	34	98	—	—
8	**2c**	**L5**	—	nr	—	nr	—
9	**2b**	**L2**	44 : 56	21	rac	28	13
10	**2c**	**L2**	5 : 95	—	—	55	93
11	**2b**	**L6**	5 : 95	—	—	30	56
12	**2c**	**L6**	5 : 95	—	—	32	62
13	**2b**	**L7**	5 : 95	—	—	39	45
14	**2c**	**L7**	5 : 95	—	—	34	51
15	**2b**	**L8**	57 : 43	<5	—	<5	—
16	**2c**	**L8**	5 : 95	—	—	51	97

^*a*^ *Reaction conditions*: **1a** (1 mmol), **2a–c** (0.2 mmol).

^*b*^Entries 1–4: Pd(OAc)_2_ (3 mol%), ligand (6 mol%), toluene, 110 °C, 62 h. Entries 5–16: Pd_2_(dba)_3_ (2.5 mol%), ligand (10 mol%), 2-Me-THF, 100 °C, 48 h.

^*c*^Determined by ^1^H NMR of the crude reaction mixture.

^*d*^Yield of pure compound after chromatography.

^*e*^Determined by GC or HPLC with a chiral column.

^*f*^Not determined.

^*g*^No reaction.

Of important note, while the catalyst loading, the relative stoichiometry between the coupling partners and the yields obtained (*ca.* 40–65%) are typical of *intermolecular* asymmetric Heck reactions, 48 h is unusually short for such a process involving cyclic olefins. Under classical thermal conditions, reaction times often range from 4 to 7 days.^12^ Unexpectedly, when the electron-deficient aryl triflate **2c** was employed, no product of cross-coupling was observed.^13^ The prototypical (P,N) ligand **L5** for intermolecular Heck reactions using unsubstituted dhf did not display better performances (entry 7 and 8). For sake of operational simplicity, the same protocol was used in the survey of several (P,P) ligands (entry 9–16). Consistent with literature precedents for the coupling of unsubstituted dhf, a marked regioselectivity switch in favor of the isomerized products **4** was observed with most ligands. Noticeably, while poor results were obtained in the coupling reactions with the electron-rich triflate **2b**, the electron-deficient aryl triflate **2c** permitted to reach excellent enantioselectivity levels with **L2**, and **L8** (entry 10 and 16).^14^

This dichotomous situation was further confirmed when the scope or aryl triflates compatible with both **L4** and **L8** was delineated (Table 2). With the (P,N) ligand **L4**, a wide variety of electron-rich aryl triflates afforded quasi-exclusively 2,5-dihydrofurans (98 : 2–99 : 1) in consistently very high enantioselectivity (88–93% ee) and in practical yields. Remarkably, even the sterically demanding *o*-methoxyphenyl triflate **2k** was cross-coupled efficiently (**3ak**: 53%, 99 : 1, 90% ee). Reduced yields were obtained with **2a** and **2l**, albeit the regio- and enantioselectivities remained excellent. Attempts to couple electron-deficient aryl triflates were unsuccessful. In contrast, with the (P,P) ligand **L8**, only electron-deficient aryl triflates **2c**, **2m–r** were found to be compatible electrophilic coupling partners. This afforded 2,3-dihydrofurans with a saturated C2 stereocenter in excellent enantioselectivity and allowed functional groups such as cyano, nitro, trifluoromethyl, ester, aldehyde, aryl and alkyl ketones to be introduced.

**Table 2:** Scope of aryl triflates^*a*^

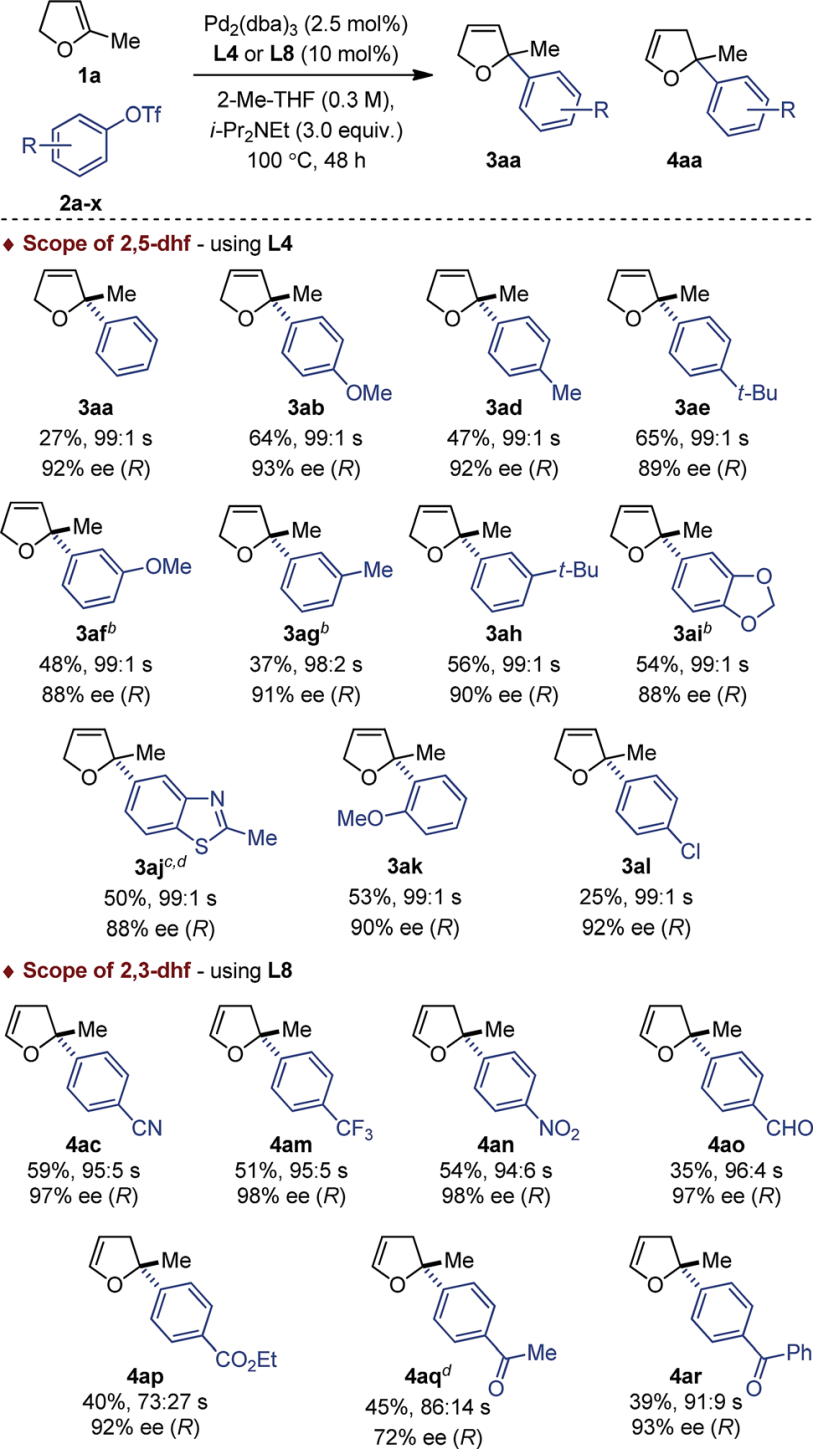

^*a*^ *Reaction conditions*: **1a** (1 mmol), **2a–c** (0.2 mmol). Absolute configurations determined by VCD. The regioselectivity 3 : 4 is denoted by s.

Seven different 5-substituted 2,3-dhfs were found to be suitable substrates with both methodologies (Table 3 and 4). These compounds proved particularly unstable and presented challenges for study because of the difficulty associated with their preparation and purification (see ESI†).

**Table 3:** Scope of 2,2-disubstituted 2,5-dhfs with **L4** ^*a*^

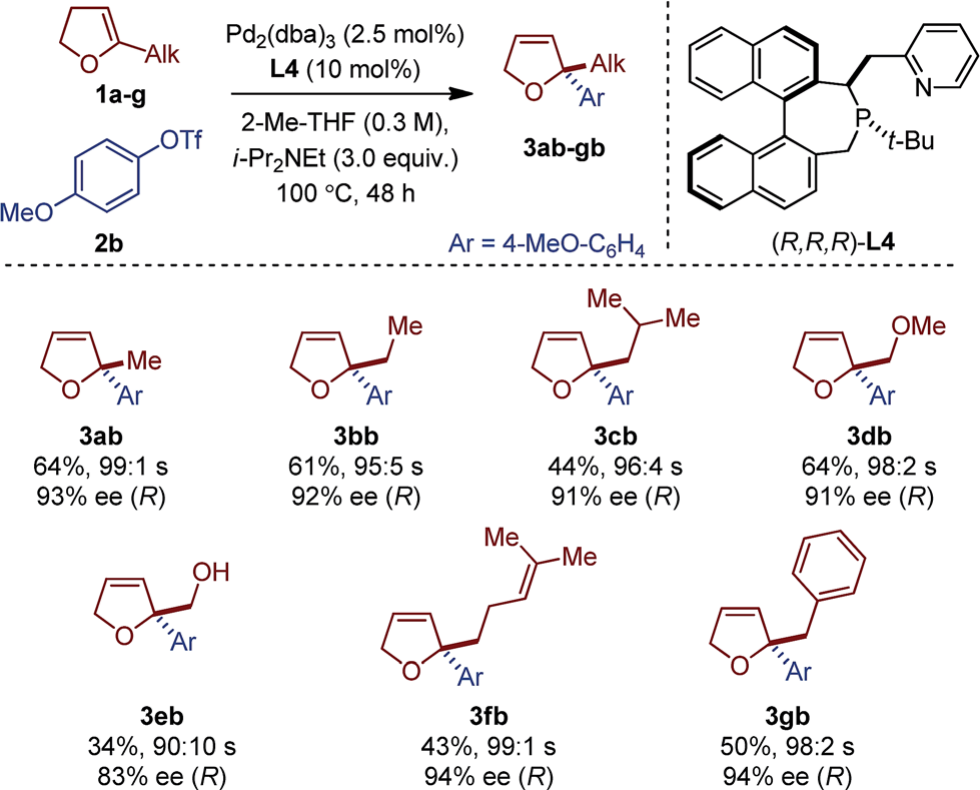

^*a*^ *Reaction conditions*: **1a** (1 mmol), **2a–c** (0.2 mmol). Absolute configurations determined by VCD.

**Table 4:** Scope of 2,2-disubstituted 2,3-dhfs with **L8** ^*a*^

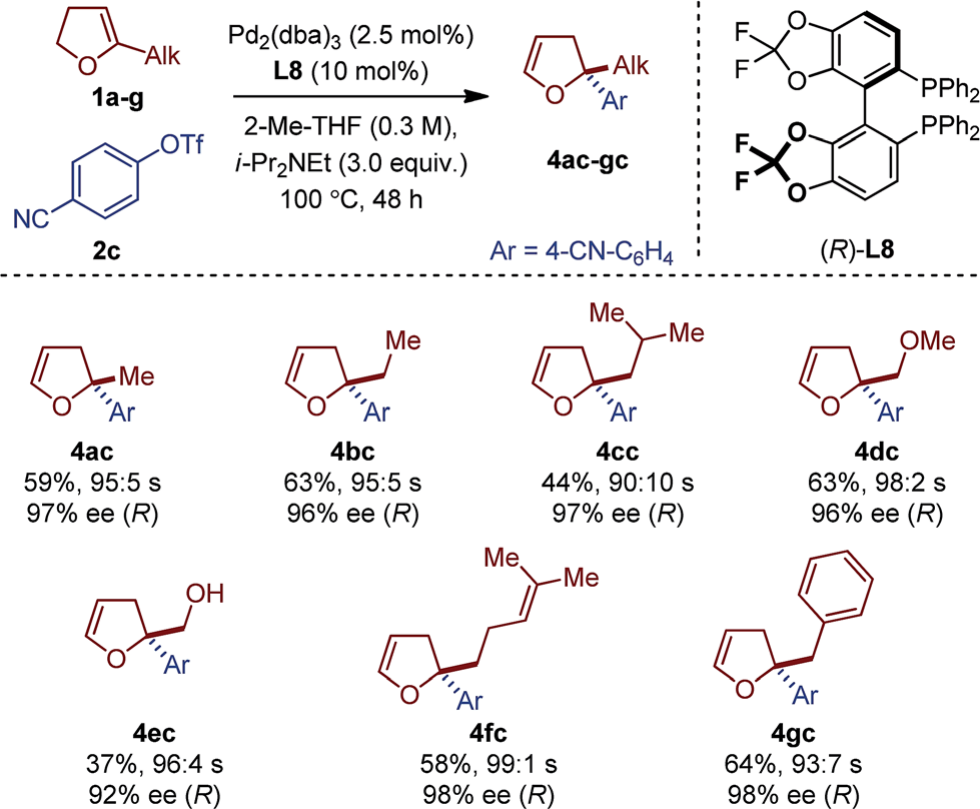

^*a*^ *Reaction conditions*: **1a** (1 mmol), **2a–c** (0.2 mmol). Absolute configurations determined by VCD.

**Fig. 2:**
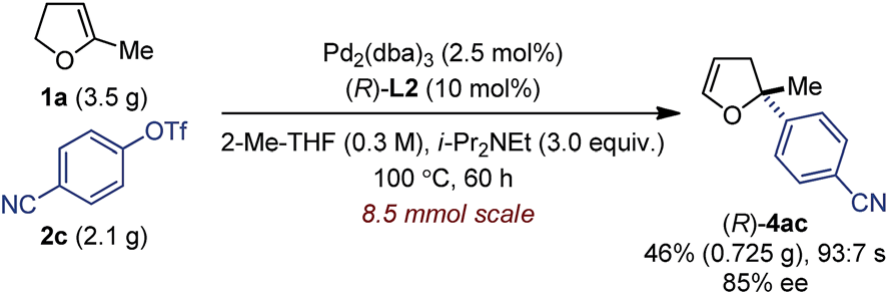
Large-scale experiment for the intermolecular asymmetric Heck reaction.

The *C*_1_-symmetric (P,N) ligand **L4** and the *C*_2_-symmetric (P,P) ligand **L8** gave access to 2,2-disubstituted 2,5-dhf and 2,2-disubstituted 2,3-dhf, respectively, in usually practical yields and with excellent regio- and enantioselectivity. Both catalytic systems behaved similarly and overall excellent results were obtained. The yield range obtained for these reactions are consistent with literature precedents.^2,3,6^ They do not only reflect the entropic cost inherent to an intermolecular cross-coupling reaction but also the difficulty associated with creating saturated stereocenters. In addition to primary alkyl substituents, a methyl ether, a free alcohol, a remote olefin and a benzyl substituent were compatible. Whereas the yields were slightly reduced in the reactions with **3e**, it is remarkable that no competing Heck arylation was observed in the reactions with **1f** which bears a remote olefinic moiety.^6^ Finally, a large-scale experiment (8.5 mmol) was conducted for the cross-coupling between **1a** and **2c** (Fig. 2). Using (*R*)-**L2**, the isomerized product (*R*)-**4ac** was isolated with excellent regioselectivity, good enantioselectivity and in 46% yield (0.725 g).^15^

The absolute configuration of the cross-coupling products was established by vibrational circular dichroism (VCD).^16^ The most stable conformer of **3ab** (obtained with (*R*,*R*,*R*)-**L4**) and **4ap** (obtained with (*R*)-**L8**) were compared to the experimental IR absorption and VCD spectra recorded in CD_2_Cl_2_ solutions (see ESI† for details). The good agreement between the predicted and measured data enabled the assignment of a (*R*) configuration to both compounds. The absolute configuration of all other cross-coupling products was assigned by analogy, on the basis of their optical activity.

## Conclusions

In summary, we have developed an efficient protocol for the enantioselective intermolecular Heck reaction using a variety of 5-substituted 2,3-dihydrofurans. We found that electron-rich aryl triflates can be coupled with various trisubstituted olefinic substrates when a chiral (P,N) ligand is used. The corresponding 2,5-dihydrofurans with fully saturated C2 stereocenters were obtained in excellent regio- and enantioselectivities. Electron-deficient aryl triflates were compatible with the method when commercial chiral (P,P) ligands were employed, leading to 2,2-disubstituted 2,3-dihydrofurans with excellent stereocontrol. The diversity, functional group tolerance and scalability of the approach have been also demonstrated and provide access to enantioenriched dihydrofurans that would be difficult to prepare otherwise.^17^ The underlying mechanistic features responsible for the marked electronic dichotomy observed between the two ligand systems, along with extension of the method to other substrate classes, are currently being investigated in our laboratories.
