# Supplementary material for: Access to enantioenriched 2,3- and 2,5-dihydrofurans with a fully substituted C2 stereocenter by Pd-catalyzed asymmetric intermolecular Heck reaction
Source: Chem Sci. 2015 Jun 3;6(8):4807–11. doi: 10.1039/c5sc01460c (PMC5667573; doi:10.1039/c5sc01460c)

Electronic Supplementary Material (ESI) for Chemical Science.
This journal is © The Royal Society of Chemistry 2015

Access to Enantioenriched 2,3- and 2,5-Dihydrofurans with a Fully Substituted C2 Stereocenter by Pd-Catalyzed Asymmetric Intermolecular Heck Reaction

Gustavo M. Borrajo-Calleja,[a] Vincent Bizet,[a] Thomas Bürgi,[b] Clément Mazet[a,*]

[a]*Departement of Organic Chemistry, University of Geneva*
[b]*Departement of Physical Chemistry, University of Geneva*
Quai Ernest Ansermet 30, 1211 Geneva 4, Switzerland
Email: Clement.Mazet@unige.ch

Supporting Information

Table of Contents

**1**	**General**	**S2**
**2**	**Optimization of the asymmetric Heck reaction**	**S3**
2.1	Reaction conditions optimization for 2-(4-methoxyphenyl)-2-methyl-2,5-dihydrofuran (**3ab**)	**S3**
2.2	Ligand screening for 2-aryl-2-methyl-2,3-dihydrofurans	**S4**
**3**	**Vibrational circular dichroism (VCD) measurements**	**S8**
3.1	(*R*)-2-(4-methoxyphenyl)-2-methyl-2,5-dihydrofuran (**3ab**)	**S9**
3.2	(*R*)-2-(4-ethoxycarbonylphenyl)-2-methyl-2,3-dihydrofuran (**4ap**)	**S14**
**4**	**Experimental procedures and characterization data**	**S19**
4.1	General procedure for the synthesis of 5-alkyl-2,3-dihydrofuran (in a 2-Me-THF solution) (**GP1**)	**S19**
4.2	Procedures for the synthesis of 5-alkyl-2,3-dihydrofuran (neat)	**S20**
4.3	General procedure for the asymmetric intermolecular Heck reaction with neat 5-alkyl-2,3-dihydrofurans (**GP2**)	**S23**
4.4	General procedure for the asymmetric intermolecular Heck reaction with 5-alkyl-2,3-dihydrofurans in a 2-Me-THF solution (**GP3**)	**S23**
4.5	Characterization data of 2-aryl-2-methyl-2,5-dihydrofurans	**S24**
4.6	Characterization data of 2-aryl-2-methyl-2,3-dihydrofurans	**S35**
4.7	Characterization data of 2-alkyl-2-aryl-2,5-dihydrofurans	**S42**
4.8	Characterization data of 2-alkyl-2-aryl-2,3-dihydrofurans	**S48**
**5**	**NMR spectra of all compounds**	**S55**

1 General

All reactions were carried out under an inert atmosphere of nitrogen using either two-manifold vacuum/inert gas lines or a *M. Braun* glove-box, unless otherwise noted. Solvents were dried over activated alumina columns and further degassed by three successive "freeze-pump-thaw" cycles if necessary.

Unless otherwise noted, commercial reagents were purchased from *Aldrich*, *Acros* or *Strem* and used without further purification. Liquid reagents were transferred with stainless steel syringes or cannula. Thin layer chromatography (TLC) was performed on plates of silica precoated with 0.25 mm Kieselgel 60 F_{254} from *Merck*. Flash chromatography was performed using silica gel SiliaFlash® P60 (230–400 mesh) from *Silicycle*. NMR spectra were recorded on ARX-300 and AMX-400 and AM-500 *Bruker Advance* spectrometers. ^{1}H and ^{13}C{^{1}H} NMR chemical shifts are given in ppm relative to $SiMe_4$, with the solvent resonance used as internal reference. ^{19}F{^{1}H} NMR chemical shifts are reported in ppm relative to $CFCl_3$. Infrared spectra were obtained on a *Perkin-Elmer* 1650 FT-IR spectrometer using neat samples on a diamond ATR Golden Gate sampler. The mass spectrometric data were obtained at the mass spectrometry facility of the University of Geneva (http://www.ms.unige.ch/sms). GC-MS analyses were performed on GC – HP 6890, column Agilent – HP1 (30 m – ID 0.32 mm, Film 0.25 µm) coupled with MS – HP 5973. The enantiomeric excesses (ee's) were determined by HPLC or GC analyses. HPLC analyses were performed on *Shimadzu* CTO-20AA, with column DAICEL OD-H, OJ-H, AD-H and IC. GC analyses were performed on HP – 6890, column Lipodex E, 50m. Retention times (t_R) are given in minutes.

The aryl trifluoromethanesulfonates were prepared according to reported procedures.[1] (*R*)-Difluorphos was purchased from *Strem*, other chiral ligands were obtained from commercial sources. The homemade ligand (*R,R,R*) and (*S,S,S*)-**L1** were prepared according to a previous report.[2] The 5-methyl-2,3-dihydrofuran 97% (**1a**) was obtained from *Sigma-Aldrich* and used without further purification. The racemates of **3aa**, **3ab**, **3ad**, **3ae**, **3af**, **3ag**, **3ah**, **3ai**, **3aj** and **3al** were obtained using **GP2**, and for **3bb** using **GP3**, with 2-(2-(diphenylphosphino)phenyl)-4,5-dihydrooxazole as ligand.[3] The racemates of **3ak**, **3db**, **3eb**, **3fb** and **3gb** were obtained using **GP2**, and for **3cb** using **GP3**, with the racemic mixture (*R,R,R/S,S,S*)-**L1** as ligand.[4] The racemates of **4ac**, **4am**, **4an**, **4ao**, **4ap**, **4aq**, **4ar**, **4dc**, **4ec**, **4fc** and **4gc** were obtained using **GP2**, and for **4bc** and **4cc** using **GP3**, with *rac*-BINAP as ligand.

1 (*a*) L. Qin, X. Ren, Y. Lu, Y. Li and J. Zhou, *Angew. Chem. Int. Ed.*, 2012, **51**, 5915; (*b*) For 2-methylbenzo[d]thiazol-5-yl trifluoromethanesulfonate, see : E. V. Vinogradova, N. H. Park, B. P. Fors and S. L. Buchwald, *Org. Lett.*, 2013, **15**, 1394.

2 P. Nareddy, L. Mantilli, L. Guénée and C. Mazet, *Angew. Chem. Int. Ed.*, 2012, **51**, 1.

3 B. Wüstenberg and A. Pfaltz, *Adv. Synth. Catal.,* 2008, **350**, 174.

4 *Rac*-**L1** was obtained by mixing both enantiomers.

2 Optimization of the asymmetric Heck reaction

2.1 Reaction conditions optimization for 2-(4-methoxyphenyl)-2-methyl-2,5-dihydrofuran (3ab)

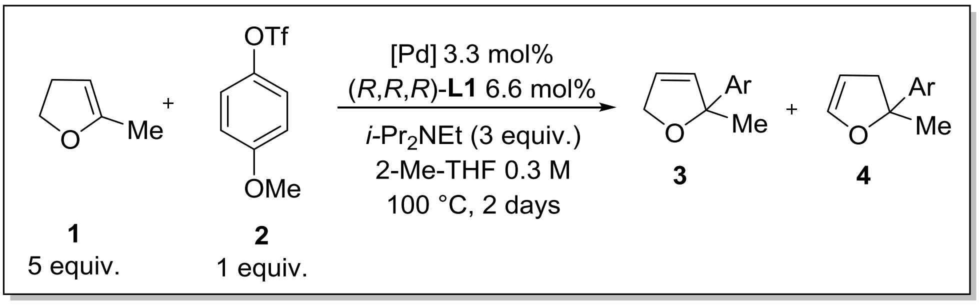

Entry	Pd source	Solvent	Base	**3/4**[a]	**3** yield (%)[b]	**3** *ee* (%)[c]
**1**	$Pd(OAc)_2$	Toluene	*i*-Pr_2NEt	99/1	*nd*[d]	*nd*[d]
**2**	$Pd(OAc)_2$	THF	*i*-Pr_2NEt	99/1	13	93
**3**	$Pd(OAc)_2$	DMF	*i*-Pr_2NEt	*nd*[d]	11	93
**4**	$Pd(OAc)_2$	Dioxane	*i*-Pr_2NEt	99/1	37	94
**5**	$Pd(OAc)_2$	2-Me-THF	*i*-Pr_2NEt	99/1	52	94
**6**	$Pd(OAc)_2$	TBME[e]	*i*-Pr_2NEt	*nd*[d]	5	92
**7**	$Pd(OAc)_2$	CPME[f]	*i*-Pr_2NEt	*nd*[d]	4	93
**8**	$Pd(OAc)_2$	BME[g]	*i*-Pr_2NEt	*nr*[h]	*nr*[h]	*nr*[h]
**9**	$Pd(OAc)_2$	2-Me-THF	PMP[i]	99/1	15	93
**10**	$Pd(OAc)_2$	2-Me-THF	Li_2CO_3	*nr*[h]	*nr*[h]	*nr*[h]
**11**	$Pd(OAc)_2$	2-Me-THF	Urotropine	*nr*[h]	*nr*[h]	*nr*[h]
**12**	$Pd(OAc)_2$	2-Me-THF	DABCO[j]	*nd*[d]	23	92
**13**[k]	$Pd(OAc)_2$	2-Me-THF	*i*-Pr_2NEt	99/1	56	93
**14**[l]	$Pd(OAc)_2$	2-Me-THF	*i*-Pr_2NEt	99/1	54	93
**15**[l,m]	$Pd(OAc)_2$	2-Me-THF	*i*-Pr_2NEt	99/1	42	93
**16**[l,n]	$Pd(OAc)_2$	2-Me-THF	*i*-Pr_2NEt	99/1	31	93
**17**[o]	$Pd(OAc)_2$	2-Me-THF	*i*-Pr_2NEt	*nd*[d]	*nd*[d]	93
**18**[l]	$Pd(TFA)_2$	2-Me-THF	*i*-Pr_2NEt	99/1	31	93
**19**[l]	$Pd(Cl)_2(CH_3CN)_2$	2-Me-THF	*i*-Pr_2NEt	*nr*[h]	*nr*[h]	*nr*[h]
**20**[l]	$Pd_2(dba)_3$	2-Me-THF	*i*-Pr_2NEt	99/1	63	92
**21**[l,p]	$Pd_2(dba)_3$	2-Me-THF	*i*-Pr_2NEt	99/1	58	93

[a] Determined by ^{1}H-NMR in the reaction crude; [b] Isolated yield; [c] Determined by HPLC; [d] Not determined; [e] *t*-butyl methyl ether; [f] Cyclopentyl methyl ether; [g] benzyl methyl ether; [h] No reaction; [i] 1,2,2,6,6-Pentamethylpiperidine; [j] 1,4-diazabicyclo[2.2.2]octane; [k] 72 h; [l] 5 mol% of Pd and 10 mol% of ligand; [m] 0.6 M and 30 h; [n] 0.15 M and 30 h; [o] 1.0 M; [p] 0.6 M.

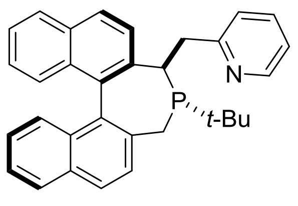

(*R*,*R*,*R*)-**L1**

2.2 Ligand screening for 2-aryl-2-methyl-2,3-dihydrofurans

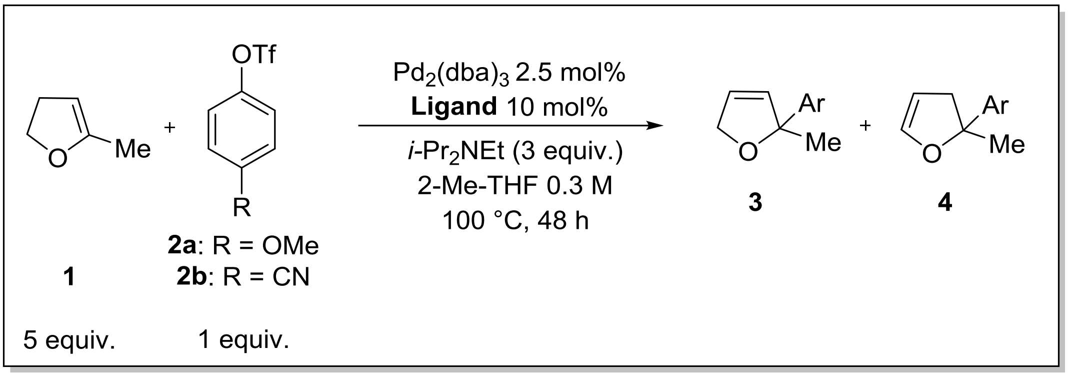

Entry	**2**	**Ligand**	**2** cons. (%)[a]	**3:4**[b]	**3** yield (%)[c]	**3** *ee* (%)[d]	**4** yield (%)[c]	**4** *ee* (%)[d]
**1**	**2a**	**L1**	97	66/33	*nd*[e]	*nd*[e]	*nd*[e]	*nd*[e]
**2**	**2b**	**L1**	99	*nd*[e]	*nd*[e]	*nd*[e]	*nd*[e]	*nd*[e]
**3**	**2a**	**L2**	33	99/1	*nd*[e]	*nd*[e]	*nd*[e]	*nd*[e]
**4**	**2b**	**L2**	99	*nd*[e]	*nd*[e]	*nd*[e]	*nd*[e]	*nd*[e]
**5**	**2a**	**L3**	0	*nr*[f]	*nr*[f]	*nr*[f]	*nr*[f]	*nr*[f]
**6**	**2b**	**L3**	99	*nd*[e]	*nd*[e]	nd[e]	10	36
**7**	**2a**	**L4**	0	*nr*[f]	*nr*[f]	*nr*[f]	*nr*[f]	*nr*[f]
**8**	**2b**	**L4**	0	*nr*[f]	*nr*[f]	*nr*[f]	*nr*[f]	*nr*[f]
**9**	**2a**	**L5**	7	*nr*[f]	*nr*[f]	*nr*[f]	*nr*[f]	*nr*[f]
**10**	**2b**	**L5**	35	10/90	*nd*	*nd*	10	85
**11**	**2a**	**L6**	5	*nr*[f]	*nr*[f]	*nr*[f]	*nr*[f]	*nr*[f]
**12**	**2b**	**L6**	18	*nr*[f]	*nr*[f]	*nr*[f]	*nr*[f]	*nr*[f]
**13**	**2a**	**L7**	0	*nr*[f]	*nr*[f]	*nr*[f]	*nr*[f]	*nr*[f]
**14**	**2b**	**L7**	12	10/90	*nd*[e]	*nd*[e]	*nd*[e]	*nd*[e]
**15**	**2a**	**L8**	15	10/90	*nd*[e]	*nd*[e]	*nd*[e]	*nd*[e]
**16**	**2b**	**L8**	45	5/95	*nd*[e]	*nd*[e]	23	31
**17**	**2a**	**L9**	99	5/95	*nd*[e]	*nd*[e]	39	45
**18**	**2b**	**L9**	99	10/90	*nd*[e]	*nd*[e]	34	51
**19**	**2a**	**L10**	92	5/95	*nd*[e]	*nd*[e]	30	56
**20**	**2b**	**L10**	99	5/95	*nd*[e]	*nd*[e]	32	62
**21**	**2a**	**L11**	99	*nr*[f]	*nr*[f]	*nr*[f]	*nr*[f]	*nr*[f]
**22**	**2b**	**L11**	99	*nd*	*nd*[e]	*nd*[e]	*nd*[e]	*nd*[e]
**23**	**2a**	**L12**	72	10/90	*nd*[e]	*nd*[e]	31	75
**24**	**2b**	**L12**	99	10/90	*nd*[e]	*nd*[e]	38	88
**25**	**2a**	**L13**	99	7/93	*nd*[e]	*nd*[e]	31	52
**26**	**2b**	**L13**	75	10/90	*nd*[e]	*nd*[e]	35	61
**27**	**2a**	**L14**	99	*nr*[f]	*nr*[f]	*nr*[f]	*nr*[f]	*nr*[f]
**28**	**2b**	**L14**	99	*nr*[f]	*nr*[f]	*nr*[f]	*nr*[f]	*nr*[f]

[a] Consumption of ArOTf **2** determined by ^{19}F-NMR of the crude; [b] Determined by ^{1}H-NMR of the crude; [c] Isolated yield; [d] Determined by HPLC; [e] Not determined; [f] No reaction.

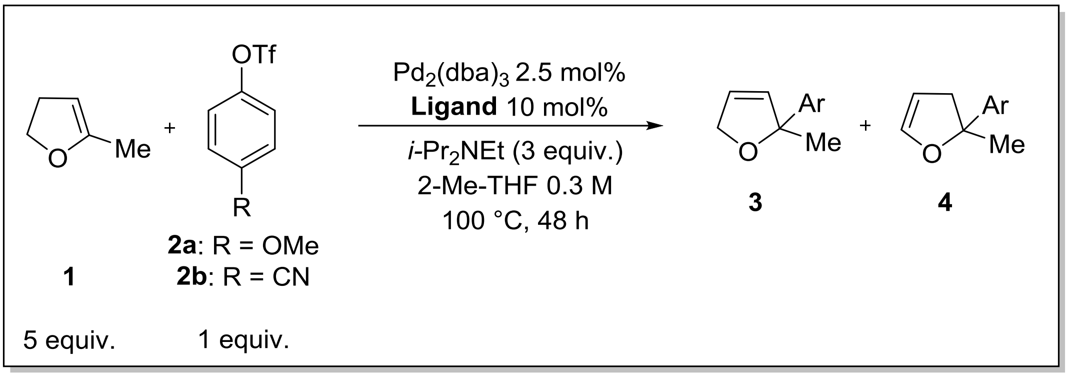

Entry	2	Ligand	2 cons. (%)[a]	3:4[b]	3 yield (%)[c]	3 ee (%)[d]	4 yield (%)[c]	4 ee (%)[d]
**29**	**2a**	**L15**	99	20/80	*nd*[e]	*nd*[e]	19	72
**30**	**2b**	**L15**	99	20/80	*nd*[e]	*nd*[e]	15	78
**31**	**2a**	**L16**	99	*nd*[e]	*nd*[e]	*nd*[e]	7	86
**32**	**2b**	**L16**	99	*nr*[f]	*nr*[f]	*nr*[f]	*nr*[f]	*nr*[f]
**33**	**2a**	**L17**	99	*nd*[e]	*nd*[e]	*nd*[e]	3	53
**34**	**2b**	**L17**	99	*nd*[e]	*nd*[e]	*nd*[e]	*nd*[e]	58
**35**	**2a**	**L18**	99	5/95	*nd*[e]	*nd*[e]	14	68
**36**	**2b**	**L18**	99	*nr*[f]	*nr*[f]	*nr*[f]	*nr*[f]	*nr*[f]
**37**	**2a**	**L19**	99	10/90	*nd*[e]	*nd*[e]	12	65
**38**	**2b**	**L19**	99	5/95	*nd*[e]	*nd*[e]	17	73
**39**	**2a**	**L20**	72	65/35	17	36	13	42
**40**	**2b**	**L20**	99	10/90	4	64	65	78
**41**	**2a**	**L21**	97	57/43	42[g]	17	42[g]	34
**42**	**2b**	**L21**	99	10/90	4	35	39	14
**43**	**2a**	**L22**	99	75/25	48[g]	69	48[g]	54
**44**	**2b**	**L22**	99	10/90	*nd*[e]	*nd*[e]	26	69
**45**	**2a**	**L23**	98	29/71	10	13	14	37
**46**	**2b**	**L23**	99	15/85	*nd*[e]	*nd*[e]	39	69
**47**	**2a**	**L24**	99	44/56	21	0	28	13
**48**	**2b**	**L24**	99	5/95	*nd*[e]	*nd*[e]	55	93
**49**	**2a**	**L25**	51	14/86	*nd*[e]	*nd*[e]	10	95
**50**	**2b**	**L25**	75	15/85	*nd*[e]	*nd*[e]	35	97
**51**	**2a**	**L26**	99	32/68	10	2	31	7
**52**	**2b**	**L26**	99	30/70	8	13	28	38
**53**	**2a**	**L27**	24	57/43	*nd*[e]	*nd*[e]	*nd*[e]	*nd*[e]
**54**	**2b**	**L27**	99	5/95	*nd*[e]	*nd*[e]	59	97
**55**	**2a**	**L28**	66	74/26	*nd*[e]	*nd*[e]	*nd*[e]	35
**56**	**2b**	**L28**	99	10/90	*nd*[e]	*nd*[e]	41	77
**57**	**2a**	**L29**	66	*nr*[f]	*nr*[f]	*nr*[f]	*nr*[f]	*nr*[f]
**58**	**2b**	**L29**	58	10/90	*nd*[e]	*nd*[e]	*nd*[e]	97

[a] Consumption of ArOTf **2** by ^{19}F-NMR of the crude; [b] Determined by ^{1}H-NMR of the crude; [c] Isolated yield; [d] Determined by HPLC; [e] Not determined; [f] No reaction; [g] Combined isolated yield of **3** and **4**.

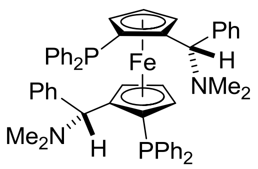

L1
(*R*)-(*S*)-NMe$_2$-PPh$_2$-Mandyphos

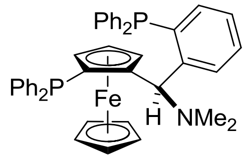

L2
(*R*)-(*S*)-Ph$_2$-PPhCHNMe$_2$-T-PPh$_2$

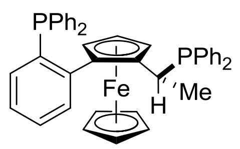

L3
(*R*)-(*R*)-Ph$_2$PPhFcCHCH$_3$PPh$_2$

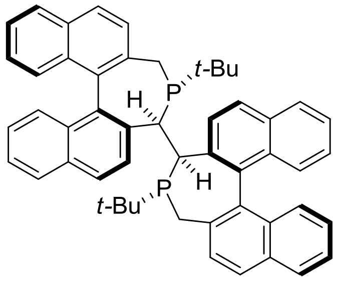

L4
(*S*)-BINAPINE

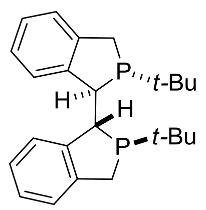

L5
(*R*,*R*,*S*,*S*)-DUANPHOS

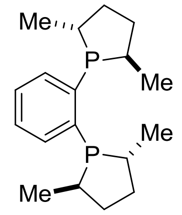

L6
(*R*,*R*)-Me-DUPHOS

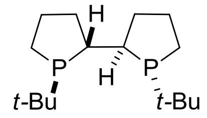

L7
(*S*,*S*,*R*,*R*)-TANGPHOS

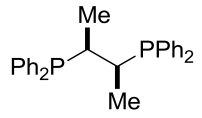

L8
(*S*,*S*)-CHIRAPHOS

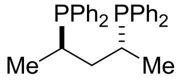

L9
(*R*,*R*)-BDPP

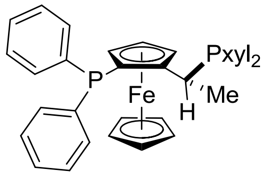

L10
(*R*)-(*S*)-PPF-Pxyl$_2$

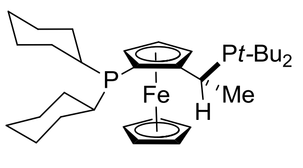

L11
(*R*)-(*S*)-cy$_2$PF-P^{t}Bu$_2$

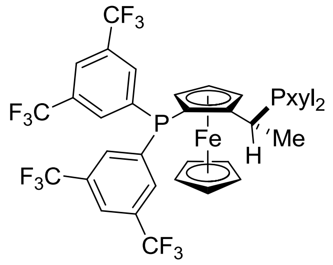

L12
(*R*)-(*S*)-(3,5-CF$_3$Ph)$_2$-PF-Pxyl$_2$

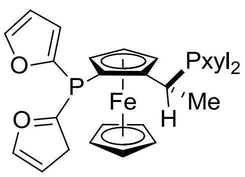

L13
(*R*)-(*S*)-2-Fur$_2$PF-Pxyl$_2$

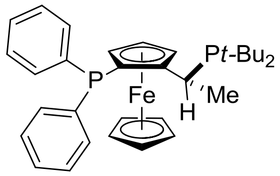

L14
(*R*)-(*S*)-PPF-P^{t}Bu$_2$

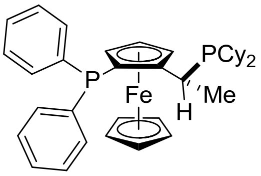

L15
(*R*)-(*S*)-PPF-Pcy$_2$

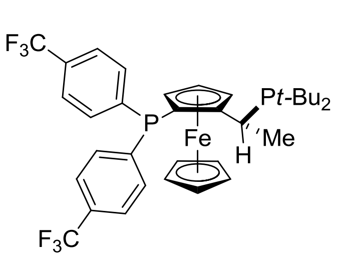

L16
(*R*)-(*S*)-(4-CF$_3$Ph)$_2$-PF-P^{t}Bu$_2$

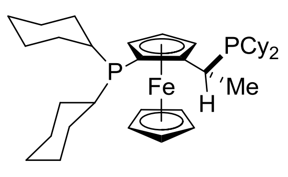

L17
(*R*)-(*S*)-cy$_2$PF-Pcy$_2$

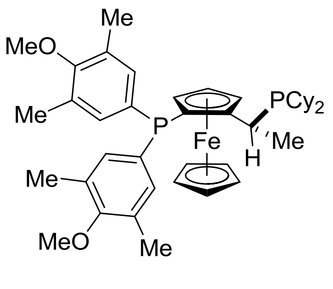

L18
(*R*)-(*S*)-(4-MeO-3,5-MePh)$_2$PF-Pcy$_2$

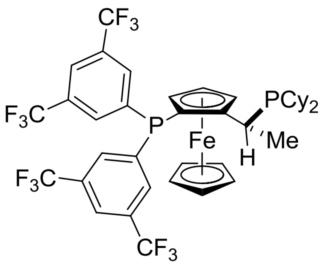

L19
(R)-(S)-(3,5-$CF_3Ph)_2PF$-Pcy_2

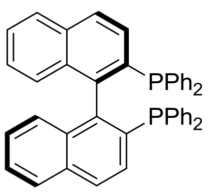

L20
(R)-BINAP

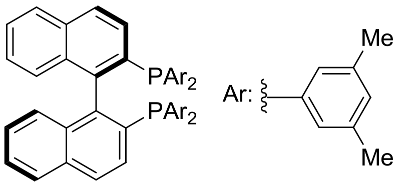

L21
(R)-DM-BINAP

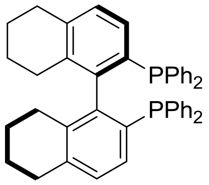

L22
(R)-H_8-BINAP

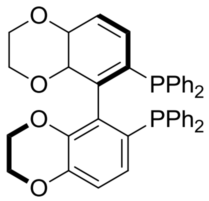

L23
(R)-SYNPHOS

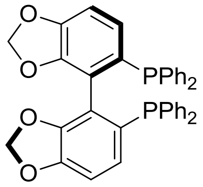

L24
(R)-SEGPHOS

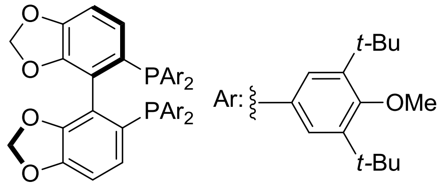

L25
(R)-DTBM-SEGPHOS

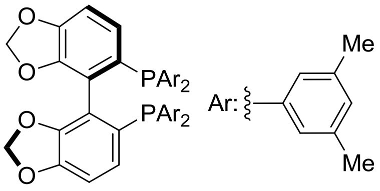

L26
(R)-DM-SEGPHOS

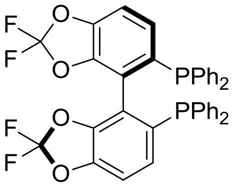

L27
(R)-DIFLUORPHOS

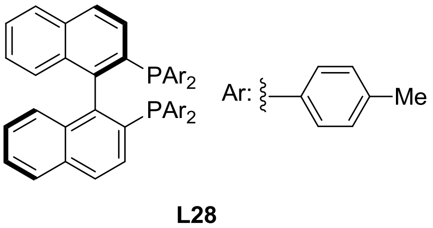

L28
(R)-T-BINAP

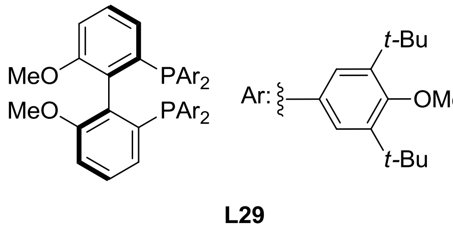

L29
(R)-DTBM-BIPHEP

3 Vibrational circular dichroism (VCD) measurements

IR and VCD measurements

IR and vibrational circular dichroism (VCD) spectra were recorded on a Bruker PMA 50 accessory coupled to a Tensor 27 Fourier transform infrared spectrometer. A photoelastic modulator (Hinds PEM 90) set at l/4 retardation was used to modulate the handedness of the circular polarized light. Demodulation was performed by a lock-in amplifier (SR830 DSP). An optical low-pass filter (< 1800 cm-1) in front of the photoelastic modulator was used to enhance the signal/noise ratio. Spectra were recorded with a transmission cell equipped with CaF_2 windows and a 0.2 mm Teflon spacer. For measurements solutions in CD_2Cl_2 were prepared. The solvent was measured under identical conditions and subtracted to from the VCD spectrum of the compound in order to eliminate artifacts. Samples were measured at a resolution of 4 cm^{-1} by averaging about 24’000 scans for both the sample and the solvent. Spectra are presented without further data processing.

IR and VCD calculations

The geometry optimizations, vibrational frequencies, IR absorption and VCD intensities were calculated with Density Functional Theory (DFT) using the B3PW91 functional and a 6-31G(d,p) basis set. Frequencies were scaled by a factor of 0.97. IR absorption and VCD spectra were constructed from calculated dipole and rotational strengths assuming Lorentzian band shape with a half-width at half maximum of 4 cm^{-1}. All calculations were performed using Gaussian09.[5]

5 Gaussian 09, Revision C.01, M. J. Frisch, G. W. Trucks, H. B. Schlegel, G. E. Scuseria, M. A. Robb, J. R. Cheeseman, G. Scalmani, V. Barone, B. Mennucci, G. A. Petersson, H. Nakatsuji, M. Caricato, X. Li, H. P. Hratchian, A. F. Izmaylov, J. Bloino, G. Zheng, J. L. Sonnenberg, M. Hada, M. Ehara, K. Toyota, R. Fukuda, J. Hasegawa, M. Ishida, T. Nakajima, Y. Honda, O. Kitao, H. Nakai, T. Vreven, J. A. Montgomery Jr., J. E. Peralta, F. Ogliaro, M. Bearpark, J. J. Heyd, E. Brothers, K. N. Kudin, V. N. Staroverov, T. Keith, R. Kobayashi, J. Normand, K. Raghavachari, A. Rendell, J. C. Burant, S. S. Iyengar, J. Tomasi, M. Cossi, N. Rega, J. M. Millam, M. Klene, J. E. Knox, J. B. Cross, V. Bakken, C. Adamo, J. Jaramillo, R. Gomperts, R. E. Stratmann, O. Yazyev, A. J. Austin, R. Cammi, C. Pomelli, J. W. Ochterski, R. L. Martin, K. Morokuma, V. G. Zakrzewski, G. A. Voth, P. Salvador, J. J. Dannenberg, S. Dapprich, A. D. Daniels, O. Farkas, J. B. Foresman, J. V. Ortiz, J. Cioslowski and D. J. Fox, Gaussian, Inc., Wallingford CT, 2010.

3.1 (*R*)-2-(4-methoxyphenyl)-2-methyl-2,5-dihydrofuran (3ab)

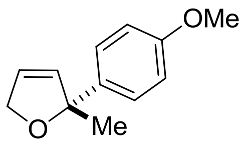

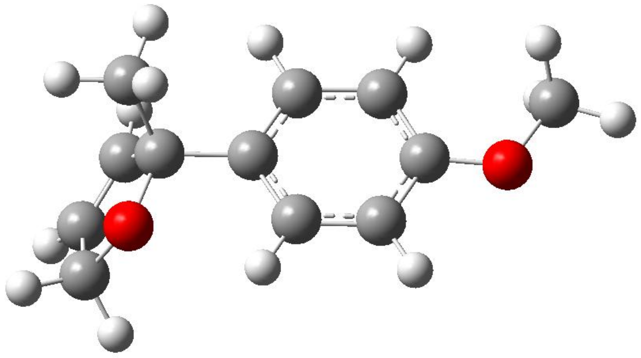

File Name	bizet1	
File Type	.log	
Calculation Type	FREQ	
Calculation Method	RB3PW91	
Basis Set	6-31G(d,p)	
Charge	0	
Spin	Singlet	
E(RB3PW91)	-615.89744326	a.u.
RMS Gradient Norm	0.00000565	a.u.
Imaginary Freq	0	
Dipole Moment	2.2896	Debye
Point Group	C1	

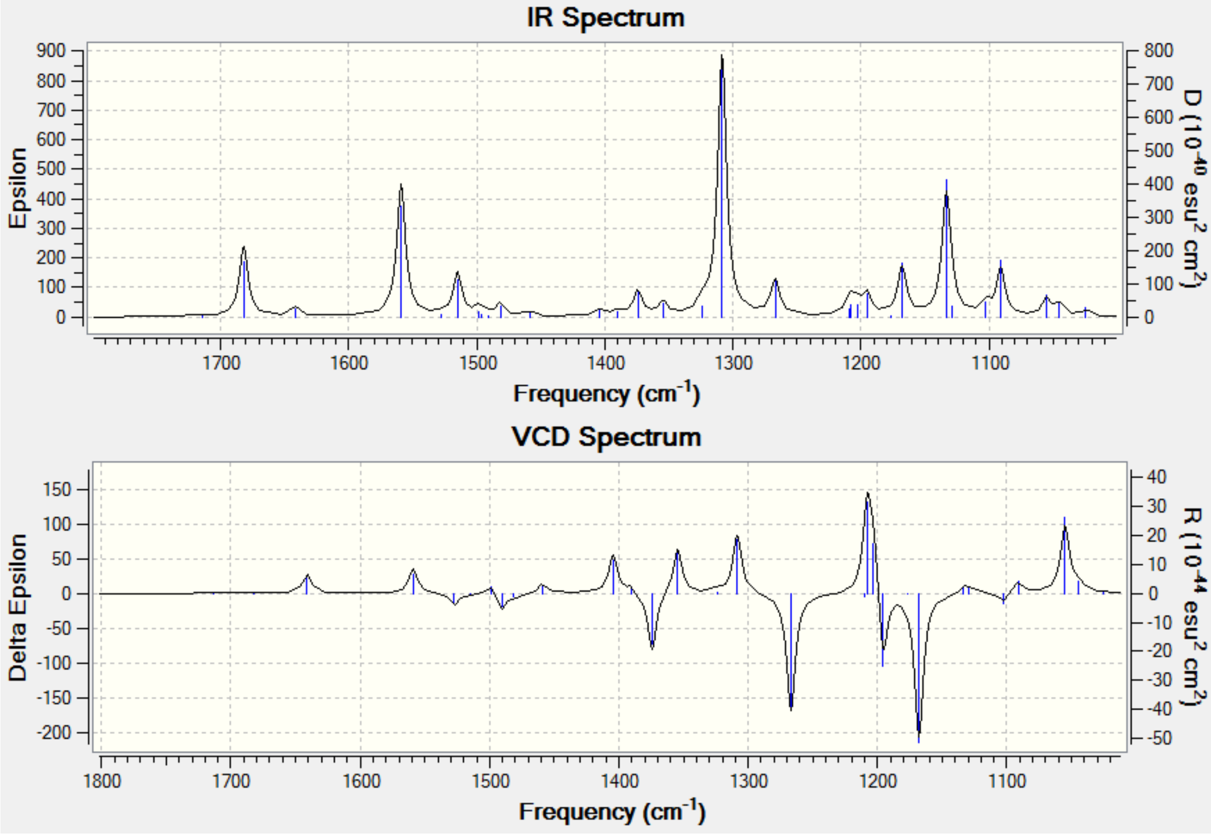

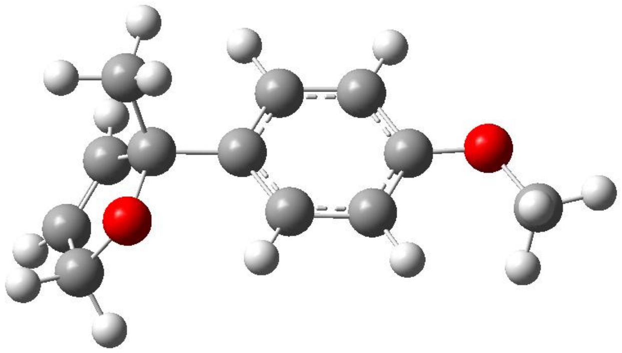

File Name	bizet2	
File Type	.log	
Calculation Type	FREQ	
Calculation Method	RB3PW91	
Basis Set	6-31G(d,p)	
Charge	0	
Spin	Singlet	
E(RB3PW91)	-615.89769655	a.u.
RMS Gradient Norm	0.00000144	a.u.
Imaginary Freq	0	
Dipole Moment	1.1160	Debye
Point Group	C1	

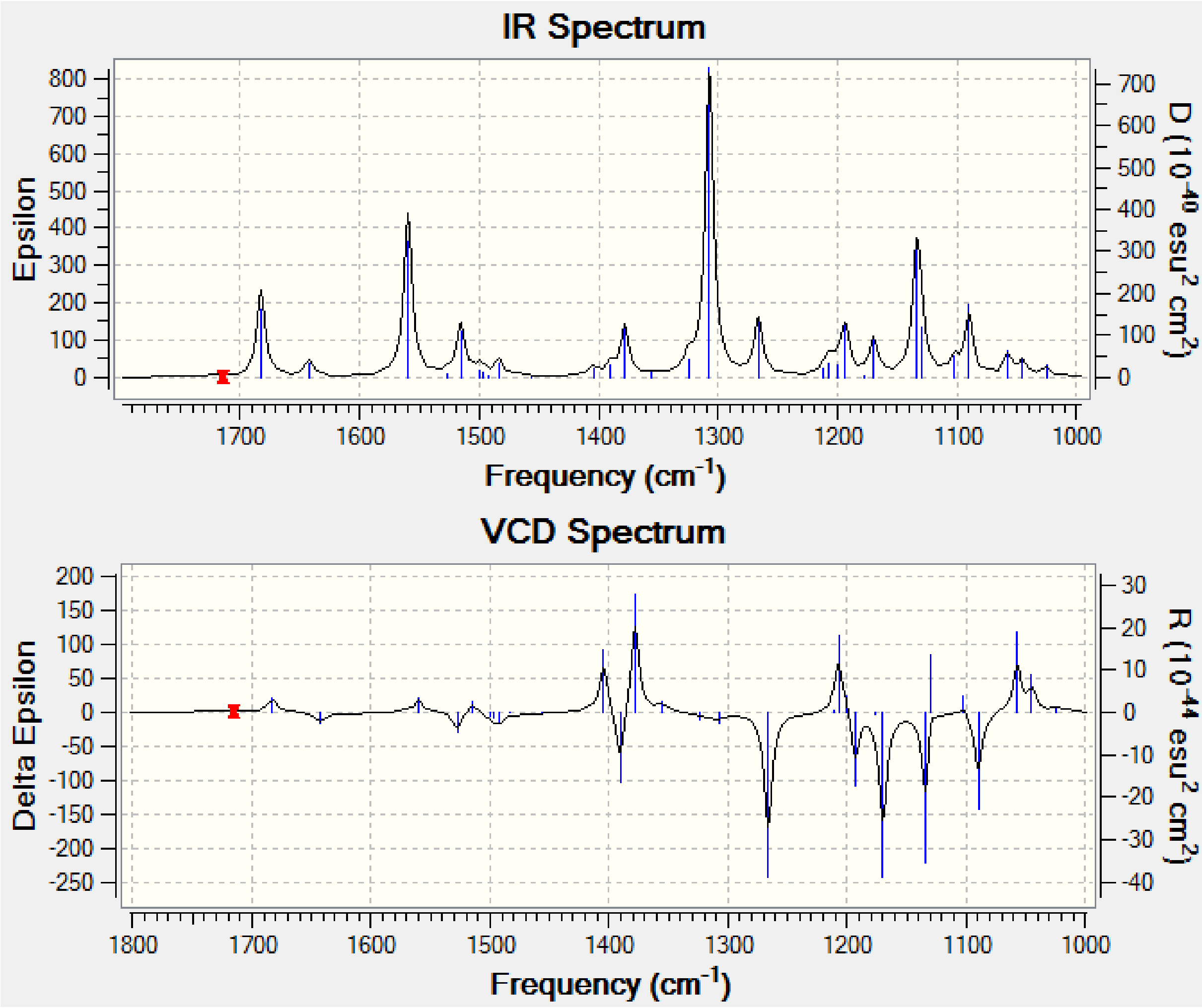

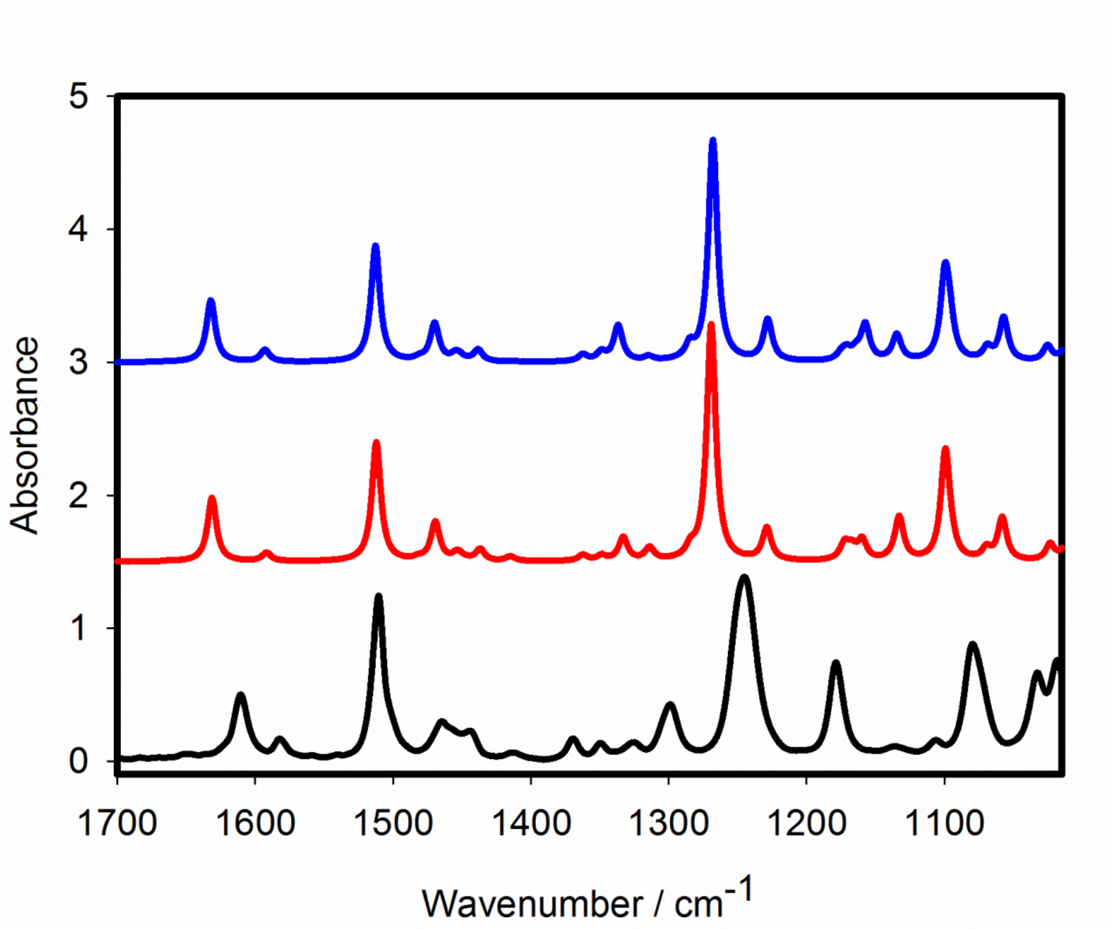

IR spectra: black: experiment (8 microliter in 200 microliter CD_2Cl_2), red and blue: calculated (two conformers).

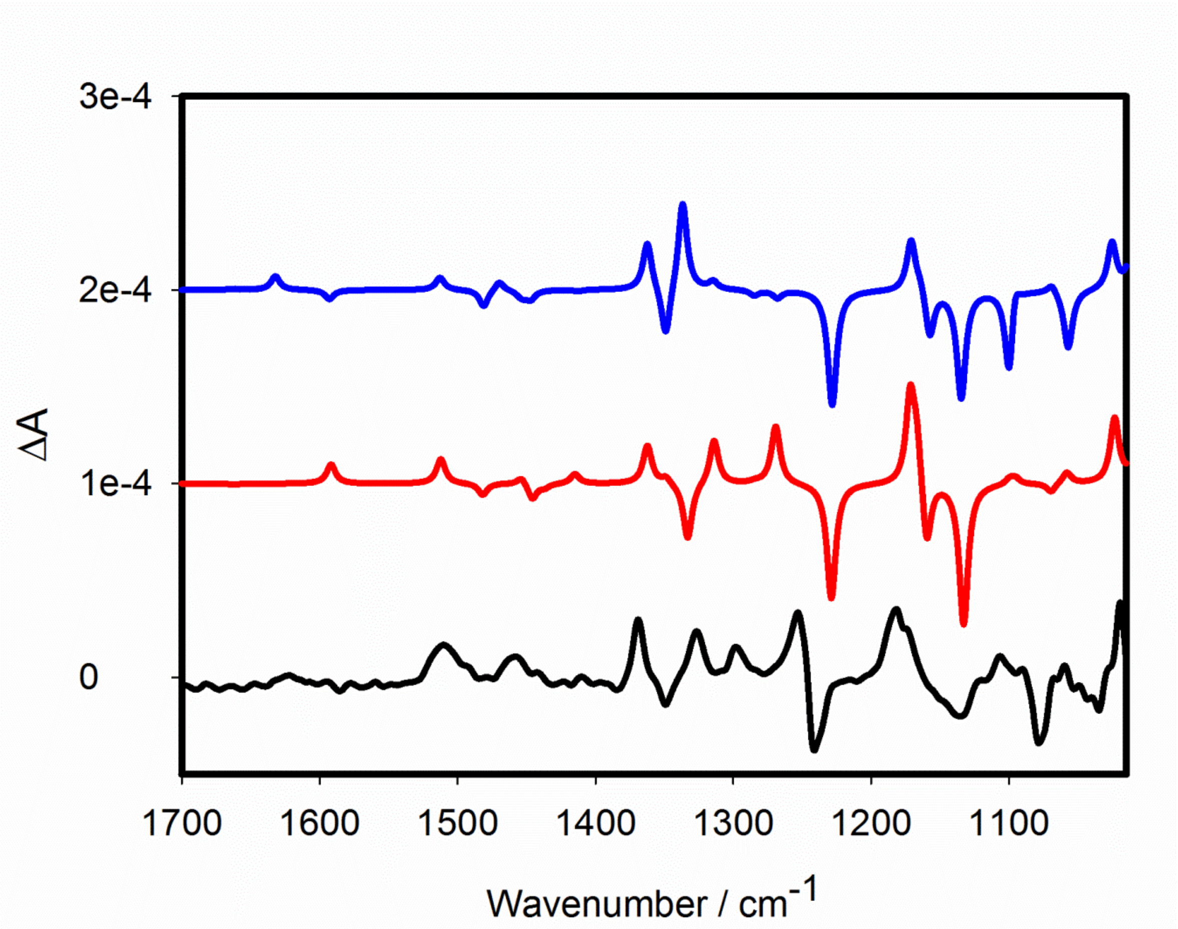

VCD spectra: black: experiment (8 microliter in 200 microliter CD_2Cl_2), red and blue: calculated (two conformers).

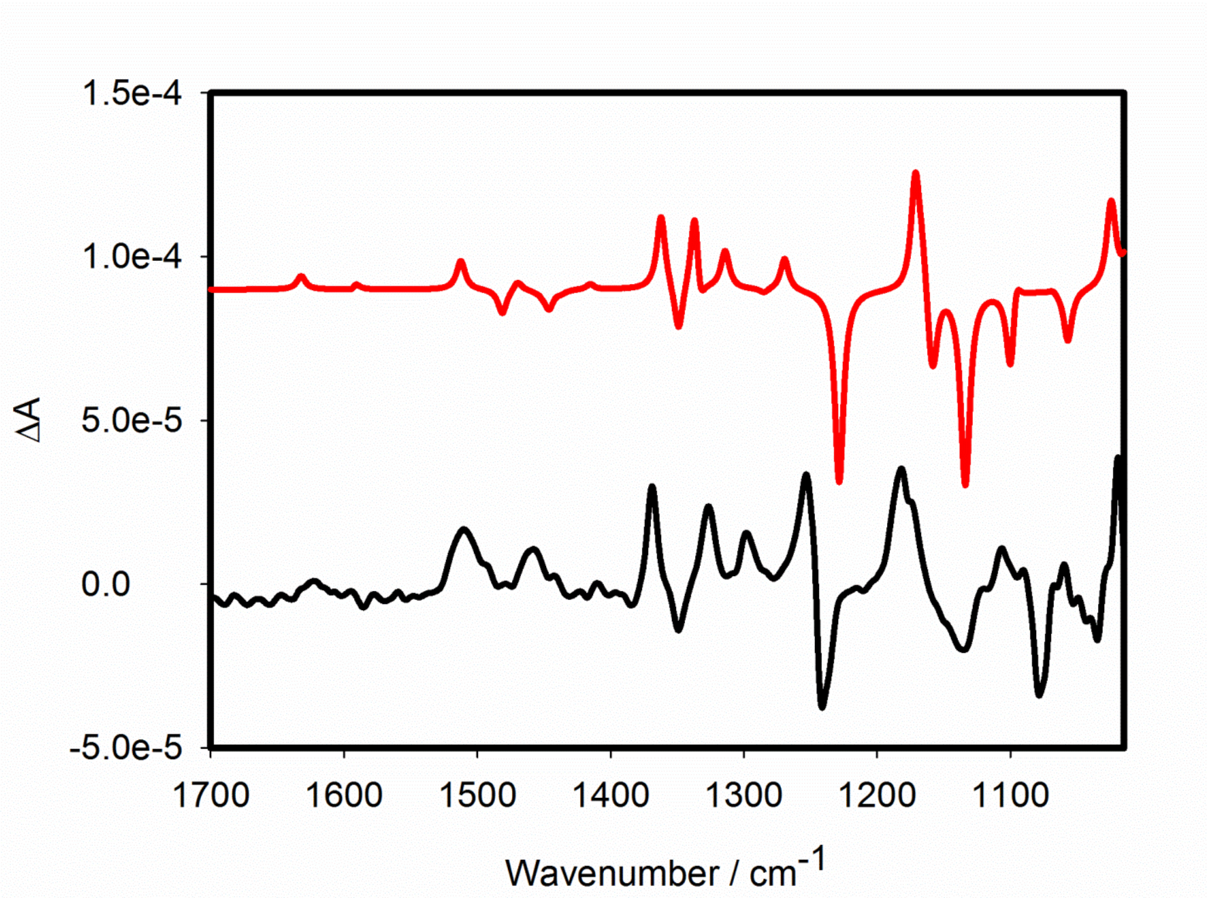

VCD spectra: black: experiment (8 microliter in 200 microliter CD_2Cl_2), red: calculated, mixture of 40% conformer 1 and 60% conformer 2.

3.2 (*R*)-2-(4-ethoxycarbonylphenyl)-2-methyl-2,3-dihydrofuran (4ap)

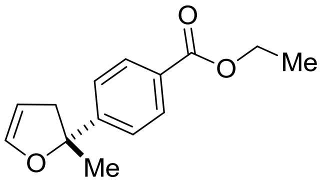

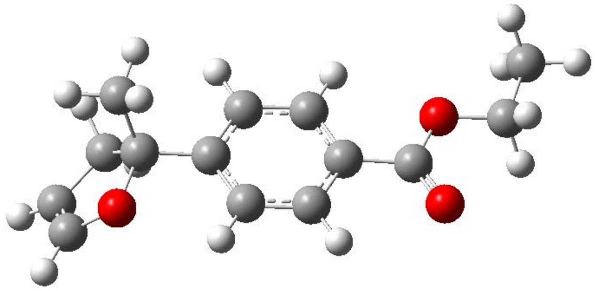

gustavo		
File Name	gustavob11	
File Type	.log	
Calculation Type	FREQ	
Calculation Method	RB3PW91	
Basis Set	6-31G(d,p)	
Charge	0	
Spin	Singlet	
E(RB3PW91)	-768.52500474	a.u.
RMS Gradient Norm	0.00000242	a.u.
Imaginary Freq	0	
Dipole Moment	2.9782	Debye
Point Group	C1	

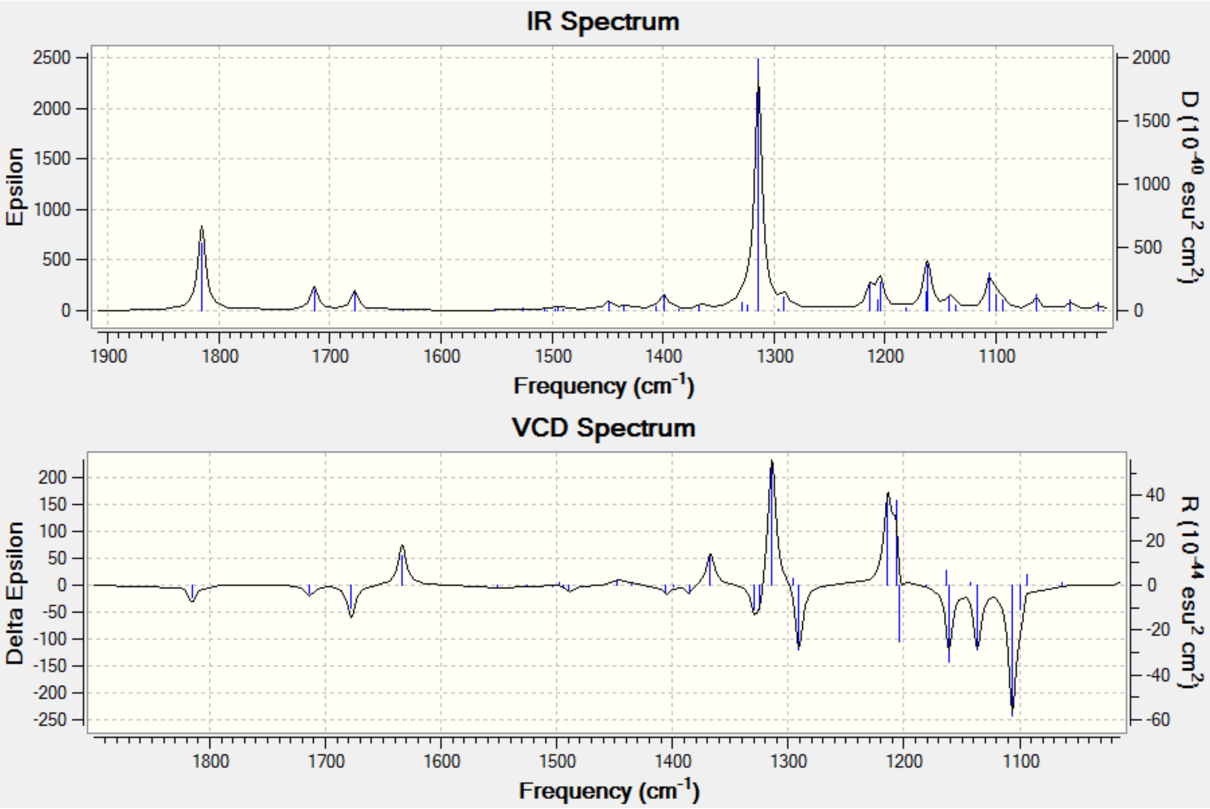

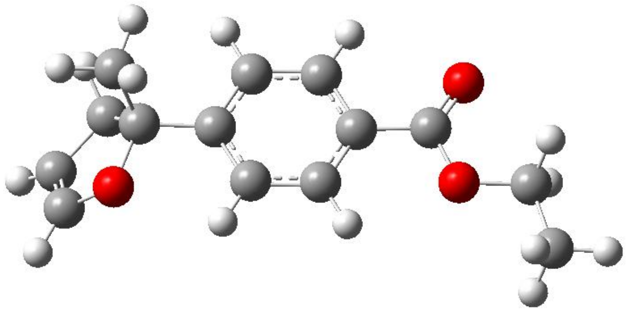

gustavo		
File Name	gustavob12	
File Type	.log	
Calculation Type	FREQ	
Calculation Method	RB3PW91	
Basis Set	6-31G(d,p)	
Charge	0	
Spin	Singlet	
E(RB3PW91)	-768.52516140	a.u.
RMS Gradient Norm	0.00000187	a.u.
Imaginary Freq	0	
Dipole Moment	1.6217	Debye
Point Group	C1	

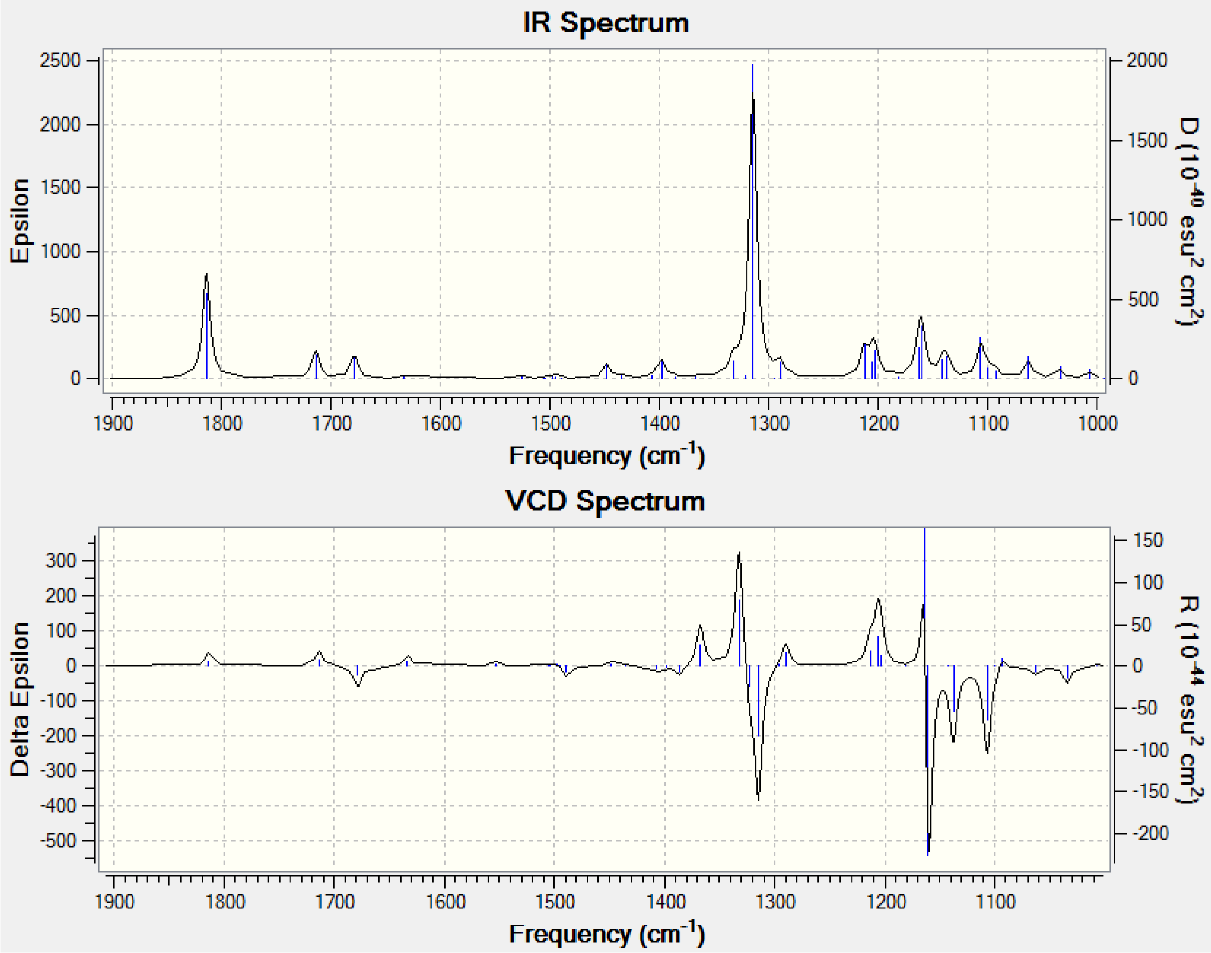

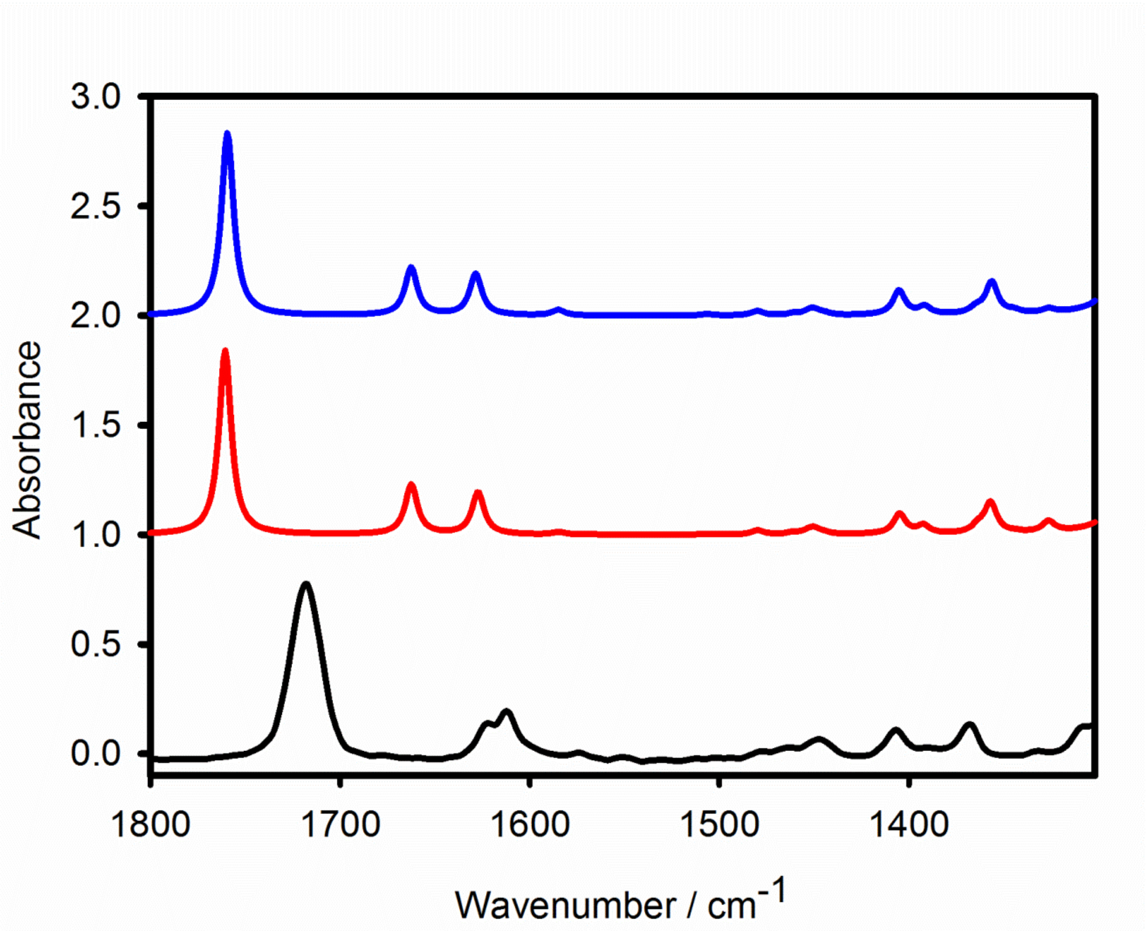

IR spectra: black: experiment (1 microliter in 200 microliter CD_2Cl_2), red and blue: calculated (two conformers).

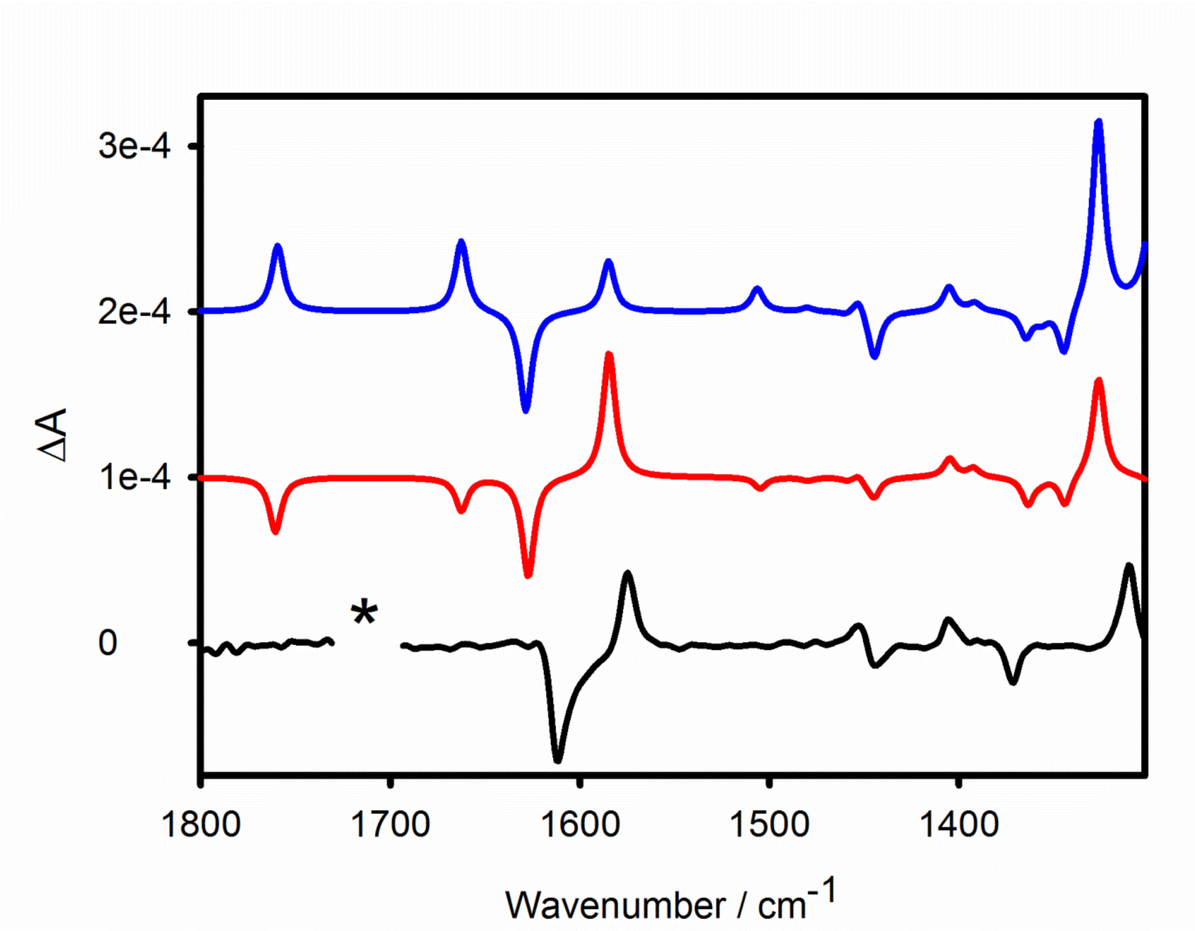

VCD spectra: black: experiment (3 microliter in 200 microliter CD_2Cl_2), red and blue: calculated (two conformers). The region marked by the asterisks could not be measured at this concentration due to strong absorption in this region.

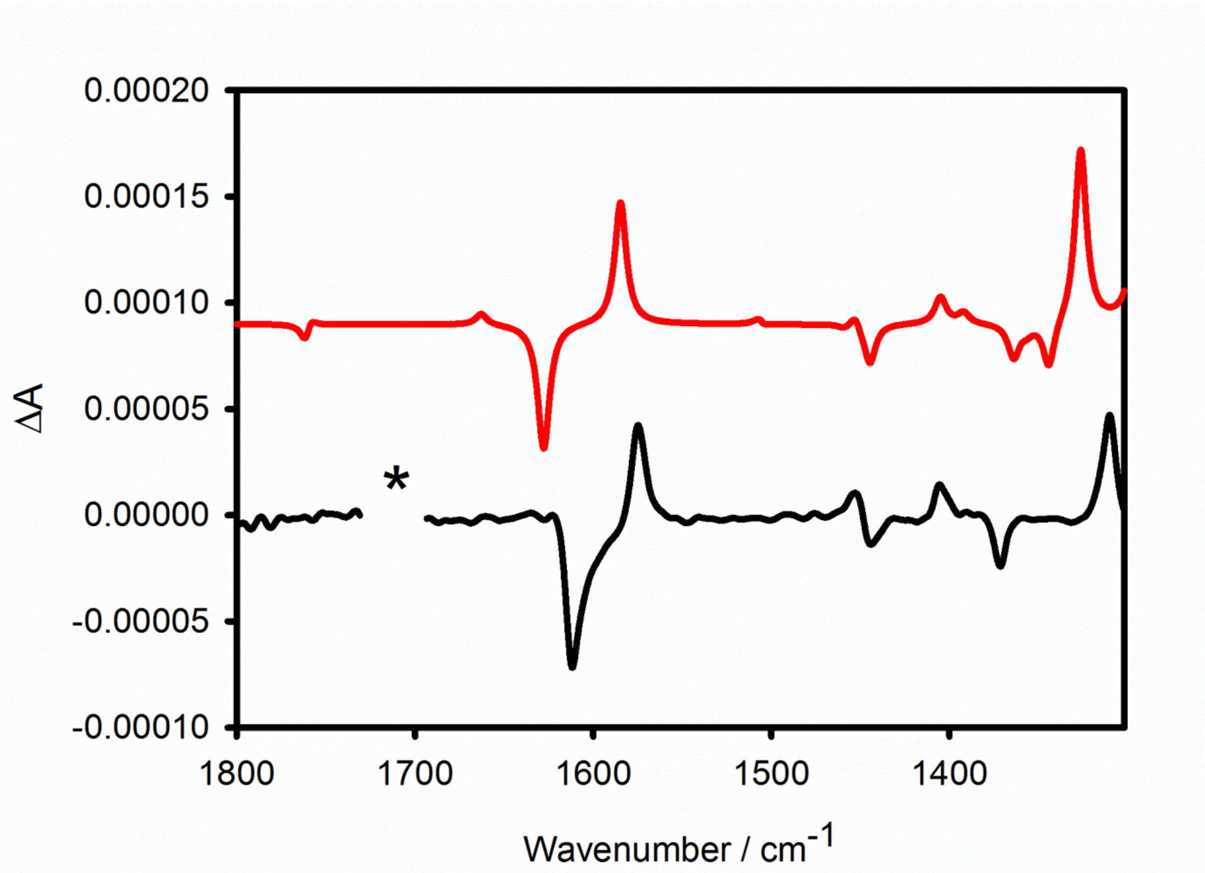

VCD spectra: black: experiment (3 microliter in 200 microliter CD_2Cl_2), red: calculated, mixture of 60% conformer 1 and 40% conformer 2.The region marked by the asterisks could not be measured at this concentration due to strong absorption in this region.

Conclusion: Quite good agreement between experiment and calculations for a mixture of both conformers. Absolute configuration of the measured sample is the same as the one of the calculated enantiomer.

4 Experimental procedures and characterization data

4.1 General procedure for the synthesis of 5-alkyl-2,3-dihydrofuran (in a 2-Me-THF solution) (GP1)

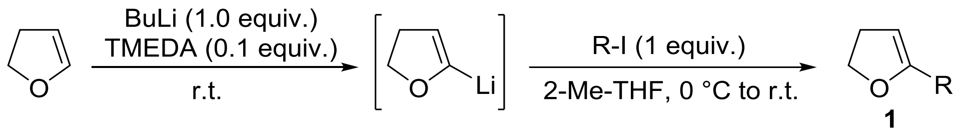

To a mixture of 2,3-dihydrofuran (2.72 mL, 36 mmol, 1 equiv.) and TMEDA (0.54 mL, 3.6 mmol, 0.1 equiv.) at room temperature in a water bath was added dropwise *n*-butyllithium (1.6 M, 22.5 mL, 36 mmol, 1 equiv.) in solution in hexane over 15 min. Over the course of addition, the reaction became light yellow with formation of an off-white precipitate. The reaction was then stirred 2 h at room temperature, then concentrated under reduced pressure and the resulting solid was dissolved in dried and degassed 2-Me-THF (5 mL). The resulting brown solution was cooled again to 0 °C and the appropriate alkyl iodide (36 mmol, 1 equiv.) was added dropwise over 15 min with vigorous stirring. The solution was stirred at room temperature for 3 h and then distilled to dryness using a dry ice cooled condenser to give a colorless liquid. The solution is then refluxed over sodium for 4 h while the mixture became blue. After stirring at room temperature overnight, the solution was distilled to give a colorless solution of 5-substituted-2,3-dihydrofuran **1** in 2-Me-THF as a colorless liquid. The concentration of **1** was determined by ^{1}H NMR using 1,3-di-tert-butyl-2-methoxy-5-methylbenzene as internal reference.

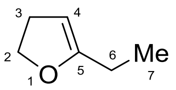

5-Ethyl-2,3-dihydrofuran (1b)

Prepared according to **GP1** using ethyl iodide as electrophile. Isolated by distillation (44% yield, 3.7 M in 2-Me-THF). **^{1}H-NMR (C_6D_6, 300 MHz)**: δ(ppm) = 1.00 (t, $^{3}J_{H\text{-}H}$ = 7.5 Hz, 3H, H-7), 2.06 (ddq, $^{3}J_{H\text{-}H}$ = 7.5 Hz, $^{4}J_{H\text{-}H}$ = 3.2 Hz, $^{5}J_{H\text{-}H}$ = 1.8 Hz, 2H, H-6), 2.32 (ddq, $^{3}J_{H\text{-}H}$ = 9.4 Hz, $^{3}J_{H\text{-}H}$ = 4.1 Hz, $^{5}J_{H\text{-}H}$ = 2.0 Hz, 2H, H-3), 4.09 (t, $^{3}J_{H\text{-}H}$ = 9.3 Hz, 2H, H-2), 4.44-4.46 (m, 1H, H-4); **^{13}C{^{1}H}-NMR (C_6D_6, 75 MHz):** δ(ppm) = 11.4 (C-7), 21.6 (C-6), 30.3 (C-3), 69.8 (C-2), 92.5 (C-4), 161.1 (C-5); **GC-MS (EI):** ($C_6H_{10}O$), 98.1 (100, M^+), 69.1 (30, M^+ – 29), 57.1 (86, M^+ – 41); **IR spectrum (2-MeTHF)** (cm^{-1}) = 2970, 1668, 1462, 1377, 1165, 1090, 1008, 932, 898, 717.

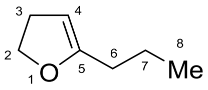

5-Propyl-2,3-dihydrofuran

Prepared according to **GP1** using propyl iodide as electrophile. Isolated by distillation (51% yield, 3.3 M in 2-Me-THF). **^{1}H-NMR (C_6D_6, 400 MHz)**: δ(ppm) = 0.85 (t, $^3J_{H-H}$ = 7.4 Hz, 3H, H-8), 1.52 (hex, $^3J_{H-H}$ = 7.4 Hz, 2H, H-7), 2.07 (t, $^3J_{H-H}$ = 7.5 Hz, 2H, H-6), 2.33 (m, 2H, H-3), 4.09 (t, $^3J_{H-H}$ = 9.3 Hz, 2H, H-2), 4.47-4.49 (m, 1H, H-4); **^{13}C{^{1}H}-NMR (C_6D_6, 100 MHz):** δ(ppm) = 13.9 (C-8), 20.4 (C-7), 26.2 (C-6 + C-3), 69.7 (C-2), 93.6 (C-4), 159.4 (C-5); **GC-MS (EI):** ($C_7H_{12}O$), 112.1 (41, M^+), 97.1 (92, M^+ – 15), 84.9 (27, M^+ – 28), 69.0 (26, M^+ – 43), 55.1 (100, M^+ – 57); **IR spectrum (2-MeTHF)** (cm^{-1}) = 2960, 1667, 1460, 1376, 1258, 1165, 1003, 963, 935, 719.

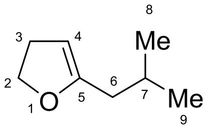

5-isobutyl-2,3-dihydrofuran (1c)

Prepared according to **GP1** using isobutyl iodide as electrophile. Isolated by distillation (25% yield, 2.0 M in 2-Me-THF). **^{1}H-NMR (C_6D_6, 400 MHz)**: δ(ppm) = 0.89 (d, $^3J_{H-H}$ = 6.5 Hz, 6H, H-8 + H-9), 1.89-1.96 (m, 1H, H-7), 1.98-1.99 (m, 2H, H-6), 2.30-2.36 (m, 2H, H-3), 4.07 (t, $^3J_{H-H}$ = 9.3 Hz, 2H, H-2), 4.47-4.48 (m, 1H, H-4); **^{13}C{^{1}H}-NMR (C_6D_6, 100 MHz):** δ(ppm) = 22.7 (C-8 + C-9), 26.5 (C-7), 30.4 (C-3), 37.6 (C-6), 69.7 (C-2), 94.7 (C-4), 158.6 (C-5); **GC-MS (EI):** ($C_8H_{14}O$), 126.1 (27, M^+), 111.1 (93, M^+ – 15), 84.1 (24, M^+ – 42), 69.1 (100, M^+ – 57), 55.1 (23, M^+ – 71); **IR spectrum (2-MeTHF)** (cm^{-1}) = 2960, 2278, 1667, 1457, 1330, 1171, 1089, 1005, 936, 812.

4.2 Procedures for the synthesis of 5-alkyl-2,3-dihydrofuran (neat)

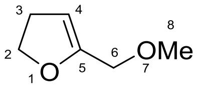

5-(methoxymethyl)-2,3-dihydrofuran (1d)

To a solution of sodium hydride (720 mg, 30 mmol, 2 equiv.) in dry THF (20 mL) at 0 °C was added dropwise 5-(methanolyl)-2,3-dihydrofuran (1.5 g, 15 mmol, 1 equiv.) over 5 min. The reaction was then stirred for 2 h at room temperature. The solution was cooled again to 0 °C and methyl iodide (1.9 mL, 30 mmol, 2 equiv.) was added dropwise over 15 min. The solution was stirred for 2 h at room temperature. The reaction was carefully quenched with water, extracted with diethyl ether, dried over magnesium sulfate, filtered and concentrated under reduced pressure. The residue was

distilled with Kugelrohr distillation set to afford 5-(methoxymethyl)-2,3-dihydrofuran (1.0 g, 60%) as a colorless oil.

^{1}H-NMR (C_6D_6, 400 MHz): δ (ppm) = 2.21-2.26 (m, 1H, H-3), 3.14 (s, 3H, H-8), 3.86 (m, 2H, H-6), 4.05 (t, $^3J_{H-H}$ = 9.4 Hz, 2H, H-2), 4.75-4.76 (m, 1H, H-4); **^{13}C{^{1}H}-NMR (C_6D_6, 100 MHz):** δ (ppm) = 30.1 (C-3), 58.1 (C-8), 67.5 (C-6), 70.1 (C-2), 97.2 (C-4), 156.2 (C-5); **GC-MS (EI):** ($C_6H_{10}O_2$), 114.1 (100, M$^+$), 99.0 (81), 81.2 (12), 72.1 (21), 58.1 (36); **IR spectrum (neat)** (cm^{-1}) = 2902, 1776, 1670, 1452, 1186, 1049, 999, 957, 926, 895, 721.

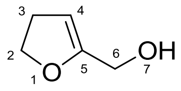

5-(methanolyl)-2,3-dihydrofuran (1e)

To a mixture of 2,3-dihydrofuran (3.8 mL, 50 mmol, 1 equiv.) in dry THF (50 mL) at room temperature in a water bath was added dropwise *n*-butyllithium (1.6 M, 31.3 mL, 50 mmol, 1 equiv.) in solution in hexane over 15 min. The reaction was then stirred 2 h at room temperature. The solution was cooled to 0 °C and paraformaldehyde (2.4 g, 80 mmol, 1.6 equiv.) was added portionwise over 5 min. The solution was then refluxed for 2 h. After cooling down to room temperature, iced water (10 mL) was added to the mixture. The organic phase was collected, while the aqueous phase was extracted 5 times with dichloromethane. Combined organic phases were dried over magnesium sulfate, filtered and concentrated under reduced pressure. The residue was distilled with Kugelrohr distillation set to afford 5-(methanolyl)-2,3-dihydrofuran (3.0 g, 60%) as a colorless oil.

^{1}H-NMR (C_6D_6, 400 MHz): δ (ppm) = 2.23-2.28 (m, 2H, H-3), 3.01 (bs, 1H, H-7), 4.05 (t, $^3J_{H-H}$ = 9.5 Hz, 2H, H-2), 4.07 (s, 2H, H-6), 4.73 (s, 1H, H-4); **^{13}C{^{1}H}-NMR (C_6D_6, 100 MHz):** δ (ppm) = 30.0 (C-3), 58.1 (C-6), 70.3 (C-2), 95.6 (C-4), 158.4 (C-5); **GC-MS (EI):** ($C_5H_8O_2$), 100.1 (6, M$^+$), 84.1 (100, M$^+$ – 16), 71.1 (10, M$^+$ – 29), 56.1 (18, M$^+$ – 44); **IR spectrum (neat)** (cm^{-1}) = 3419, 2877, 1674, 1263, 1179, 1058, 1024, 999, 929, 893, 732.

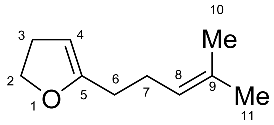

5-(4-methylpent-3-en-1-yl)-2,3-dihydrofuran (1f)

To a mixture of 2,3-dihydrofuran (1.13 mL, 15 mmol, 1 equiv.) and TMEDA (0.22 mL, 1.5 mmol, 0.1 equiv.) in dry THF (30 mL), *n*-butyllithium (1.6 M, 9.4 mL, 15 mmol, 1 equiv.) in solution in hexane

was added dropwise over 15 min at room temperature. The reaction was stirred for 2 h at room temperature. The solution was cooled to 0 °C and 5-bromo-2-methylpent-2-ene[6] (1 mL, 7.5 mmol, 0.5 equiv.) was added dropwise with vigorous stirring over 5 min. The solution was stirred at room temperature overnight. The remaining traces of 5-bromo-2-methylpent-2-ene were removed by refluxing the solution over small pieces of sodium for 4 h. Then the reaction was diluted with diethyl ether (20 mL), filtered over a short pad of Celite® and concentrated under reduced pressure. The residue was distilled with Kugelrohr distillation set to afford 5-(4-methylpent-3-en-1-yl)-2,3-dihydrofuran (520 mg, 46%) as a colorless oil.

^{1}H-NMR (C_6D_6, 400 MHz): δ (ppm) = 1.52 (s, 3H, H-10), 1.63 (s, 3H, H-11), 2.19-2.23 (m, 2H, H-6), 2.26-2.36 (m, 4H, H-7 + H-3), 4.09 (t, $^3J_{H-H}$ = 9.3 Hz, 2H, H-2), 4.51-4.52 (m, 1H, H-4), 5.17-5.21 (m, 1H, H-8); **^{13}C{^{1}H}-NMR (C_6D_6, 100 MHz):** δ (ppm) = 17.7 (C-10), 25.8 (C-11), 25.9 (C-7), 28.7 (C-6), 30.4 (C-3), 69.8 (C-2), 93.7 (C-4), 124.4 (C-8), 131.8 (C-9), 159.3 (C-5); **GC-MS (EI):** ($C_{10}H_{16}O$), 152.1 (35, M^+), 137.1 (25, M^+ – 15), 84.1 (77, M^+ – 68), 69.1 (100, M^+ – 83), 55.1 (29, M^+ – 97); **IR spectrum (neat)** (cm^{-1}) = 2917, 1667, 1445, 1376, 1246, 1162, 1003, 958, 930, 902, 718.

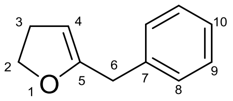

5-benzyl-2,3-dihydrofuran (1g)

To a mixture of 2,3-dihydrofuran (1.13 mL, 15 mmol, 1 equiv.) and TMEDA (0.22 mL, 1.5 mmol, 0.1 equiv.) in dry THF (30 mL) at room temperature in a water bath was added dropwise *n*-butyllithium (1.6 M, 9.4 mL, 15 mmol, 1 equiv.) in solution in hexane over 15 min. The reaction was then stirred for 2 h at room temperature. The solution was cooled to 0 °C and benzyl bromide (1.8 mL, 15 mmol, 1 equiv.) was added dropwise with vigorous stirring over 15 min. Then, the solution was stirred at room temperature overnight. The remaining traces of benzyl bromide were removed by refluxing the solution over small pieces of sodium for 4 h. Then the reaction was diluted with diethyl ether (20 mL), filtered over a short pad of Celite® and concentrated under reduced pressure. The residue was distilled with Kugelrohr distillation set to afford 5-benzyl-2,3-dihydrofuran (1.2 g, 48%, 95% purity) as a colorless oil.

^{1}H-NMR (C_6D_6, 400 MHz): δ (ppm) = 2.22-2.28 (m, 2H, H-3), 3.34 (s, 2H, H-6), 4.03 (t, $^3J_{H-H}$ = 9.3 Hz, 2H, H-2), 4.41-4.42 (m, 1H, H-4), 7.04-7.08 (m, 1H, H-10), 7.12-7.16 (m, 2H, H-9), 7.20-7.21 (m, 2H, H-8); **^{13}C{^{1}H}-NMR (C_6D_6, 100 MHz):** δ (ppm) = 30.3 (C-3), 35.1 (C-6), 70.1 (C-2), 95.4 (C-4), 126.6 (C-10),

6 Prepared according to B. D. Schwartz, D. P. Tilly, R. Heim, S. Wiedemann, C. M. Williams and P. V. Bernhardt, *Eur. J. Org. Chem.* 2006, 3181.

128.6 (C-9), 129.4 (C-8), 138.4 (C-7), 158.5 (C-5); **GC-MS (EI):** ($C_{11}H_{12}O$), 160.1 (100, M^+), 118.0 (100, $M^+ - 42$), 104.1 (45, $M^+ - 56$), 90.1 (88, $M^+ - 70$); **IR spectrum (neat)** (cm^{-1}) = 2895, 1666, 1494, 1454, 1375, 1256, 1156, 1002, 944, 699.

4.3 General procedure for the asymmetric intermolecular Heck reaction with neat 5-alkyl-2,3-dihydrofurans (GP2)

In a glovebox, a 5 mL Young valve Schlenk was charged with $Pd_2(dba)_3$ (4.57 mg, 0.005 mmol, 2.5 mol%), the appropriate chiral ligand (0.02 mmol, 10 mol%) and 0.68 mL of degassed 2-Me-THF. The Schlenk was taken outside the glovebox, connecting to a two-manifold line and the mixture was stirred at r.t. for 15 min. Next, $PhCF_3$ (i.e. internal standard; 29.2 mg, 0.2 mmol, 1 equiv.), ArOTf (0.2 mmol, 1 equiv.), $^iPr_2NEt$ (0.105 ml, 0.6 mmol, 3 equiv.) and neat 5-alkyl-2,3-dihydrofuran (1 mmol, 5 equiv.) were added consecutively under a flow of N_2 gas. The sealed reaction tube was immerged in a 100 °C pre-heated oil bath for 48-72 h. After cooling to r.t., the reaction mixture was poured into pentane (5 mL) under vigorous stirring, and the resulting precipitate was removed passing the solution trough a short pad of Celite®. The filtrate was concentrated to dryness to give an oil which was directly subjected to flash chromatography (Cyclohexane:AcOEt).

The **large scale experiment** described on Figure 2 of the manuscript was performed according to GP2 using 5-methyl-2,3-dihydrofuran (3.87 mL, 42.5 mmol, 5 equiv.), 4-cyanophenyl trifluoromethanesulfonate (2.13 g, 8.5 mmol, 1 equiv.), $Pd_2(dba)_3$ (192 mg, 0.21 mmol, 2.5 mol%), (*R*)-**L2** (518 mg, 0.85 mmol, 10 mol%), $^iPr_2NEt$ (4.44 ml, 25.5 mmol, 3 equiv.) in 2-Me-THF (29 mL) and the reaction run for 60 h.

4.4 General procedure for the asymmetric intermolecular Heck reaction with 5-alkyl-2,3-dihydrofuran in a 2-Me-THF solution (GP3)

For 5-alkyl-2,3-dihydrofurans in 2-Me-THF solutions the overall concentration of the reaction is maintained to 0.3 M (i.e. volume of the 5-alkyl-2,3-dihydrofuran in 2-Me-THF + volume of 2-Me-THF added in the glovebox to prepare the catalyst, V_{Tot} = 0.68 mL).

4.5 Characterization data of 2-aryl-2-methyl-2,5-dihydrofurans

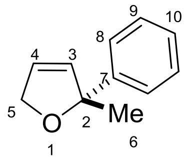

(*R*)-2-methyl-2-phenyl-2,5-dihydrofuran (3aa)

Prepared according to **GP2** using 5-methyl-2,3-dihydrofuran (**1a**). Isolated by column chromatography (Cyclohexane:AcOEt 80:1) as a pale yellow oil (27% yield, 92% *ee*) with R_F = 0.67 (Cyclohexane:AcOEt 10:1); **^{1}H-NMR (C_6D_6, 400 MHz)**: δ (ppm) = 1.63 (s, 3H, H-6), 4.53 (ddd, $^2J_{H-H}$ = 12.9 Hz, $^3J_{H-H}$ = 2.4 Hz, $^4J_{H-H}$ =1.6 Hz, 2H, H-5), 4.55 (ddd, $^2J_{H-H}$ = 12.9 Hz, $^3J_{H-H}$ = 2.4 Hz, $^4J_{H-H}$ = 1.6 Hz, 1H, H-5´), 5.39 (dt, $^3J_{H-H}$ = 6.0 Hz, $^4J_{H-H}$ = 1.6 Hz, 1H, H-3), 5.72 (dt, $^3J_{H-H}$ = 6.0 Hz, $^3J_{H-H}$ = 2.4 Hz, 1H, H-4), 6.83 (m, 2H, H-9), 7.35 (m, 2H, H-8); **^{13}C{^{1}H}-NMR (C_6D_6, 100 MHz):** 28.8 (C-6), 74.7 (C-5), 90.8 (C-2), 125.10 (C-3), 125.15 (C-8), 126.89 (C-10), 128.50 (C-9), 134.55 (C-4), 147.36 (C-7); **MS (ESI)**: $C_{11}H_{12}OLi$, 167.1 $[M+Li]^+$; **IR spectrum (neat)** (cm^{-1}) = 2973, 2925, 2849, 1600, 1492, 1444, 1367, 1347, 1237, 1134, 1083, 1067, 1017, 904, 863, 762, 696, 710; $[\alpha]^{23}_D$ = + 108.2 (*c* 0.83, CH_2Cl_2); **HPLC:** OJ-H, 220 nm, Hexane:*i*PrOH, 98:2, 1 mL/min, 30 °C, t_{R1} = 10.7 and t_{R2} = 12.6 min.

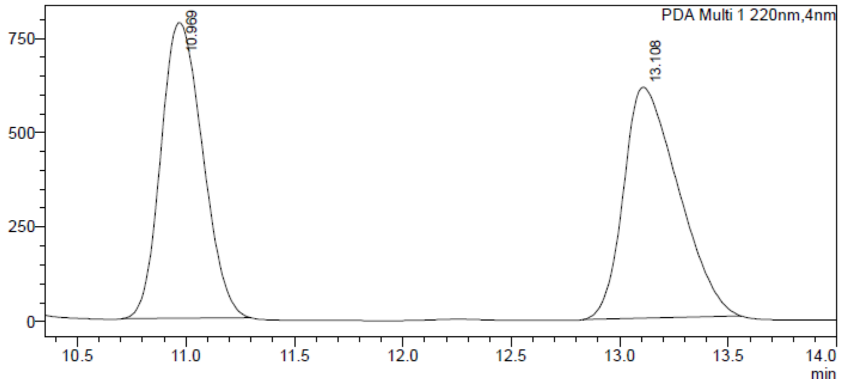

<Peak Table>

PDA Ch1 220nm

Peak#	Ret. Time	Area%
1	10.969	49.336
2	13.108	50.664
Total		100.000

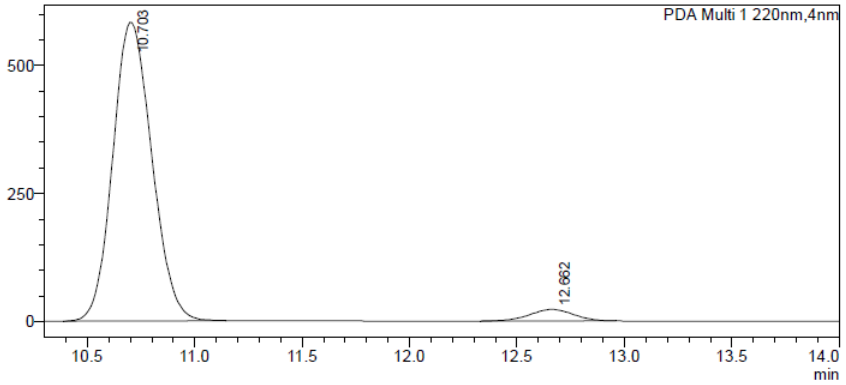

<Peak Table>

PDA Ch1 220nm

Peak#	Ret. Time	Area%
1	10.703	95.865
2	12.662	4.135
Total		100.000

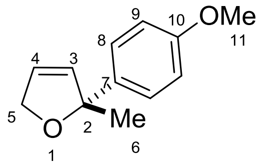

(*R*)-2-(4-methoxyphenyl)-2-methyl-2,5-dihydrofuran (3ab)

Prepared according to **GP2** using 5-methyl-2,3-dihydrofuran (**1a**). Isolated by column chromatography (Cyclohexane:AcOEt 60:1) as a pale yellow oil (64% yield, 93% *ee*) with R_F = 0.67 (Cyclohexane:AcOEt 10:1); **^{1}H-NMR (C_6D_6, 400 MHz)**: δ (ppm) = 1.63 (s, 3H, H-6), 3.32 (s, 3H, H-11), 4.53 (ddd, $^2J_{H\text{-}H}$ = 12.9 Hz, $^3J_{H\text{-}H}$ = 2.4 Hz, $^4J_{H\text{-}H}$ =1.6 Hz, 2H, H-5), 4.55 (ddd, $^2J_{H\text{-}H}$ = 12.9 Hz, $^3J_{H\text{-}H}$ = 2.4 Hz, $^4J_{H\text{-}H}$ = 1.6 Hz, 1H, H-5´), 5.39 (dt, $^3J_{H\text{-}H}$ = 6.0 Hz, $^4J_{H\text{-}H}$ = 1.6 Hz, 1H, H-3), 5.72 (dt, $^3J_{H\text{-}H}$ = 6.0 Hz, $^3J_{H\text{-}H}$ = 2.4 Hz, 1H, H-4), 6.83 (m, 2H, H-9), 7.35 (m, 2H, H-8); **^{13}C{^{1}H}-NMR (C_6D_6, 100 MHz):** δ (ppm) = 28.7 (C-6), 54.8 (C-11), 74.6 (C-5), 90.5 (C-2), 113.9 (C-9), 124.8 (C-3), 126.4 (C-8), 134.9 (C-4), 139.4 (C-7), 159.0 (C-10); **HRMS** (ESI positive) calculated for $C_{12}H_{15}O_2$, 191.1067 $[M+H]^+$, found 191.1058; **IR spectrum (neat)** (cm^{-1}) = 2969, 1610, 1509, 1457, 1299, 1241, 1176, 1017, 829, 704; $[\alpha]^{23}_D$ = + 137.1 (*c* 0.63, CH_2Cl_2); **HPLC:** OJ-H, 220 nm, Hexane:*i*PrOH, 98:2, 1 mL/min, 30 °C, t_{R1} = 27.6 and t_{R2} = 41.6 min.

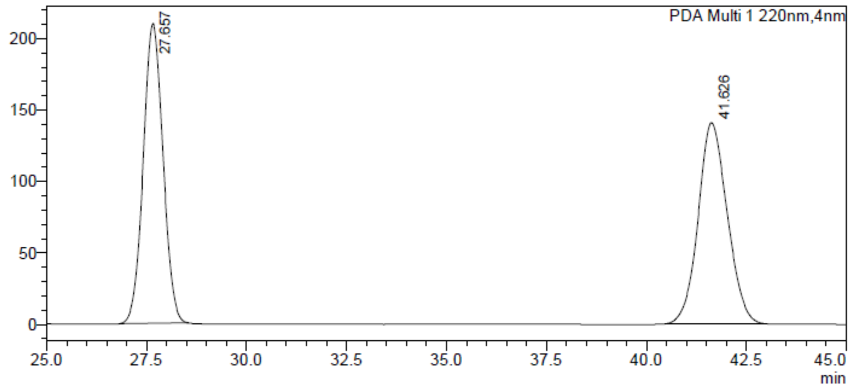

<Peak Table>

PDA Ch1 220nm

Peak#	Ret. Time	Area%
1	27.657	50.313
2	41.626	49.687
Total		100.000

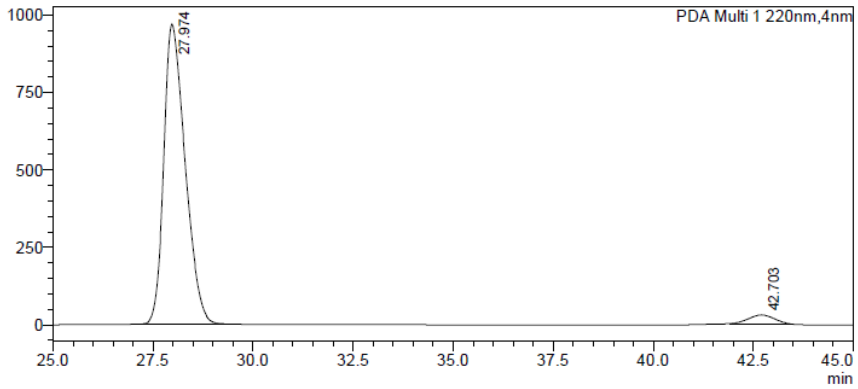

<Peak Table>

PDA Ch1 220nm

Peak#	Ret. Time	Area%
1	27.974	96.720
2	42.703	3.280
Total		100.000

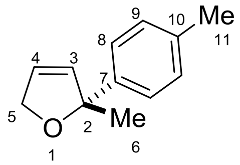

(*R*)-2-methyl-2-(p-tolyl)-2,5-dihydrofuran (3ad)

Prepared according to **GP2** using 5-methyl-2,3-dihydrofuran (**1a**). Isolated by column chromatography (Cyclohexane:AcOEt 70:1) as a pale yellow oil (47% yield, 92% *ee*) with R_F = 0.58 (Cyclohexane:AcOEt 10:1); **^{1}H-NMR (C_6D_6, 400 MHz)** δ (ppm) = 1.62 (s, 3H, H-6), 2.13 (s, 3H, H-11), 4.52 (ddd, $^2J_{H-H}$ = 12.9 Hz, $^3J_{H-H}$ = 2.4 Hz, $^4J_{H-H}$ = 1.7 Hz, 1H, H-5), 4.59 (ddd, $^2J_{H-H}$ = 12.9 Hz, $^3J_{H-H}$ = 2.4 Hz, $^4J_{H-H}$ = 1.7 Hz, 1H, H-5´), 5.37 (dt, $^3J_{H-H}$ = 6.0 Hz, $^4J_{H-H}$ = 1.6 Hz, 1H, H-3), 5.73 (dt, $^3J_{H-H}$ = 6.0 Hz, $^3J_{H-H}$ = 2.4 Hz, 1H, H-4), 7.04 (m, 2H, H-9), 7.36 (m, 2H, H-8); **^{13}C{^{1}H}-NMR (C_6D_6, 100 MHz):** δ (ppm) = 21.0 (C-11), 28.8 (C-6), 74.7 (C-5), 90.7 (C-2), 124.9 (C-3), 125.1 (C-8 or C-9), 128.7 (C-8 or C-9), 134.8 (C-4), 136.1 (C-10), 144.5 (C-7); **HRMS** (ESI positive) calculated for $C_{12}H_{13}O$, 173.0961 $[M-H]^+$, found 173.0939; **IR spectrum (neat)** (cm^{-1}) =2973, 1511, 1240, 1078, 1017, 814, 726, 701; $[\alpha]^{23}_D$ = + 240.7 (*c* 0.23, CH_2Cl_2); **HPLC :** OJ-H, 204 nm, Hexane:*i*PrOH, 98:2, 1 mL/min, 30 °C, t_{R1} = 10.8 and t_{R2} = 14.3 min.

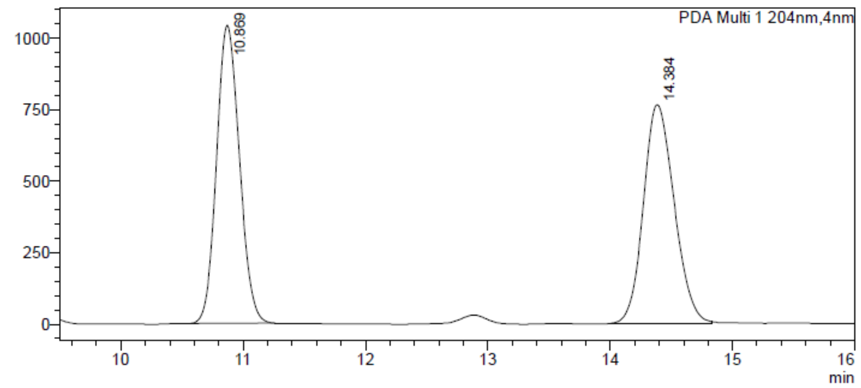

<Peak Table>

PDA Ch1 204nm

Peak#	Ret. Time	Area%
1	10.869	50.281
2	14.384	49.719
Total		100.000

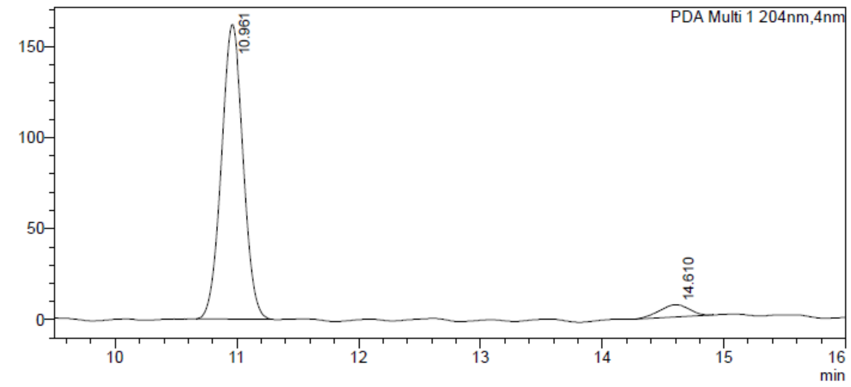

<Peak Table>

PDA Ch1 204nm

Peak#	Ret. Time	Area%
1	10.961	94.478
2	14.610	5.522
Total		100.000

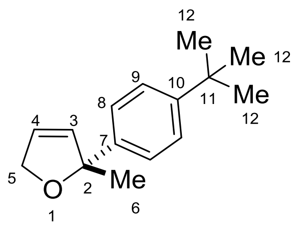

(*R*)-2-(4-(tert-butyl)phenyl)-2-methyl-2,5-dihydrofuran (3ae)

Prepared according to **GP2** using 5-methyl-2,3-dihydrofuran (**1a**). Isolated by column chromatography (Cyclohexane:AcOEt 60:1) as a pale yellow oil (65 % yield, 89% *ee*) with R_F = 0.50 (Cyclohexane:AcOEt 10:1); **^{1}H-NMR(C_6D_6, 400 MHz):** δ (ppm) = 1.24 (s, 9H, H-12), 1.65 (s, 3H, H-6), 4.54 (ddd, $^2J_{H-H}$ = 12.9 Hz, $^3J_{H-H}$ = 2.4 Hz, $^4J_{H-H}$ = 1.6 Hz, 1H, H-5), 4.61 (ddd, $^2J_{H-H}$ = 12.9 Hz, $^3J_{H-H}$ = 2.4 Hz, $^4J_{H-H}$ = 1.6 Hz, 1H, H-5'), 5.38 (dt, $^3J_{H-H}$ = 6.0 Hz, $^4J_{H-H}$ = 1.6 Hz, 1H, H-3), 5.76 (dt, $^3J_{H-H}$ = 6.0 Hz, $^3J_{H-H}$ = 2.4 Hz, 1H, H-4), 7.32 (m, 2H, H-9), 7.43 (m, 2H, H-8); **^{13}C{^{1}H}-NMR(C_6D_6, 100 MHz)**: δ (ppm) = 28.6 (C-6), 31.2 (C-12), 34.1 (C-11), 74.5 (C-5), 90.5 (C-2), 124.7 (C-3), 124.7 (C-8), 125.1 (C-9), 134.5 (C-4), 144.2 (C-7), 149.1 (C-11); **HRMS** (ESI positive) calculated for $C_{15}H_{21}O$, 217.1587 $[M+H]^+$, found 217.1596; **IR spectrum (neat)** (cm^{-1}) = 2961, 1082, 1015, 836, 727, 702, 569; $[\alpha]^{23}_D$ = +84.7 (*c* 0.62, CH_2Cl_2); **HPLC:** IC, 203 nm, Hexane:*i*PrOH, 98:2, 1 mL/min, 30 °C, t_{R1} = 4.2 and t_{R2} = 5.7 min.

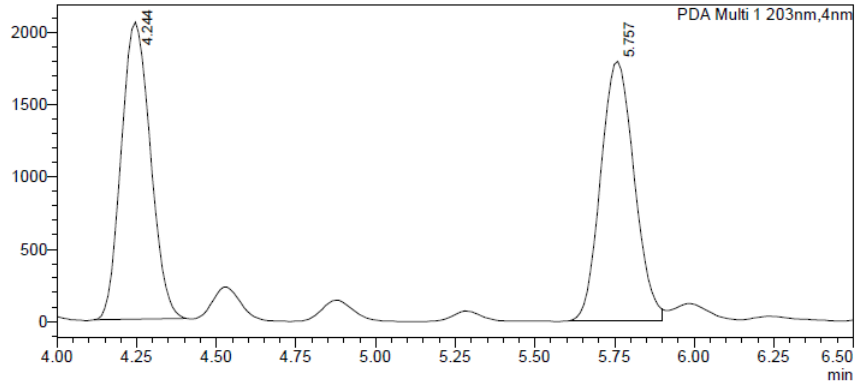

<Peak Table>

PDA Ch1 203nm

Peak#	Ret. Time	Area%
1	4.244	49.868
2	5.757	50.132
Total		100.000

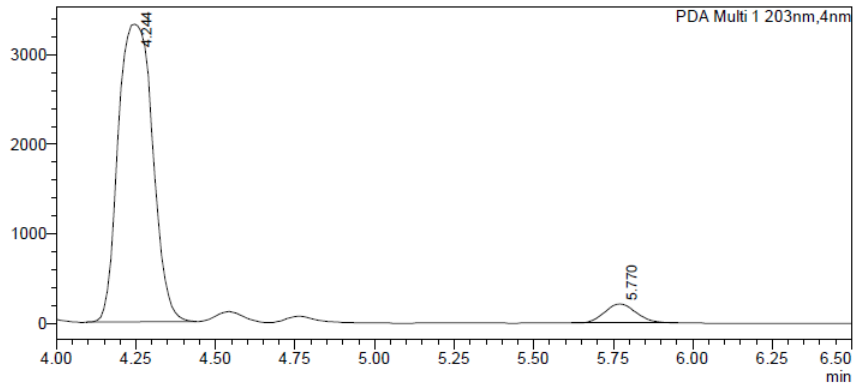

<Peak Table>

PDA Ch1 203nm

Peak#	Ret. Time	Area%
1	4.244	49.984
2	5.757	50.016
Total		100.000

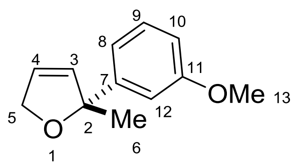

(*R*)-2-(3-methoxyphenyl)-2-methyl-2,5-dihydrofuran (3af)

Prepared according to **GP2** using 5-methyl-2,3-dihydrofuran (**1a**). Isolated by column chromatography (Cyclohexane:AcOEt 60:1) as a pale yellow oil (48% yield, 88% *ee*) with R_F = 0.50 (Cyclohexane:AcOEt 10:1); **^{1}H-NMR (C_6D_6, 400 MHz):** δ (ppm) = 1.61 (s, 3H, H-6), 3.36 (s, 3H, H-13), 4.50 (ddd, $^2J_{H\text{-}H}$ = 12.9 Hz, $^3J_{H\text{-}H}$ = 2.4 Hz, $^4J_{H\text{-}H}$ = 1.6 Hz, 1H, H-5), 4.57 (ddd, $^2J_{H\text{-}H}$ = 12.9 Hz, $^3J_{H\text{-}H}$ = 2.4 Hz, $^4J_{H\text{-}H}$ = 1.6 Hz, 1H, H-5´), 5.35 (dt, $^3J_{H\text{-}H}$ = 6.0 Hz, $^4J_{H\text{-}H}$ = 1.6 Hz, 1H, H-3), 5.72 (dt, $^3J_{H\text{-}H}$ = 6.0 Hz, $^3J_{H\text{-}H}$ = 2.4 Hz, 1H, H-4), 6.70 (ddd, $^3J_{H\text{-}H}$ = 8.1 Hz, $^4J_{H\text{-}H}$ = 2.6 Hz,$^4J_{H\text{-}H}$ = 0.9 Hz ,1H, H-8), 7.01 (ddd, $^3J_{H\text{-}H}$ = 7.7 Hz, $^4J_{H\text{-}H}$ = 1.6 Hz, $^4J_{H\text{-}H}$ = 1.0 Hz, 1H, H-10), 7.14 (t, $^3J_{H\text{-}H}$ = 7.9 Hz, 1H, H-9), 7.24 (dd, $^4J_{H\text{-}H}$ = 2.6 Hz, $^4J_{H\text{-}H}$ = 1.6 Hz, 1H, H-12); **^{13}C{^{1}H}-NMR(C_6D_6, 100 MHz):** δ (ppm) = 28.6 (C-6), 54.3 (C-13), 74.3 (C-5), 90.5 (C-2), 110.9 (C-9), 111.8 (C-8), 117.1 (C-10), 124.7 (C-3), 129.1 (C-12), 134.1 (C-4), 148.7 (C-7), 160.0 (C-11); **HRMS** (ESI positive) calculated for $C_{12}H_{14}O_2Li$, 197.1148 $[M+Li]^+$, found 197.1144; **IR spectrum (neat)** (cm^{-1}) = 2969, 1601, 1583, 1483, 1432, 1264, 1209, 1080, 1044, 1018, 782, 717, 695; $[\alpha]^{23}_D$ = + 72.6 (*c* 0.55, CH_2Cl_2); **HPLC:** OJ-H, 209 nm, Hexane:*i*PrOH, 98:2, 1 mL/min, 30 °C, t_{R1} = 15.2 and t_{R2} = 17.3 min.

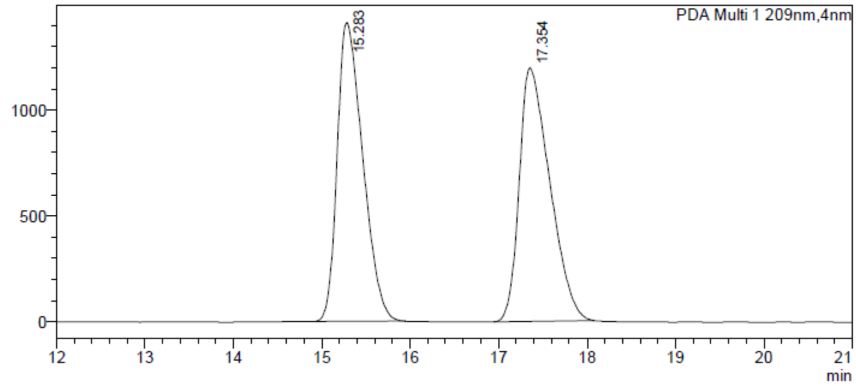

<Peak Table>

PDA Ch1 209nm

Peak#	Ret. Time	Area%
1	15.283	49.656
2	17.354	50.344
Total		100.000

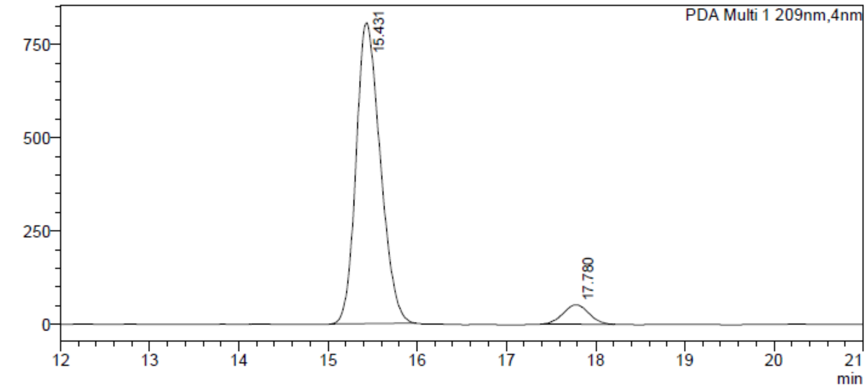

<Peak Table>

PDA Ch1 209nm

Peak#	Ret. Time	Area%
1	15.431	93.618
2	17.780	6.382
Total		100.000

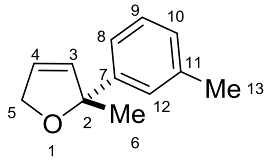

(*R*)-2-methyl-2-(m-tolyl)-2,5-dihydrofuran (3ag)

Prepared according to **GP2** using 5-methyl-2,3-dihydrofuran (**1a**). Isolated by column chromatography (Cyclohexane:AcOEt 75:1) as a pale yellow oil (37% yield, 91% *ee*) with R_F = 0.52 (Cyclohexane:AcOEt 10:1); **^{1}H-NMR (C_6D_6, 400 MHz)**: δ (ppm) = 1.63 (s, 3H, H-6), 2.17 (s, 3H, H-13), 4.53 (ddd, $^2J_{H\text{-}H}$ = 12.9 Hz, $^3J_{H\text{-}H}$ = 2.4 Hz, $^4J_{H\text{-}H}$ = 1.6 Hz, 2H, H-5), 4.60 (ddd, $^2J_{H\text{-}H}$ = 12.9 Hz, $^3J_{H\text{-}H}$ = 2.4 Hz, $^4J_{H\text{-}H}$ = 1.6 Hz, 1H, H-5´), 5.36 (dt, $^3J_{H\text{-}H}$ = 6.0 Hz, $^4J_{H\text{-}H}$ = 1.6 Hz, 1H, H-3), 5.72 (dt, $^3J_{H\text{-}H}$ = 6.0 Hz, $^3J_{HH}$ = 2.5 Hz, 1H, H-4), 6.93 (m, 1H, H-10), 7.26 (m, 1H, H-8), 7.35 (m, 1H, H-12); **^{13}C{^{1}H}-NMR (C_6D_6, 100 MHz)**: δ (ppm) = 21.6 (C-13), 29.0 (C-6), 74.7 (C-5), 90.9 (C-2), 122.3 (C-8), 125.0 (C-3), 125.8 (C-12), 127.6 (C-10), 128.4 (C-9), 134.6 (C-4), 137.8 (C-11), 147.3 (C-7); **HRMS** (ESI positive) calculated for $C_{12}H_{13}O$ 173.0961 $[M\text{-}H]^+$, found 173.0936; **IR spectrum (neat)** (cm^{-1}) = 2974, 2847, 1606, 1485, 1346, 1260, 1189, 1076, 1018, 784, 715, 698; $[\alpha]^{23}_D$ = + 93.5 (*c* 0.55, CH_2Cl_2); **HPLC:** OJ-H, 203 nm, Hexane:*i*PrOH, 99.5:0.5, 1 mL/min, 30 °C, t_{R1} = 10.2 and t_{R2} = 11.5 min.

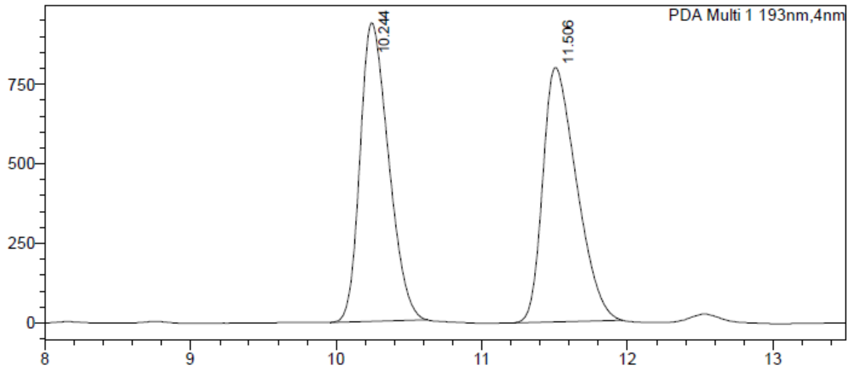

<Peak Table>

PDA Ch1 193nm

Peak#	Ret. Time	Area%
1	10.244	49.727
2	11.506	50.273
Total		100.000

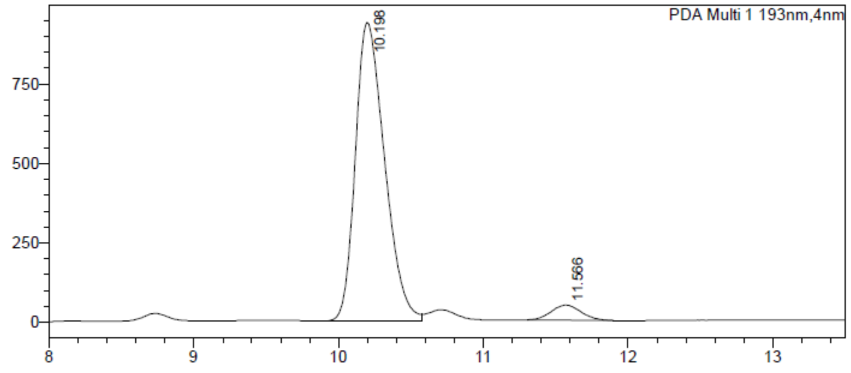

<Peak Table>

PDA Ch1 193nm

Peak#	Ret. Time	Area%
1	10.198	95.103
2	11.566	4.897
Total		100.000

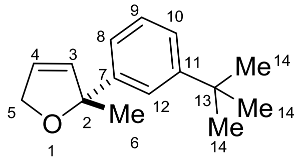

(*R*)-2-(3-(tert-butyl)phenyl)-2-methyl-2,5-dihydrofuran (3ah)

Prepared according to **GP2** using 5-methyl-2,3-dihydrofuran (**1a**). Isolated by column chromatography (Cyclohexane:AcOEt 60:1) as a pale yellow oil (56% yield, 90% *ee*) with R_F = 0.62 (Cyclohexane:AcOEt 10:1); **^{1}H-NMR (C_6D_6, 400 MHz)**: δ (ppm) = 1.27 (s, 9H, H-14), 1.65 (s, 3H, H-6), 4.55 (ddd, $^2J_{H\text{-}H}$ = 13.0 Hz, $^3J_{H\text{-}H}$ = 2.4 Hz, $^4J_{H\text{-}H}$ = 1.6 Hz, 1H, H-5), 4.62 (ddd, $^2J_{H\text{-}H}$ = 13.0 Hz, $^3J_{H\text{-}H}$ = 2.4 Hz, $^4J_{H\text{-}H}$ = 1.6 Hz, 1H, H-5´), 5.38 (dt, $^3J_{H\text{-}H}$ = 6.0 Hz, $^4J_{H\text{-}H}$ = 1.6 Hz, 1H, H-3), 5.79 (dt, $^3J_{H\text{-}H}$ = 6.0 Hz, $^3J_{H\text{-}H}$ =2.4 Hz, 1H, H-4), 7.21-7.25 (m, 3H, H-8, H-10 and H-12), 7.70 (m, 1H, H-9); **^{13}C{^{1}H}-NMR (C_6D_6, 100 MHz):** δ (ppm) = 28.9 (C-6), 31.3 (C-14), 34.6 (C-13), 74.4 (C-5), 90.4 (C-2), 121.5 (C-9), 122.2, 123.7 and 128.0 (C-8, C-10 and C-12), 124.8 (C-3), 134.5 (C-4), 146.8 (C-7), 150.9 (C-11); **HRMS** (ESI positive) calculated for $C_{15}H_{21}O$, 217.1587 $[M+H]^+$, found 217.1581; **IR spectrum (neat)** (cm^{-1}) = 2962, 1601, 1364, 1225, 1077, 1019, 794, 732, 708; $[\alpha]^{23}_D$ = + 96.4 (*c* 0.61, CH_2Cl_2); **HPLC:** IC, 191 nm, Hexane:*i*PrOH, 99.5:0.5, 1 mL/min, 30 °C, t_{R1} = 4.5 and t_{R2} = 5.4 min).

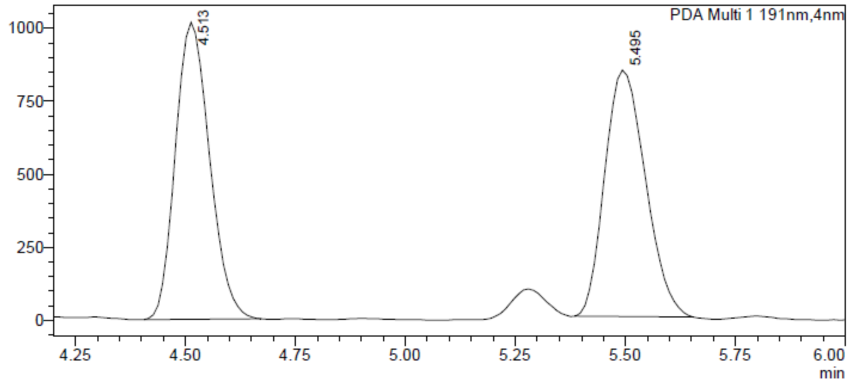

<Peak Table>

PDA Ch1 191nm

Peak#	Ret. Time	Area%
1	4.513	50.309
2	5.495	49.691
Total		100.000

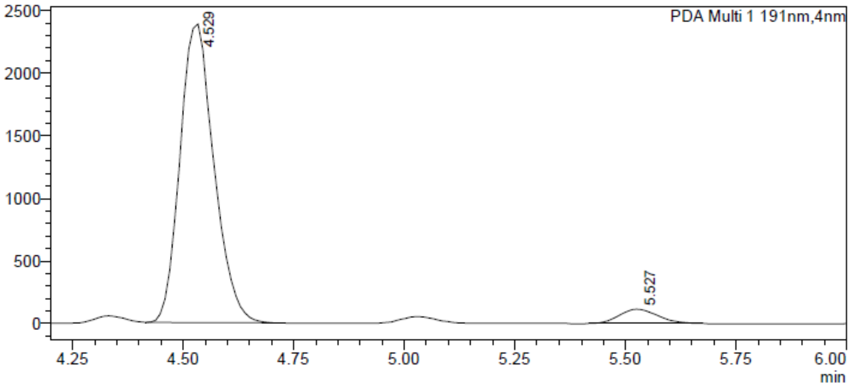

<Peak Table>

PDA Ch1 191nm

Peak#	Ret. Time	Area%
1	4.529	94.880
2	5.527	5.120
Total		100.000

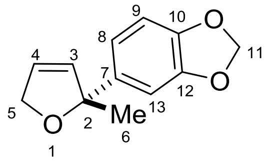

(*R*)-2-methyl-2-(3,4-methylenedioxyphenyl)-2,5-dihydrofuran (3ai)

Prepared according to **GP2** using 5-methyl-2,3-dihydrofuran (**1a**). Isolated by column chromatography (Cyclohexane:AcOEt 60:1) as a pale yellow oil (54% yield, 88% *ee*) with R_F = 0.47 (Cyclohexane:AcOEt 10:1); **^{1}H-NMR (C_6D_6, 400 MHz)**: δ (ppm) = 1.55 (s, 3H, H-6), 4.44 (ddd, $^2J_{H\text{-}H}$ = 13.0 Hz, $^3J_{H\text{-}H}$ = 6.1 Hz, $^4J_{H\text{-}H}$ = 1.6 Hz, 2H, H-5), 4.53 (ddd, $^2J_{H\text{-}H}$ = 13.0 Hz, $^3J_{H\text{-}H}$ = 6.1 Hz, $^4J_{H\text{-}H}$ = 1.6 Hz, 1H, H-5´), 5.32 (s, 2H, H-11), 5.33 (m, 1H, H-3), 5.62 (dt, $^3J_{H\text{-}H}$ = 6.0 Hz, $^3J_{H\text{-}H}$ = 2.4 Hz, 1H, H-4), 6.68 (d, $^3J_{H\text{-}H}$ = 8.0 Hz, 1H, H-9), 6.80 (dd, $^3J_{H\text{-}H}$ = 8.0 Hz, $^4J_{H\text{-}H}$ = 1.7 Hz 1H, H-8), 7.06 (d, $^4J_{H\text{-}H}$ = 1.7 Hz, 1H, H-13); **^{13}C{^{1}H}-NMR (C_6D_6, 100 MHz):** δ (ppm) = 28.7 (C-6), 74.6 (C-5), 90.6 (C-2), 100.8 (C-11), 106.4 (C-13), 108.1 (C-9), 118.1 (C-8), 125.0 (C-3) 134.6 (C-4), 141.6 (C-7), 146.8 (C-10), 148.2 (C-12); **HRMS (ESI positive)** calculated for $C_{12}H_{13}O_3$, 205.0859 $[M+H]^+$, found 205.0858; **IR spectrum (neat)** (cm^{-1}) = 1504, 1484, 1432, 1346, 1240, 1078, 1036, 1017, 938, 809, 730, 710; $[\alpha]^{23}_D$ = + 86.3 (*c* 0.90, CH_2Cl_2); **HPLC:** OJ-H, 206 nm, Hexane:*i*PrOH, 98:2, 1 mL/min, 30 °C, t_{R1} = 18.7 and t_{R2} = 20.6 min.

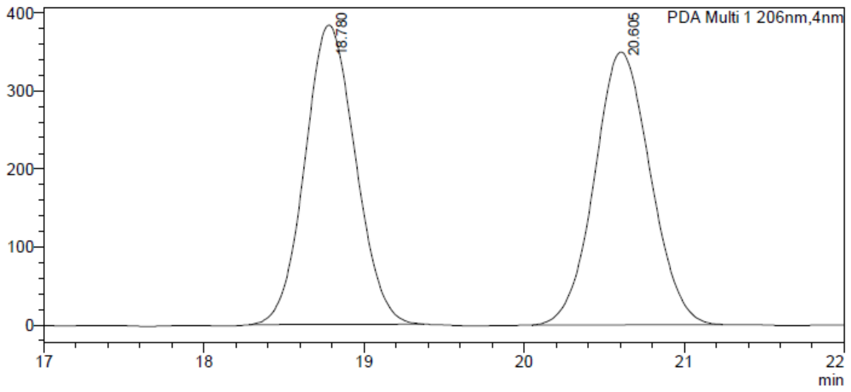

<Peak Table>

PDA Ch1 206nm

Peak#	Ret. Time	Area%
1	18.780	49.866
2	20.605	50.134
Total		100.000

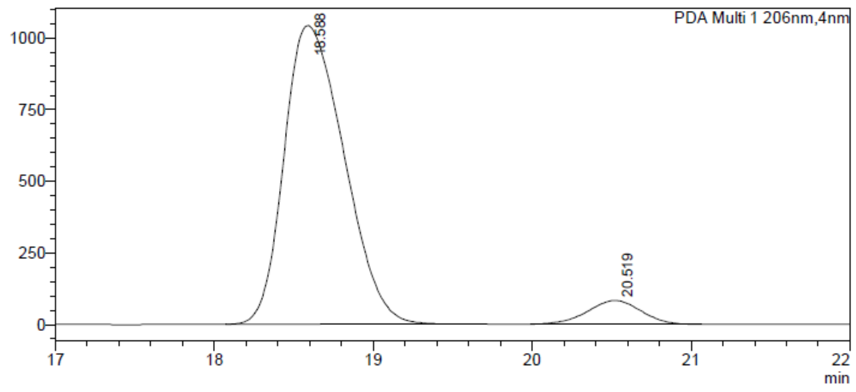

<Peak Table>

PDA Ch1 206nm

Peak#	Ret. Time	Area%
1	18.588	93.364
2	20.519	6.636
Total		100.000

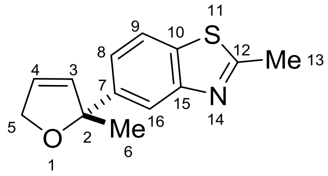

(*R*)-2-methyl-2-(2-methyl-5-benzo[*d*]thiazolyl)- 2,5-dihydrofuran (3aj)

Prepared according to **GP2** using 5-methyl-2,3-dihydrofuran (**1a**). Isolated by column chromatography (Cyclohexane:AcOEt 10:1) as a colorless oil (50% yield, >95% purity, 88% *ee*) with R_F = 0.2 (Cyclohexane:AcOEt 4:1); **^{1}H-NMR (C_6D_6, 400 MHz)**: δ (ppm) = 1.60 (s, 3H, H-6), 2.35 (s, 3H, H-13), 4.47 (ddd, $^2J_{H-H}$ = 13.1 Hz, $^3J_{H-H}$ = 2.5 Hz, $^3J_{H-H}$ = 1.6 Hz, 1H, H-5), 4.55 (ddd, $^2J_{H-H}$ = 13.1 Hz, $^3J_{H-H}$ = 2.5 Hz, $^3J_{H-H}$ = 1.6 Hz, 1H, H-5'), 5.34 (dt, $^3J_{H-H}$ = 6.0 Hz, $^4J_{H-H}$ = 1.7 Hz, 1H, H-3), 5.69 (dt, $^3J_{H-H}$ = 6.0 Hz, $^3J_{H-H}$ = 2.4 Hz, 1H, H-4), 7.36 (dd, $^3J_{H-H}$ = 8.4 Hz, $^4J_{H-H}$ = 1.8 Hz, 1H, H-8), 7.46 (d, $^3J_{H-H}$ = 8.4 Hz, 1H, H-9), 8.29 (d, $^4J_{H-H}$ = 1.8 Hz, 1H, H-16); **^{13}C{^{1}H}-NMR (C_6D_6, 100 MHz):** δ (ppm) = 19.8 (C-13), 29.0 (C-6), 74.8 (C-5), 90.8 (C-2), 119.4 (C-16), 121.2 (C-9), 122.4 (C-8), 125.3 (C-3), 134.5 (C-4), 134.6 (C-10), 145.8 (C-7), 154.8 (C-15), 166.5 (C-12); **HRMS (EI positive)** calculated for $C_3H_{14}NOS$, 232.0791 $[M+H]^+$, found 232.0794; **IR spectrum (neat)** (cm^{-1}) = 2973, 2925, 1621, 1525, 1417, 1154, 1053, 879, 813, 703, 643; $[\alpha]^{23}_D$ = +88.0 (*c* 1.09, CH_2Cl_2); **HPLC:** OJ-H, 206 nm, Hexane:*i*-PrOH, 98:2, 1 mL/min, 30 °C, t_{R1} = 22.1 and t_{R2} = 27.3 min.

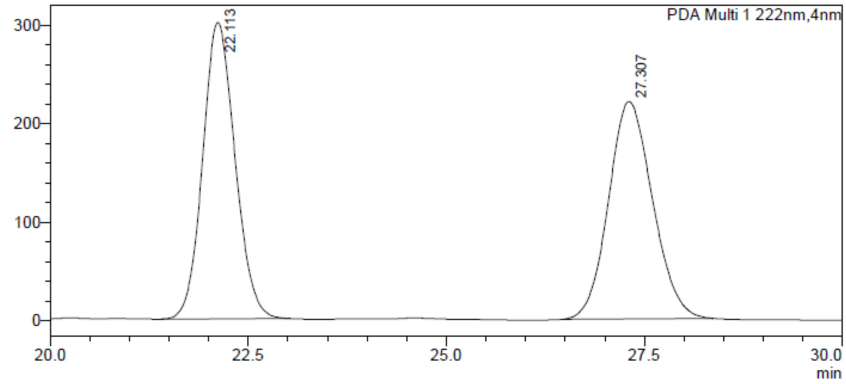

<Peak Table>

PDA Ch1 222nm

Peak#	Ret. Time	Area%
1	22.113	50.060
2	27.307	49.940
Total		100.000

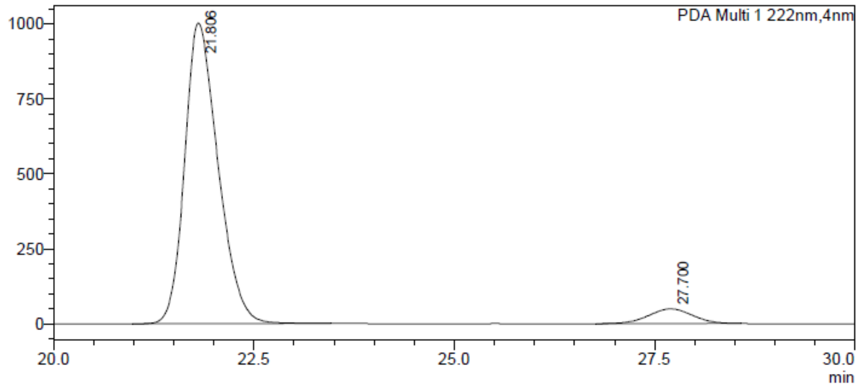

<Peak Table>

PDA Ch1 222nm

Peak#	Ret. Time	Area%
1	21.806	93.934
2	27.700	6.066
Total		100.000

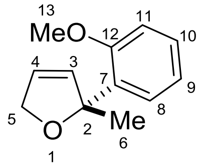

(*R*)-2-(2-methoxyphenyl)-2-methyl-2,5-dihydrofuran (3ak)

Prepared according to **GP2** using 5-methyl-2,3-dihydrofuran (**1a**). Isolated by column chromatography (Cyclohexane:AcOEt 80:1) as a colorless oil (53% yield, 90% *ee*) with R_F = 0.63 (Cyclohexane:AcOEt 10:1); **^{1}H-NMR (C_6D_6, 400 MHz)**: δ (ppm) = 2.87 (s, 3H, H-6), 3.24 (s, 3H, H-13), 4.52 (ddd, $^2J_{H-H}$ = 12.8 Hz, $^3J_{H-H}$ = 2.6 Hz, $^4J_{H-H}$ = 1.6 Hz, 1H, H-5), 4.63 (ddd, $^2J_{H-H}$ = 12.8 Hz, $^3J_{H-H}$ = 2.6 Hz, $^4J_{H-H}$ = 1.6 Hz, 1H, H-5'), 5.41 (dt, $^3J_{H-H}$ = 6.1 Hz, $^4J_{H-H}$ = 1.6 Hz, 1H, H-3), 6.52 (m, 2H, H-4 and H-11), 6.98 (td, $^3J_{H-H}$ = 7.5 Hz, $^4J_{H-H}$ = 1.1 Hz, 1H, H-9), 7.09 (td, $^3J_{H-H}$ = 7.3 Hz, $^4J_{H-H}$ = 1.8 Hz, 1H, H-10), 8.02 (dd, $^3J_{H-H}$ = 7.6 Hz, $^4J_{H-H}$ = 1.8 Hz, 1H, H-8); **^{13}C{^{1}H}-NMR (C_6D_6, 100 MHz):** δ (ppm) = 27.8 (C-6), 54.6 (C-13), 73.8 (C-5), 90.4 (C-2), 111.2 (C-11), 121.2 (C-9), 124.6 (C-3), 126.2 (C-8), 128.1 (C-10), 134.1 (C-4), 135.4 (C-7), 155.4 (C-12); **IR spectrum (neat)** (cm^{-1}) = 2928, 2867, 1727, 1598, 1583, 1484, 1435, 1362, 1321, 1279, 1236, 1179, 1143, 1111, 1079, 1063, 1019, 865, 811, 786, 752, 706, 651; $[\alpha]^{23}_D$ = +148.4 (*c* 0.83, CH_2Cl_2); **HPLC:** OJ-H, 206 nm, Hexane:*i*-PrOH, 99:1, 0.5 mL/min, 30 °C, t_{R1} = 11.5 and t_{R2} = 12.3 min.

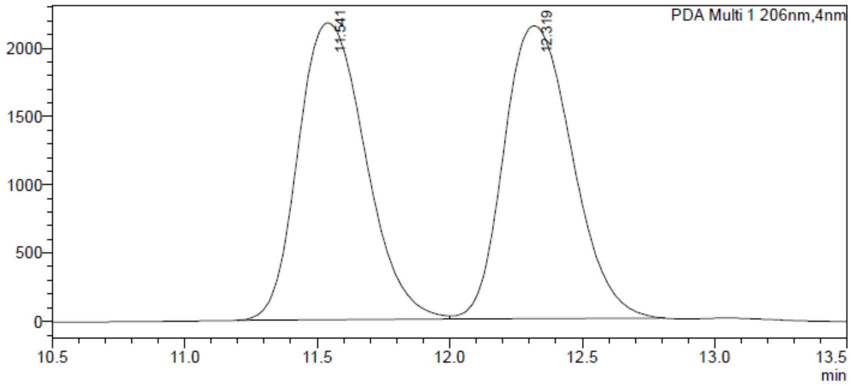

<Peak Table>

PDA Ch1 206nm

Peak#	Ret. Time	Area%
1	11.541	49.922
2	12.319	50.078
Total		100.000

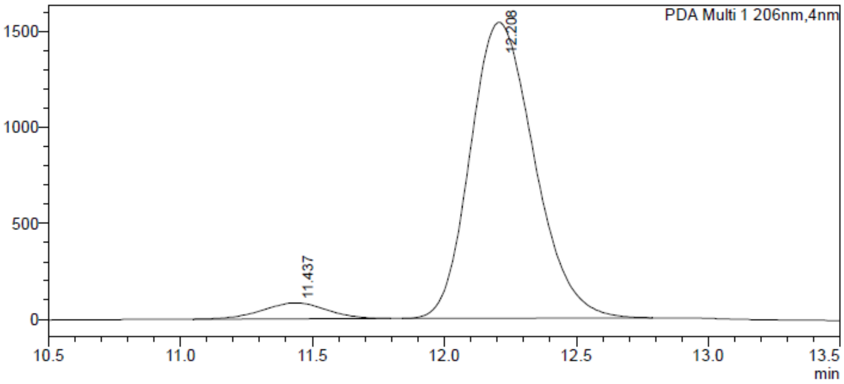

<Peak Table>

PDA Ch1 206nm

Peak#	Ret. Time	Area%
1	11.437	5.107
2	12.208	94.893
Total		100.000

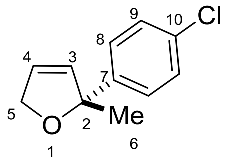

(*R*)-2-(2-chlorophenyl)-2-methyl-2,5-dihydrofuran (3al)

Prepared according to **GP2** using 5-methyl-2,3-dihydrofuran (**1a**). Isolated by column chromatography (Cyclohexane:AcOEt 60:1) as a colorless oil (25% yield, 92% *ee*) with R_F = 0.6 (Cyclohexane:AcOEt 10:1); **^{1}H-NMR (C_6D_6, 400 MHz)**: δ (ppm) = 1.46 (s, 3H, H-6), 4.38 (ddd, $^2J_{H-H}$ = 13.0 Hz, $^3J_{H-H}$ = 2.4 Hz, $^4J_{H-H}$ = 1.6 Hz, 1H, H-5), 4.50 (ddd, $^2J_{H-H}$ = 13.0 Hz, $^3J_{H-H}$ = 2.4 Hz, $^4J_{H-H}$ = 1.6 Hz, 1H, H-5´), 5.31 (dt, $^3J_{H-H}$ = 6.0 Hz, $^4J_{H-H}$ = 1.6 Hz, 1H, H-3), 5.55 (dt, $^3J_{H-H}$ = 6.0 Hz, $^3J_{H-H}$ = 2.4 Hz,1H, H-4), 7.11 (m, 4H, H-8 and H-9); **^{13}C{^{1}H}-NMR (C_6D_6, 100 MHz):** δ (ppm) = 28.6 (C-6), 74.7 (C-5), 90.3 (C-2), 125.3 (C-3), 126.6 (C-8 or C-9), 128. 6 (C-8 or C-9), 132.7 (C-10), 134.0 (C-4), 145.8 (C-7); **IR spectrum (neat)** (cm^{-1}) = 2923, 1720, 1489, 1083, 1012, 826, 730, 696, 578; $[\alpha]^{23}_D$ = +110.1.0 (*c* 0.4, CH_2Cl_2); **HPLC:** OJ-H, 193 nm, Hexane:*i*-PrOH, 98:2, 1 mL/min, 30 °C, t_{R1} = 7.0 and t_{R2} = 7.4 min.

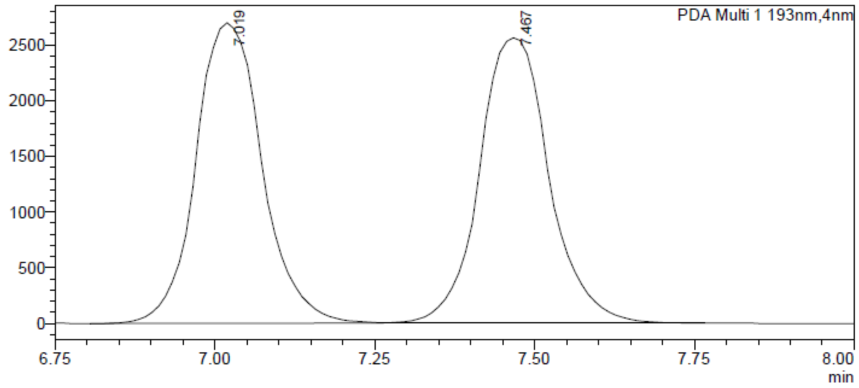

<Peak Table>

PDA Ch1 193nm

Peak#	Ret. Time	Area%
1	7.019	50.064
2	7.467	49.936
Total		100.000

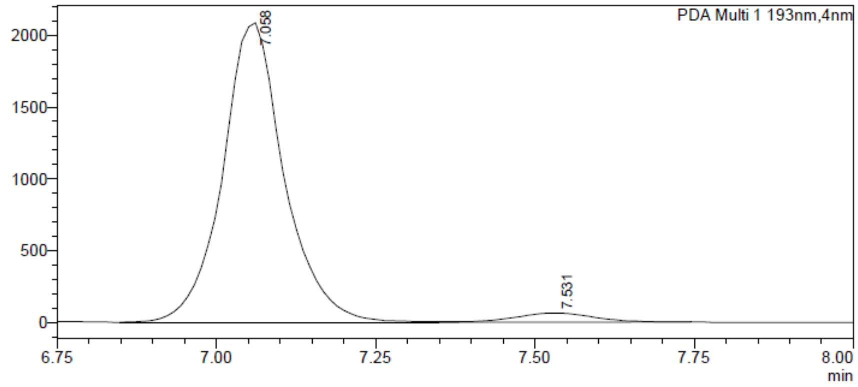

<Peak Table>

PDA Ch1 193nm

Peak#	Ret. Time	Area%
1	7.058	96.234
2	7.531	3.766
Total		100.000

4.6 Characterization data of 2-aryl-2-methyl-2,3-dihydrofurans

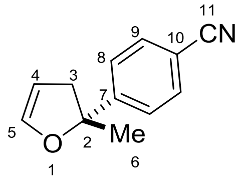

(*R*)-2-(4-cyanophenyl)-2-methyl-2,3-dihydrofuran (4ac)

Prepared according to **GP2** using 5-methyl-2,3-dihydrofuran (**1a**). Isolated by column chromatography (Cyclohexane:AcOEt 60:1) as a pale yellow oil (59% yield, 97% *ee*) with R_F = 0.42 (Cyclohexane:AcOEt 10:1); **^{1}H-NMR (C_6D_6, 400 MHz)**: δ (ppm) = 1.29 (s, 3H, H-6), 2.32 (m, 2H, H-3), 4.51 (m, 1H, H-4), 6.10 (m, 1H, H-5), 6.95 (m, 2H, H-8), 7.04 (m, 2H, H-9); **^{13}C{^{1}H}-NMR (C_6D_6, 100 MHz):** δ (ppm) = 28.8 (C-6), 44.1 (C-3), 86.7 (C-2), 98.4 (C-4), 111,3 (C-10), 118.8 (C-11), 125.3 (C-8), 132.1 (C-9), 144.4 (C-5), 152.70 (C-7); **HRMS (ESI positive)** calculated for $C_{12}H_{11}NO$, 185.08325[M]$^+$, found 185.08352; **IR spectrum (neat)** (cm^{-1}) = 2927, 2228, 1624, 1607, 1503, 1294, 1160, 1053, 978, 835, 712, 581; $[\alpha]^{23}_D$ = + 24.2 (*c* 1.05, CH_2Cl_2); **HPLC:** OJ-H, 223 nm, Hexane:*i*PrOH, 98:2, 1 mL/min, 30 °C, t_{R1} = 10.1 and t_{R2} = 10.8 min.

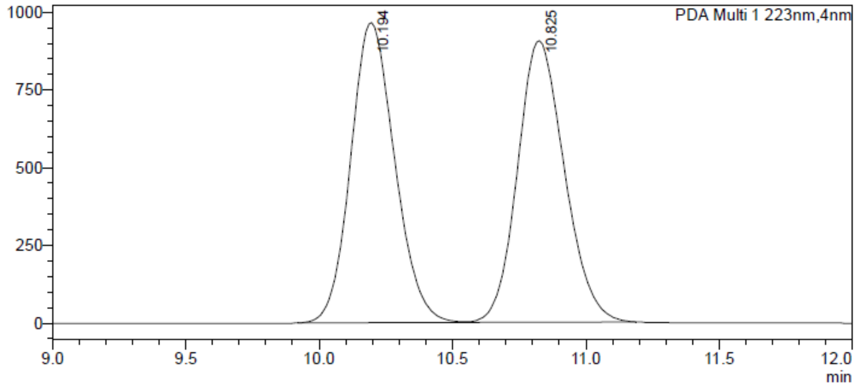

<Peak Table>

PDA Ch1 223nm

Peak#	Ret. Time	Area%
1	10.194	50.012
2	10.825	49.988
Total		100.000

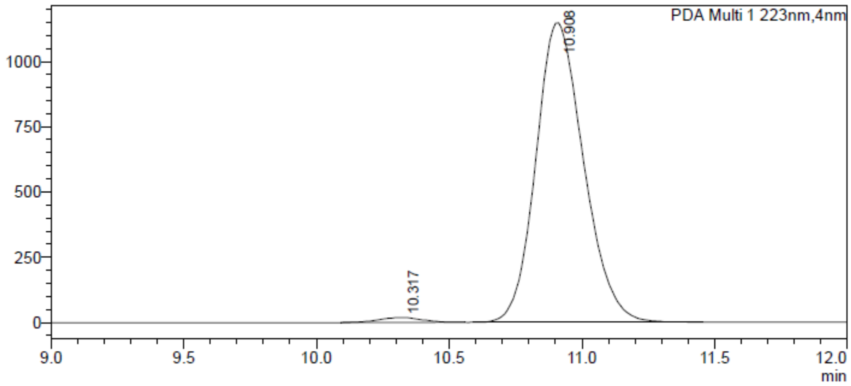

<Peak Table>

PDA Ch1 223nm

Peak#	Ret. Time	Area%
1	10.317	1.416
2	10.908	98.584
Total		100.000

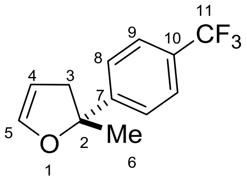

(*R*)-2-methyl-2-(4-(trifluoromethyl)phenyl)-2,3-dihydrofuran (4am)

Prepared according to **GP2** using 5-methyl-2,3-dihydrofuran (**1a**). Isolated by column chromatography (Cyclohexane:AcOEt 10:1) as a colorless oil (51% yield, 98% *ee*) with R_F = 0.44 (Cyclohexane:AcOEt 10:1); **^{1}H-NMR (C_6D_6, 400 MHz)**: δ (ppm) = 1.38 (s, 3H, H-6), 2.39 (dt, $^2J_{H-H}$ = 15.1 Hz, $^3J_{H-H}$ = 2.5 Hz, 1H, H-3), 2.45 (dt, $^2J_{H-H}$ = 15.1 Hz, $^3J_{H-H}$ = 2.5 Hz, 1H, H-3'), 4.55-4.57 (m, 1H, H-4), 6.15-6.17 (m, 1H, H-5), 7.15-7.17 (m, 2H, H-8), 7.35-7.37 (m, 2H, H-9); **^{13}C{^{1}H}-NMR (C_6D_6, 100 MHz):** δ (ppm) = 29.2 (C-6), 44.3 (C-3), 86.9 (C-2), 98.5 (C-4), 125.07 (q, $^1J_{C-F}$ = 271.8 Hz, C-11), 125.3 (C-8), 125.5 (q, $^3J_{C-F}$ = 3.8 Hz, C-9), 129.2 (q, $^2J_{C-F}$ = 32.2 Hz, C-10), 144.5 (C-5), 152.2 (C-7); **GC-MS (EI):** ($C_{12}H_{11}F_3O$), 228.1 (45, M^+), 213.1 (60), 199.1 (83), 159.1 (100), 115.1 (49), 103.0 (22); **IR spectrum (neat)** (cm^{-1}) = 2978, 2935, 2861, 1620, 1450, 1410, 1324, 1161, 1117, 1054, 1014, 978, 919, 841, 704, 607; $[\alpha]^{23}_D$ = +44.9 (*c* 0.81, CH_2Cl_2); **GC:** Lipodex E, 60-20-1-100-20-170, 45 cm/s, H_2, t_{R1} = 30.7 and t_{R2} = 32.7 min.

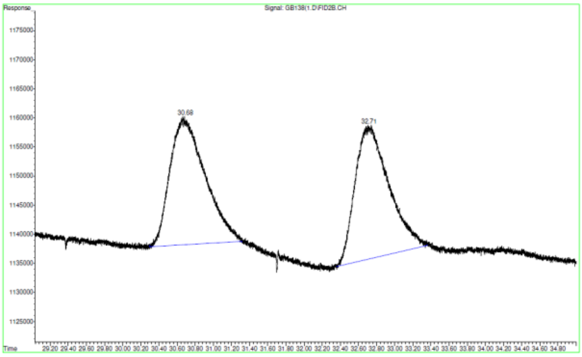


```
1 30.680  30.296  31.315    M  22032   5865242  99.10% 49.773%
2 32.707  32.360  33.359    M  23065   5918727 100.00% 50.227%
```

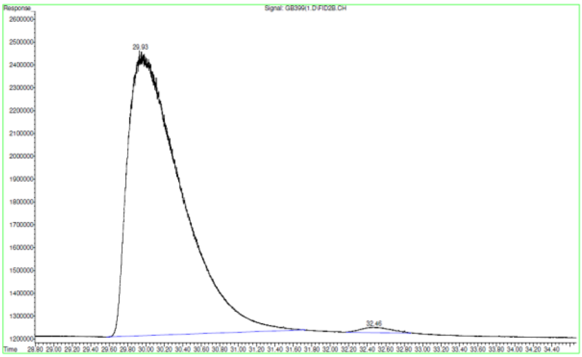


```
1 29.930  29.597  31.813    M1250770 502126435 100.00% 99.015%
2 32.463  32.168  32.866    M  23974   4994602   0.99%  0.985%
```

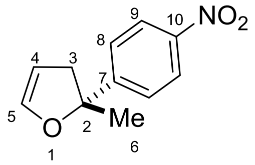

(*R*)-2-methyl-2-(4-nitrophenyl)-2,3-dihydrofuran (4an)

Prepared according to **GP2** using 5-methyl-2,3-dihydrofuran (**1a**). Isolated by column chromatography (Cyclohexane:AcOEt 75:1) as a pale yellow oil (54% yield, 98% *ee*) with R_F = 0.54 (Cyclohexane:AcOEt 10:1); **^{1}H-NMR (C_6D_6, 400 MHz)**: δ (ppm) = 1.31 (s, 3H, H-6), 2.34 (m, 2H, H-3 and H-3′), 4.52 (m, 1H, H-4), 6.10 (m, 1H, H-5), 6.97 (m, 2H, H-8), 7.83 (m, 2H, H-9); **^{13}C{^{1}H}-NMR (C_6D_6, 100 MHz):** δ (ppm) = 28.9 (C-6), 44.1 (C-3), 86.7 (C-2), 98.5 (C-4), 123.6 (C-9), 125.4 (C-8), 144.4 (C-5), 147.2 (C-10), 154.6 (C-7); **HRMS (ESI positive)** calculated for $C_{11}H_{11}NO_3$, 205.2130 $[M]^+$, found 205.3; **IR spectrum (neat)** (cm^{-1}) = 1603, 1518, 1160, 1052, 853, 699; $[\alpha]^{23}_D$ = +16.1 (*c* 0.81, CH_2Cl_2); **HPLC:** OD-H, 264 nm, Hexane:*i*PrOH, 99.5:0.5, 1 mL/min, 30 °C, t_{R1} = 9.5 and t_{R2} = 10.3 min.

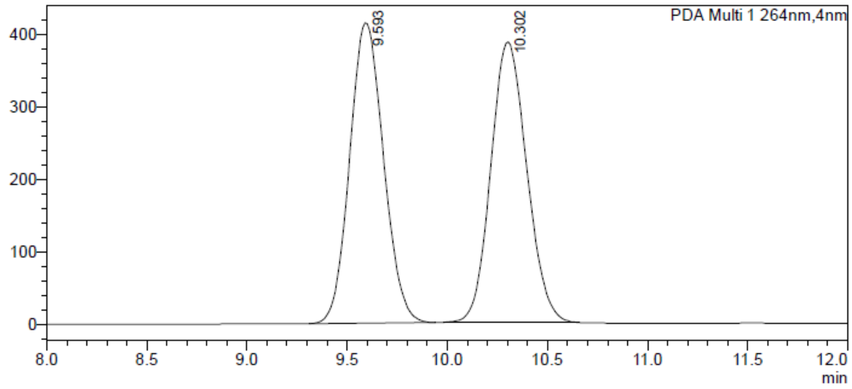

<Peak Table>

PDA Ch1 264nm

Peak#	Ret. Time	Area%
1	9.593	50.166
2	10.302	49.834
Total		100.000

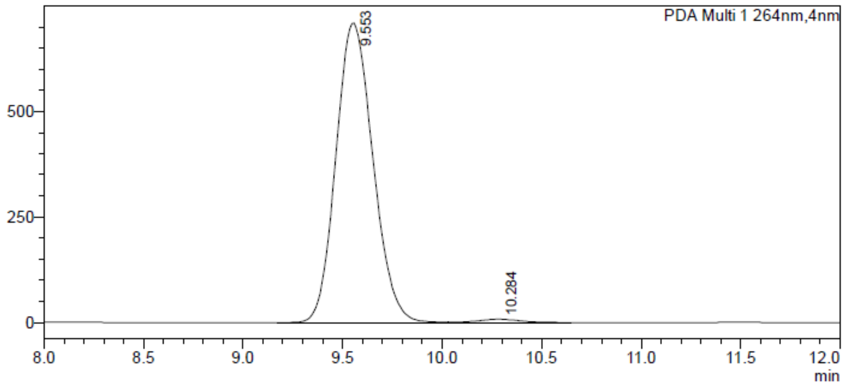

<Peak Table>

PDA Ch1 264nm

Peak#	Ret. Time	Area%
1	9.553	98.871
2	10.284	1.129
Total		100.000

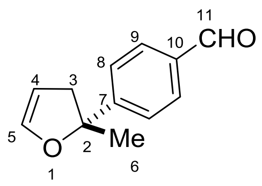

(*R*)-2-(4-formylphenyl)-2-methyl-2,3-dihydrofuran (4ao)

Prepared according to **GP2** using 5-methyl-2,3-dihydrofuran (**1a**). Isolated by column chromatography (Cyclohexane:AcOEt 60:1) as a pale yellow oil (35% yield, 97% *ee*) with R_F = 0.48 (Cyclohexane:AcOEt 10:1); **^{1}H-NMR (C_6D_6, 400 MHz)**: δ (ppm) = 1.40 (s, 3H, H-6), 2.41 (dt, $^2J_{H-H}$ = 14.8 Hz, $^3J_{H-H}$ = 2.4 Hz, 1H, H-3), 2.46 (dt, $^2J_{H-H}$ = 14.8 Hz, $^3J_{H-H}$ = 2.4 Hz, 1H, H-3´), 4.55 (m, 1H, H-4), 6.17 (m, 1H, H-5), 7.24 (m, 2H, H-8), 7.56 (m, 2H, H-9), 9.68 (s, 1H, H-11); **^{13}C{^{1}H}-NMR (C_6D_6, 100 MHz):** δ (ppm) = 29.1 (C-6), 44.2 (C-3), 87.1 (C-2), 98.5 (C-4), 125.3 (C-8), 129.8 (C-9), 135.8 (C-10), 144.5 (C-5), 154.3 (C-10), 190.9 (C-11); **MS (ESI)**: $C_{12}H_{12}O_2$, 189.3 [M+H]$^+$; **IR spectrum (neat)** (cm^{-1}) = 1698, 1607, 1211, 1161, 1053, 827, 705, 568; $[\alpha]^{23}_D$ = +15.4 (*c* 0.55, CH_2Cl_2); **HPLC:** OJ-H, 246 nm, Hexane:*i*PrOH, 98:2, 1 mL/min, 30 °C, t_{R1} = 12.6 and t_{R2} = 14.0 min.

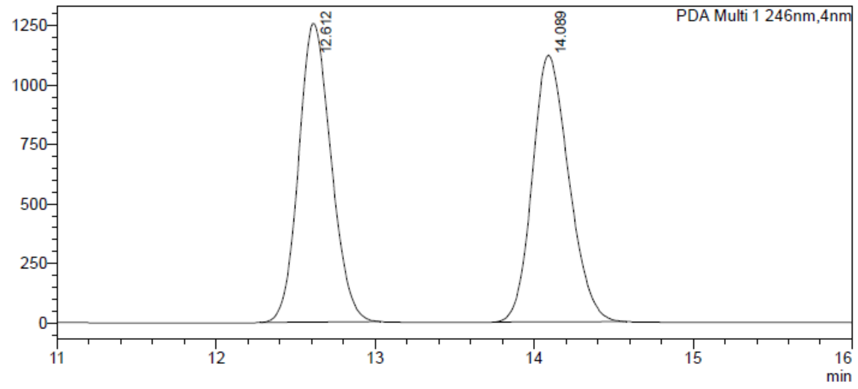

<Peak Table>

PDA Ch1 246nm

Peak#	Ret. Time	Area%
1	12.612	49.941
2	14.089	50.059
Total		100.000

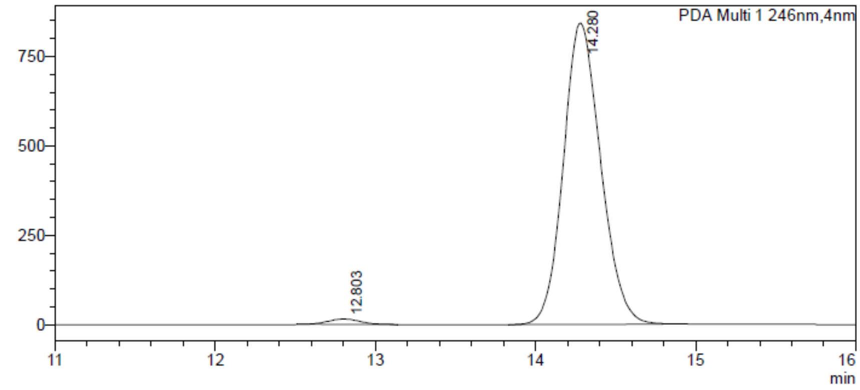

<Peak Table>

PDA Ch1 246nm

Peak#	Ret. Time	Area%
1	12.803	1.529
2	14.280	98.471
Total		100.000

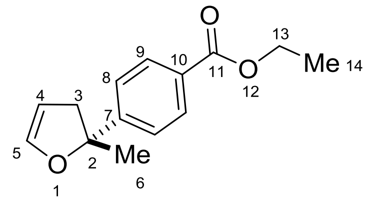

(*R*)-2-(4-ethoxycarbonylphenyl)-2-methyl-2,3-dihydrofuran (4ap)

Prepared according to **GP2** using 5-methyl-2,3-dihydrofuran (**1a**). Isolated by column chromatography (Cyclohexane:AcOEt 85:1) as a pale yellow oil (40% yield, 92% *ee*) with R_F = 0.53 (Cyclohexane:AcOEt 10:1); **^{1}H-NMR (C_6D_6, 400 MHz)**: δ(ppm) = 1.03 (t, $^3J_{H-H}$ = 7 Hz, 3H, H-14), 1.44 (s, 3H, H-6), 2.42 (dt, $^2J_{H-H}$ = 15.0 Hz, $^3J_{H-H}$ = 2.4 Hz, 2H, H-3), 2.53 (dt, $^2J_{H-H}$ = 15.0 Hz, $^3J_{H-H}$ = 2.4 Hz, 2H , H-3´), 4.14 (c, $^3J_{H-H}$ = 7 Hz, 2H, H-13), 4.56 (m, 1H, H-4), 6.18 (m, 1H, H-5), 7.32 (m, 2H, H-8), 8.19 (m, 2H, H-9); **^{13}C{^{1}H}-NMR(C_6D_6, 100 MHz):** δ(ppm) = 13.1 (C-13), 27.9 (C-6), 43.1 (C-3), 59.5 (C-12), 86.0 (C-2), 97.2 (C-4), 123.7 (C-8), 128.6 (C-10), 128.8 (C-9), 143.3 (C-5), 151.9 (C-7), 164.9 (C-11); **HRMS (ESI positive)** calculated for **$C_{14}H_{16}O_3$**, 233.1172 $[M+H]^+$, found 233.1167; **IR spectrum (neat)** (cm^{-1}) = 1714, 1613, 1270, 1162, 1104, 1054, 1019, 858, 773, 705; $[\alpha]^{23}_D$ = +10.0 (*c* 0.87, CH_2Cl_2); **HPLC:** OJ-H, 226 nm, Hexane:*i*PrOH, 98:2, 1 mL/min, 30 °C, t_{R1} = 11.3 and t_{R2} = 12.3 min.

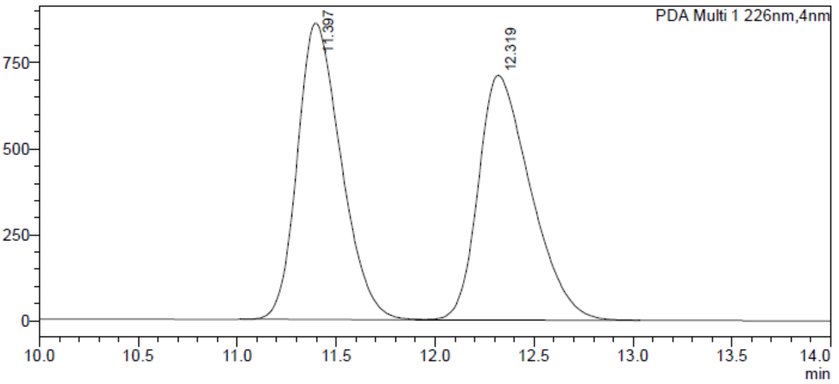

<Peak Table>

PDA Ch1 226nm

Peak#	Ret. Time	Area%
1	11.397	50.286
2	12.319	49.714
Total		100.000

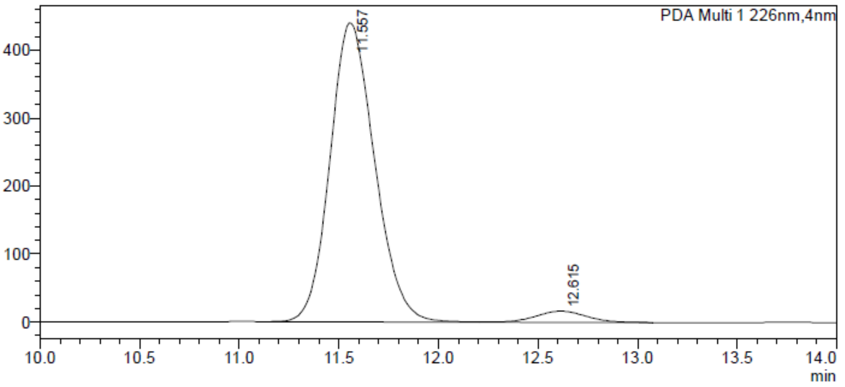

<Peak Table>

PDA Ch1 226nm

Peak#	Ret. Time	Area%
1	11.557	96.005
2	12.615	3.995
Total		100.000

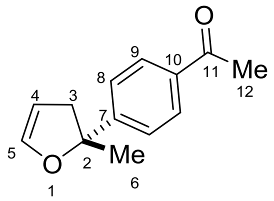

(*R*)-2-methyl-2-(4-methylcarbonylphenyl)-2,3-dihydrofuran (4aq)

Prepared according to **GP2** using 5-methyl-2,3-dihydrofuran (**1a**). Isolated by column chromatography (Cyclohexane:AcOEt 60:1) as a pale yellow oil (45% yield with 95% purity, 72% *ee*) with R_F = 0.35 (Cyclohexane:AcOEt 10:1); **^{1}H-NMR (C_6D_6, 400 MHz)**: δ (ppm) = 1.46 (s, 3H, H-6), 2.12 (s, 3H, H-12), 2.44 (dt, $^2J_{H\text{-}H}$ = 15.2 Hz, $^3J_{H\text{-}H}$ = 2.4 Hz, 2H, H-3), 2.56 (dt, $^2J_{H\text{-}H}$ = 15.2 Hz, $^3J_{H\text{-}H}$ = 2.4 Hz, 2H, H-3´), 4.58 (m, 1H, H-4), 6.21 (m, 1H, H-5), 7.28 (m, 2H, H-8), 7.80 (m, 2H, H-9); **^{13}C{^{1}H}-NMR (C_6D_6, 100 MHz):** δ (ppm) = 26.1 (C-12), 29.1 (C-6), 44.3 (C-3), 87.2 (C-2), 98.5 (C-4), 124.9 (C-8), 128.7 (C-9), 136.4 (C-10), 144.6 (C-5), 153.0 (C-7), 196.1 (C-11); **HRMS (ESI positive)** calculated for $C_{13}H_{15}O_2$, 203.1067 $[M+H]^+$, found 203.1070; **IR spectrum (neat)** (cm^{-1}) = 1681, 1622, 1607, 1358, 1267, 1161, 1054, 1015, 958, 837, 706, 598; $[\alpha]^{23}_D$ = + 6.3 (*c* 0.87, CH_2Cl_2); **HPLC:** OJ-H, 206 nm, Hexane:*i*PrOH, 98:2, 1 mL/min, 30 °C, t_{R1} = 18.5 and t_{R2} = 20.0 min.

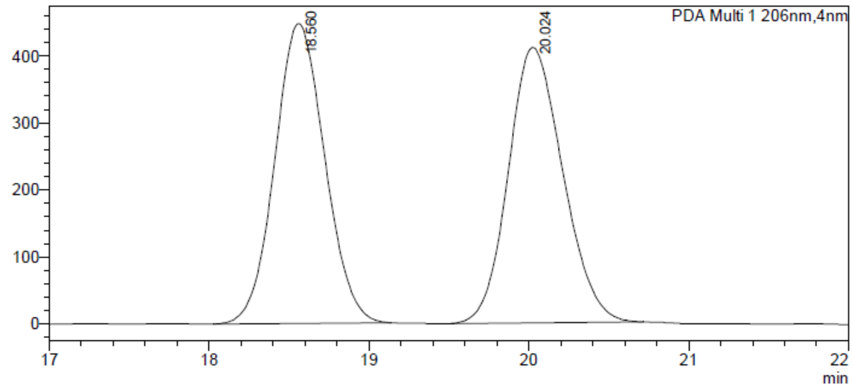

<Peak Table>

PDA Ch1 206nm

Peak#	Ret. Time	Area%
1	18.560	49.899
2	20.024	50.101
Total		100.000

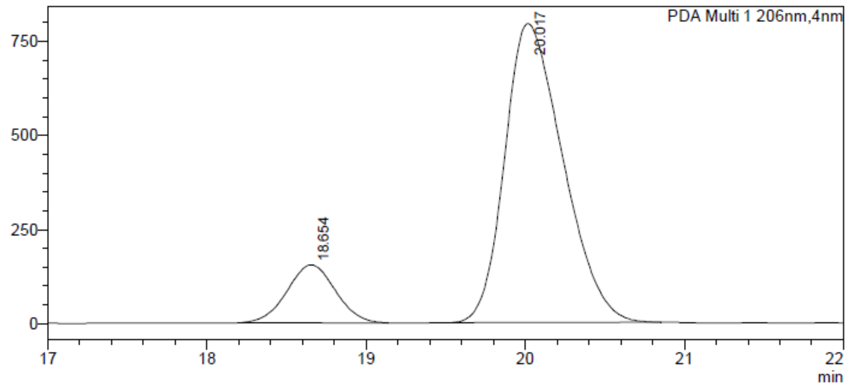

<Peak Table>

PDA Ch1 206nm

Peak#	Ret. Time	Area%
1	18.654	13.841
2	20.017	86.159
Total		100.000

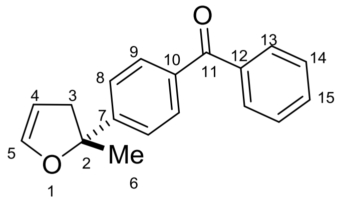

(*R*)-2-(benzophenone-4-yl)-2-methyl-2,3-dihydrofuran (4ar)

Prepared according to **GP2** using 5-methyl-2,3-dihydrofuran (**1a**). Isolated by column chromatography (Cyclohexane:AcOEt 85:1) as a pale yellow oil (39% yield, 93% *ee*) with R_F = 0.54 (Cyclohexane:AcOEt 10:1); **^{1}H-NMR (C_6D_6, 400 MHz)**: δ (ppm) = 1.47 (s, 3H, H-6), 2.44 (dt, $^2J_{H-H}$ = 15.0 Hz, $^3J_{H-H}$ = 2.3 Hz, 2H, H-3), 2.58 (dt, $^2J_{H-H}$ = 15.0 Hz, $^3J_{H-H}$ = 2.3 Hz, 2H, H-3′), 4.55 (m, 1H, H-4), 6.20 (m, 1H, H-5), 7.05 (m, 2H, H-14), 7.12 (m, 1H, H-15), 7.28 (m, 2H, H-8), 7.74 (m, 4H, H-9 and H-13); **^{13}C{^{1}H}-NMR (C_6D_6, 100 MHz):** δ (ppm) = 29.2 (C-6), 44.3 (C-3), 87.23 (C-2), 98.5 (C-4), 124.7 (C-8), 128.4 (C-14), 130.2 (C-13 or C-9), 130.4 (C-13 or C-9), 132.1 (C-15), 136.8 (C-10), 138.4 (C-12), 144.6 (C-5), 152.4 (C-7), 195.4 (C-11); **HRMS (ESI positive)** calculated for $C_{18}H_{17}O_2$, 265.1223 $[M+H]^+$, found265.1222; **IR spectrum (neat)** (cm^{-1}) = 1657, 1604, 1446, 1403, 1372, 1313, 1274, 1161, 1053, 922, 851, 698; $[\alpha]^{23}_D$ = -4.4 (*c* 0.75, CH_2Cl_2); **HPLC:** OJ-H, 206 nm, Hexane:*i*PrOH, 98:2, 1 mL/min, 30 °C, t_{R1} = 15.8 and t_{R2} = 17.1 min.

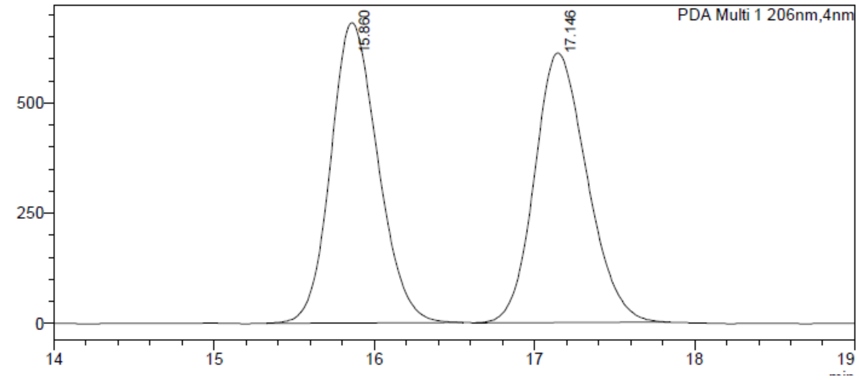

<Peak Table>

PDA Ch1 206nm

Peak#	Ret. Time	Area%
1	15.860	50.047
2	17.146	49.953
Total		100.000

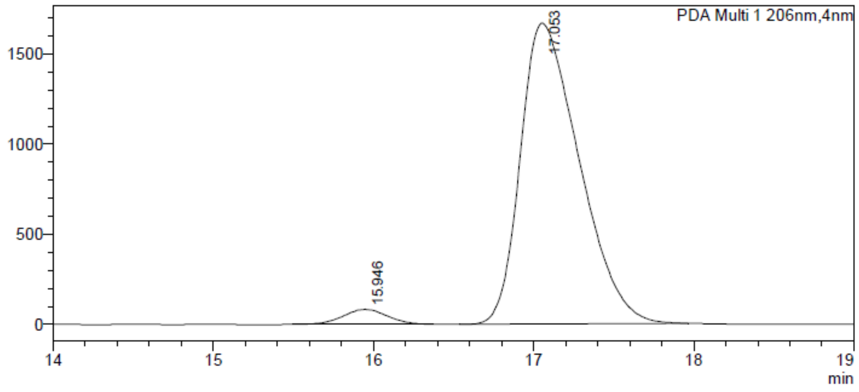

<Peak Table>

PDA Ch1 206nm

Peak#	Ret. Time	Area%
1	15.946	3.605
2	17.053	96.395
Total		100.000

4.7 Characterization data of 2-alkyl-2-aryl-2,5-dihydrofurans

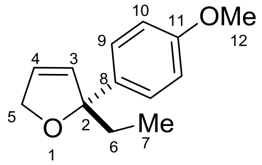

(*R*)-2-Ethyl-2-(4-methoxyphenyl)-2,5-dihydrofuran (3bb)

Prepared according to **GP3** using 5-ethyl-2,3-dihydrofuran (**1b**). Isolated by column chromatography (Cyclohexane:AcOEt 80:1) as a colorless oil (61% yield, 92% *ee*) with R_F = 0.17 (Cyclohexane:AcOEt 10:1); **^{1}H-NMR (C_6D_6, 400 MHz)**: δ(ppm) = 0.95 (t, $^3J_{H\text{-}H}$ = 7.4 Hz, 3H, H-7), 1.85 (dq, $^2J_{H\text{-}H}$ = 14.4 Hz, $^3J_{H\text{-}H}$ = 7.4 Hz, 1H, H-6), 1.96 (dq, $^2J_{H\text{-}H}$ = 14.8 Hz, $^3J_{H\text{-}H}$ = 7.4 Hz, 1H, H-6'), 3.33 (s, 3H, H-12), 4.52 (ddd, $^2J_{H\text{-}H}$ = 12.9 Hz, $^3J_{H\text{-}H}$ = 2.4 Hz, $^4J_{H\text{-}H}$ = 1.7 Hz, 1H, H-5), 4.57 (ddd, $^2J_{H\text{-}H}$ = 12.9 Hz, $^3J_{H\text{-}H}$ = 2.4 Hz, $^4J_{H\text{-}H}$ = 1.7 Hz, 1H, H-5´), 5.43 (dt, $^3J_{H\text{-}H}$ = 6.1 Hz, $^4J_{H\text{-}H}$ = 1.7 Hz, 1H, H-3), 5.69 (dt, $^3J_{H\text{-}H}$ = 6.1 Hz, $^3J_{H\text{-}H}$ = 2.4 Hz, 1H, H-4), 6.82-6.84 (m, 2H, H-10), 7.31-7.35 (m, 2H, H-9); **^{13}C{^{1}H}-NMR (C_6D_6, 100 MHz):** δ(ppm) = 8.7 (C-7), 34.9 (C-6), 54.8 (C-12), 75.2 (C-5), 93.8 (C-2), 113.9 (C-10), 125.7 (C-3), 126.5 (C-9), 133.0 (C-4), 138.8 (C-8), 159.0 (C-11); **GC-MS (EI):** ($C_{13}H_{16}O_2$), 204.0 (1, M^+), 175.1 (100, M^+ – 29), 160.0 (23, M^+ – 44); **IR spectrum (neat)** (cm^{-1}) = 2966, 2839, 2610, 1509, 1245, 1176, 1083, 1033, 829, 809, 699; $[\alpha]^{23}_D$ = +115.4 (*c* 1.00, CH_2Cl_2); **HPLC:** OJ-H, 226 nm, Hexane:*i*-PrOH, 98:2, 1 mL/min, 30 °C, t_{R1} = 22.6 and t_{R2} = 24.2 min.

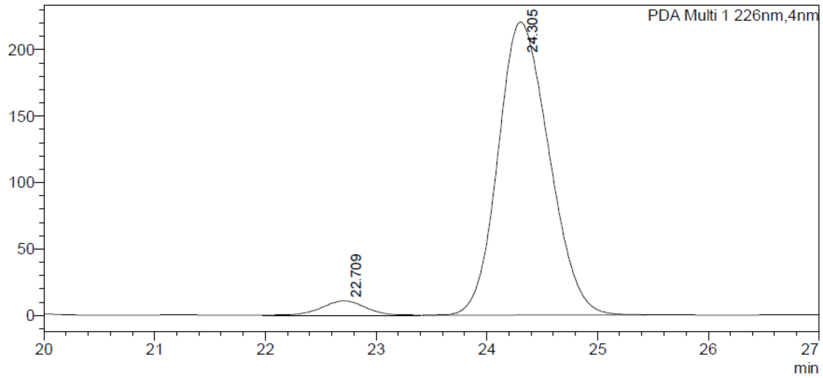

<Peak Table>
PDA Ch1 226nm

Peak#	Ret. Time	Area%
1	22.610	50.440
2	24.241	49.560
Total		100.000

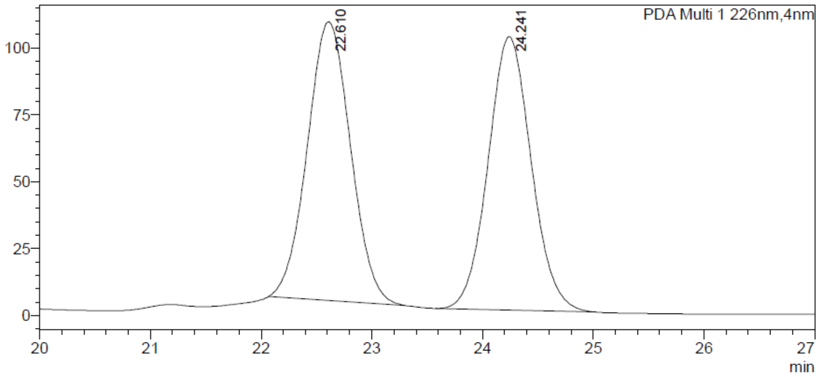

<Peak Table>
PDA Ch1 226nm

Peak#	Ret. Time	Area%
1	22.709	3.927
2	24.305	96.073
Total		100.000

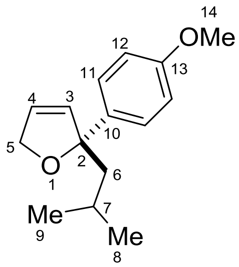

(*R*)-2-isobutyl-2-(4-methoxyphenyl)-2,5-dihydrofuran (3cb)

Prepared according to **GP3** using 5-isobutyl-2,3-dihydrofuran (**1c**). Isolated by column chromatography (Cyclohexane:AcOEt 70:1) as a pale yellow oil (44% yield, 91% *ee*) with R_F = 0.65 (Cyclohexane:AcOEt 10:1); **^{1}H-NMR (C_6D_6, 500 MHz):** δ (ppm) =0.93 (d, $^3J_{H\text{-}H}$ = 6.5 Hz, 3H, H-8 or H-9), 1.02 (d, $^3J_{H\text{-}H}$ = 6.4 Hz, 3H, H-8 or H-9), 1.75 (dd, $^2J_{H\text{-}H}$ = 13.7 Hz, $^3J_{H\text{-}H}$ = 6.3 Hz, 3H, H-6), 1.81 (m, 1H, H-7), 1.90 (dd, $^2J_{H\text{-}H}$ = 13.7 Hz, $^3J_{H\text{-}H}$ = 6.3 Hz, 3H, H-6´), 3.33 (s, 3H, H-14), 4.49 (ddd, $^2J_{H\text{-}H}$ = 12.9 Hz, $^3J_{H\text{-}H}$ = 2.3 Hz, $^4J_{H\text{-}H}$ = 1.7 Hz, 1H, H-5), 4.57 (ddd, $^2J_{H\text{-}H}$ = 12.9 Hz, $^3J_{H\text{-}H}$ = 2.3 Hz, $^4J_{H\text{-}H}$ = 1.7 Hz, 1H, H-5´), 5.38 (dt, $^3J_{H\text{-}H}$ = 6.1 Hz, $^4J_{H\text{-}H}$ = 1.6 Hz, 1H, H-3), 5.75 (dt, $^3J_{H\text{-}H}$ = 6.0 Hz, $^3J_{H\text{-}H}$ = 2.4 Hz, 1H, H-4), 6.84 (m, 2H, H-12), 7.32 (m, 2H, H-11); **^{13}C{^{1}H}-NMR(C_6D_6, 125 MHz):** δ (ppm) = 24.5 (C-8 or C-9), 24.7 (C-8 or C-9), 25.0 (C-7), 50.6 (C-6), 54.8 (C-14), 74.8 (C-5), 93.8 (C-2), 113.9 (C-12), 125.0 (C-3), 126.3 (C-11), 134.2 (C-4), 139.1 (C-10), 158.8 (C-13); **MS (ESI)**: $C_{15}H_{20}O_2$,233.4 $[M]^+$; **MS (EI positive)** calculated for $[M]^+$ 233.14, found 233.4; **IR spectrum(neat)** (cm^{-1}) = 2952, 2837, 1610, 1608, 1463, 1351, 1298, 1245, 1124, 1084, 10396, 530, 831, 807, 735, 701, 651; $[\alpha]^{23}_D$ = + 100.6 (*c* 0.79, CH_2Cl_2); **HPLC:** OJ-H, 205 nm, Hexane:*i*PrOH, 98:2, 1 mL/min, 30 °C, t_{R1} = 10.0 and t_{R2} = 11.0 min.

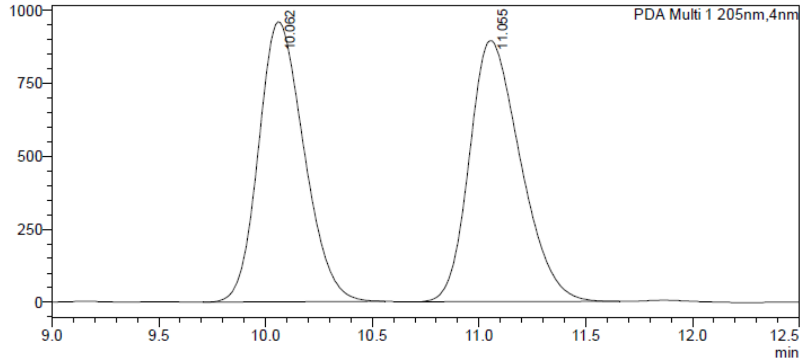

<Peak Table>

PDA Ch1 205nm

Peak#	Ret. Time	Area%
1	10.062	48.786
2	11.055	51.214
Total		100.000

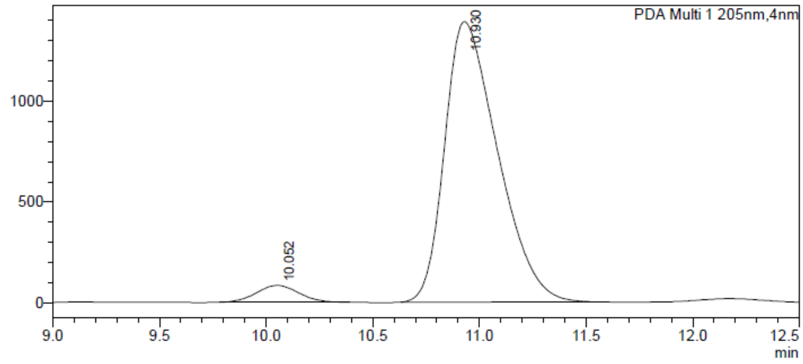

<Peak Table>

PDA Ch1 205nm

Peak#	Ret. Time	Area%
1	10.052	4.595
2	10.930	95.405
Total		100.000

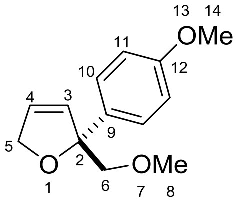

(*R*)-2-(Methoxymethyl)-2-(4-methoxyphenyl)-2,5-dihydrofuran (3db)

Prepared according to **GP2** using 5-(methoxymethyl)-2,3-dihydrofuran (**1d**). Isolated by column chromatography (Cyclohexane:AcOEt 10:1) as a colorless oil (64% yield, 91% *ee*) with R_F = 0.35 (Cyclohexane:AcOEt 2:1); **^{1}H-NMR (C_6D_6, 400 MHz)**: δ (ppm) = 3.15 (s, 3H, H-8), 3.32 (s, 3H, H-14), 3.58 (d, $^2J_{H\text{-}H}$ = 9.9 Hz, 1H, H-6), 3.61 (d, $^2J_{H\text{-}H}$ = 9.9 Hz, 1H, H-6'), 4.52 (ddd, $^2J_{H\text{-}H}$ = 12.8 Hz, $^3J_{H\text{-}H}$ = 2.5 Hz, $^3J_{H\text{-}H}$ = 1.6 Hz, 1H, H-5), 4.67 (ddd, $^2J_{H\text{-}H}$ = 12.8 Hz, $^3J_{H\text{-}H}$ = 2.4 Hz, $^3J_{H\text{-}H}$ = 1.6 Hz, 1H, H-5'), 5.53 (dt, $^3J_{H\text{-}H}$ = 6.1 Hz, $^4J_{H\text{-}H}$ = 1.6 Hz, 1H, H-3), 5.98 (dt, $^3J_{H\text{-}H}$ = 6.1 Hz, $^3J_{H\text{-}H}$ = 2.4 Hz, 1H, H-4), 6.82-6.86 (m, 2H, H-11), 7.42-7.46 (m, 2H, H-10); **^{13}C{^{1}H}-NMR (C_6D_6, 100 MHz):** δ (ppm) = 54.8 (C-14), 59.3 (C-8), 75.3 (C-5), 79.8 (C-6), 93.3 (C-2), 113.9 (C-11), 126.9 (C-3), 127.2 (C-10), 131.1 (C-4), 136.4 (C-12), 159.3 (C-9); **GC-MS (EI):** ($C_{13}H_{16}O_3$), 221.0 (9, M^+ + H), 175.1 (100), 160.1 (25), 147.1 (20), 115.1 (21), 91.0 (15), 77.1 (10); **IR spectrum (neat)** (cm^{-1}) = 2931, 2844, 1610, 1510, 1295, 1176, 1106, 1078, 1024, 828, 706; $[\alpha]^{23}_D$ = +4.3 (*c* 0.87, CH_2Cl_2); **HPLC:** OJ-H, 204 nm, Hexane:*i*-PrOH, 98:2, 1 mL/min, 30 °C, t_{R1} = 31.8 and t_{R2} = 37.1 min.

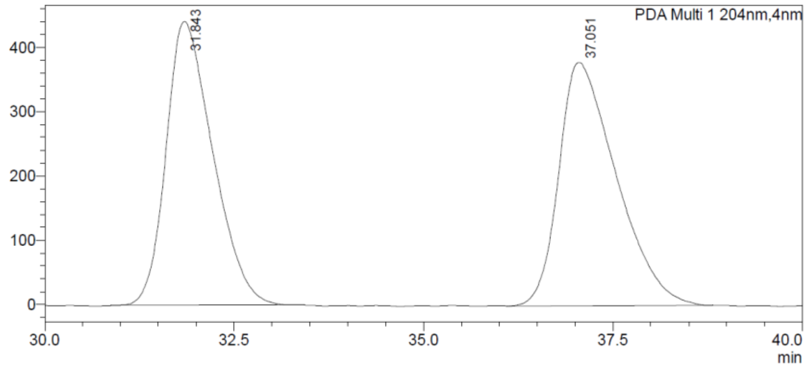

<Peak Table>

PDA Ch1 204nm

Peak#	Ret. Time	Area%
1	31.843	48.752
2	37.051	51.248
Total		100.000

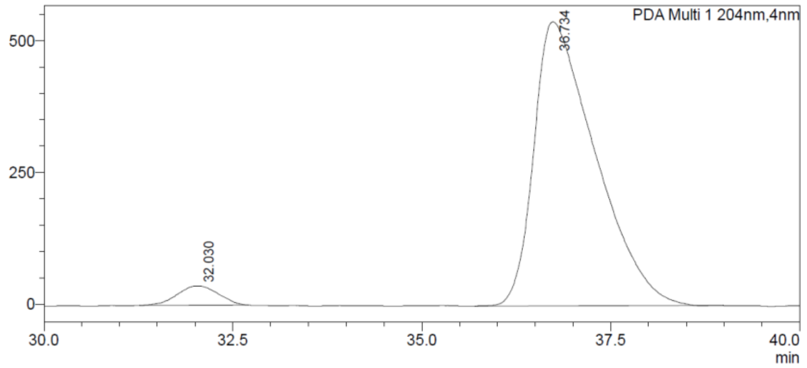

<Peak Table>

PDA Ch1 204nm

Peak#	Ret. Time	Area%
1	32.030	4.311
2	36.734	95.689
Total		100.000

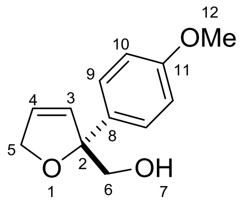

(*R*)-2-hydroxymethyl-2-(4-methoxyphenyl)-2,5-dihydrofuran (3eb)

Prepared according to **GP2** using 5-(methanolyl)-2,3-dihydrofuran (**1e**). Isolated by column chromatography (Cyclohexane:AcOEt 3:1) as a pale yellow oil (34% yield, 83% *ee*) with R_F = 0.13 (Cyclohexane:AcOEt 3:1); **^{1}H-NMR (C_6D_6, 400 MHz)** δ (ppm) = 1.72 (dd, $^3J_{H-H}$ = 8.1 Hz, $^3J_{H-H}$ = 5.2 Hz, 1H, H-7), 3.30 (s, 3H, H-12), 3.70 (dd, $^2J_{H-H}$ = 11.4 Hz, $^3J_{H-H}$ = 8.0 Hz, 1H, H-6), 3.77 (dd, $^2J_{H-H}$ = 11.4 Hz, $^3J_{H-H}$ = 5.0 Hz, 1H, H-6´), 4.42 (m, 2H, H-5), 5.46 (dt, $^3J_{H-H}$ = 6.1 Hz, $^4J_{H-H}$ = 1.7 Hz, 1H, H-3), 5.74 (dt, $^3J_{H-H}$ = 6.2 Hz, $^3J_{H-H}$ = 2.4 Hz, 1H, H-4), 6.79 (m, 2H, H-10), 7.24 (m, 2H, H-9); **^{13}C{^{1}H}-NMR (C_6D_6, 100 MHz):** δ (ppm) = 54.8 (C-12), 69.2 (C-6), 75.4 (C-5), 94.4 (C-2), 113.6 (C-10), 126.8 (C-9), 127.3 (C-3), 130.6 (C-4), 135.4 (C-8), 159.4 (C-11); **MS (ESI)**: $C_{12}H_{14}O_3$,205.1 [M-H]$^+$; **IR spectrum (neat)** (cm^{-1}) = 3437, 2853, 1610, 1510, 1460, 1359, 1247, 1176, 1082, 1038, 941, 881, 829, 732, 702, 633, 591; $[\alpha]^{23}_D$ = +81.0 (*c* 0.59, CH_2Cl_2); **HPLC:** IC, 226 nm, Hexane:*i*PrOH, 95:5, 1 mL/min, 30 °C, t_{R1} = 27.8 and t_{R2} = 38.1 min.

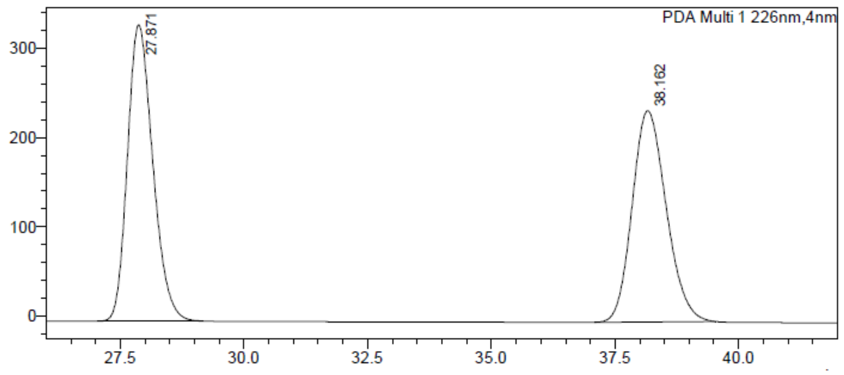

<Peak Table>

PDA Ch1 226nm

Peak#	Ret. Time	Area%
1	27.871	51.167
2	38.162	48.833
Total		100.000

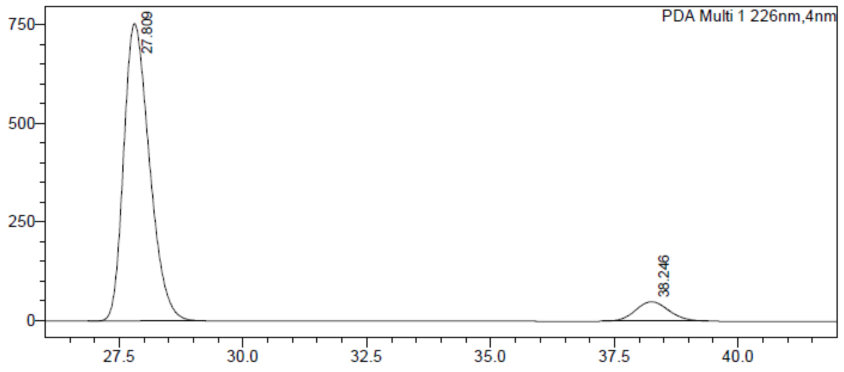

<Peak Table>

PDA Ch1 226nm

Peak#	Ret. Time	Area%
1	27.809	92.201
2	38.246	7.799
Total		100.000

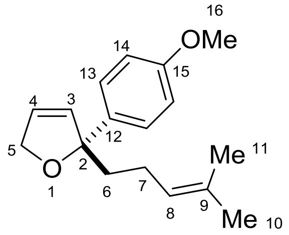

(*R*)-2-(4-methoxyphenyl)-2-(4-methylpent-3-en-1-yl)-2,5-dihydrofuran (3fb)

Prepared according to **GP2** using 5-(4-methylpent-3-en-1-yl)-2,3-dihydrofuran (**1f**). Isolated by column chromatography (Cyclohexane:AcOEt 90:1) as a pale yellow oil (43% yield, 94% *ee*) with R_F = 0.58 (Cyclohexane:AcOEt 10:1); **^{1}H-NMR (C_6D_6, 400 MHz)**: δ (ppm) = 1.54 (s, 3H, H-10 or H-11), 1.65 (s, 3H, H-10 or H-11), 1.96 (m, 1H, H-6), 2.06 (m, 1H, H-6´), 2.22 (m, 2H, H-7), 3.33 (s, 3H, H-16), 4.52 (ddd, $^2J_{H-H}$ = 12.9 Hz, $^3J_{H-H}$ = 2.4 Hz, $^4J_{H-H}$ = 1.7 Hz, 1H, H-5), 4.59 (ddd, $^2J_{H-H}$ = 12.9 Hz, $^3J_{H-H}$ = 2.4 Hz, $^4J_{H-H}$ = 1.7 Hz, 1H, H-5´), 5.24 (m, 1H, H-8), 5.42 (dt, $^3J_{H-H}$ = 6.1 Hz, $^4J_{H-H}$ = 1.6 Hz, 1H, H-3), 5.74 (dt, $^3J_{H-H}$ = 6.0 Hz, $^3J_{H-H}$ = 2.4 Hz, 1H, H-4), 6.84 (m, 2H, H-14), 7.34 (m, 2H, H-13); **^{13}C{^{1}H}-NMR (C_6D_6, 100 MHz)**: δ (ppm) = 17.6 (C-10 or C-11), 23.5 (C-7), 25.8 (C-10 or C-11), 42.3 (C-6), 54.8 (C-16), 75.1 (C-5), 93.4 (C-2), 113.9 (C-14), 125.2 (C-8), 125.5 (C-3), 126.4 (C-13), 131.1 (C-9), 133.3 (C-4), 138.8 (C-12), 158.9 (C-15); **MS (ESI)**: $C_{17}H_{22}O_2$, 259.5 $[M+H]^+$; **IR spectrum (neat)** (cm^{-1}) = 2916, 2840, 1619, 1509, 1159, 1377, 1299, 1243, 1175, 1071, 1031, 828, 808, 701, 575; $[\alpha]^{23}_D$ = +68.2 (*c* 0.91, CH_2Cl_2); **HPLC:** OJ-H, 205 nm, Hexane:*i*PrOH, 98:2, 1 mL/min, 30 °C, t_{R1} = 15.4 and t_{R2} = 18.1 min.

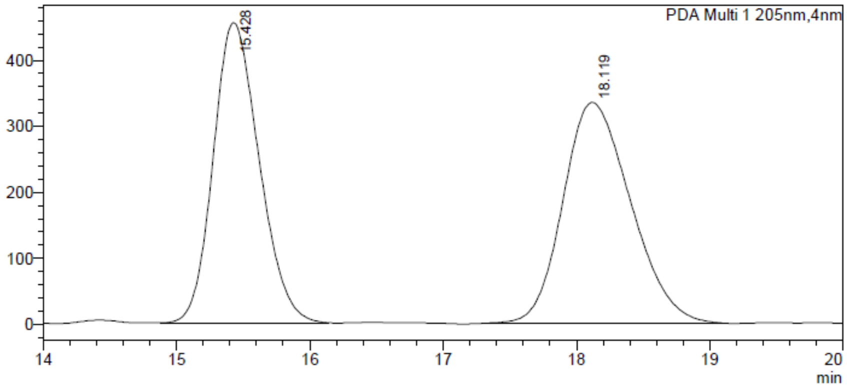

<Peak Table>

PDA Ch1 205nm

Peak#	Ret. Time	Area%
1	15.428	48.023
2	18.119	51.977
Total		100.000

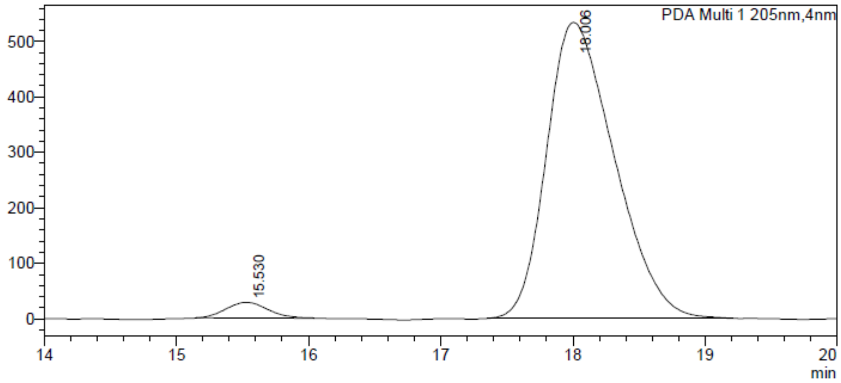

<Peak Table>

PDA Ch1 205nm

Peak#	Ret. Time	Area%
1	15.530	3.175
2	18.006	96.825
Total		100.000

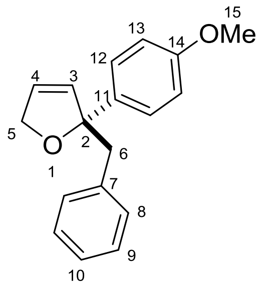

(*R*)-2-benzyl-2-(4-methoxyphenyl)-2,5-dihydrofuran (3gb)

Prepared according to **GP2** using 5-benzyl-2,3-dihydrofuran (**1g**). Isolated by column chromatography (Cyclohexane:AcOEt 70:1) as a pale yellow oil (50% yield, 94% *ee*) with R_F = 0.50 (Cyclohexane:AcOEt 10:1); **^{1}H-NMR (C_6D_6, 400 MHz)**: δ (ppm) = 3.08 (d, $^2J_{H-H}$ = 13.4 Hz, 1H, H-6), 3.23 (d, $^2J_{H-H}$ = 13.4 Hz, 1H, H-6´), 3.31 (s, 3H, H-15), 4.28 (ddd, $^2J_{H-H}$ = 12.8 Hz, $^3J_{H-H}$ = 2.5 Hz, $^4J_{H-H}$ = 1.6 Hz, H-5), 4.40 (ddd, $^2J_{H-H}$ = 12.8 Hz, $^3J_{H-H}$ = 2.5 Hz, $^4J_{H-H}$ = 1.6 Hz, H-5´), 5.28 (dt, $^3J_{H-H}$ = 6.1 Hz, $^4J_{H-H}$ = 1.6 Hz, H-3), 5.77 (dt, $^3J_{H-H}$ = 6.1 Hz, $^3J_{H-H}$ = 2.5 Hz, H-4), 6.81 (m, 2H, H-13), 7.04 (m, 5H, H-13, H-8 and H-9), 7.30 (m, 2H, H-12); **^{13}C{^{1}H}-NMR (C_6D_6, 100 MHz):** δ (ppm) = 48.9 (C-6), 54.7 (C-15), 75.2 (C-5), 93.8 (C-2), 113.8 (C-13), 126.1 (C-3), 126.3 (C-10), 126.7 (C-12), 128.0 (C-8 or C-9), 131.2 (C-8 or C-9), 132.4 (C-4), 138.0 (C-7 or C-11), 138.5 (C-7 or C-11), 159.0 (C-14); **MS (ESI)**: $C_{18}H_{18}O_2$, 267.5 $[M+H]^+$; **IR spectrum (neat)** (cm^{-1}) = 2837, 1605, 1502, 1454, 1346, 1299, 1241, 1174, 1114, 1077, 1035, 994, 953, 903, 837, 813, 724, 695, 637, 568; $[\alpha]^{23}_D$ = -225.1 (*c* 0.83, CH_2Cl_2); **HPLC:** OJ-H, 205nm, Hexane:*i*PrOH, 98:2, 1 mL/min,30 °C, t_{R1} = 39.9 and t_{R2} = 45.7 min.

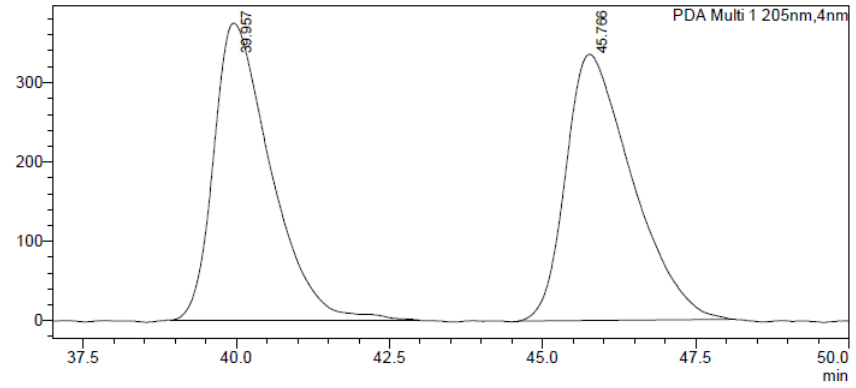

<Peak Table>

PDA Ch1 205nm

Peak#	Ret. Time	Area%
1	39.957	48.914
2	45.766	51.086
Total		100.000

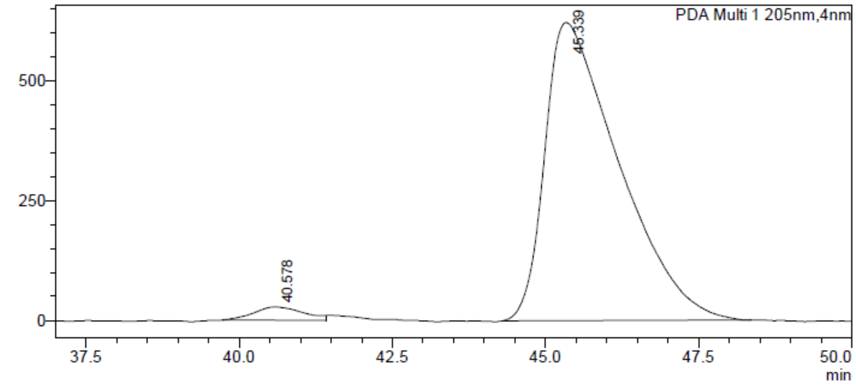

<Peak Table>

PDA Ch1 205nm

Peak#	Ret. Time	Area%
1	40.578	2.964
2	45.339	97.036
Total		100.000

4.8 Characterization data of 2-alkyl-2-aryl-2,3-dihydrofurans

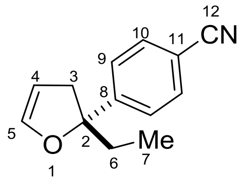

(*R*)-2-(4-cyanophenyl)-2-ethyl-2,3-dihydrofuran (4bc)

Prepared according to **GP3** using 5-ethyl-2,3-dihydrofuran (**1b**). Isolated by column chromatography (Cyclohexane:AcOEt 80:1) as a colorless oil (63% yield, 96% *ee*) with R_F = 0.44 (Cyclohexane:AcOEt 10:1); **^{1}H-NMR (C_6D_6, 400 MHz)**: δ(ppm) = 0.67 (t, $^3J_{H-H}$ = 7.3 Hz, 3H, H-7), 1.48 (dq, $^2J_{H-H}$ = 14.5 Hz, $^3J_{H-H}$ = 7.3 Hz, 1H, H-6), 1.68 (dq, $^2J_{H-H}$ = 14.5 Hz, $^3J_{H-H}$ = 7.3 Hz, 1H, H-6'), 2.29 (dt, $^2J_{H-H}$ = 15.2 Hz, $^3J_{H-H}$ = 2.4 Hz, 1H, H-3), 2.40 (dt, $^2J_{H-H}$ = 15.2 Hz, $^3J_{H-H}$ = 2.4 Hz, 1H, H-3'), 4.50-4.52 (m, 1H, H-4), 6.09-6.10 (m, 1H, H-5), 6.92-6.94 (m, 2H, H-9), 7.03-7.05 (m, 2H, H-10); **^{13}C{^{1}H}-NMR (C_6D_6, 100 MHz):** δ(ppm) = 8.2 (C-7), 35.1 (C-6), 42.7 (C-3), 89.6 (C-2), 98.6 (C-4), 111.3 (C-11), 118.9 (C-12), 125.8 (C-9), 132.0 (C-10), 144.5 (C-5), 151.7 (C-8); **GC-MS (EI):** ($C_{13}H_{13}NO$), 199.1 (9, M^+), 170.1 (100), 142.1 (40), 116.1 (30), 115.0 (30), 89.1 (10); **IR spectrum (neat)** (cm^{-1}) = 2971, 2930, 2229, 1625, 1608, 1155, 1055, 840, 707; $[\alpha]^{23}_D$ = +33.9 (*c* 1.00, CH_2Cl_2); **HPLC:** OJ-H, 229 nm, Hexane:*i*-PrOH, 98:2, 1 mL/min, 30 °C, t_{R1} = 8.6 and t_{R2} = 9.3 min.

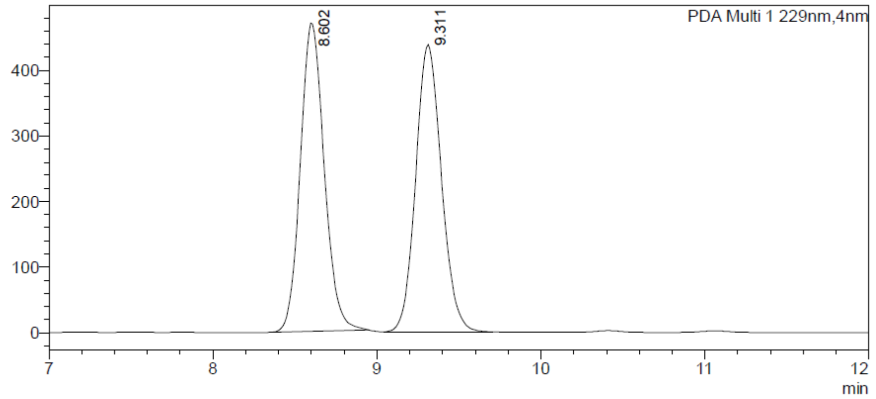

<Peak Table>

PDA Ch1 229nm

Peak#	Ret. Time	Area%
1	8.602	49.961
2	9.311	50.039
Total		100.000

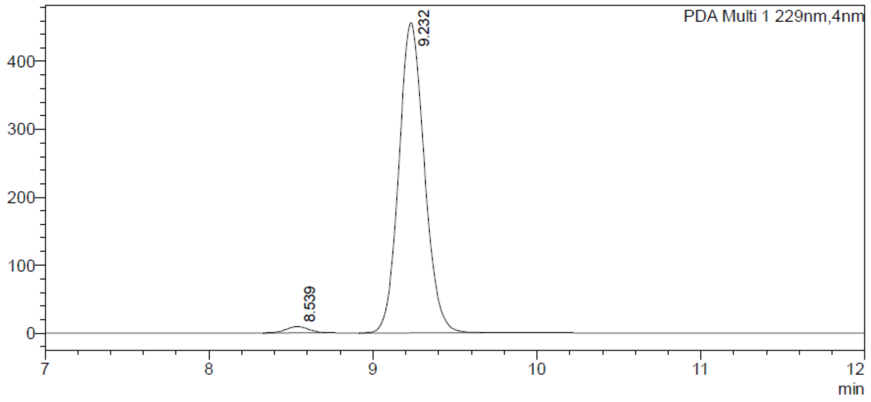

<Peak Table>

PDA Ch1 229nm

Peak#	Ret. Time	Area%
1	8.539	1.782
2	9.232	98.218
Total		100.000

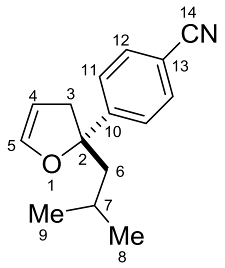

(*R*)-2-(4-cyanophenyl)-2-isobutyl-2,3-dihydrofuran (4cc)

Prepared according to **GP3** using 5-isobutyl-2,3-dihydrofuran (**1c**). Isolated by column chromatography (Cyclohexane:AcOEt 90:1) as a pale yellow oil (44% yield, 97% *ee*) with R_F = 0.54 (Cyclohexane:AcOEt 10:1); **^{1}H-NMR (C_6D_6, 400 MHz)**: δ(ppm) = 0.64 (d, $^3J_{H\text{-}H}$ = 6.5 Hz, 3H, H-8 or H-9), 0.89 (d, $^3J_{H\text{-}H}$ = 6.5 Hz, 3H, H-8 or H-9), 1.45 (m, 2H, H-6 and H-7), 1.65 (dd, $^2J_{H\text{-}H}$ = 14.1 Hz, $^3J_{H\text{-}H}$ = 6.7 Hz, 1H, H-6´), 2.30 (dt, $^2J_{H\text{-}H}$ = 15.2 Hz, $^3J_{H\text{-}H}$ = 2.4 Hz, 1H, H-3), 2.42 (dt, $^2J_{H\text{-}H}$ = 15.2 Hz, $^3J_{H\text{-}H}$ = 2.4 Hz, 1H, H-3´), 4.50 (m, 1H, H-4), 6.00 (m, 1H, H-5), 6.93 (m, 2H, H-11), 7.04 (m, 2H, H-12); **^{13}C{^{1}H}-NMR (C_6D_6, 100 MHz):** δ(ppm) = 23.9 (C-8 or C-9), 24.3 (C-8 or C-9), 24.8 (C-7), 44.8 (C-3), 50.6 (C-6), 89.7 (C-2), 98.5 (C-4), 111.1 (C-13), 118.8 (C-14), 125.8 (C-11), 132.0 (C-12), 144.4 (C-5), 152.2 (C-10); **MS (ESI)**: $C_{15}H_{17}NO$, 226.1 $[M\text{-}H]^+$. **IR spectrum (neat)** (cm^{-1}) = 2954, 2910, 2229, 1624, 1608, 1502, 1466, 1387, 1279, 1155, 1123, 1056, 967, 844, 704, 631; $[\alpha]^{23}_D$= +37.1 (*c* 0.85, CH_2Cl_2); **HPLC:** OJ-H, 229 nm, Hexane:*i*PrOH, 98:2, 1 mL/min, 30 °C, t_{R1} = 6.5 and t_{R2} = 6.9 min.

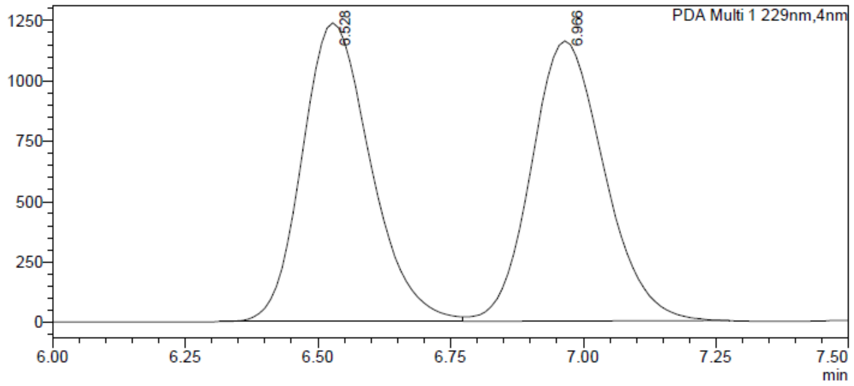

<Peak Table>

PDA Ch1 229nm

Peak#	Ret. Time	Area%
1	6.528	50.004
2	6.966	49.996
Total		100.000

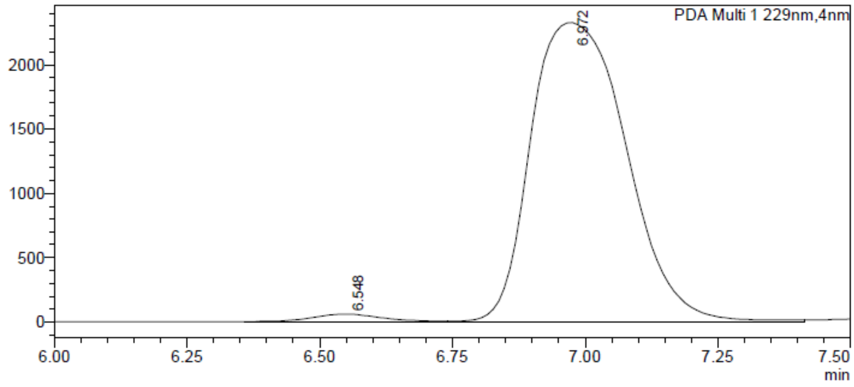

<Peak Table>

PDA Ch1 229nm

Peak#	Ret. Time	Area%
1	6.548	1.682
2	6.972	98.318
Total		100.000

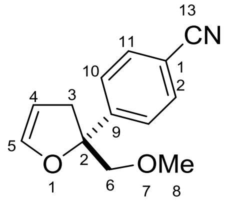

(*R*)-2-(4-cyanophenyl)-2-(methoxymethyl)-2,3-dihydrofuran (4dc)

Prepared according to **GP2** using 5-(methoxymethyl)-2,3-dihydrofuran (**1d**). Isolated by column chromatography (Cyclohexane:AcOEt 10:1) as a colorless oil (63% yield, 96% *ee*) with R_F = 0.45 (Cyclohexane:AcOEt 2:1); **^{1}H-NMR (C_6D_6, 400 MHz)**: δ (ppm) = 2.29 (dt, $^2J_{H-H}$ = 15.2 Hz, $^3J_{H-H}$ = 2.4 Hz, H-3), 2.81 (dt, $^2J_{H-H}$ = 15.3 Hz, $^3J_{H-H}$ = 2.4 Hz, 1H, H-3'), 2.98 (s, 3H, H-8), 3.21 (d, $^2J_{H-H}$ = 9.9 Hz, 1H, H-6), 3.25 (d, $^2J_{H-H}$ = 10.0 Hz, 1H, H-6'), 4.56-4.58 (m, 1H, H-4), 6.10-6.12 (m, 1H, H-5), 7.06-7.08 (m, 2H, H-11), 7.11-7.13 (m, 2H, H-10); **^{13}C{^{1}H}-NMR (C_6D_6, 100 MHz):** δ (ppm) = 39.4 (C-3), 59.2 (C-8), 77.9 (C-6), 88.7 (C-2), 99.1 (C-4), 111.7 (C-12), 118.9 (C-13), 126.4 (C-10), 131.9 (C-11), 144.6 (C-5), 149.8 (C-9); **GC-MS (EI):** ($C_{13}H_{13}NO_2$), 215.1 (3, M^+), 170.1 (100), 152.0 (14), 142.1 (31), 116.0 (28), 102.0 (12), 89.0 (17); **IR spectrum (neat)** (cm^{-1}) = 2926, 2228, 1623, 1149, 1108, 1056, 841, 702, 557; $[\alpha]^{23}_D$ = +38.5 (*c* 0.88, CH_2Cl_2); **HPLC:** AD-H, 204 nm, Hexane:*i*-PrOH, 98:2, 1 mL/min, 30 °C, t_{R1} = 9.4 and t_{R2} = 11.3 min.

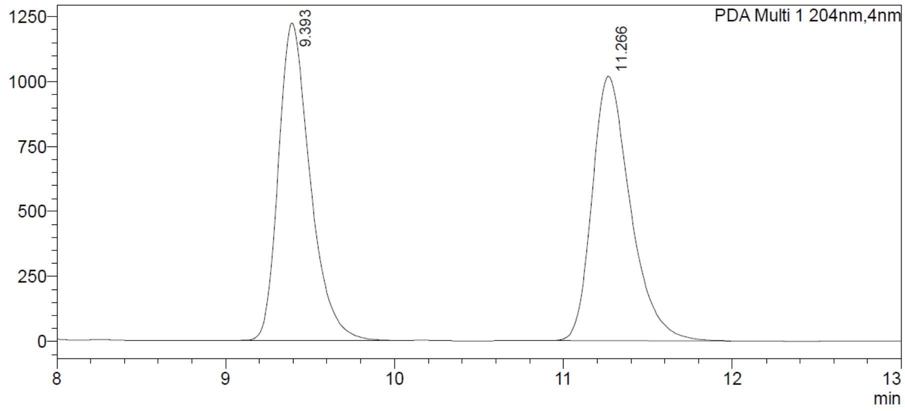

<Peak Table>

PDA Ch1 204nm

Peak#	Ret. Time	Area%
1	9.393	49.891
2	11.266	50.109
Total		100.000

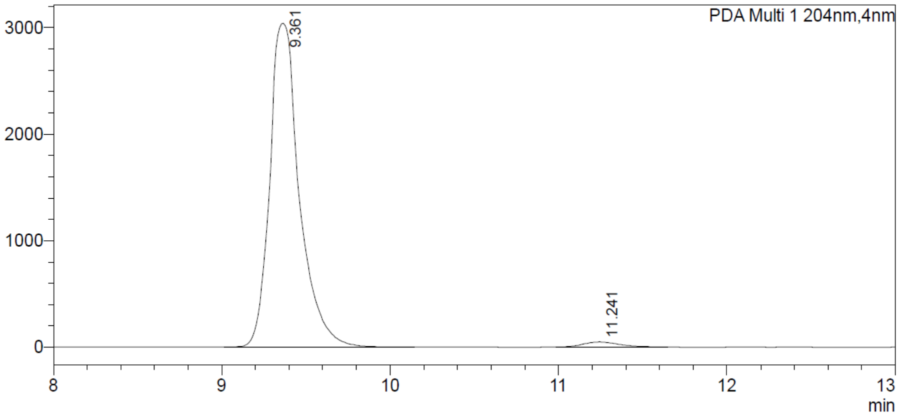

<Peak Table>

PDA Ch1 204nm

Peak#	Ret. Time	Area%
1	9.361	98.102
2	11.241	1.898
Total		100.000

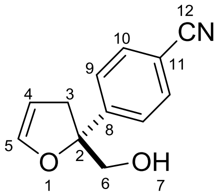

(*R*)-2-(4-cyanophenyl)-2-hydroxymethyl-2,3-dihydrofuran (4ec)

Prepared according to **GP2** using 5-(methanolyl)-2,3-dihydrofuran (**1e**). Isolated by column chromatography (Cyclohexane:AcOEt 4:1) as a pale yellow oil (37% yield, 92% *ee*) with R_F = 0.14 (Cyclohexane:AcOEt 4:1). **^{1}H-NMR (C_6D_6, 400 MHz) :** δ (ppm) = 1.36 (m, 1H, H-7), 2.17 (dt, $^2J_{H-H}$ = 15.2 Hz, $^3J_{H-H}$ = 2.4 Hz, 1H, H-3), 2.65 (dt, $^2J_{H-H}$ = 15.2 Hz, $^3J_{H-H}$ = 2.4 Hz, 1H, H-3´), 3.32 (m, 2H, H-6), 4.51 (m, 2H, H-4), 6.00 (m, 2H, H-5), 6.90 (m, 2H, H-9), 7.00 (m, 2H, H-10); **^{13}C{^{1}H}-NMR(C_6D_6, 100 MHz):** δ (ppm) = 38.7 (C-3), 68.4 (C-6), 89.7 (C-2), 99.7 (C-4), 111.8 (C-11), 118.7 (C-12), 126.0 (C-9), 132.0 (C-10), 144.2 (C-5), 149.0 (C-8); **MS (ESI)**: $C_{12}H_{11}NO_2$, 202.1 $[M+H]^+$; **IR spectrum (neat)** (cm^{-1}) = 3428, 2976, 2864, 2229, 1625, 1609, 1503, 1448, 1383, 1286, 1150, 1113, 1052, 968, 908, 841, 708, 628, 558; $[\alpha]^{23}_D$ = + 9.7 (*c* 0.90, CH_2Cl_2); **HPLC:** OD-H, 228 nm, Hexane:*i*PrOH, 95:5, 1 mL/min, 30 °C, t_{R1} = 22.8 and t_{R2} = 24.8 min.

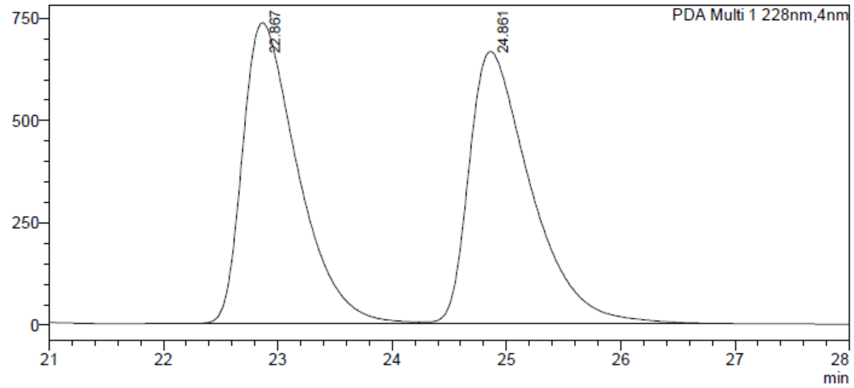

<Peak Table>

PDA Ch1 228nm

Peak#	Ret. Time	Area%
1	22.867	49.781
2	24.861	50.219
Total		100.000

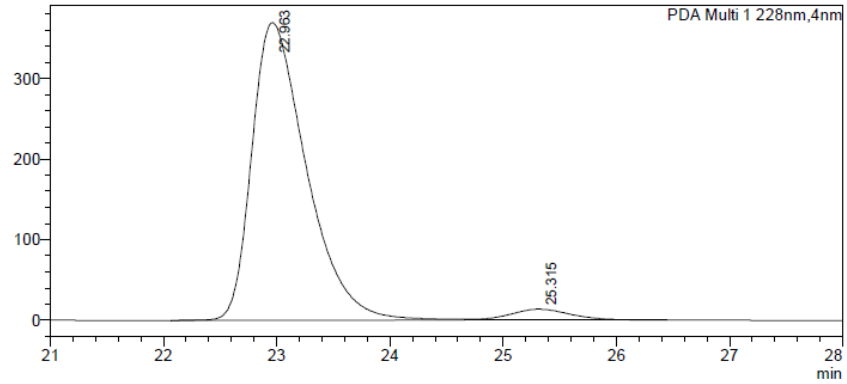

<Peak Table>

PDA Ch1 228nm

Peak#	Ret. Time	Area%
1	22.963	96.072
2	25.315	3.928
Total		100.000

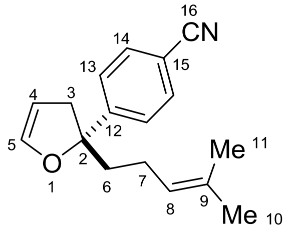

(*R*)-2-(4-cyanophenyl)-2-(4-methylpent-3-en-1-yl)-2,3-dihydrofuran (4fc)

Prepared according to **GP2** using 5-(4-methylpent-3-en-1-yl)-2,3-dihydrofuran (**1f**). Isolated by column chromatography (Cyclohexane:AcOEt 85:1) as a pale yellow oil (58% yield, 98% *ee*) with R_F = 0.60 (Cyclohexane:AcOEt 10:1); **^{1}H-NMR (C_6D_6, 400 MHz)**: δ (ppm) = 1.42 (s, 3H, H-10 or H-11), 1.60 (H-10 or H-11), 1.75-1.83 (m, 3H, H-6, H-6′ and H-7), 2.1 (m, 1H, H-7′), 2.32 (dt, $^2J_{H-H}$ = 15.2 Hz, $^3J_{H-H}$ = 2.4 Hz, 1H, H-3), 2.47 (dt, $^2J_{H-H}$ = 15.2 Hz, $^3J_{H-H}$ = 2.4 Hz, 1H, H-3′), 4.5 (m, 1H, H-4), 5.05 (m, 1H, H-14), 6.1 (m, 1H, H-5), 6.97 (m, 2H, H-13), 7.05 (m, 2H, H-14); **^{13}C{^{1}H}-NMR (C_6D_6, 100 MHz)**: δ (ppm) = 17.5 (C-10 or C-11), 22.9 (C-7), 25.7 (C-10 or C-11), 42.5 (C-6), 43.2 (C-3), 89.2 (C-2), 98.6 (C-4), 111.2 (C-15), 118.8 (C-16), 124.1 (C-8), 125.7 (C-13), 131.8 (C-9), 132.0 (C-14), 144.5 (C-5), 151.8 (C-12); **MS (ESI)**: $C_{17}H_{19}NO$, 254.6 $[M+H]^+$; **IR spectrum (neat)** (cm^{-1}) = 2921, 2229, 1624, 1609, 1503, 1448, 1407, 1377, 1327, 1147, 1124, 1053, 1016, 965, 930, 882, 842, 775, 706, 560; $[\alpha]^{23}_D$= +57.2 (*c* 0.85, CH_2Cl_2); **HPLC:** OJ-H, 205 nm,Hexane:*i*PrOH, 98:2, 1 mL/min, 30 °C, t_{R1} = 7.4 and t_{R2} = 8.1 min.

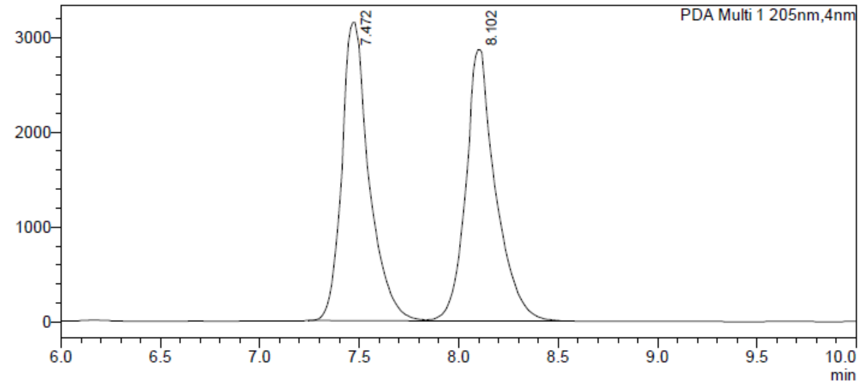

<Peak Table>

PDA Ch1 205nm

Peak#	Ret. Time	Area%
1	7.472	50.150
2	8.102	49.850
Total		100.000

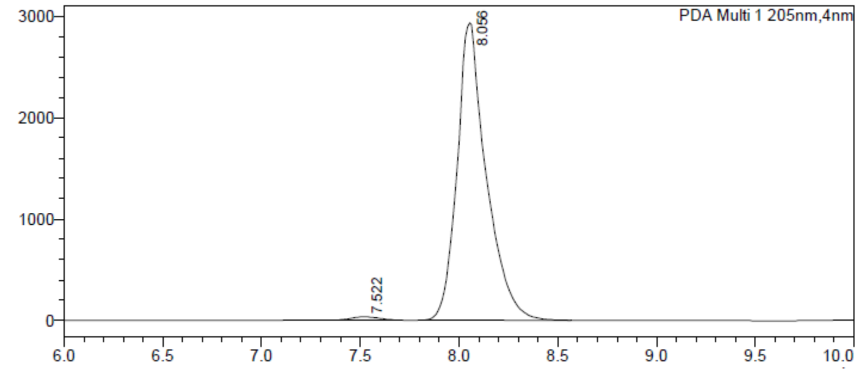

<Peak Table>

PDA Ch1 205nm

Peak#	Ret. Time	Area%
1	7.522	1.051
2	8.056	98.949
Total		100.000

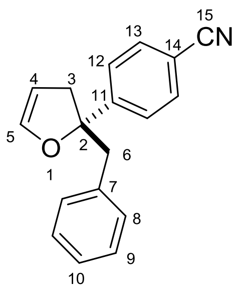

(*R*)-2-(4-cyanophenyl)-2-benzyl-2,3-dihydrofuran (4gc)

Prepared according to **GP2** using 5-benzyl-2,3-dihydrofuran (**1g**). Isolated by column chromatography (Cyclohexane:AcOEt 80:1) as a pale yellow oil (64% yield, 98% *ee*) with R_F = 0.45 (Cyclohexane:AcOEt 10:1); **^{1}H-NMR (C_6D_6, 400 MHz)**: δ(ppm) = 2.35 (dt, $^2J_{H\text{-}H}$ = 15.1 Hz, $^3J_{H\text{-}H}$ = 2.4 Hz, 1H, H-3), 2.55 (dt, $^2J_{H\text{-}H}$ = 15.1 Hz, $^3J_{H\text{-}H}$ = 2.4 Hz, 1H, H-3´), 2.71 (d, $^2J_{H\text{-}H}$ = 13.7 Hz, H-6), 2.92 (d, $^2J_{H\text{-}H}$ = 13.7 Hz, H-6´), 4.49 (m, 1H, H-4), 6.05 (m, 1H, H-5), 6.80 (m, 2H, H-12), 6.83 (m, 2H, H-8), 6.95 (m, 2H, H-13), 7.02 (m, 3H, H-9 and H-10); **^{13}C{^{1}H}-NMR(C_6D_6, 100 MHz):** δ(ppm) = 42.1 (C-3), 48.0 (C-6), 89.5 (C-2), 99.0 (C-4), 111.3 (C-14), 118.8 (C-15), 126.2 (C-12), 126.9 (C-10), 128.0(C-9), 130.8 (C-8), 131.7 (C-13), 136.1 (C-7), 144.2 (C-5), 151.1 (C-11); **MS (ESI)**: $C_{18}H_{15}NO$, 262.0 $[M+H]^+$; **IR spectrum (neat)** (cm^{-1}) = 2919, 2228, 1624, 1607, 1497, 1453, 1403, 1333, 1281, 1146, 1055, 994, 970, 843, 753, 698, 628; $[\alpha]^{23}_D$= -41.5 (*c* 0.86, CH_2Cl_2); **HPLC:** OJ-H, 205 nm, Hexane:*i*PrOH, 98:2, 1 mL/min,30 °C, t_{R1} = 25.7 and t_{R2} = 28.2 min.

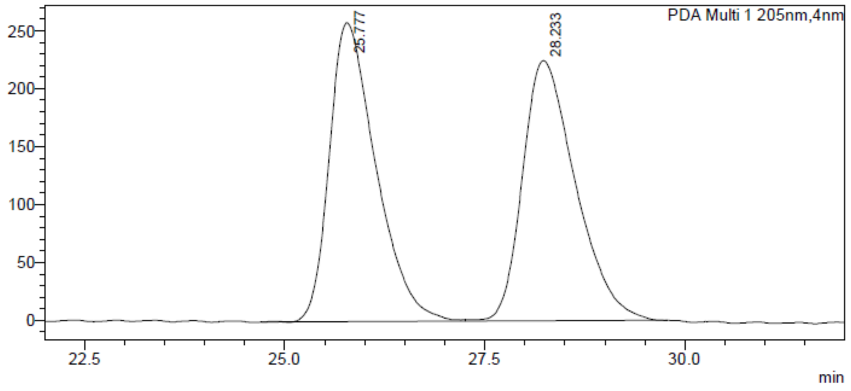

<Peak Table>

PDA Ch1 205nm

Peak#	Ret. Time	Area%
1	25.777	50.241
2	28.233	49.759
Total		100.000

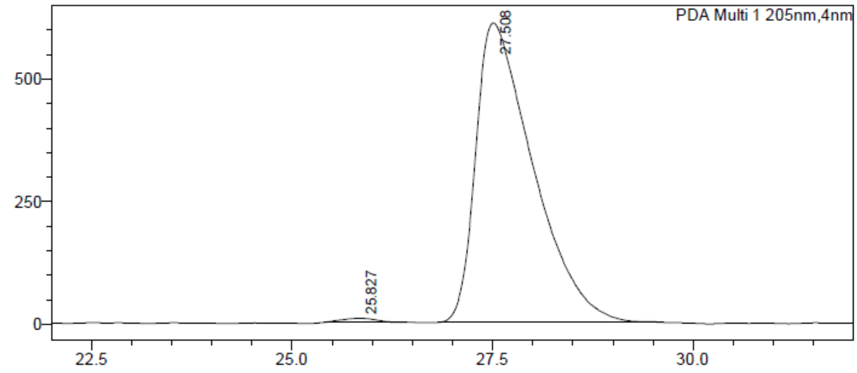

<Peak Table>

PDA Ch1 205nm

Peak#	Ret. Time	Area%
1	25.827	0.798
2	27.508	99.202
Total		100.000

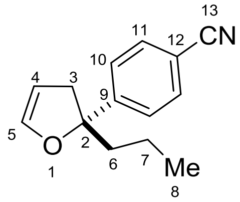

(*R*)-2-(4-cyanophenyl)-2-propyl-2,3-dihydrofuran

Prepared according to **GP3** using 5-propyl-2,3-dihydrofuran (**xxx**). Isolated by column chromatography (Cyclohexane:AcOEt 80:1) as a colorless oil (66% yield, 96% *ee*) with R_F = 0.5 (Cyclohexane:AcOEt 10:1); **^{1}H-NMR (C_6D_6, 400 MHz)**: δ (ppm) = 0.72 (t, $^3J_{H\text{-}H}$ = 7.3 Hz, 3H, H-8), 0.89-0.99 (m, 1H, H-7), 1.25-1.32 (m, 1H, H-7'), 1.46 (ddd, $^2J_{H\text{-}H}$ = 13.6 Hz, $^3J_{H\text{-}H}$ = 11.7 Hz, $^3J_{H\text{-}H}$ = 4.5 Hz, 1H, H-6), 1.65 (ddd, $^2J_{H\text{-}H}$ = 13.7 Hz, $^3J_{H\text{-}H}$ = 11.6 Hz, $^3J_{H\text{-}H}$ = 4.6 Hz, 1H, H-6'), 2.31 (dt, $^2J_{H\text{-}H}$ = 15.2 Hz, $^3J_{H\text{-}H}$ = 2.4 Hz, 1H, H-3), 2.42 (dt, $^2J_{H\text{-}H}$ = 15.2 Hz, $^3J_{H\text{-}H}$ = 2.4 Hz, 1H, H-3'), 4.51-4.53 (m, 1H, H-4), 6.09-6.11 (m, 1H, H-5), 6.93-6.96 (m, 2H, H-10), 7.04-7.07 (m, 2H, H-11); **^{13}C{^{1}H}-NMR (C_6D_6, 100 MHz):** δ (ppm) = 14.3 (C-8), 17.4 (C-7), 43.2 (C-3), 44.7 (C-6), 89.4 (C-2), 98.5 (C-4), 111.3 (C-12), 118.9 (C-13), 125.8 (C-10), 132.0 (C-11), 144.5 (C-5), 152.06 (C-9); **GC-MS (EI):** ($C_{14}H_{15}NO$), 213.1 (11, M^+), 170.1 (100), 154.0 (23), 142.1 (40), 127.1 (13), 116.0 (33), 102.0 (10); **IR spectrum (neat)** (cm^{-1}) = 2958, 2872, 2280, 1624, 1608, 1457, 1155, 1051, 842, 704, 557; $[\alpha]^{23}_D$ = +42.4 (*c* 0.41, CH_2Cl_2); **HPLC:** OJ-H, 226 nm, Hexane:*i*-PrOH, 98:2, 1 mL/min, 30°C, t_{R1} = 7.4 and t_{R2} = 7.8 min.

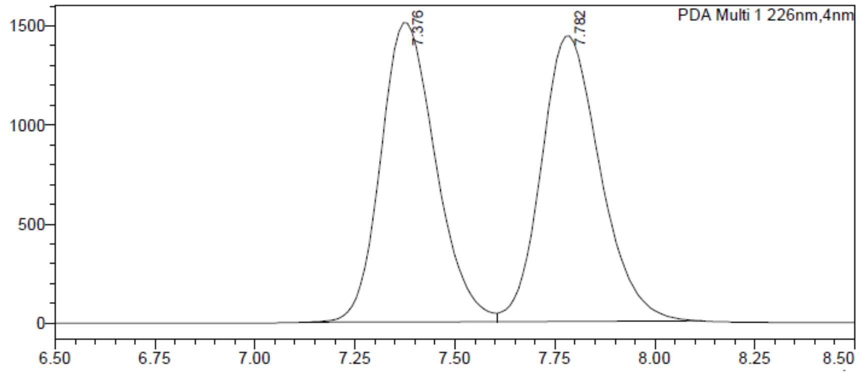

<Peak Table>

PDA Ch1 226nm

Peak#	Ret. Time	Area%
1	7.376	49.437
2	7.782	50.563
Total		100.000

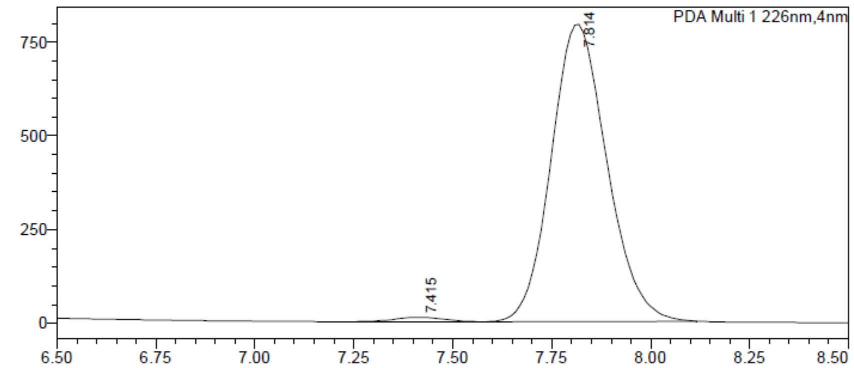

<Peak Table>

PDA Ch1 226nm

Peak#	Ret. Time	Area%
1	7.415	1.270
2	7.814	98.730
Total		100.000

5 NMR spectra of all compounds

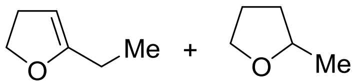

A **B**

(**A**:**B** = 1:1.5)

1b

^{1}H-NMR

C_6D_6, 400 MHz, 298 K

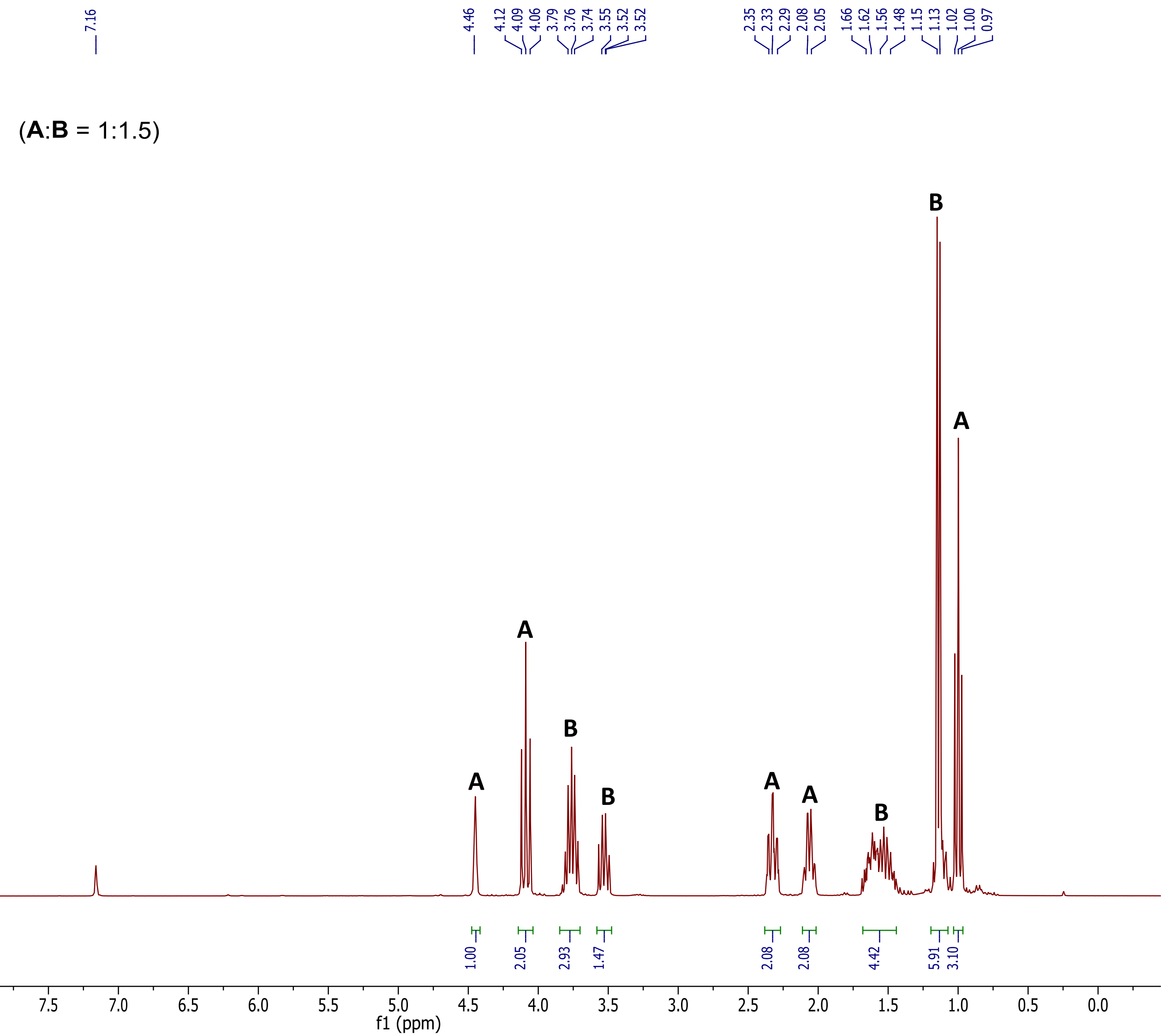

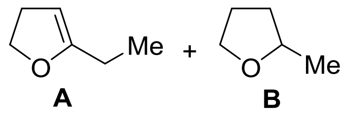

1b
$^{13}C\{^1H\}$-NMR
C_6D_6, 100 MHz, 298 K

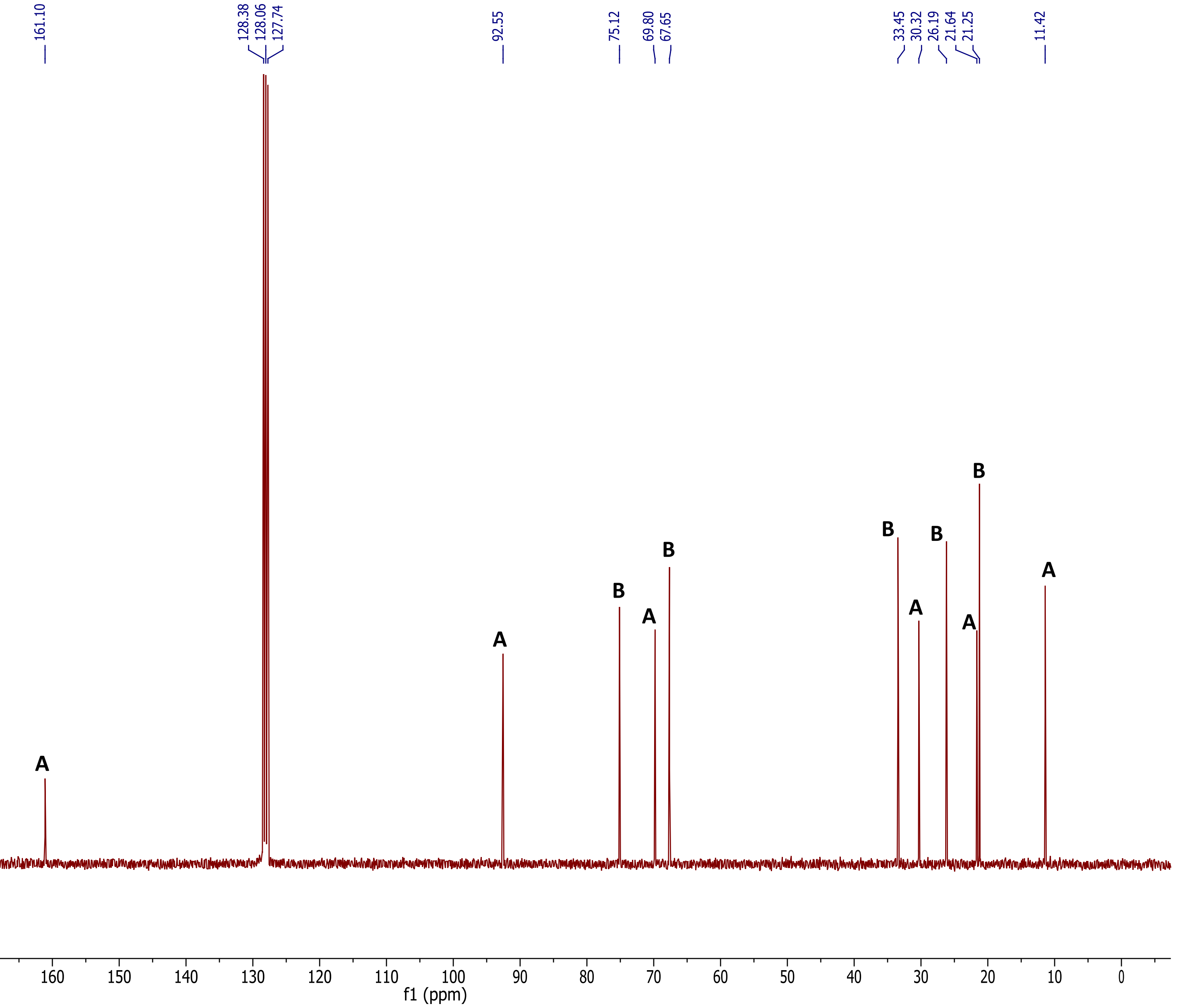

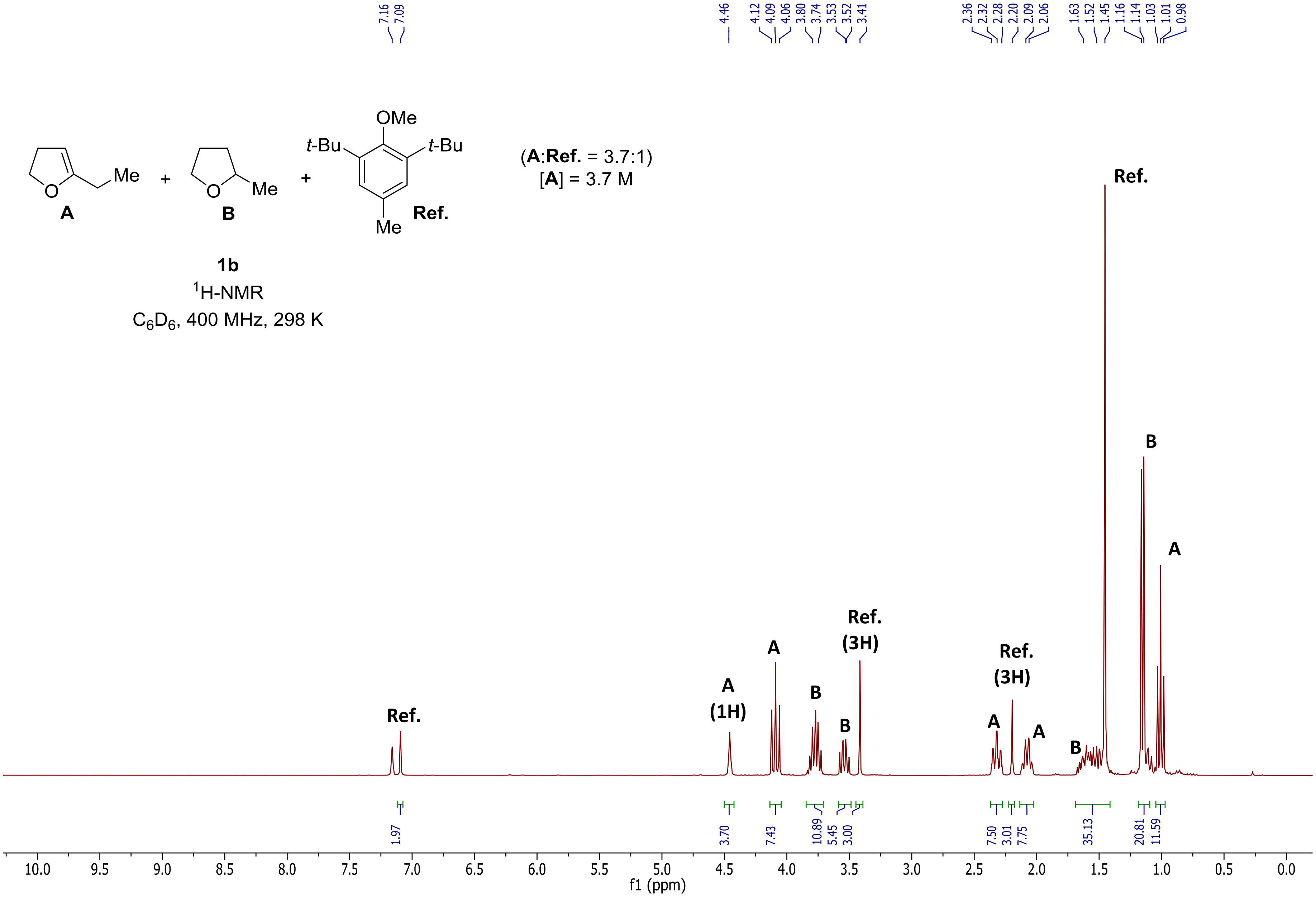

OMe
t-Bu
t-Bu
Me
Me
Me
A
B
Ref.
1b
1H-NMR
C6D6, 400 MHz, 298 K
(A:Ref. = 3.7:1)
[A] = 3.7 M
7.16
7.09
4.46
4.12
4.09
4.06
3.80
3.74
3.53
3.52
3.41
2.36
2.32
2.28
2.20
2.09
2.06
1.63
1.52
1.45
1.16
1.14
1.03
1.01
0.98
Ref.
A (1H)
A
B
B
Ref. (3H)
Ref. (3H)
A
A
B
Ref.
B
A
1.97
3.70
7.43
10.89
5.45
3.00
7.50
3.01
7.75
35.13
20.81
11.59
10.0
9.5
9.0
8.5
8.0
7.5
7.0
6.5
6.0
5.5
5.0
4.5
4.0
3.5
3.0
2.5
2.0
1.5
1.0
0.5
0.0
f1 (ppm)

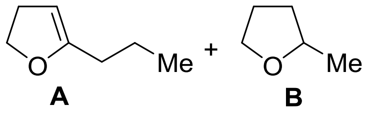

(**A**:**B** = 1:1.33)

^{1}H-NMR
C_6D_6, 400 MHz, 298 K

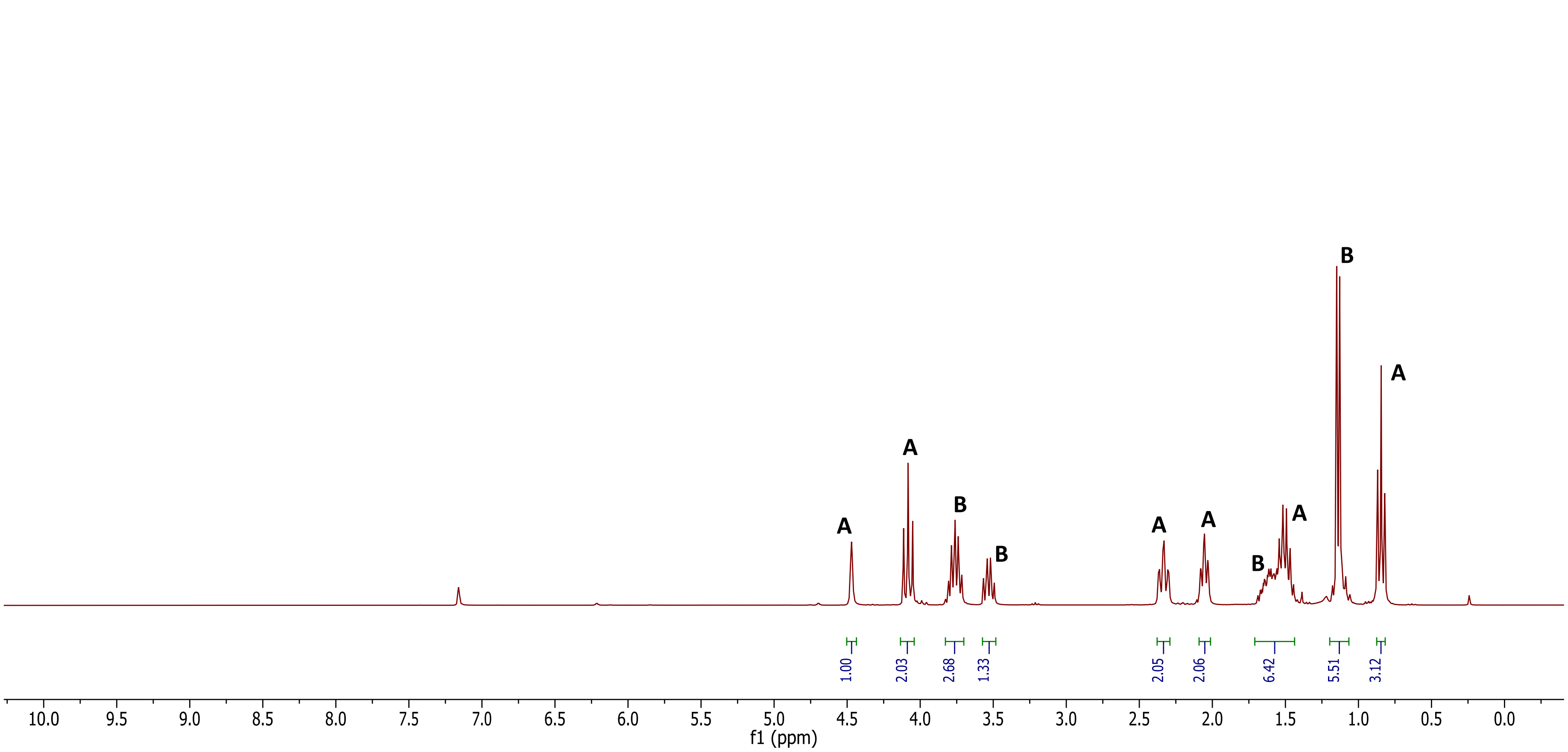

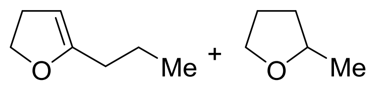

A + **B**

$^{13}C\{^1H\}$-NMR
C_6D_6, 100 MHz, 298 K

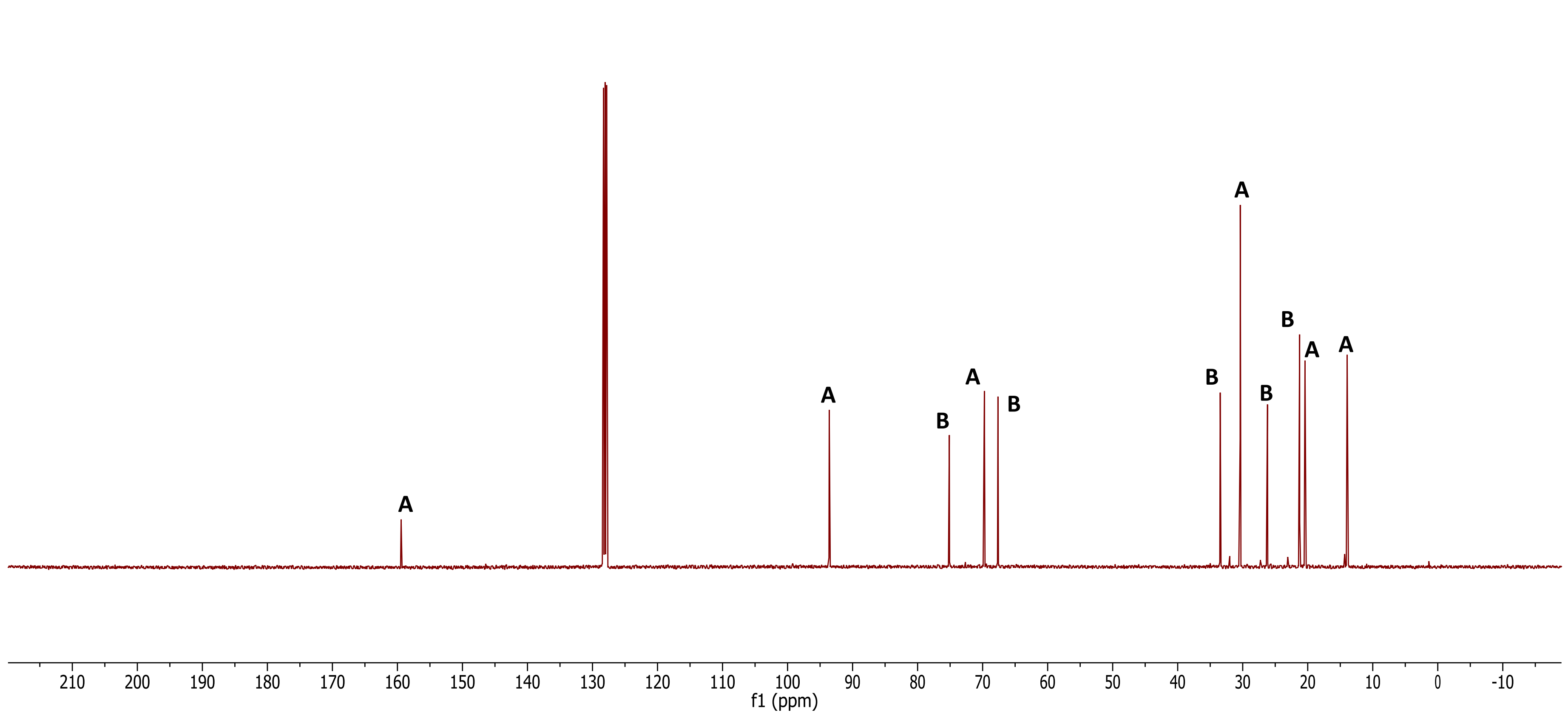

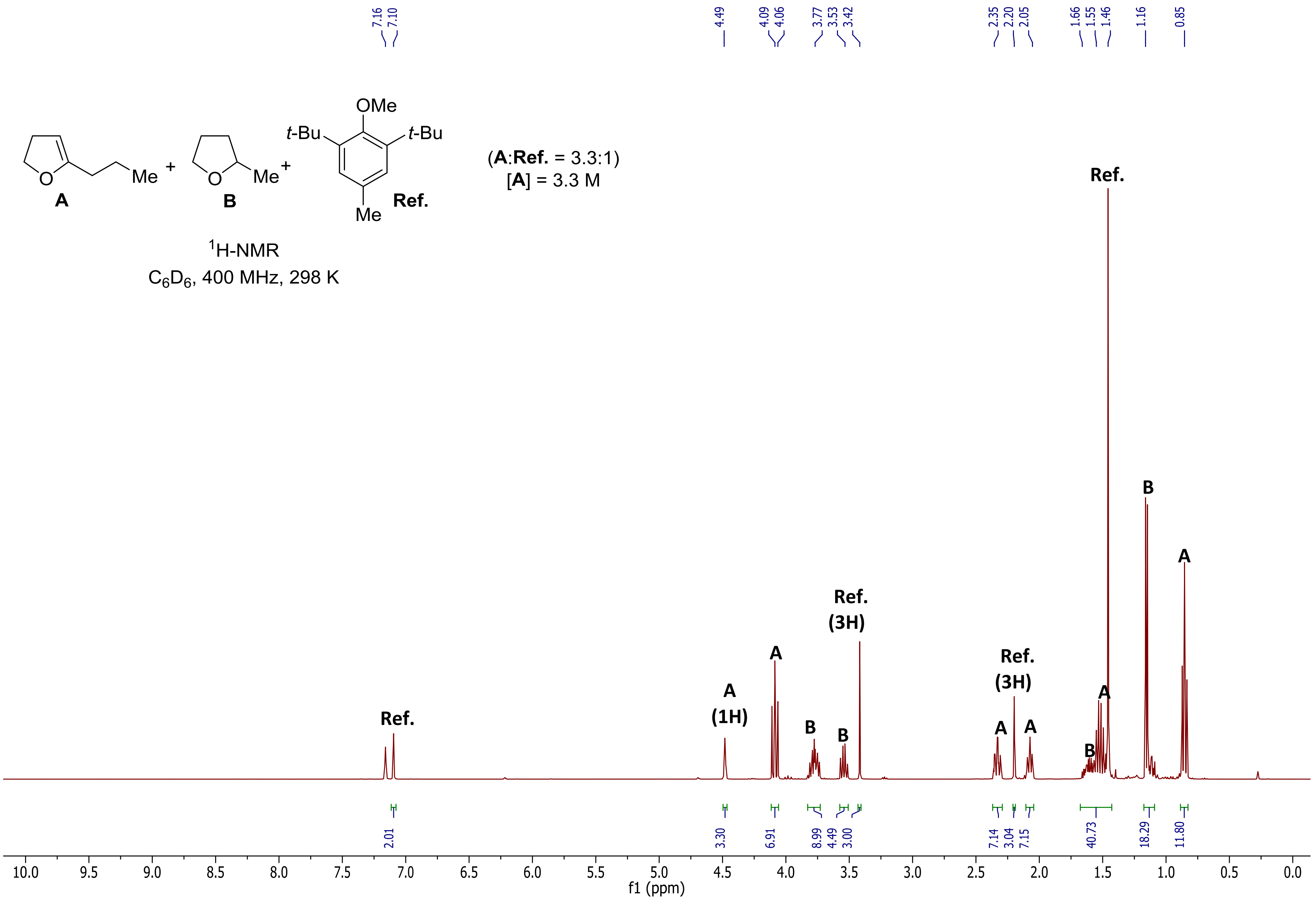
A
B
Ref.
OMe
t-Bu
t-Bu
Me
Me
(A:Ref. = 3.3:1)
[A] = 3.3 M
1H-NMR
C6D6, 400 MHz, 298 K
7.16
7.10
4.49
4.09
4.06
3.77
3.53
3.42
2.35
2.20
2.05
1.66
1.55
1.46
1.16
0.85
Ref.
A (1H)
Ref. (3H)
2.01
3.30
6.91
8.99
4.49
3.00
7.14
3.04
7.15
40.73
18.29
11.80
f1 (ppm)

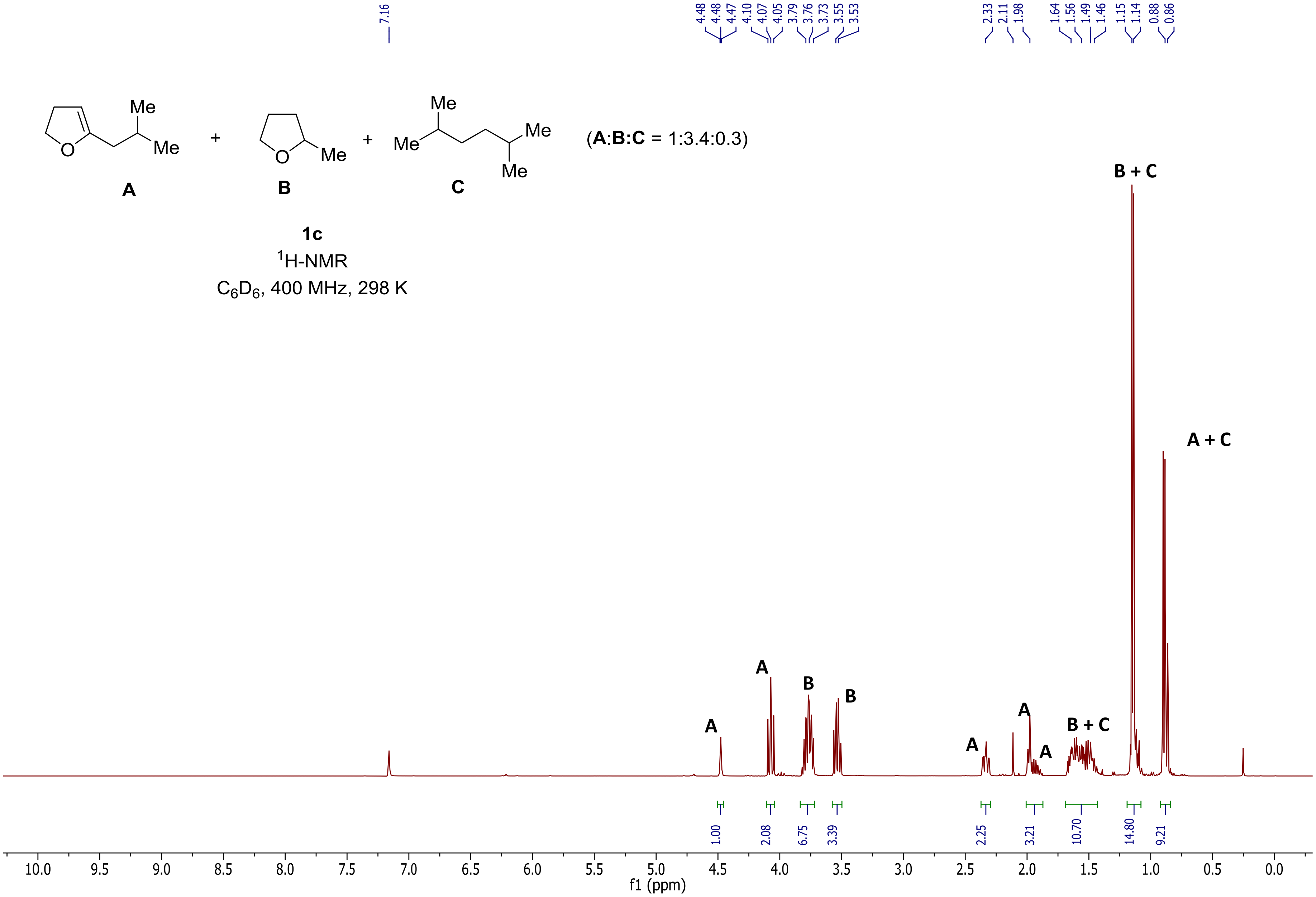
7.16
4.48
4.48
4.47
4.10
4.07
4.05
3.79
3.76
3.73
3.55
3.53
2.33
2.11
1.98
1.64
1.56
1.49
1.46
1.15
1.14
0.88
0.86
Me
Me
Me
Me
Me
Me
Me
A
B
C
(A:B:C = 1:3.4:0.3)
1c
1H-NMR
C6D6, 400 MHz, 298 K
B + C
A + C
A
A
B
B
A
A
A
B + C
1.00
2.08
6.75
3.39
2.25
3.21
10.70
14.80
9.21
10.0
9.5
9.0
8.5
8.0
7.5
7.0
6.5
6.0
5.5
5.0
4.5
4.0
3.5
3.0
2.5
2.0
1.5
1.0
0.5
0.0
f1 (ppm)

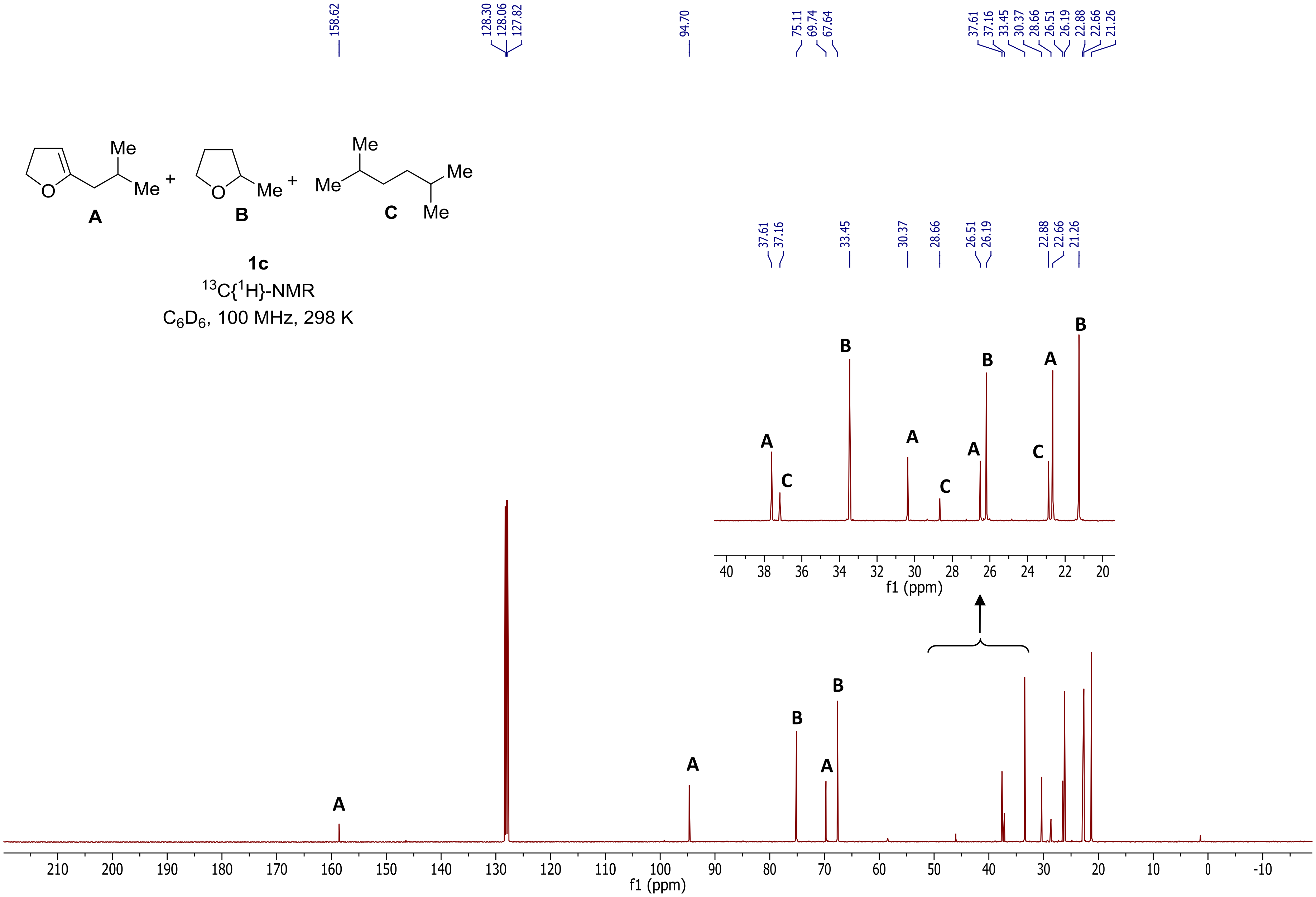
158.62
128.30
128.06
127.82
94.70
75.11
69.74
67.64
37.61
37.16
33.45
30.37
28.66
26.51
26.19
22.88
22.66
21.26
Me
Me
O
A
+
O
Me
B
+
Me
Me
Me
C
Me
1c
$^{13}C\{^{1}H\}$-NMR
C_6D_6, 100 MHz, 298 K
A
B
B
A
f1 (ppm)

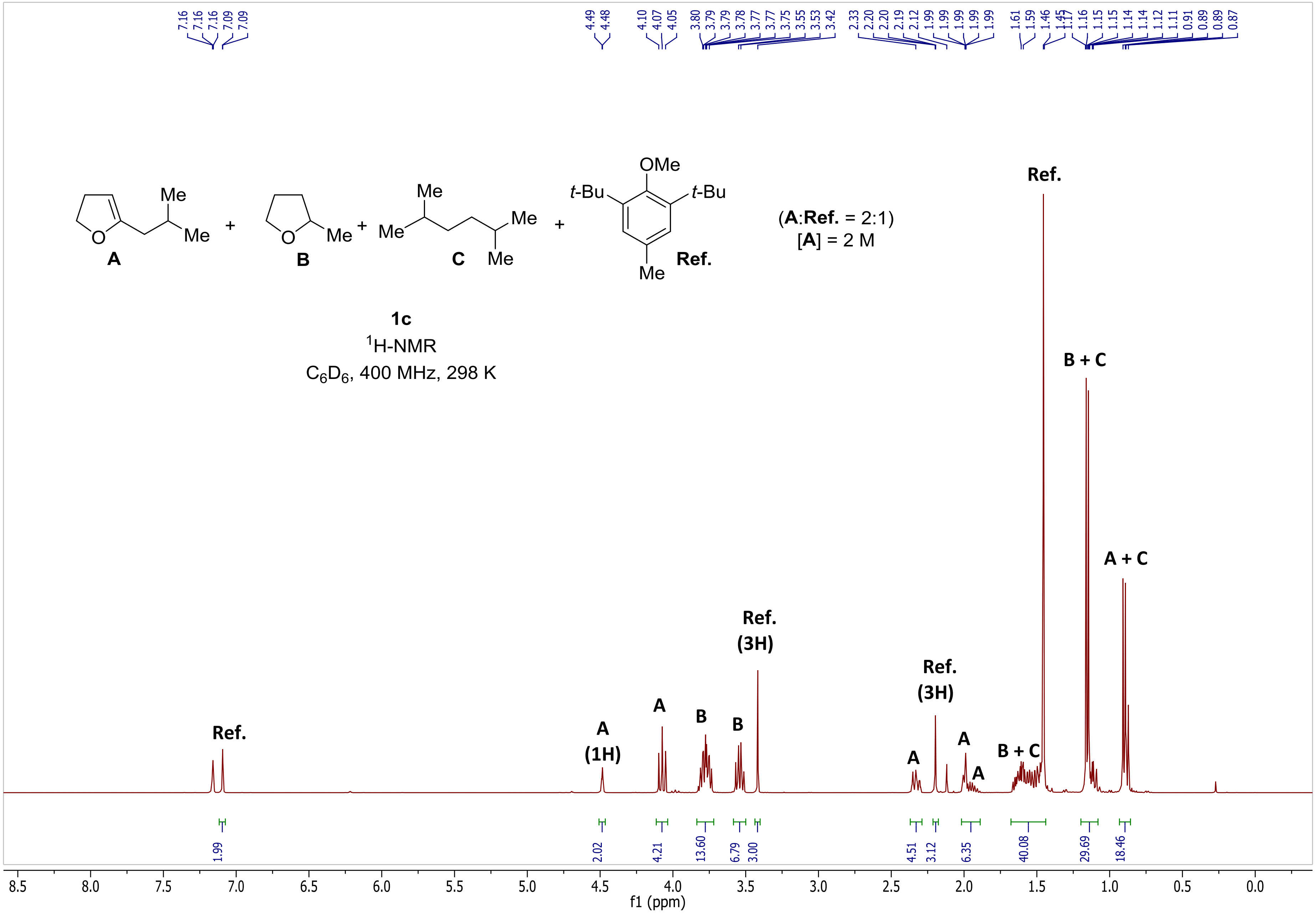
A
B
C
Ref.
1c
¹H-NMR
C6D6, 400 MHz, 298 K
(A:Ref. = 2:1)
[A] = 2 M
Ref.
B + C
A + C
Ref. (3H)
A (1H)
f1 (ppm)

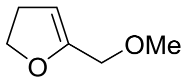

1d

^{1}H-NMR

C_6D_6, 400 MHz, 298 K

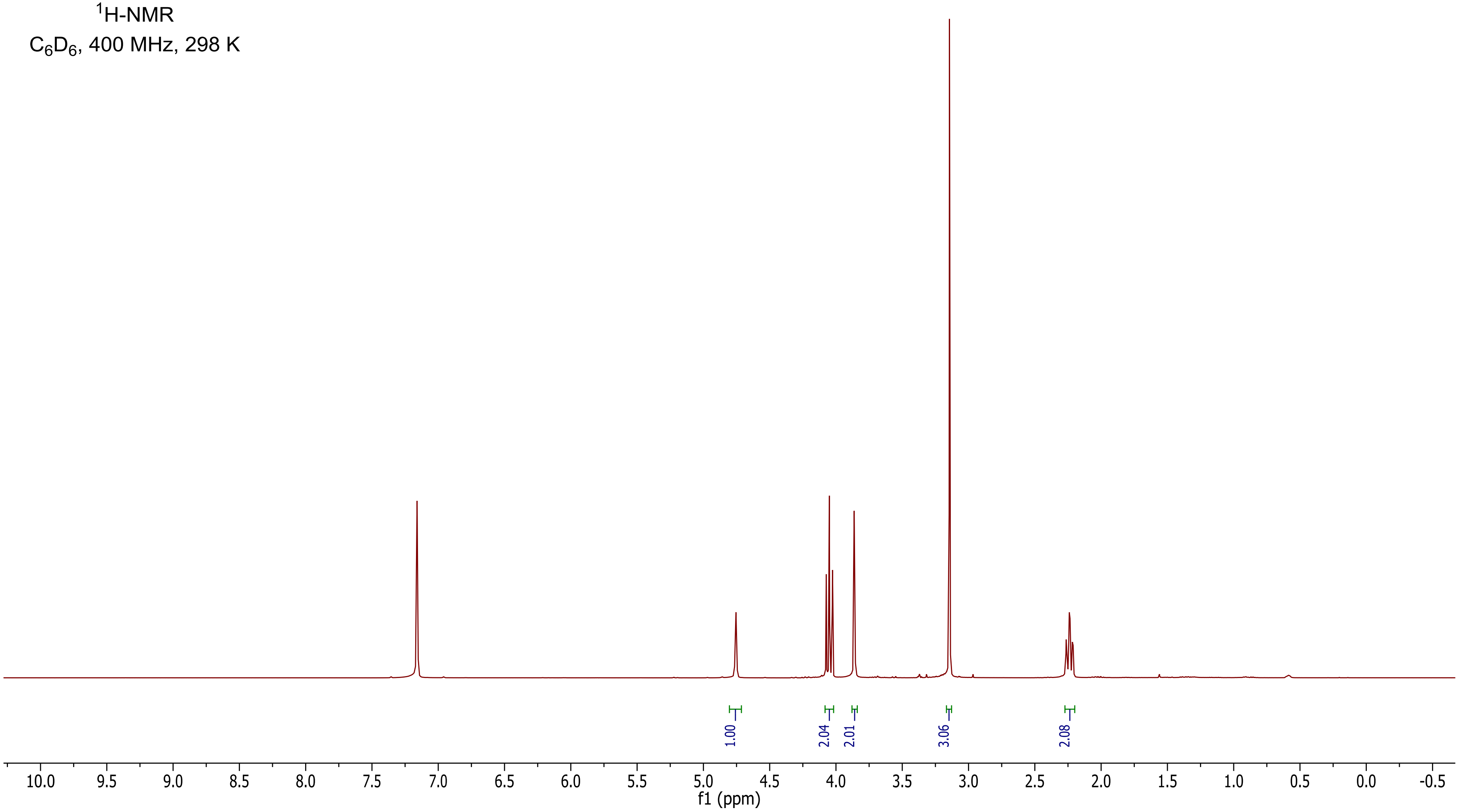

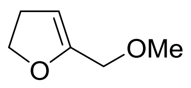

1d

$^{13}C\{^{1}H\}$-NMR

C_6D_6, 100 MHz, 298 K

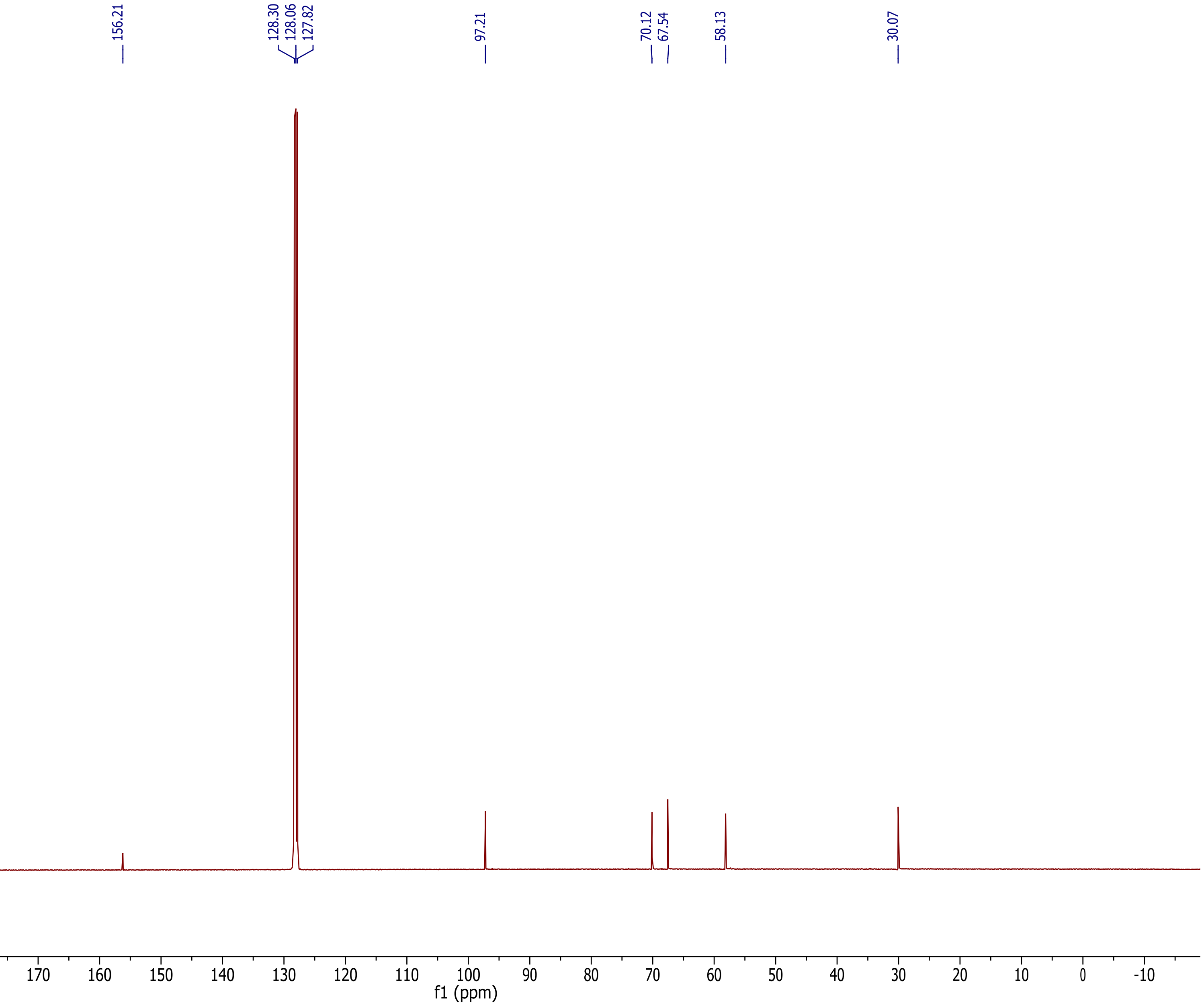

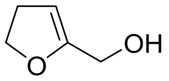

1e

^{1}H-NMR

C_6D_6, 400 MHz, 298 K

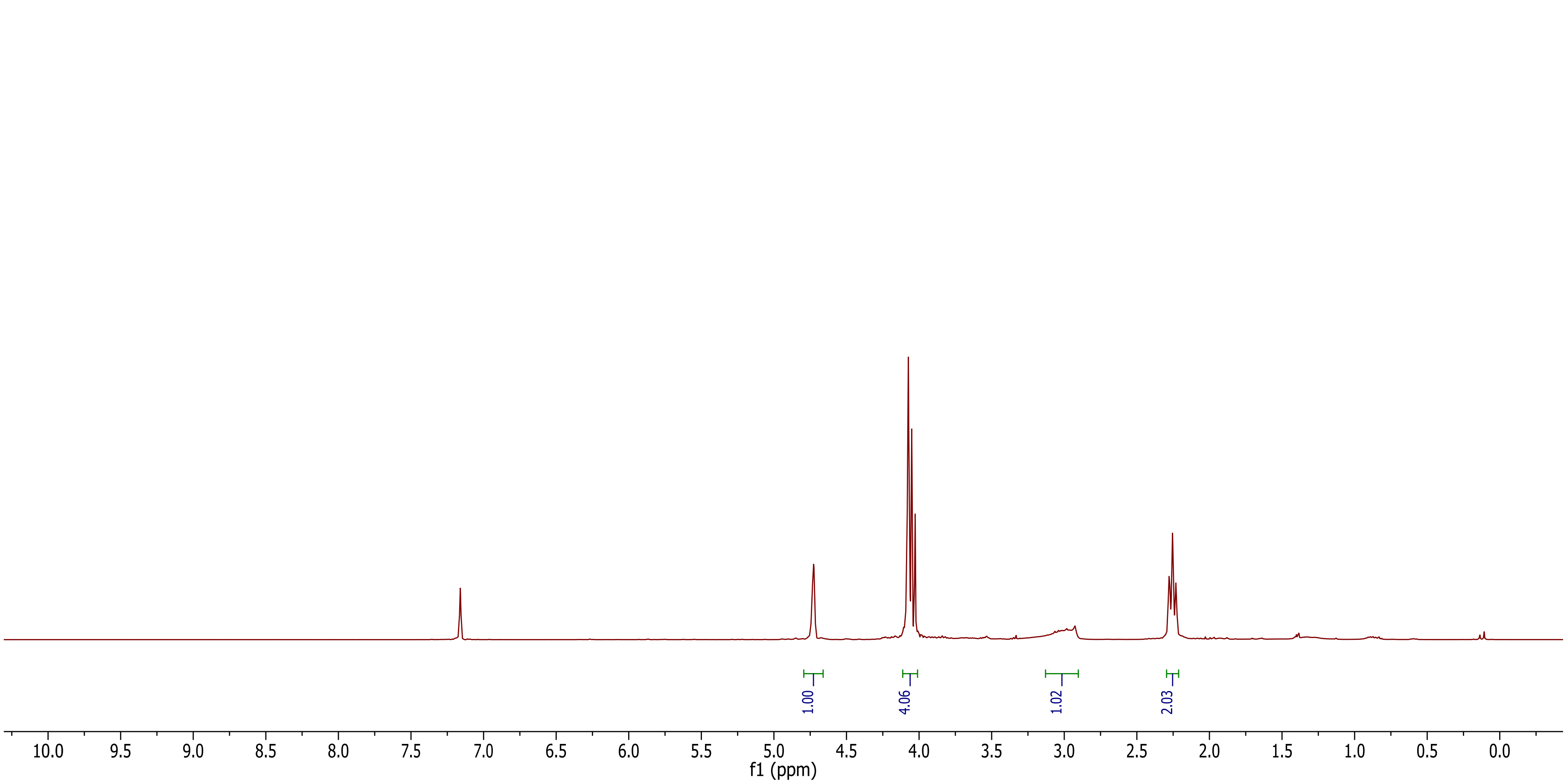

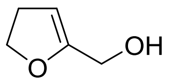

1e

$^{13}C\{^{1}H\}$-NMR

C_6D_6, 100 MHz, 298 K

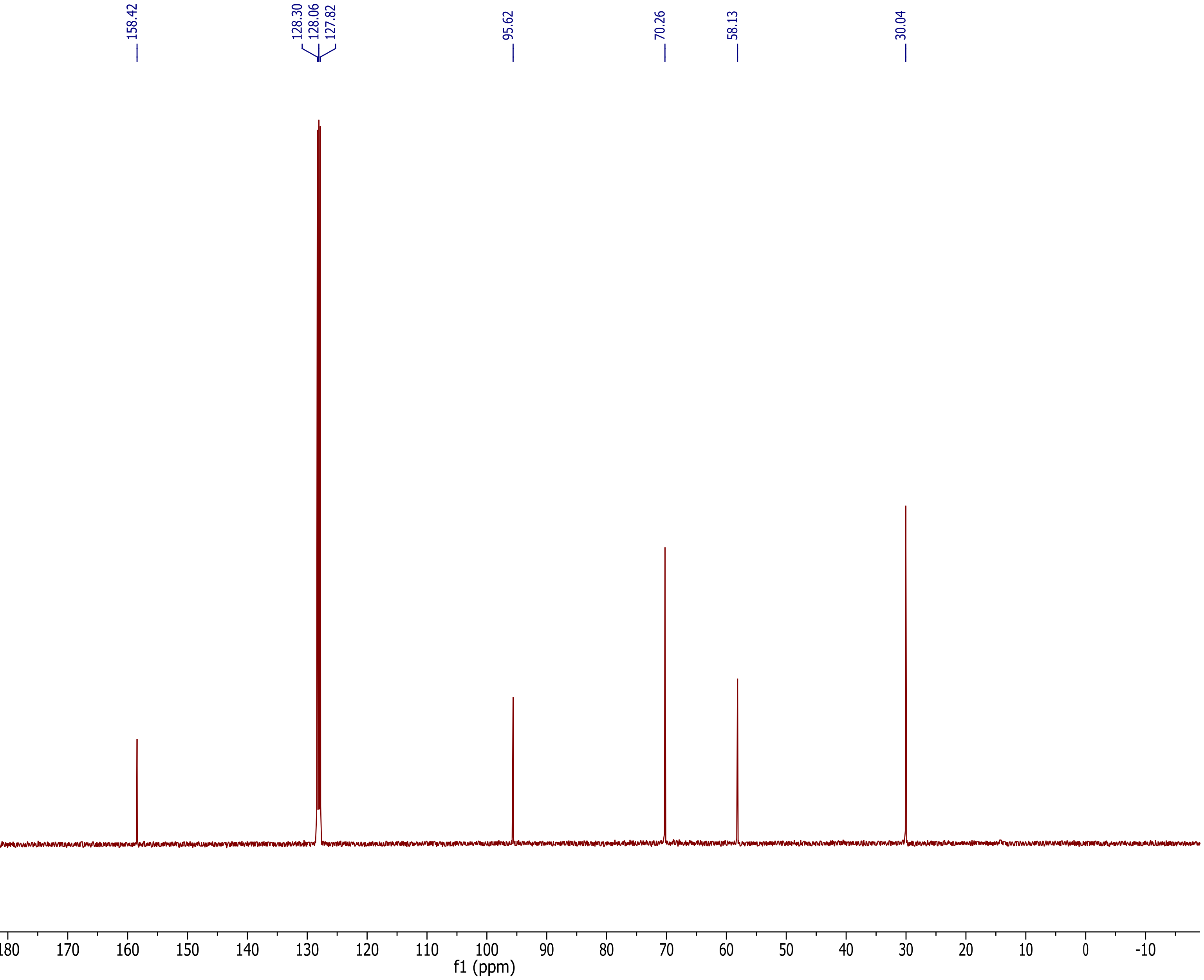

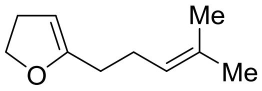

1f

^{1}H-NMR

C_6D_6, 400 MHz, 298 K

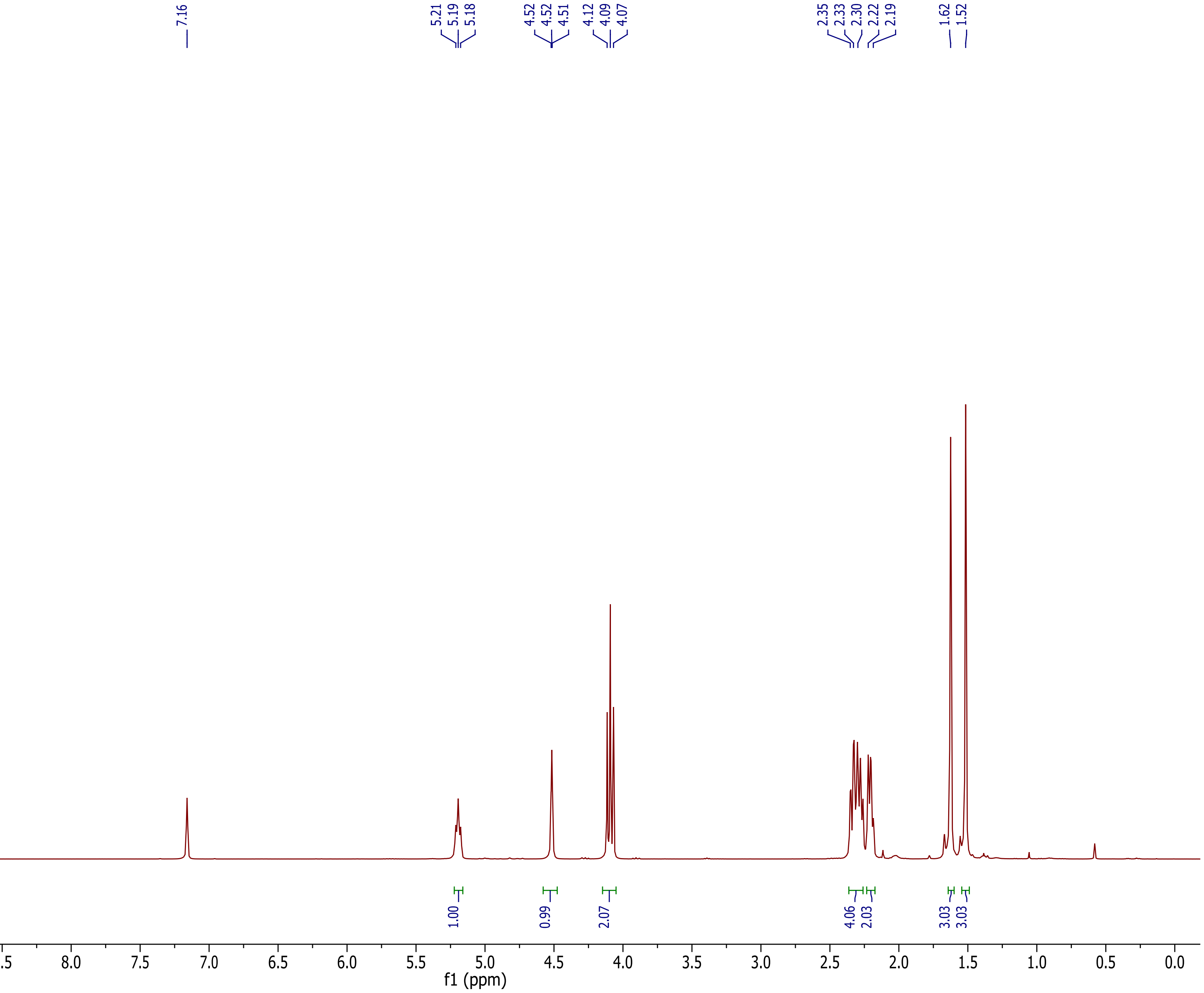

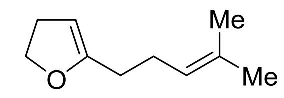

1f

$^{13}C\{^1H\}$-NMR

C_6D_6, 100 MHz, 298 K

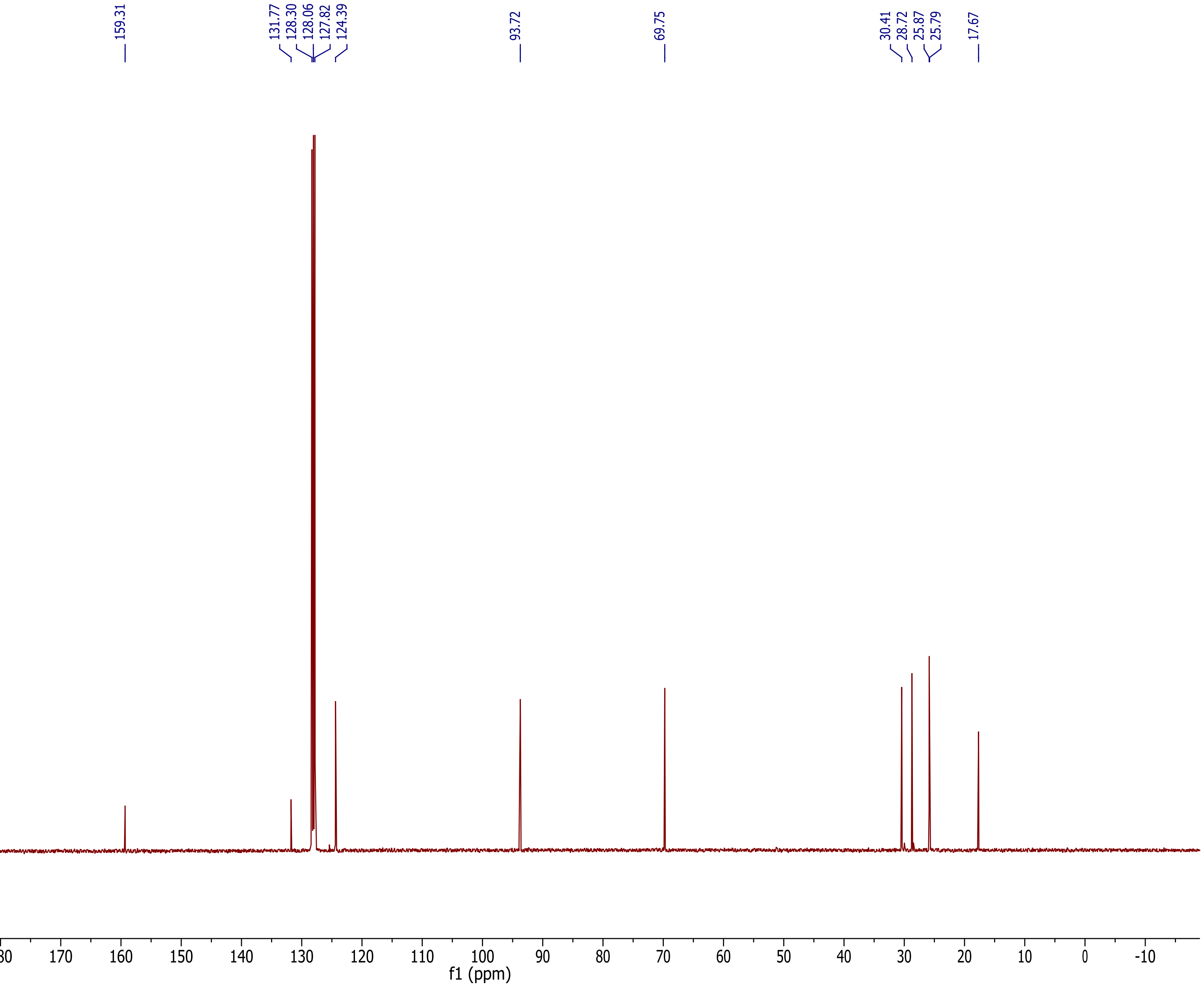

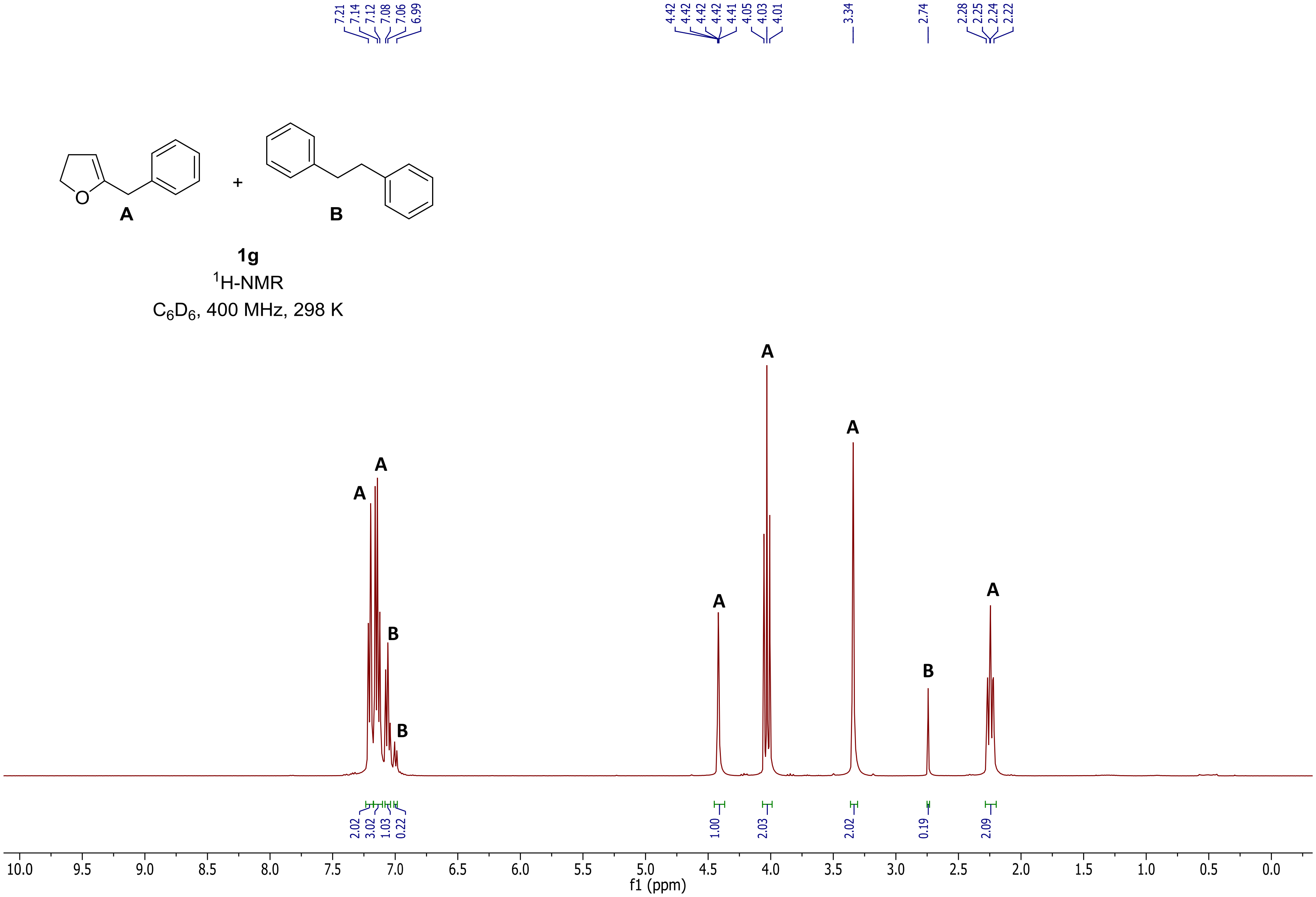
7.21
7.14
7.12
7.08
7.06
6.99
4.42
4.42
4.42
4.42
4.41
4.05
4.03
4.01
3.34
2.74
2.28
2.25
2.24
2.22
A
+
B
1g
1H-NMR
C6D6, 400 MHz, 298 K
A
A
A
B
B
A
A
B
A
2.02
3.02
1.03
0.22
1.00
2.03
2.02
0.19
2.09
10.0
9.5
9.0
8.5
8.0
7.5
7.0
6.5
6.0
5.5
5.0
4.5
4.0
3.5
3.0
2.5
2.0
1.5
1.0
0.5
0.0
f1 (ppm)

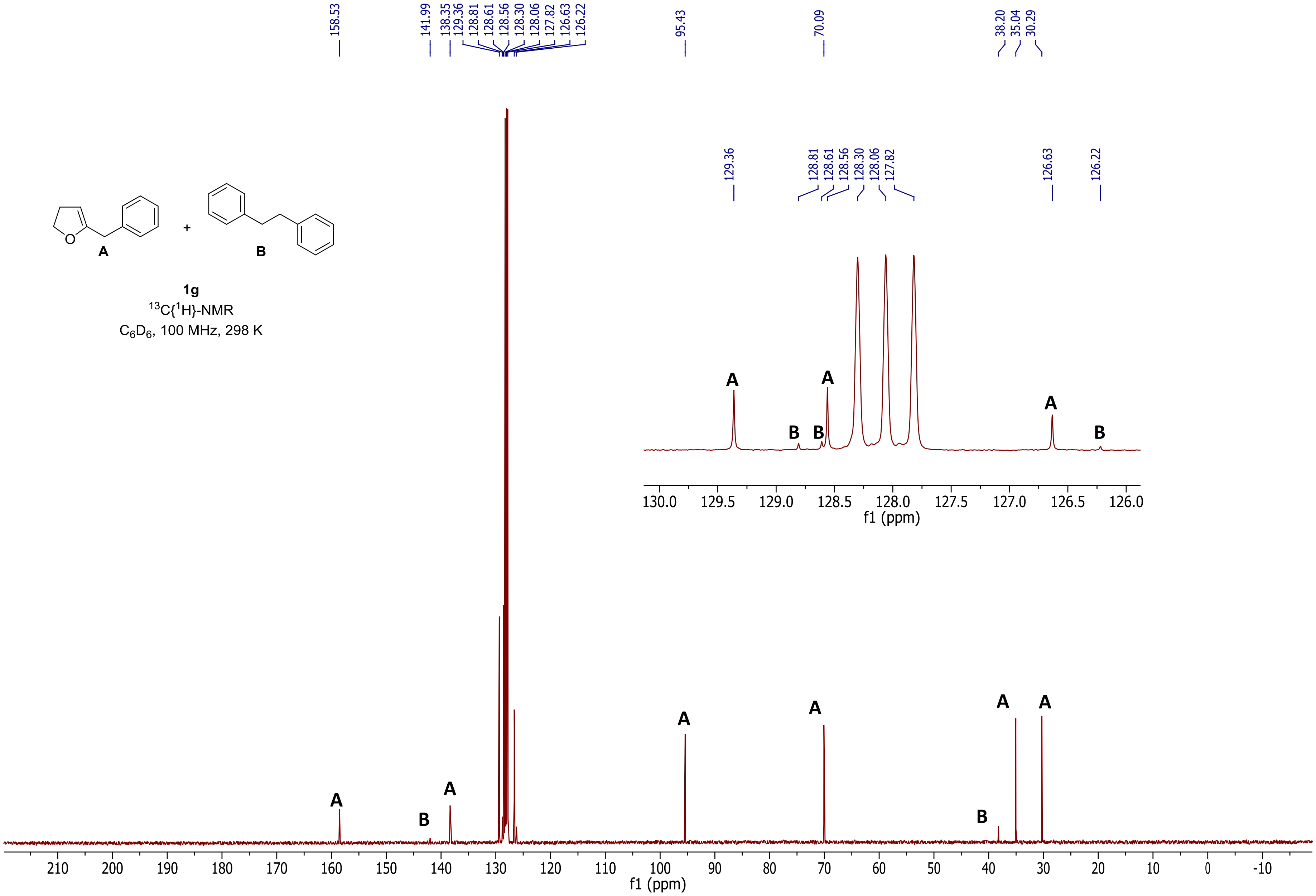

$^{13}C\{^{1}H\}$-NMR

C_6D_6, 100 MHz, 298 K

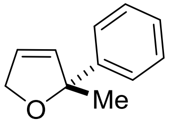

3aa

^{1}H-NMR

C_6D_6, 400 MHz, 298 K

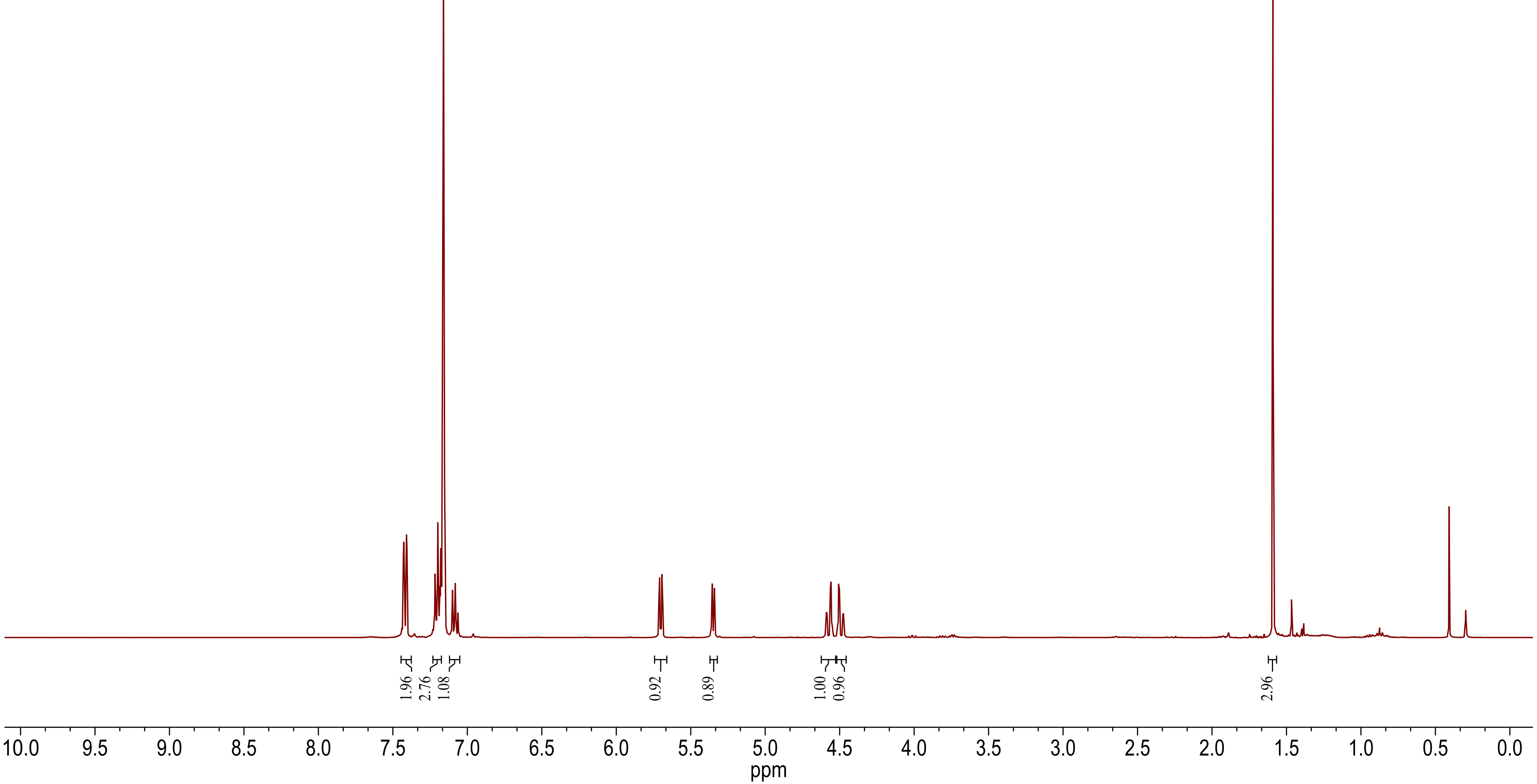

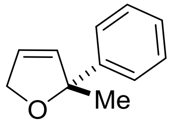

3aa
{^{13}C}^{1}H-NMR
C_6D_6, 125 MHz, 298 K

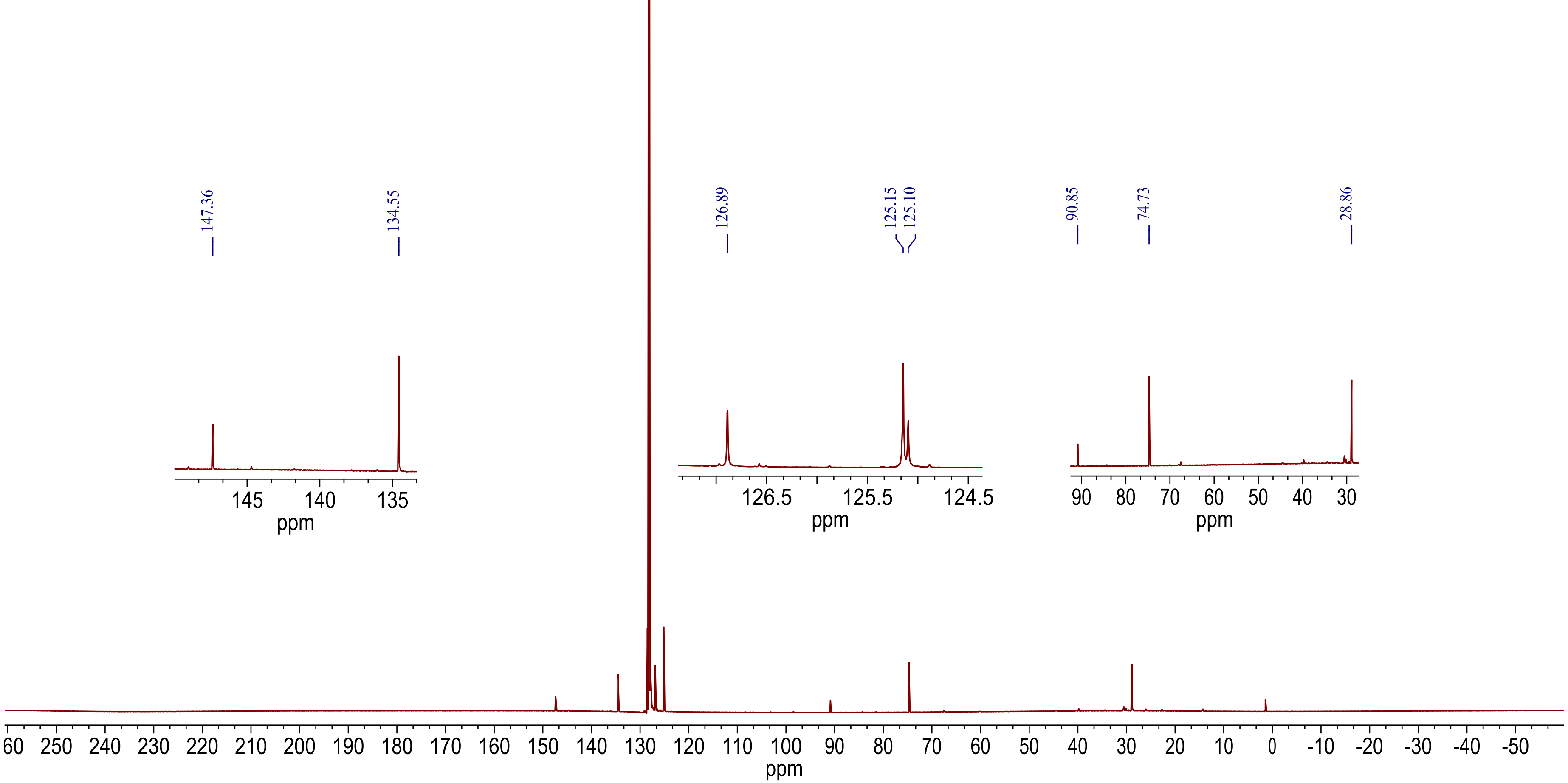

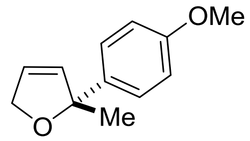

3ab
^{1}H-NMR
C_6D_6, 400 MHz, 298 K

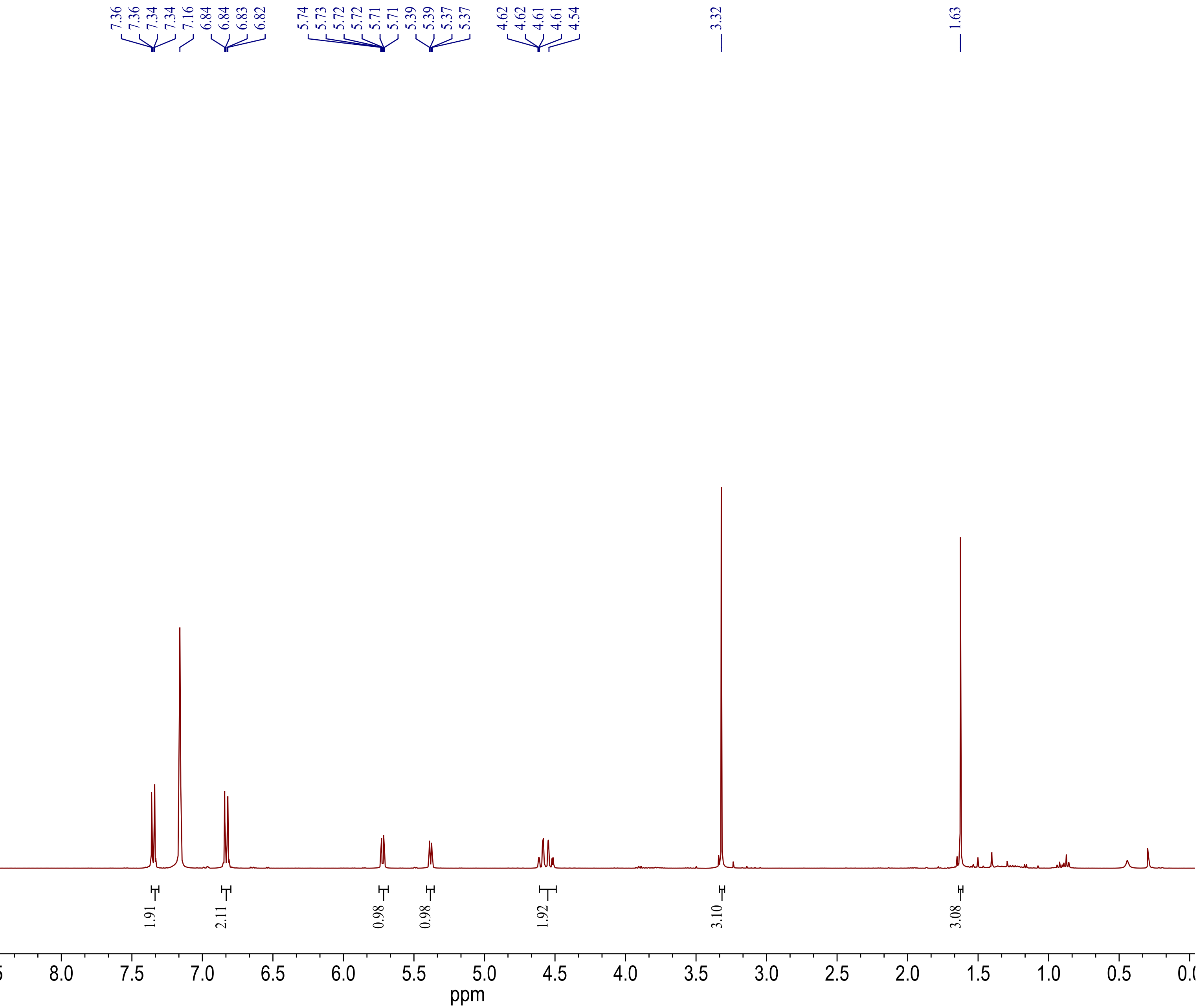

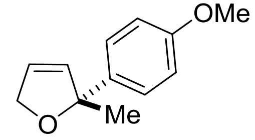

3ab

$^{13}C\{^{1}H\}$-NMR

C_6D_6, 100 MHz, 298 K

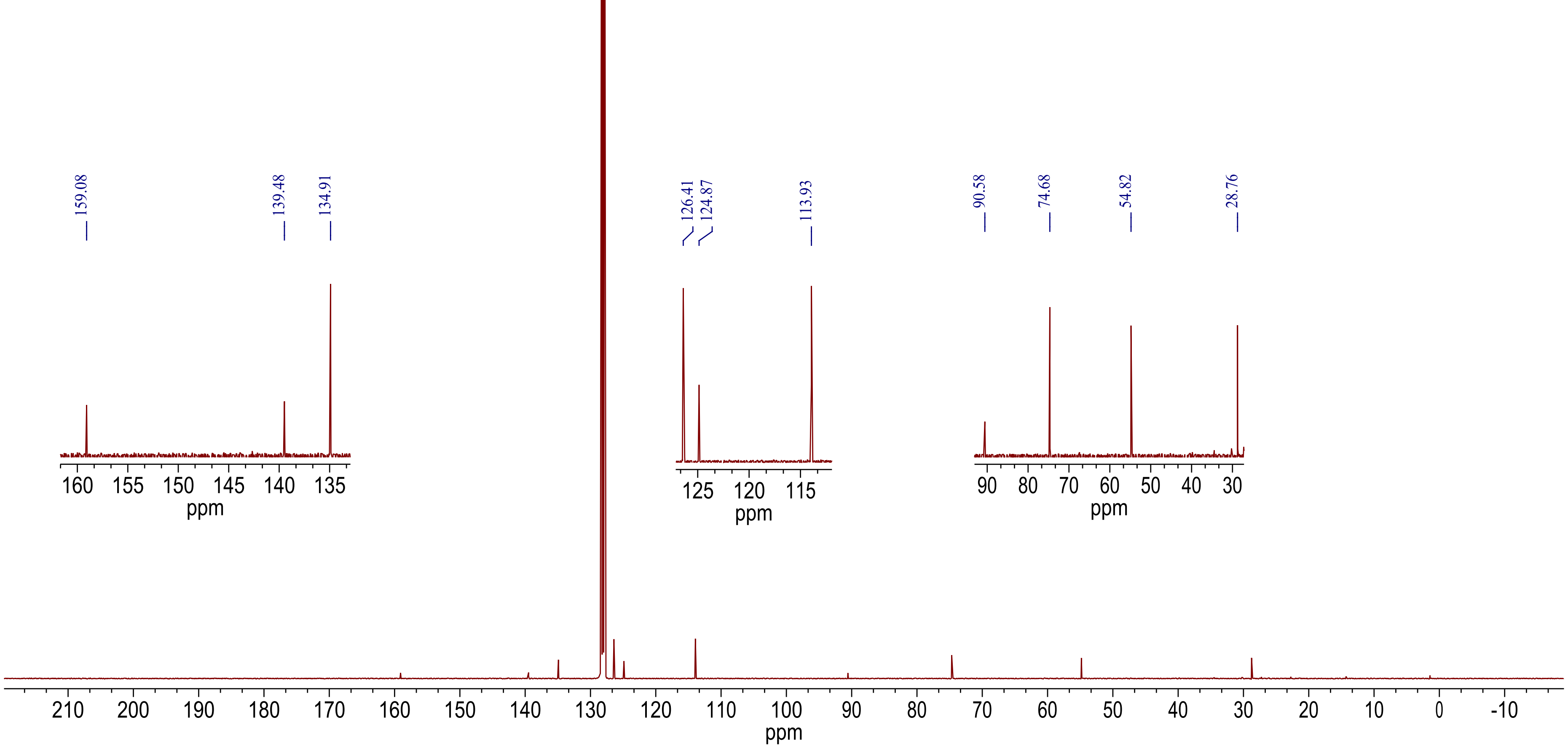

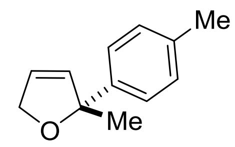

3ad
^{1}H-NMR
C_6D_6, 400 MHz, 298 K

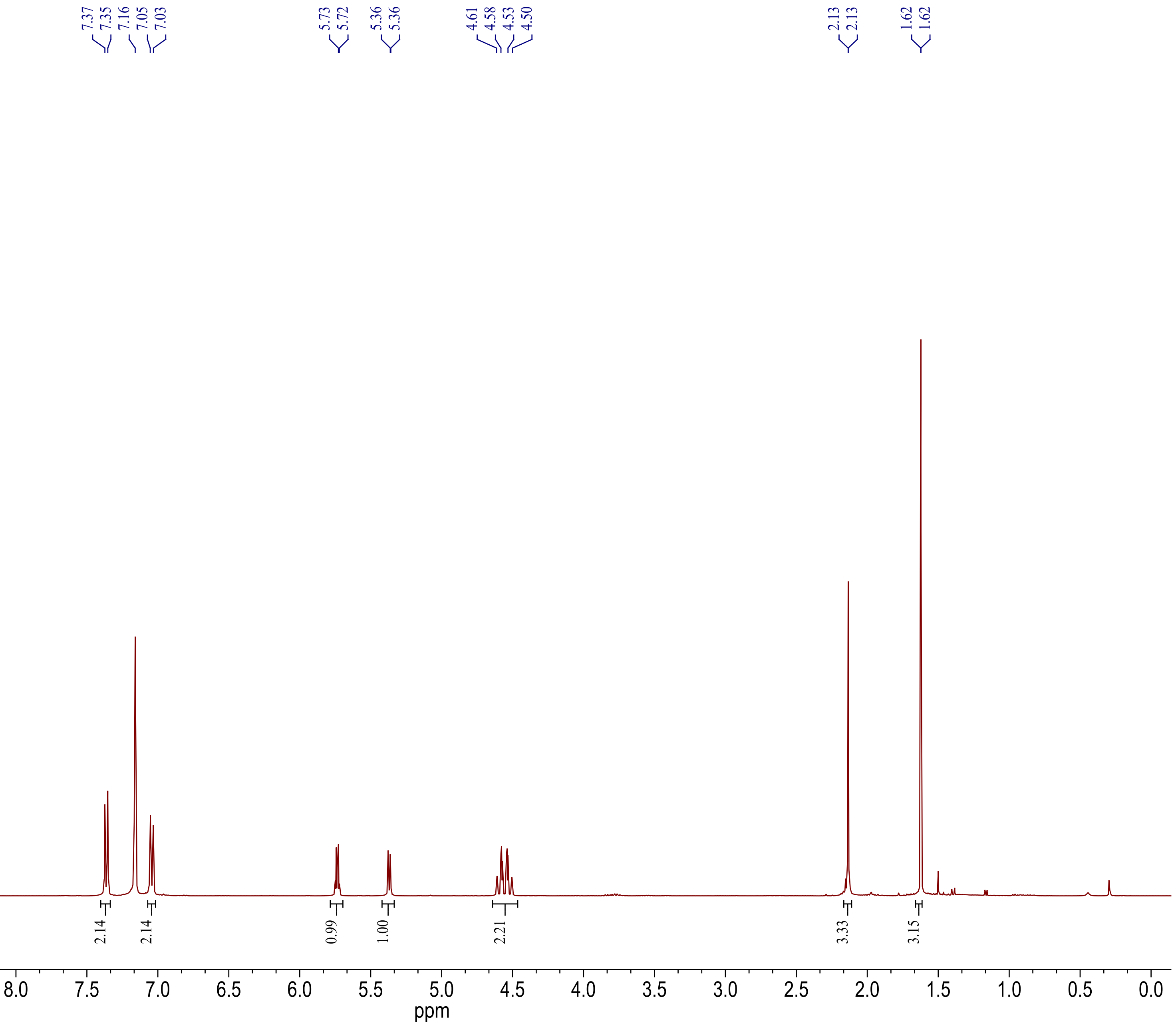

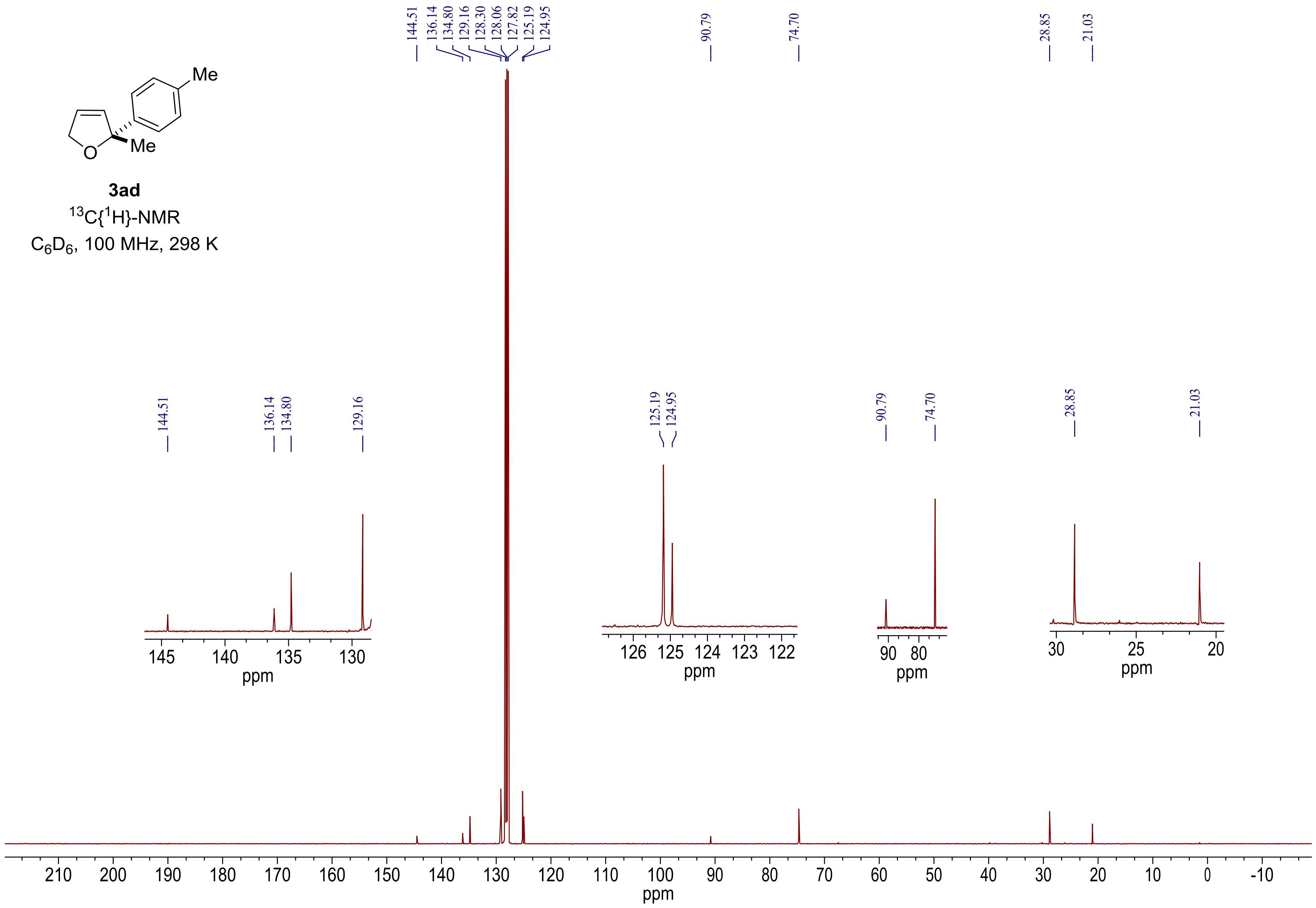

3ad
$^{13}C\{^{1}H\}$-NMR
C_6D_6, 100 MHz, 298 K

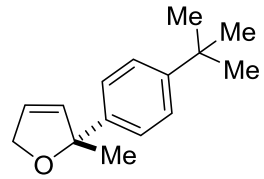

3ae

^{1}H-NMR

C_6D_6, 400 MHz, 298 K

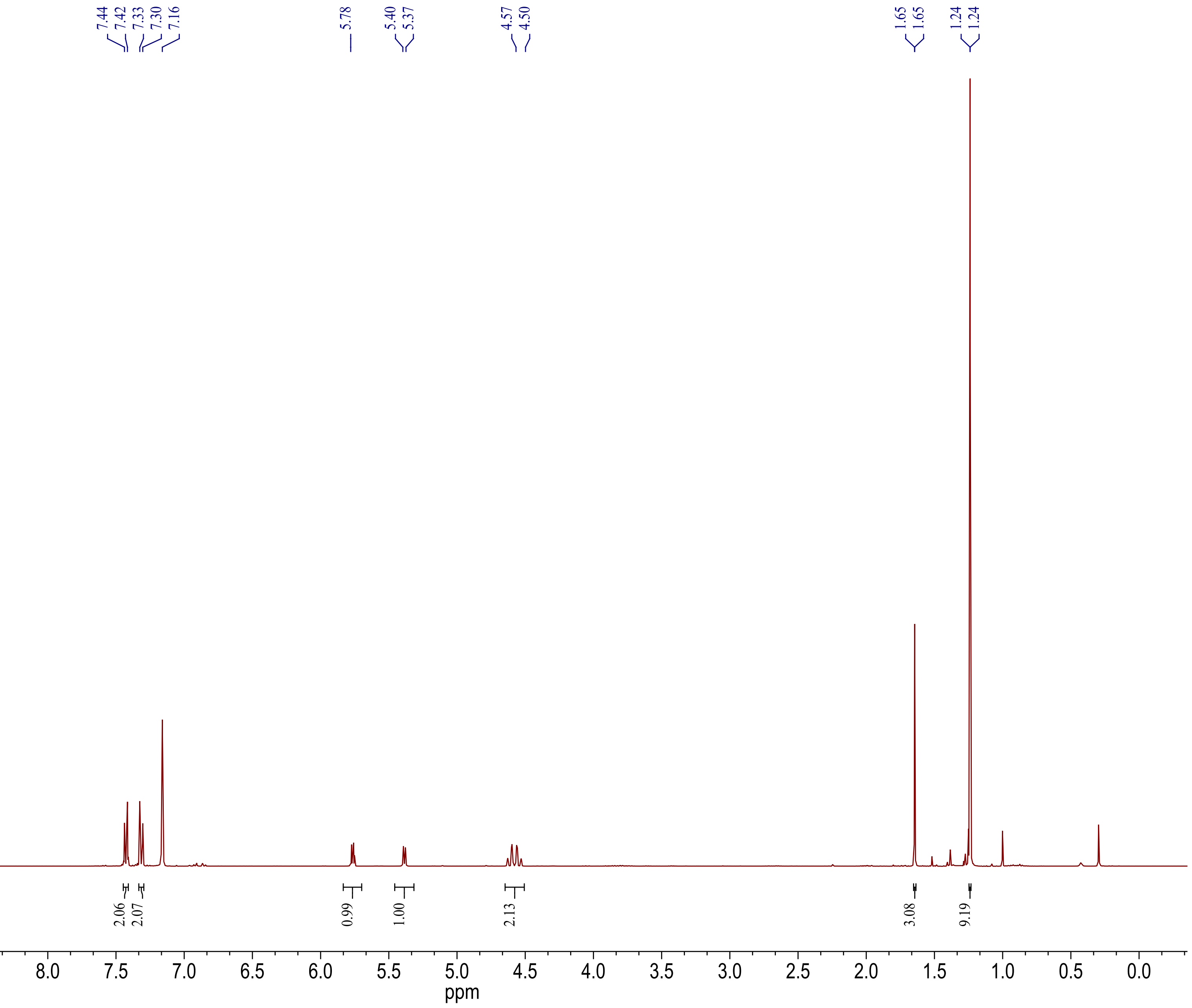

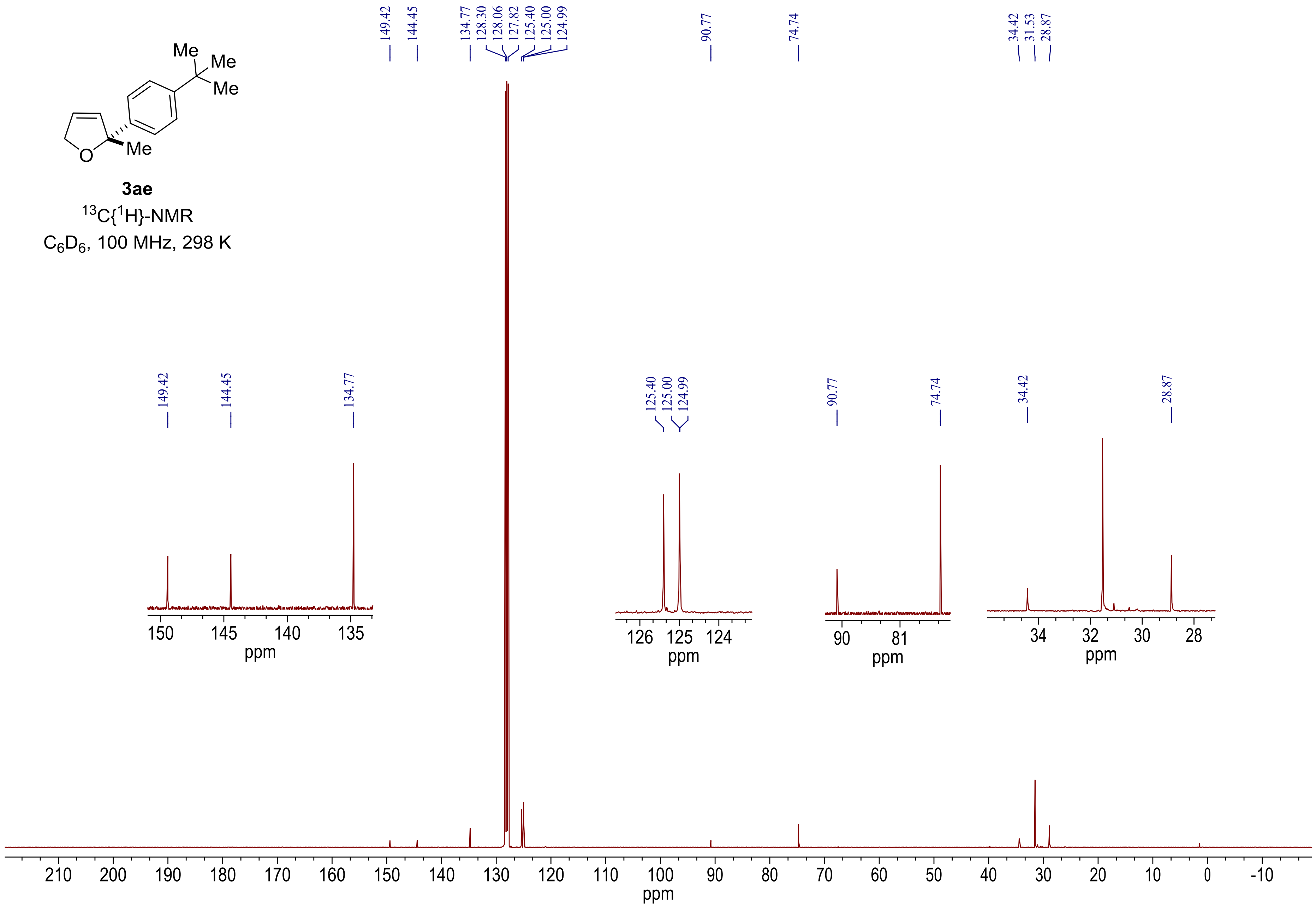

Me
Me
Me
O
Me
3ae
$^{13}C\{^{1}H\}$-NMR
C_6D_6, 100 MHz, 298 K
149.42
144.45
134.77
128.30
128.06
127.82
125.40
125.00
124.99
90.77
74.74
34.42
31.53
28.87
ppm

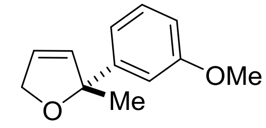

3af
^{1}H-NMR
C_6D_6, 400 MHz, 298 K

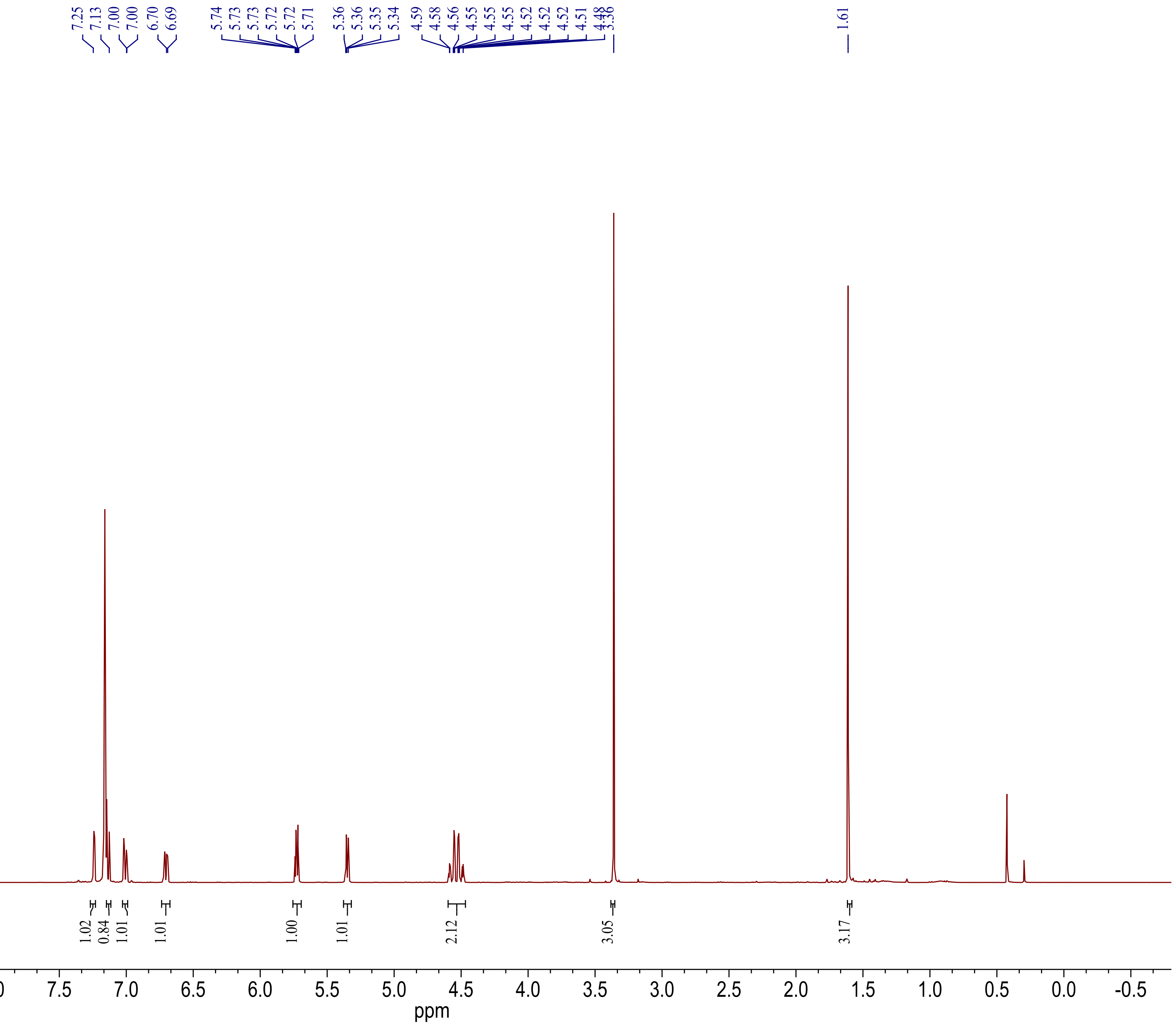

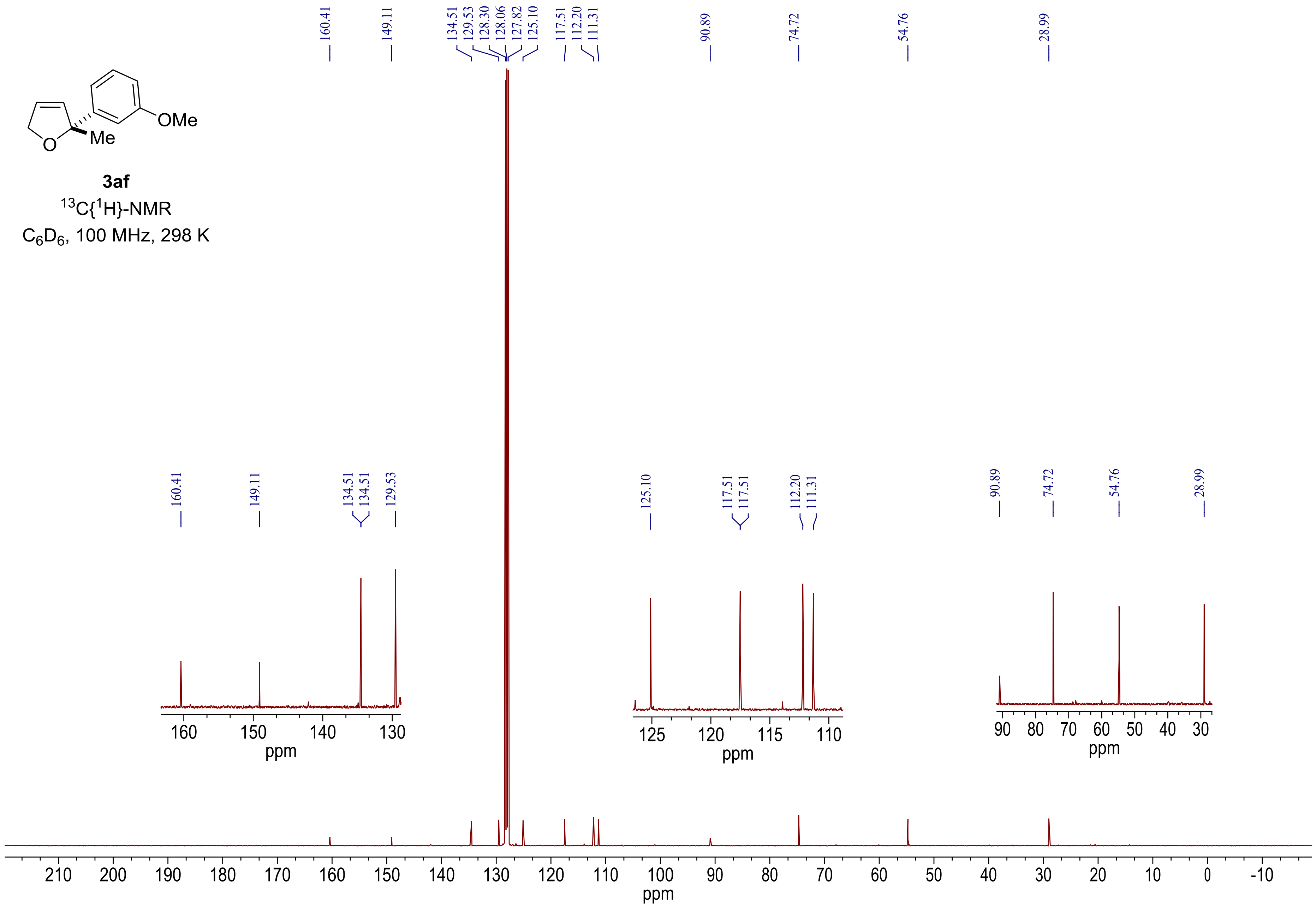
OMe
Me
O
3af
$^{13}C\{^{1}H\}$-NMR
C_6D_6, 100 MHz, 298 K
160.41
149.11
134.51
129.53
128.30
128.06
127.82
125.10
117.51
112.20
111.31
90.89
74.72
54.76
28.99
ppm

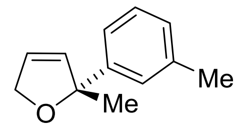

3ag

^{1}H-NMR

C_6D_6, 400 MHz, 298 K

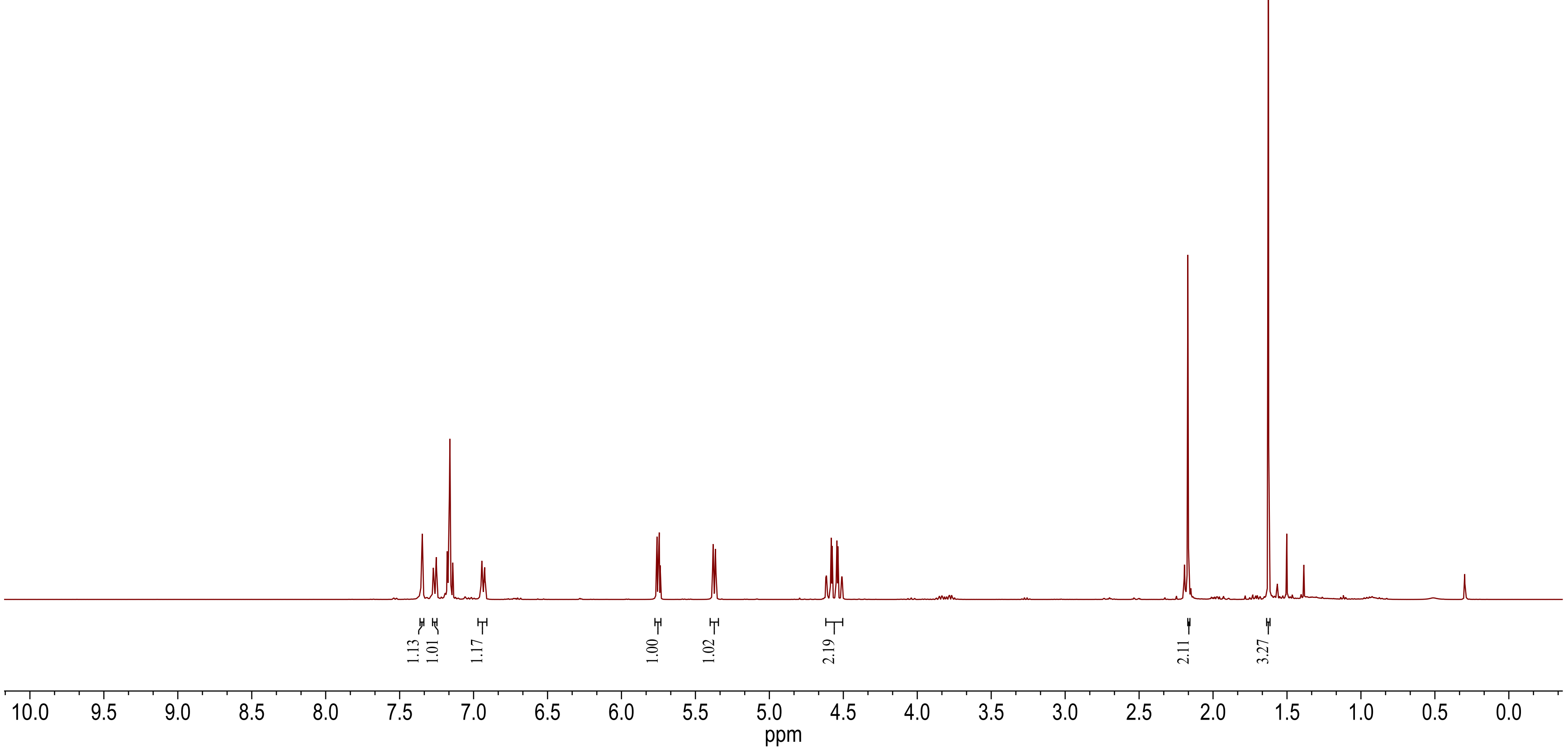

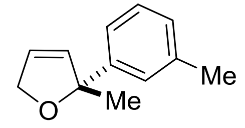

3ag

$^{13}C\{^{1}H\}$-NMR

C_6D_6, 100 MHz, 298 K

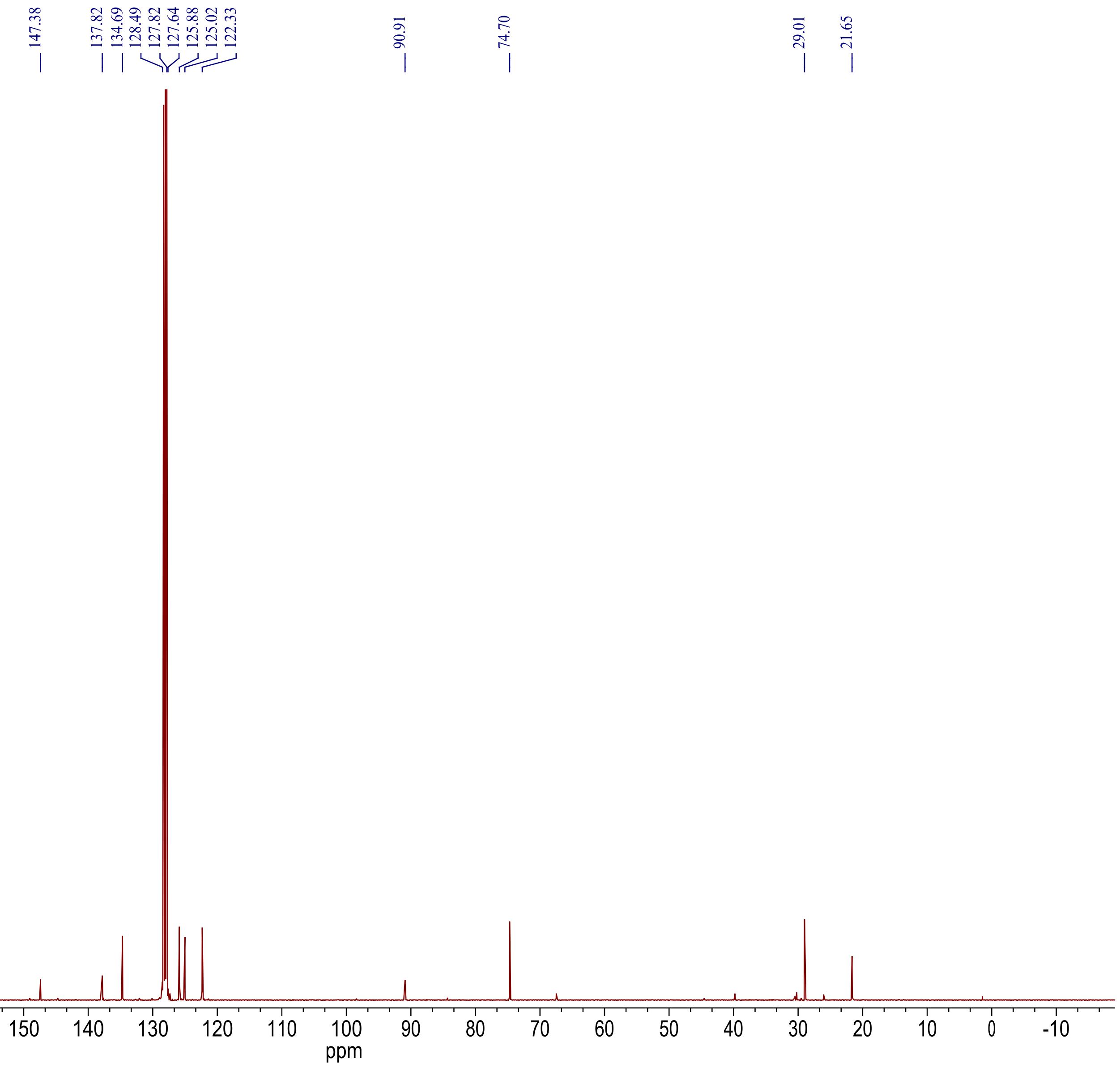

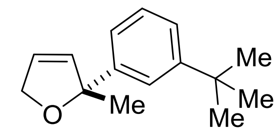

3ah

^{1}H-NMR

C_6D_6, 400 MHz, 298 K

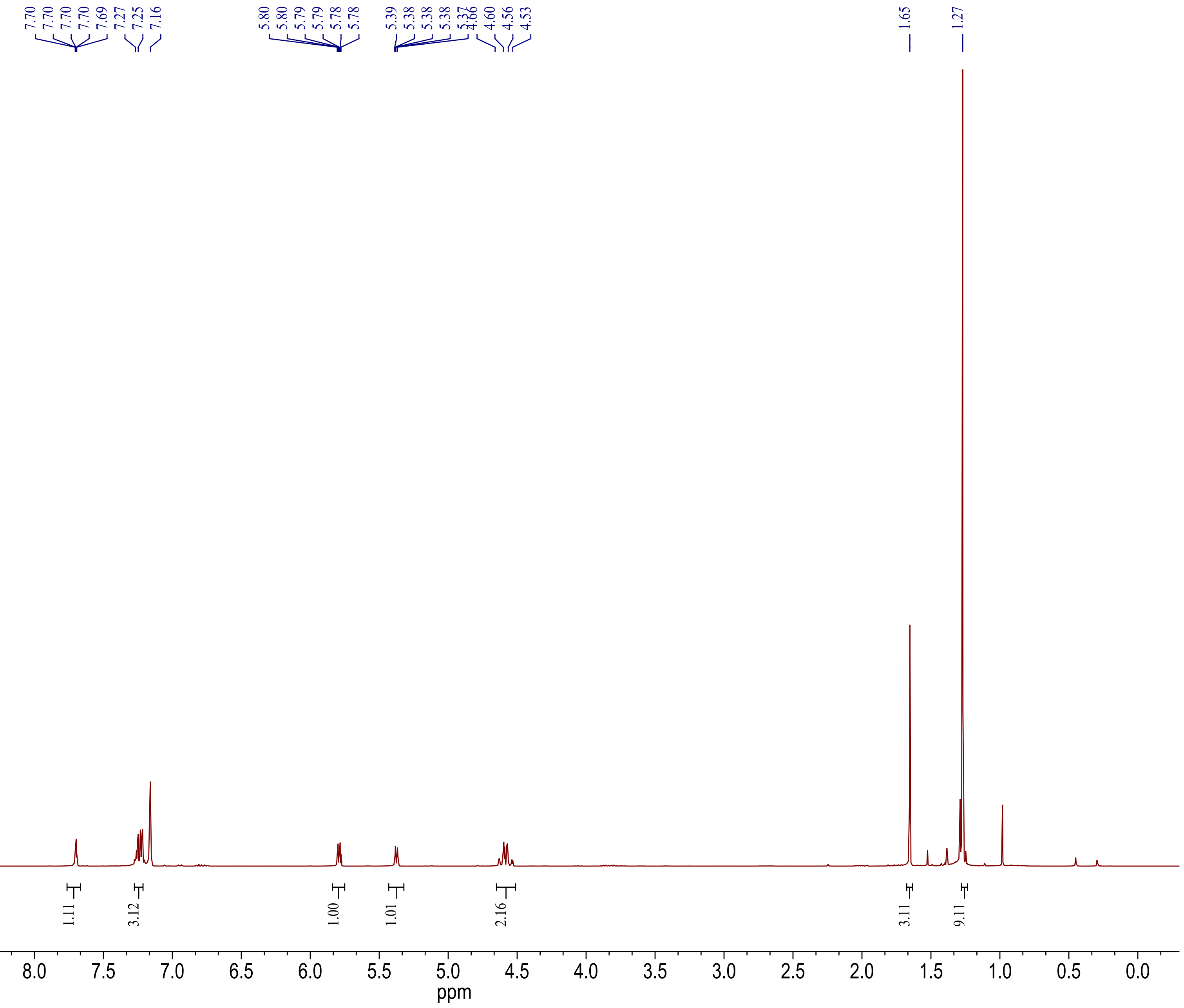

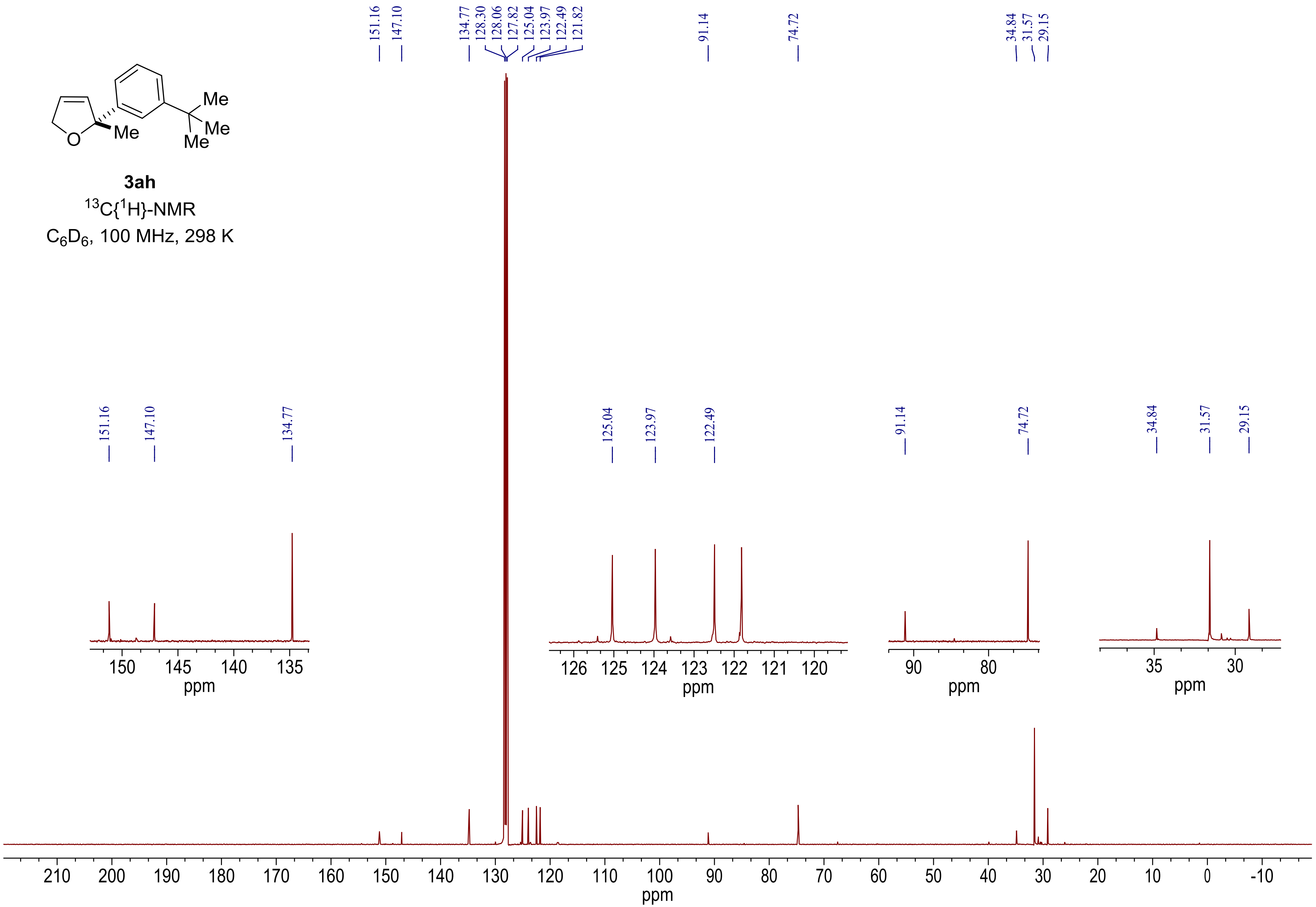

3ah

$^{13}C\{^{1}H\}$-NMR

C_6D_6, 100 MHz, 298 K

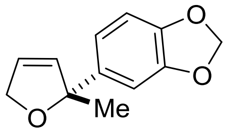

3ai

^{1}H-NMR

C_6D_6, 400 MHz, 298 K

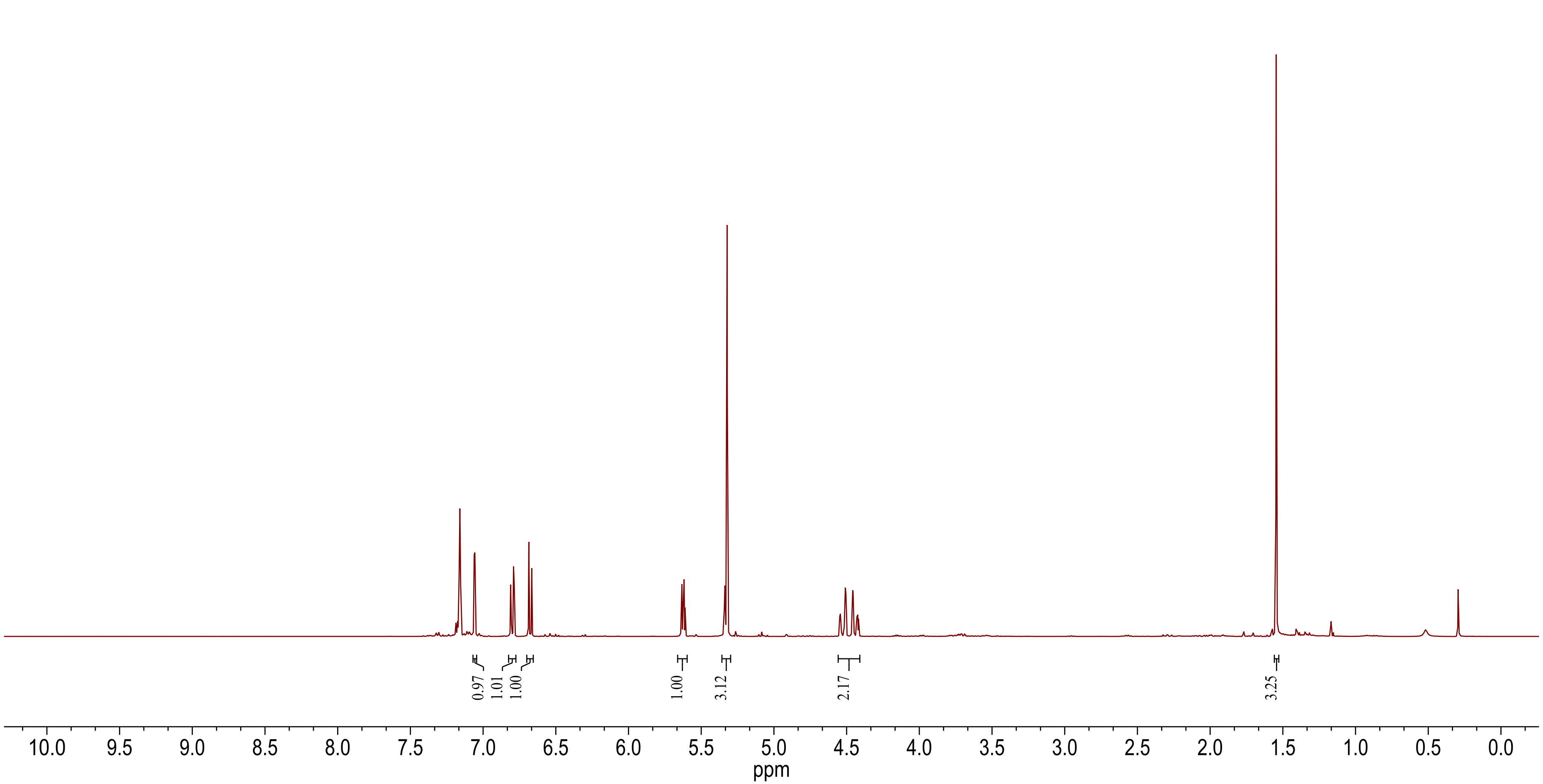

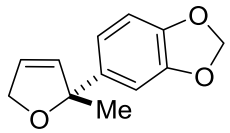

3ai

$^{13}C\{^1H\}$-NMR

C_6D_6, 100 MHz, 298 K

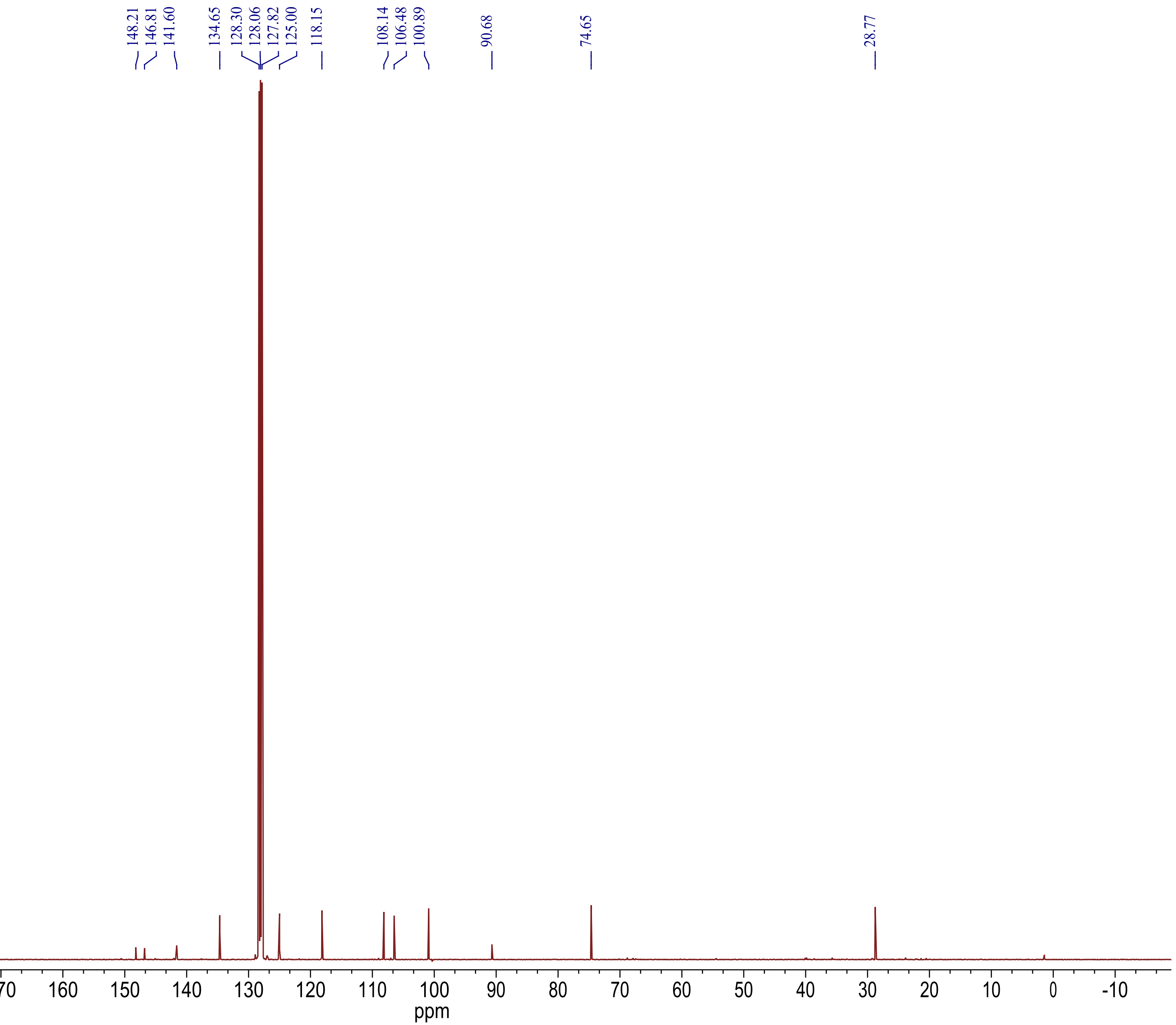

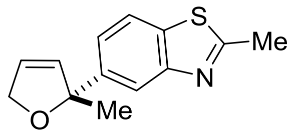

3aj
^{1}H-NMR
C_6D_6, 400 MHz, 298 K

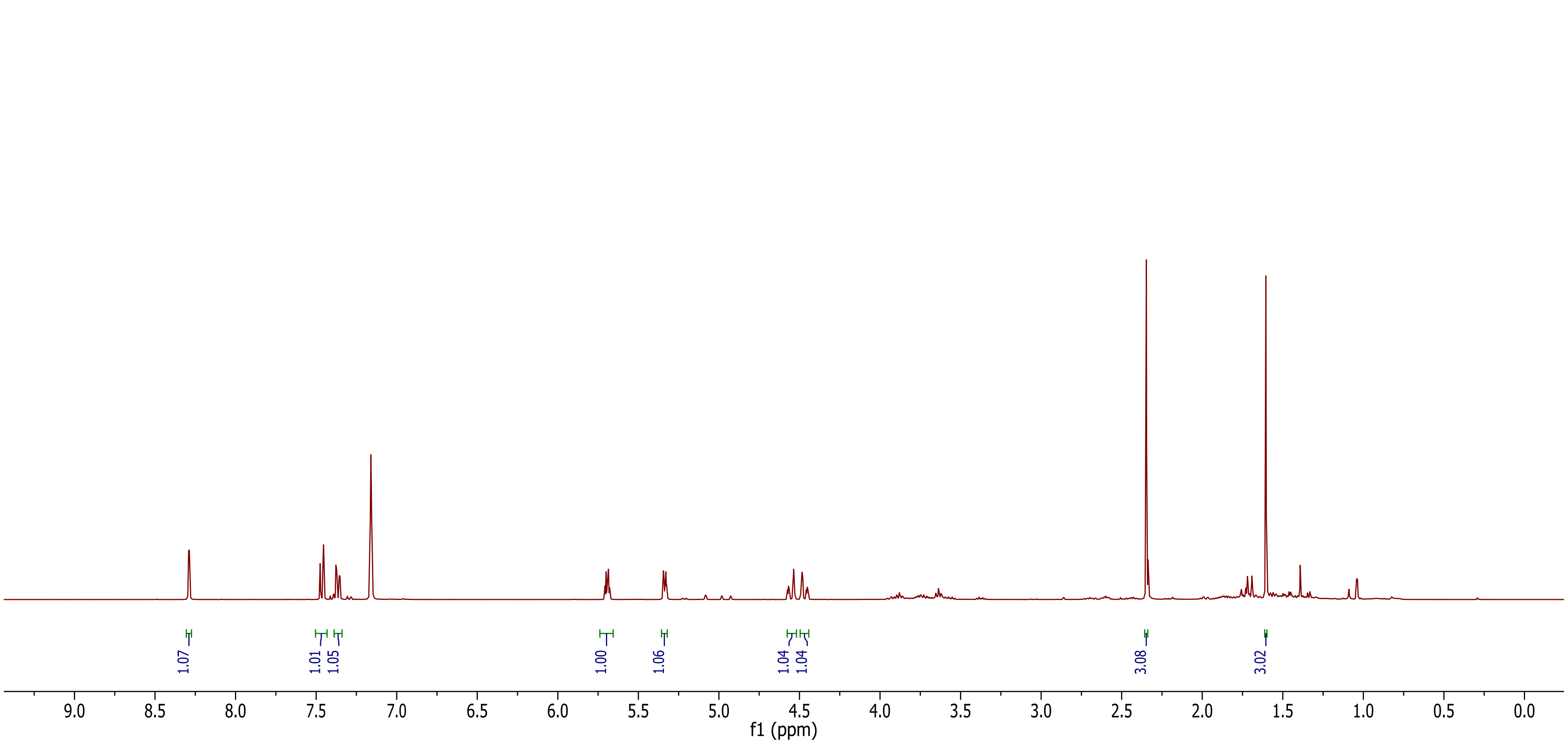

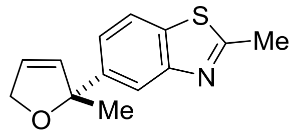

3aj
$^{13}C\{^1H\}$-NMR
C_6D_6, 100 MHz, 298 K

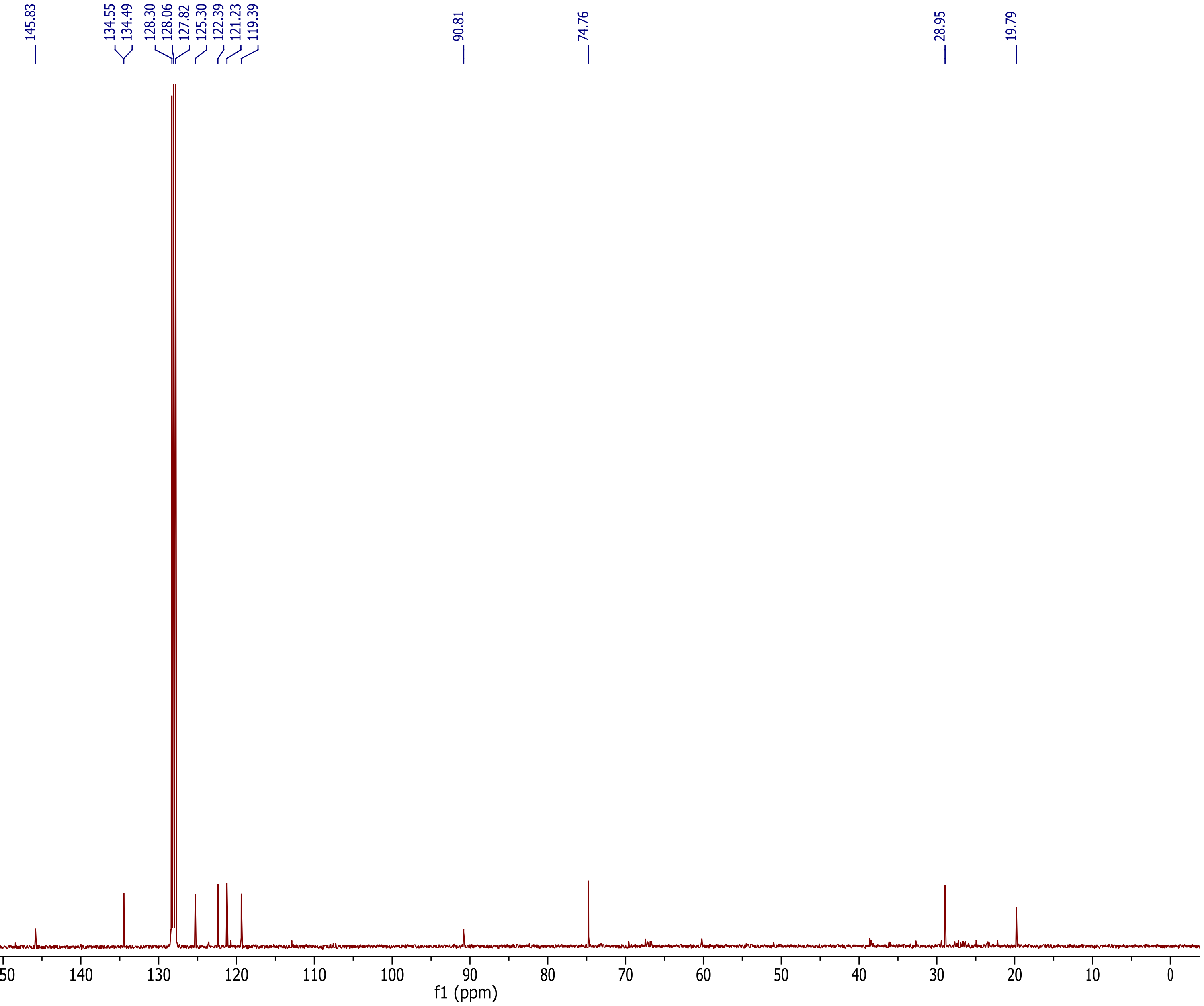

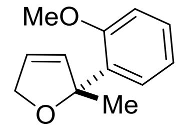

3ak

^{1}H-NMR

C_6D_6, 500 MHz, 298 K

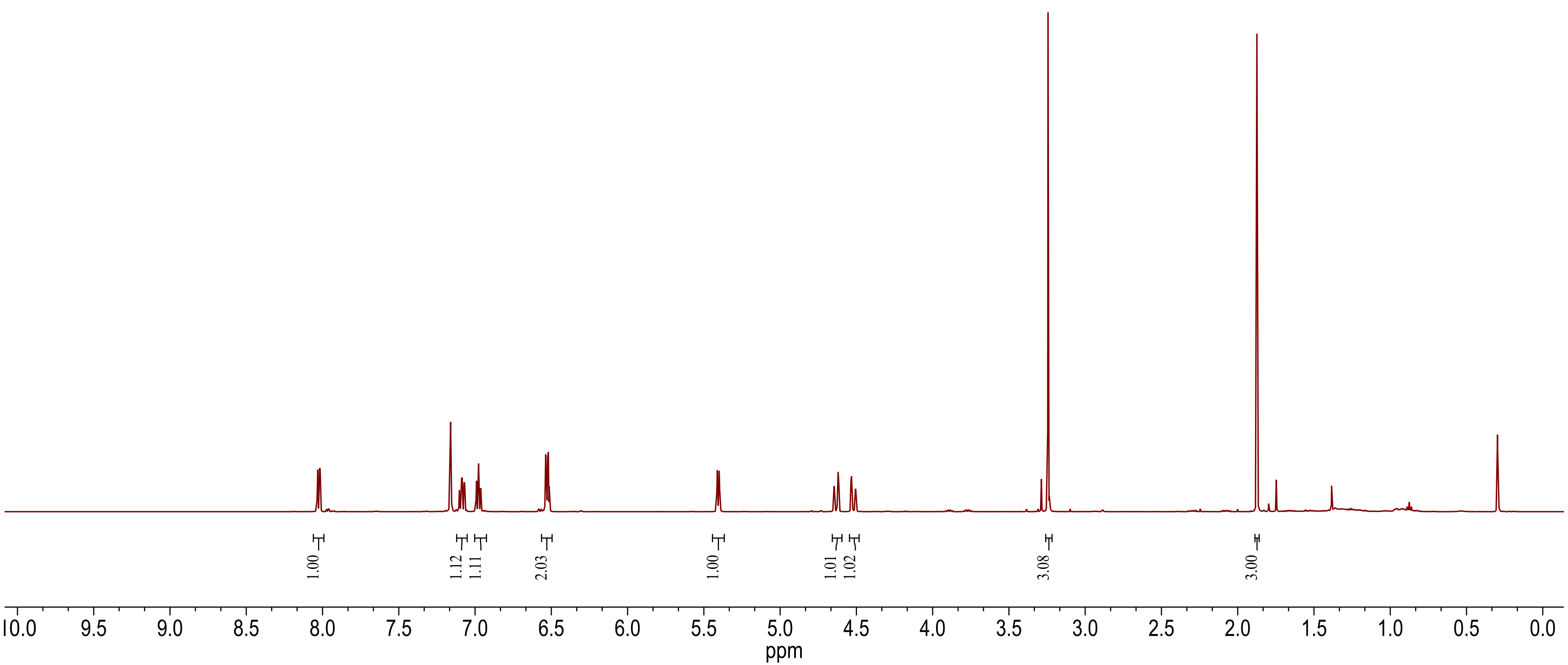

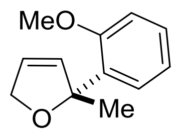

3ak
{^{13}C}^{1}H-NMR
C_6D_6, 125 MHz, 298 K

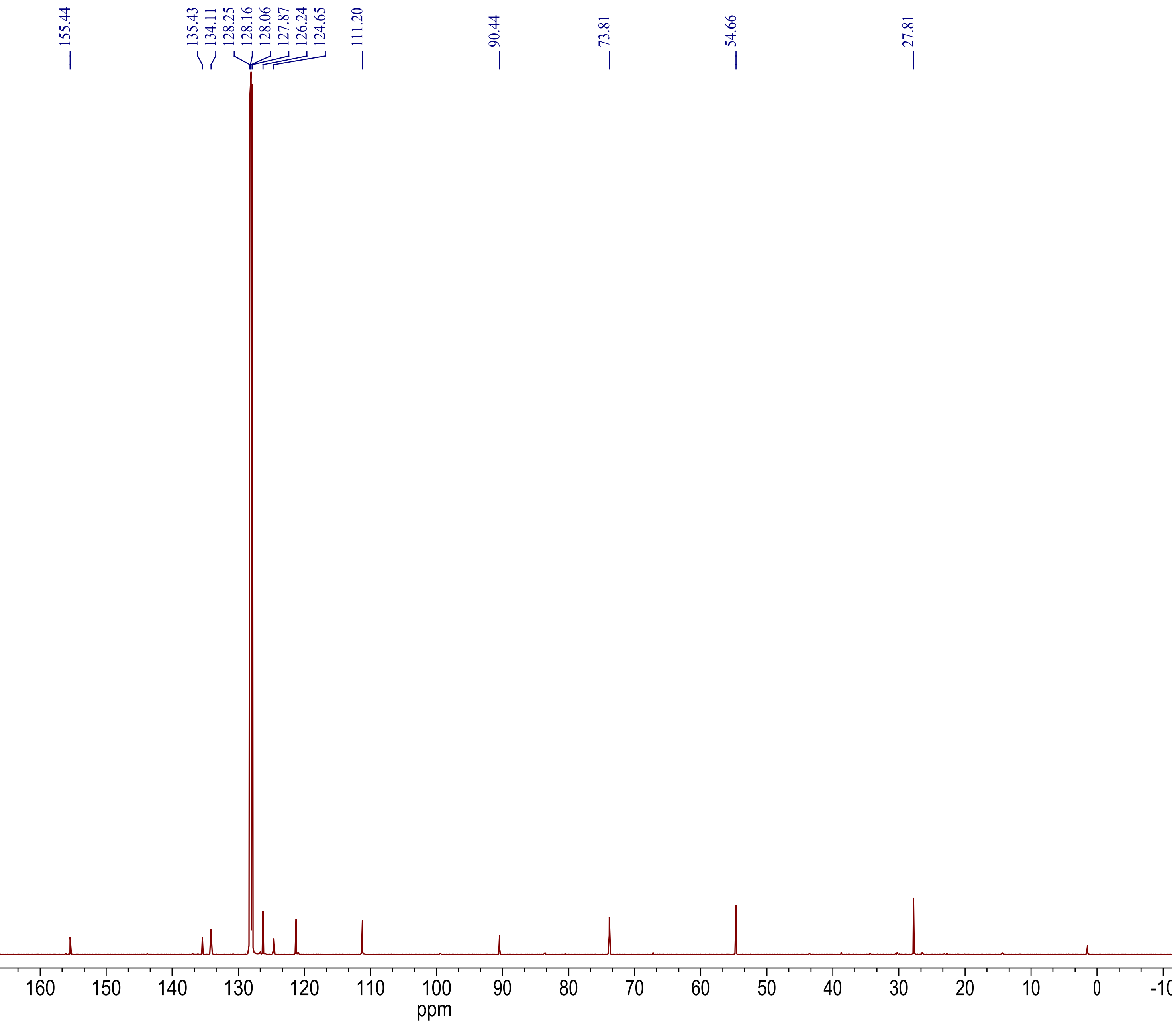

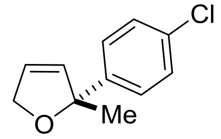

3al

^{1}H-NMR

C_6D_6, 400 MHz, 298 K

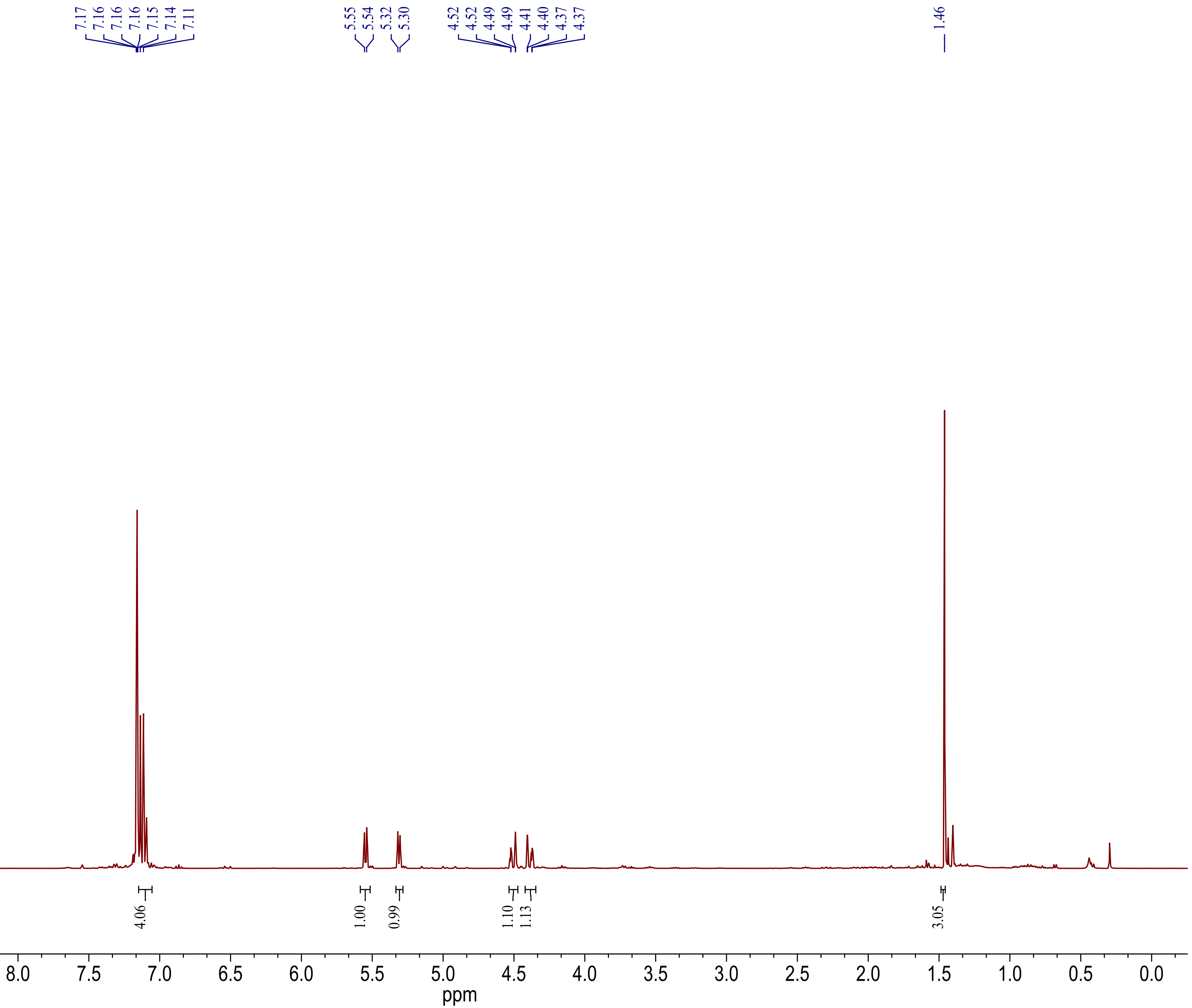

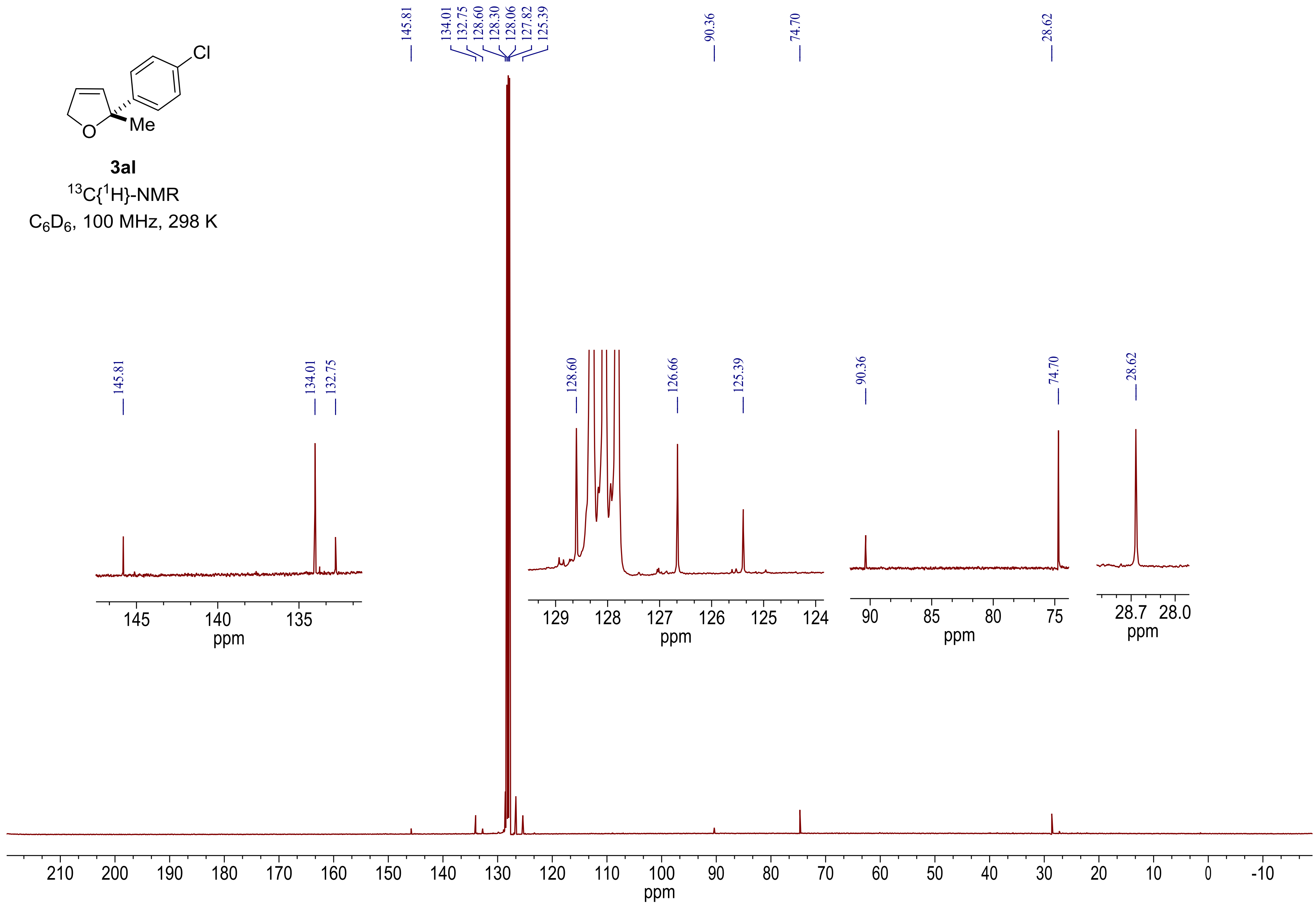

Cl
Me
O
3al
$^{13}C\{^{1}H\}$-NMR
C_6D_6, 100 MHz, 298 K
145.81
134.01
132.75
128.60
128.30
128.06
127.82
125.39
90.36
74.70
28.62
126.66
ppm

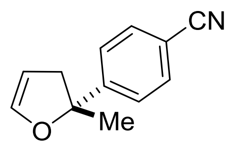

4ac
^{1}H-NMR
C_6D_6, 400 MHz, 298 K

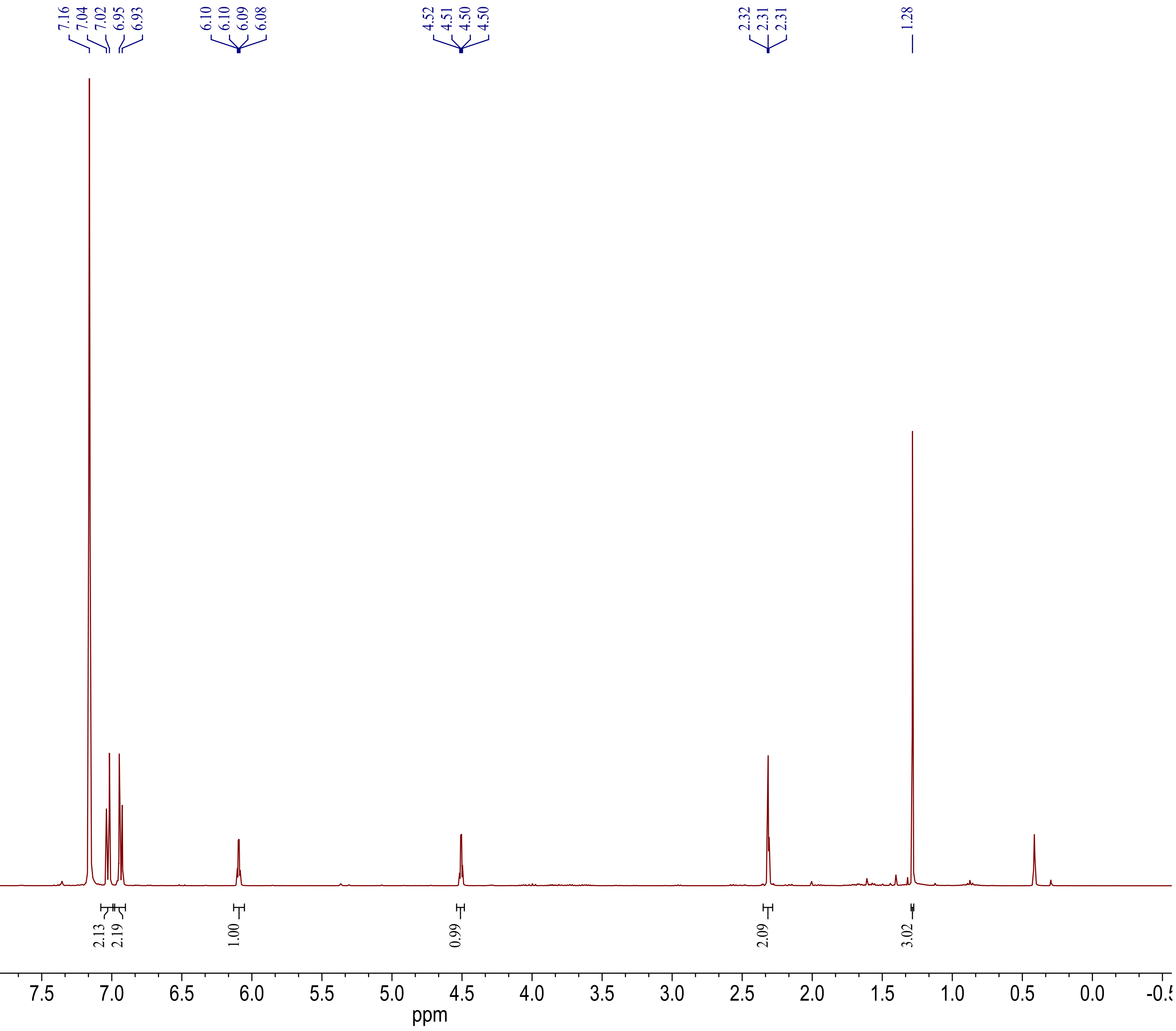

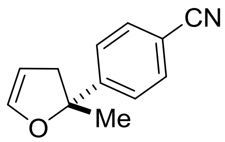

4ac
$^{13}C\{^1H\}$-NMR
C_6D_6, 100 MHz, 298 K

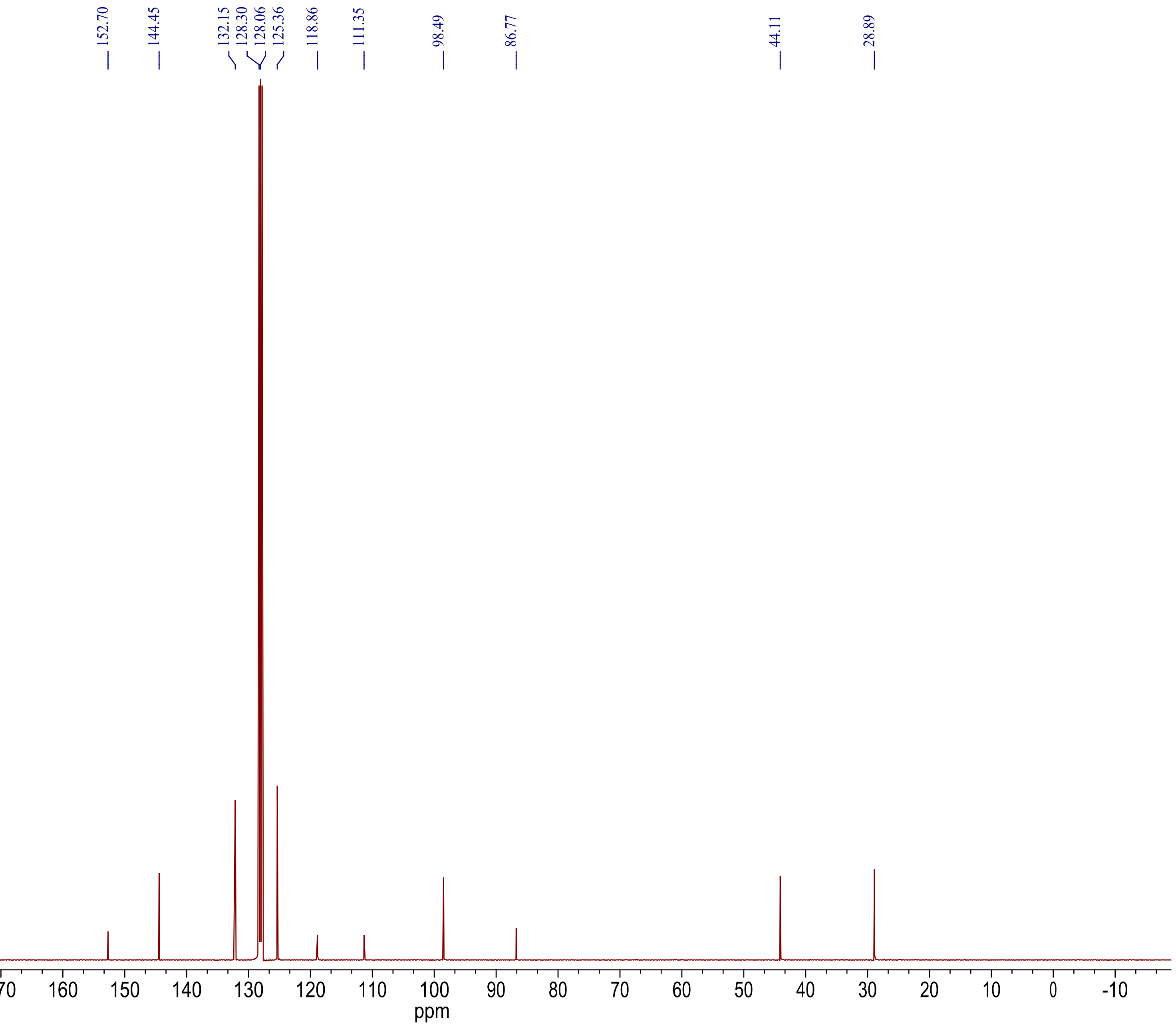

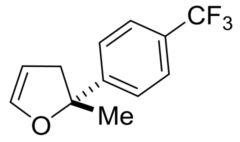

4am

^{1}H-NMR

C_6D_6, 400 MHz, 298 K

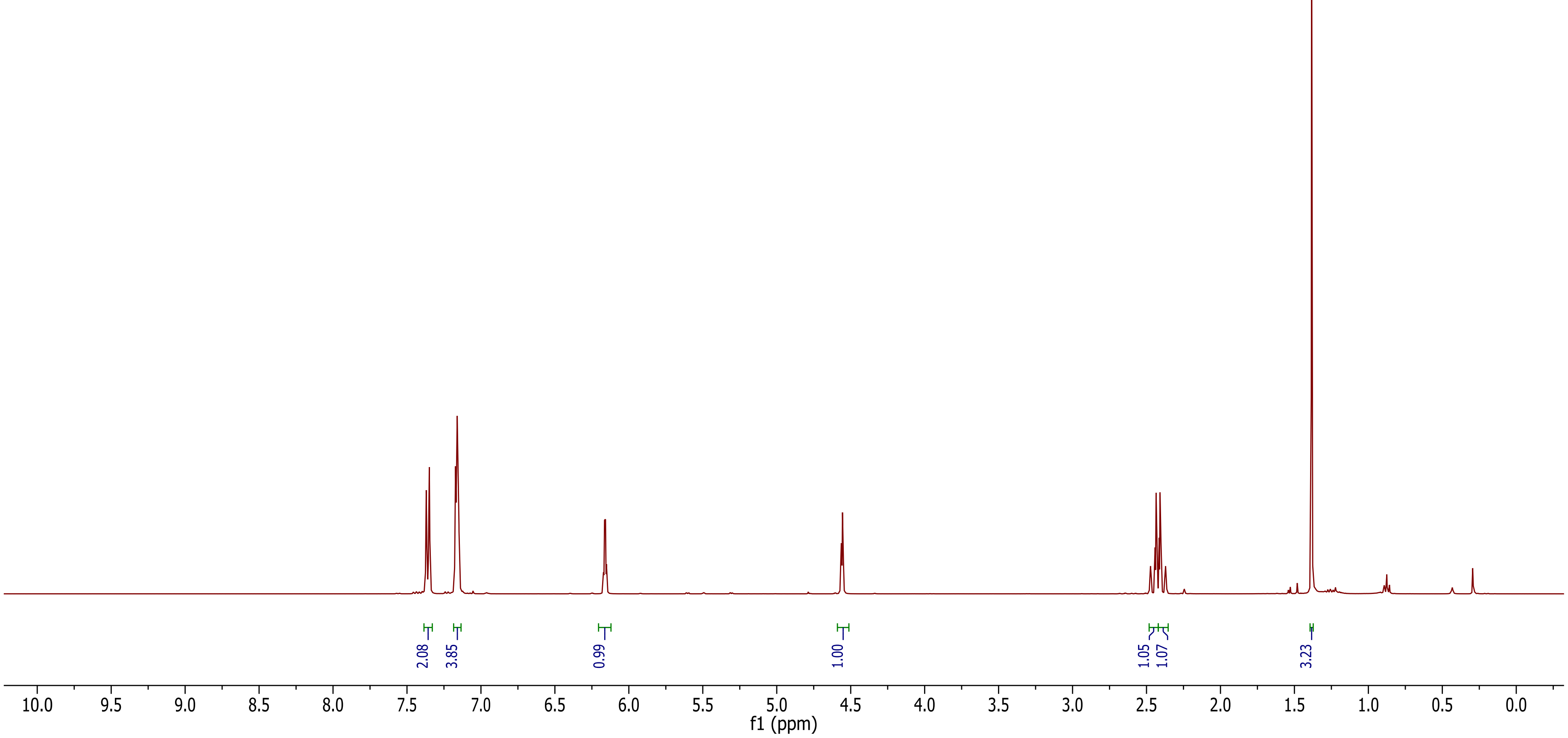

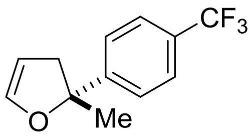

4am

^{19}F-NMR

C_6D_6, 282 MHz, 298 K

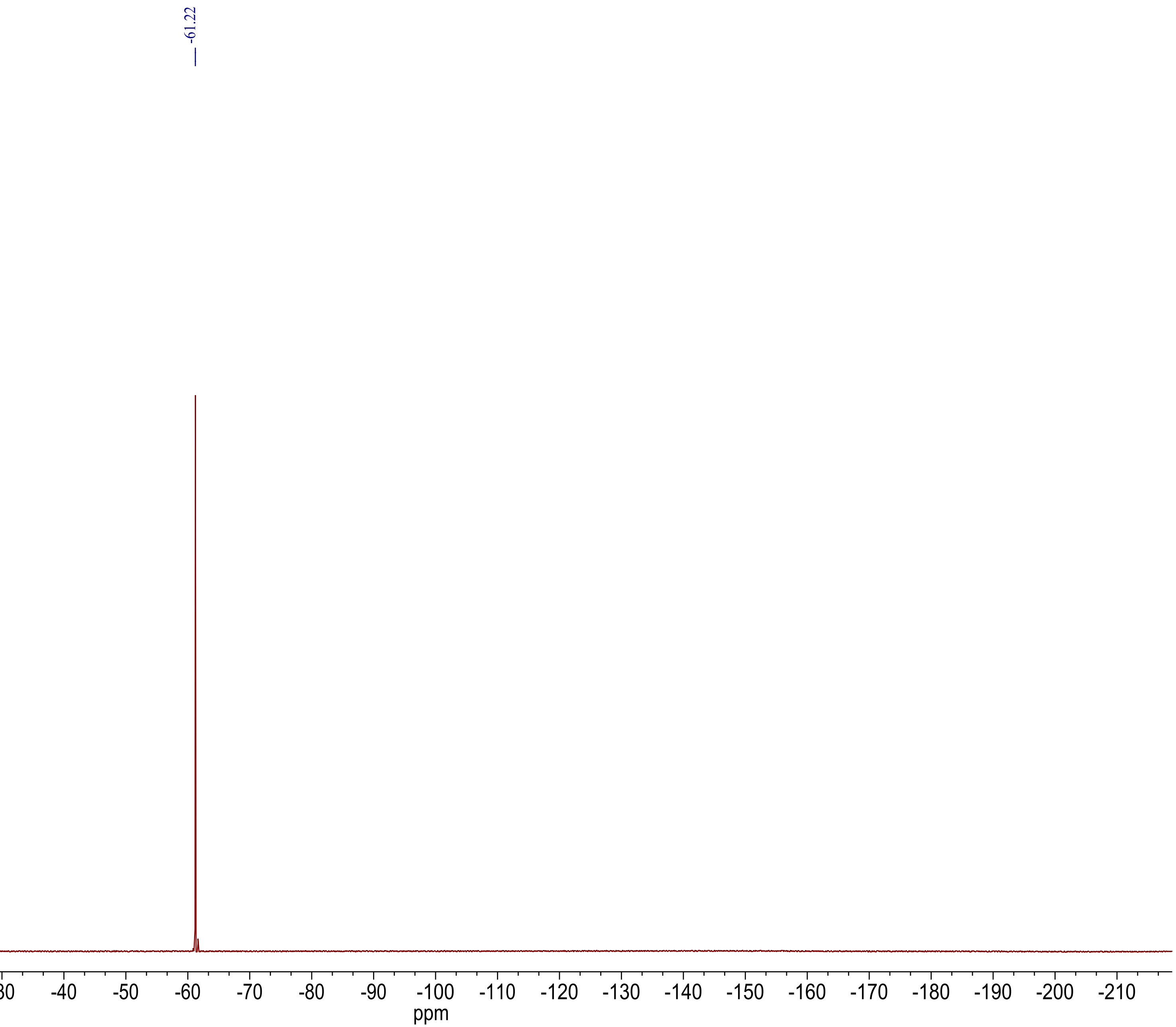

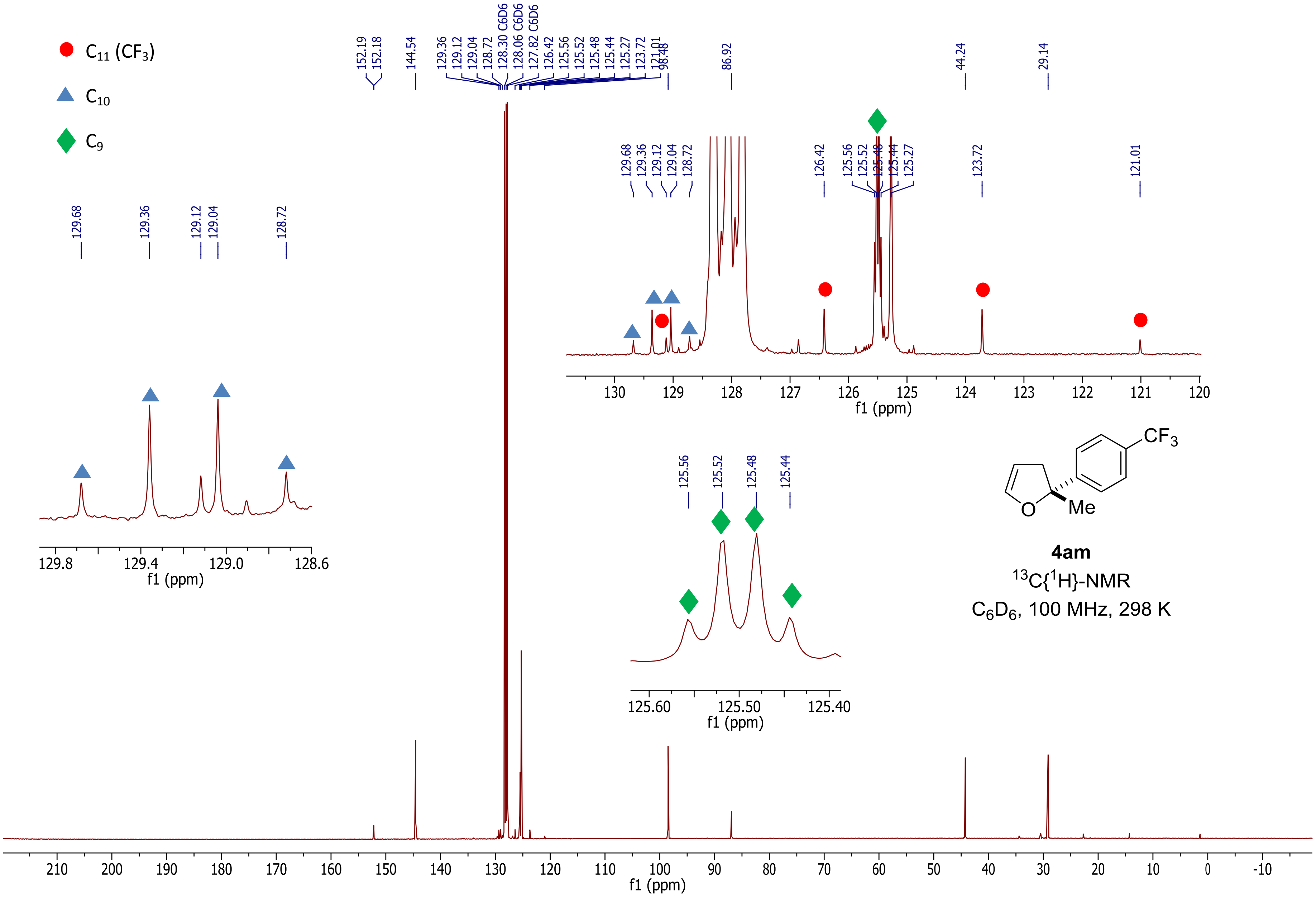

C11 (CF3)
C10
C9
4am
13C{1H}-NMR
C6D6, 100 MHz, 298 K
CF3
O
Me
f1 (ppm)

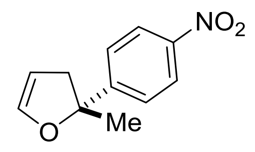

4an

^{1}H-NMR

C_6D_6, 400 MHz, 298 K

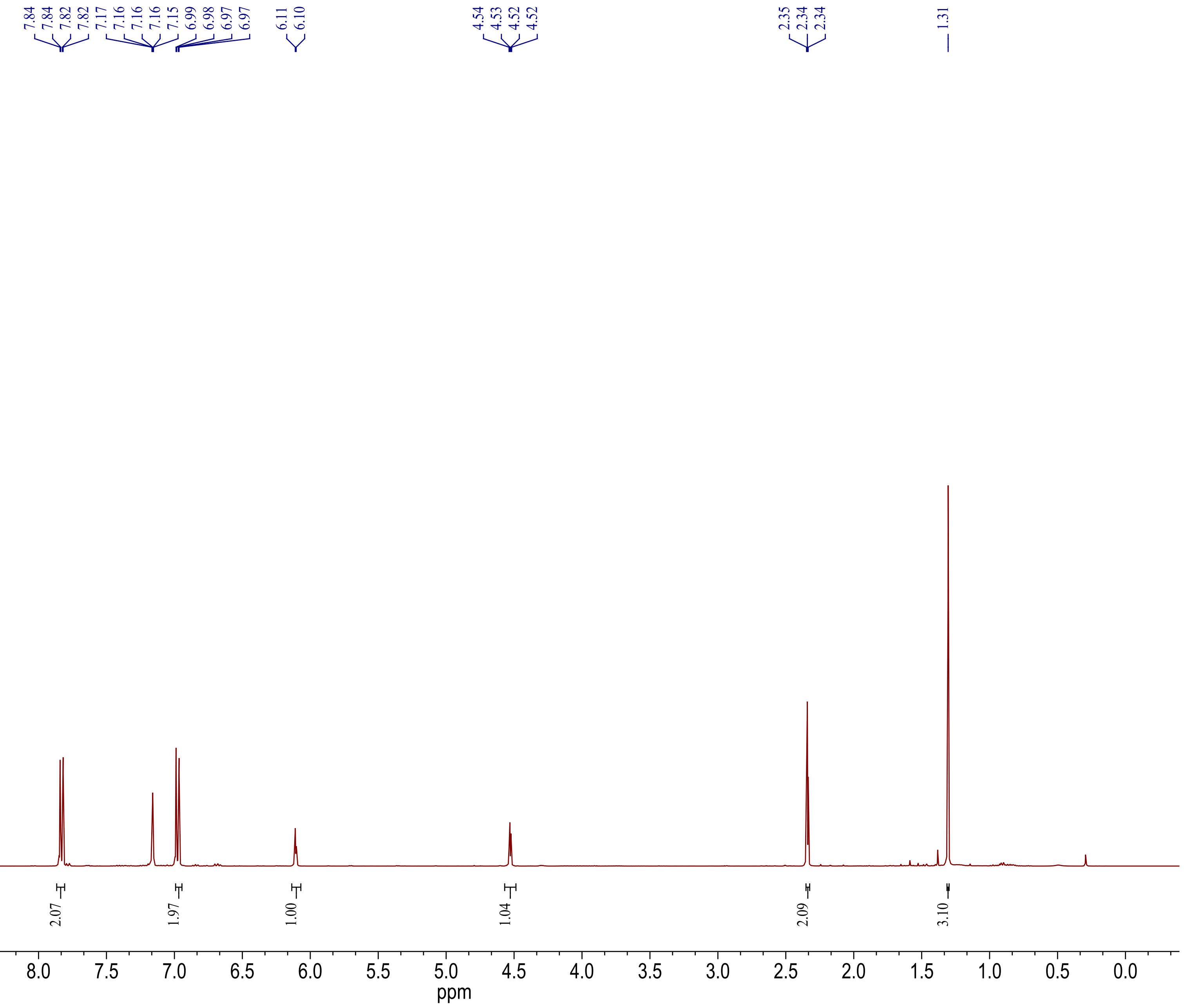

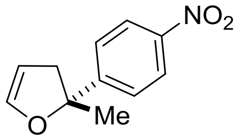

4an

$^{13}C\{^1H\}$-NMR

C_6D_6, 100 MHz, 298 K

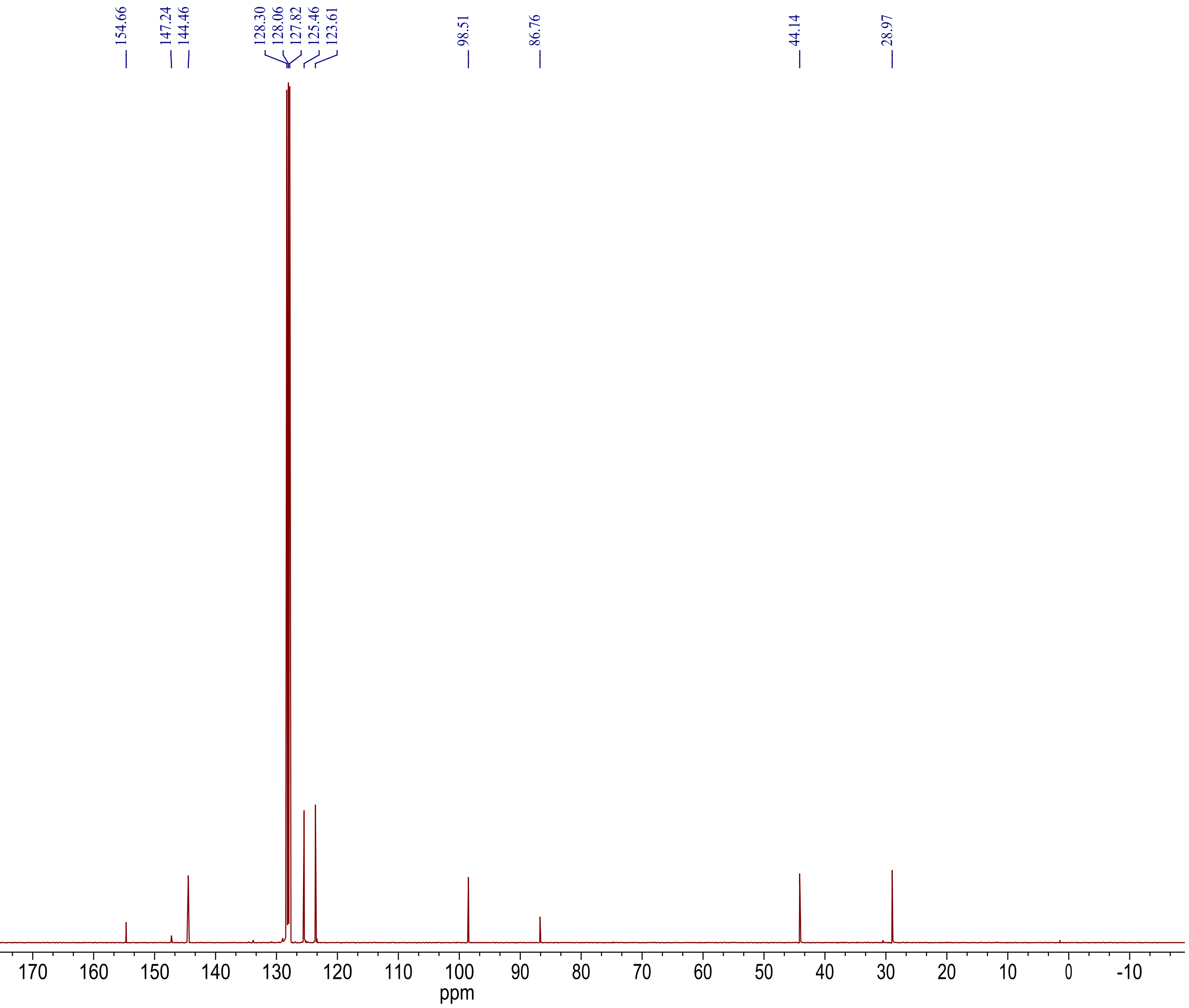

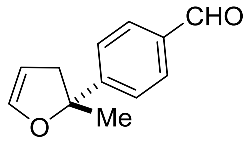

4ao
^{1}H-NMR
C_6D_6, 400 MHz, 298 K

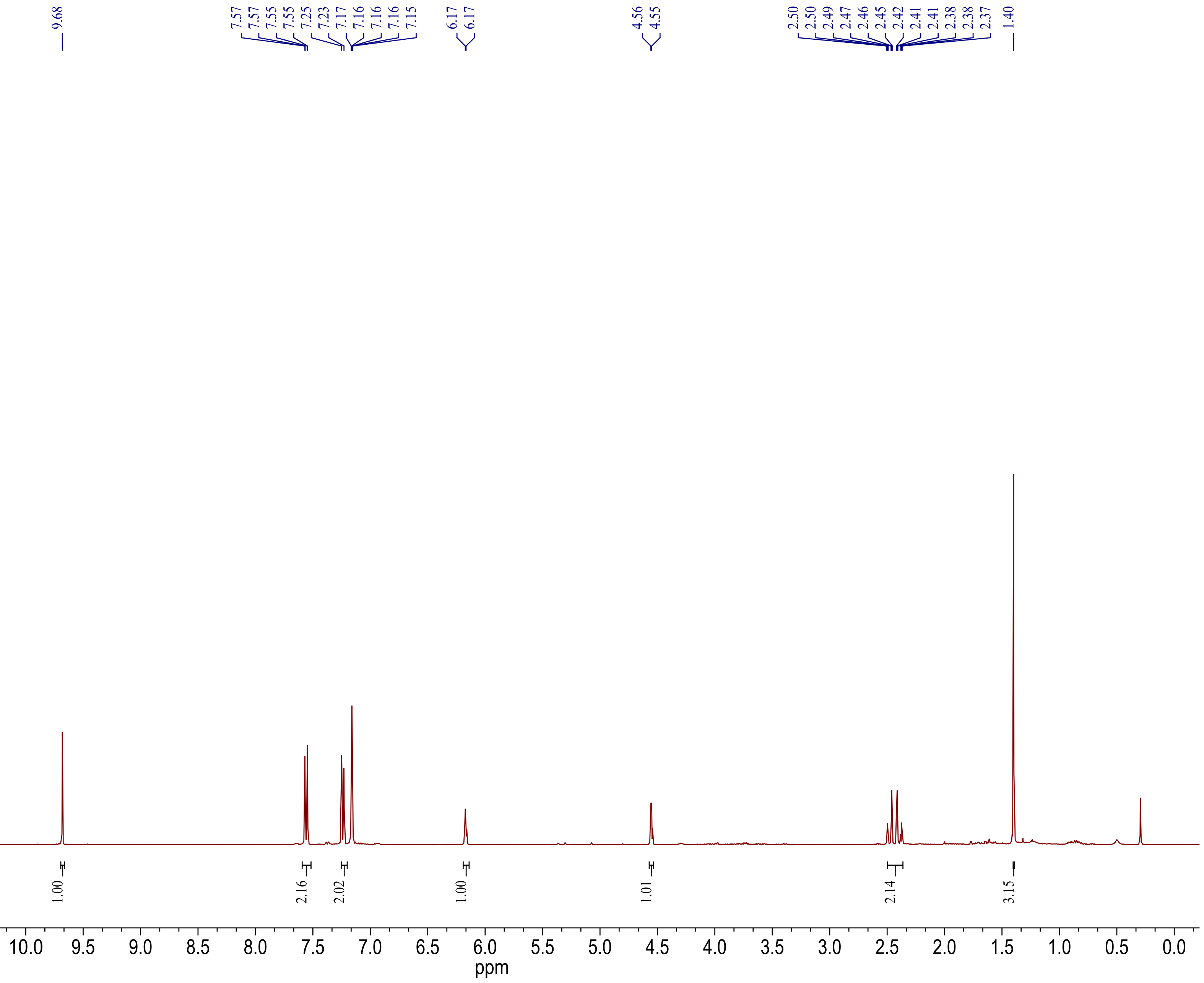

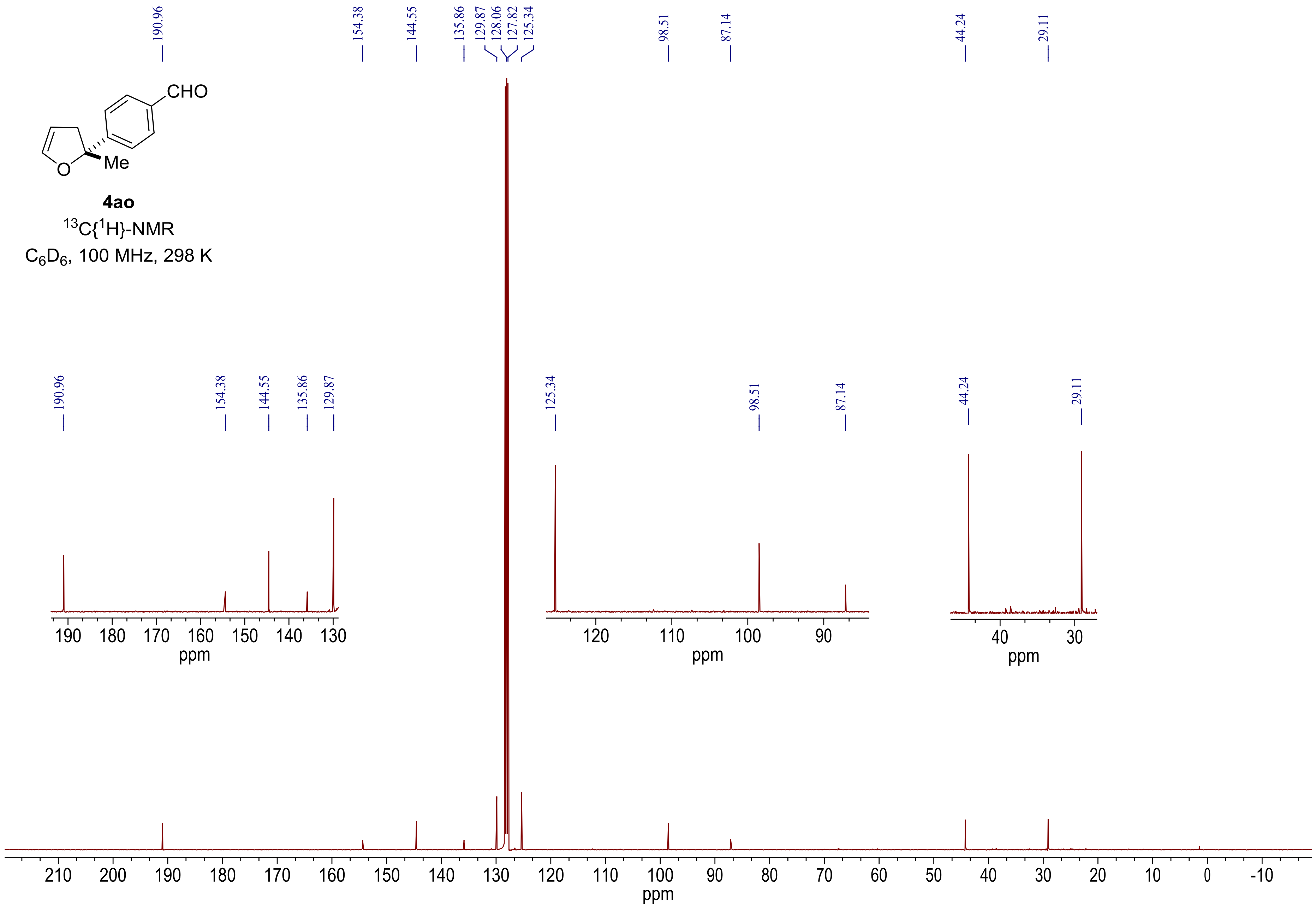
CHO
Me
O
4ao
13C{1H}-NMR
C6D6, 100 MHz, 298 K
190.96
154.38
144.55
135.86
129.87
128.06
127.82
125.34
98.51
87.14
44.24
29.11
ppm

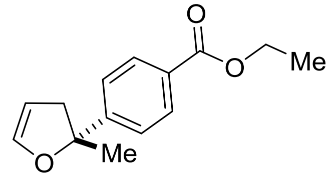

4ap
^{1}H-NMR
C_6D_6, 400 MHz, 298 K

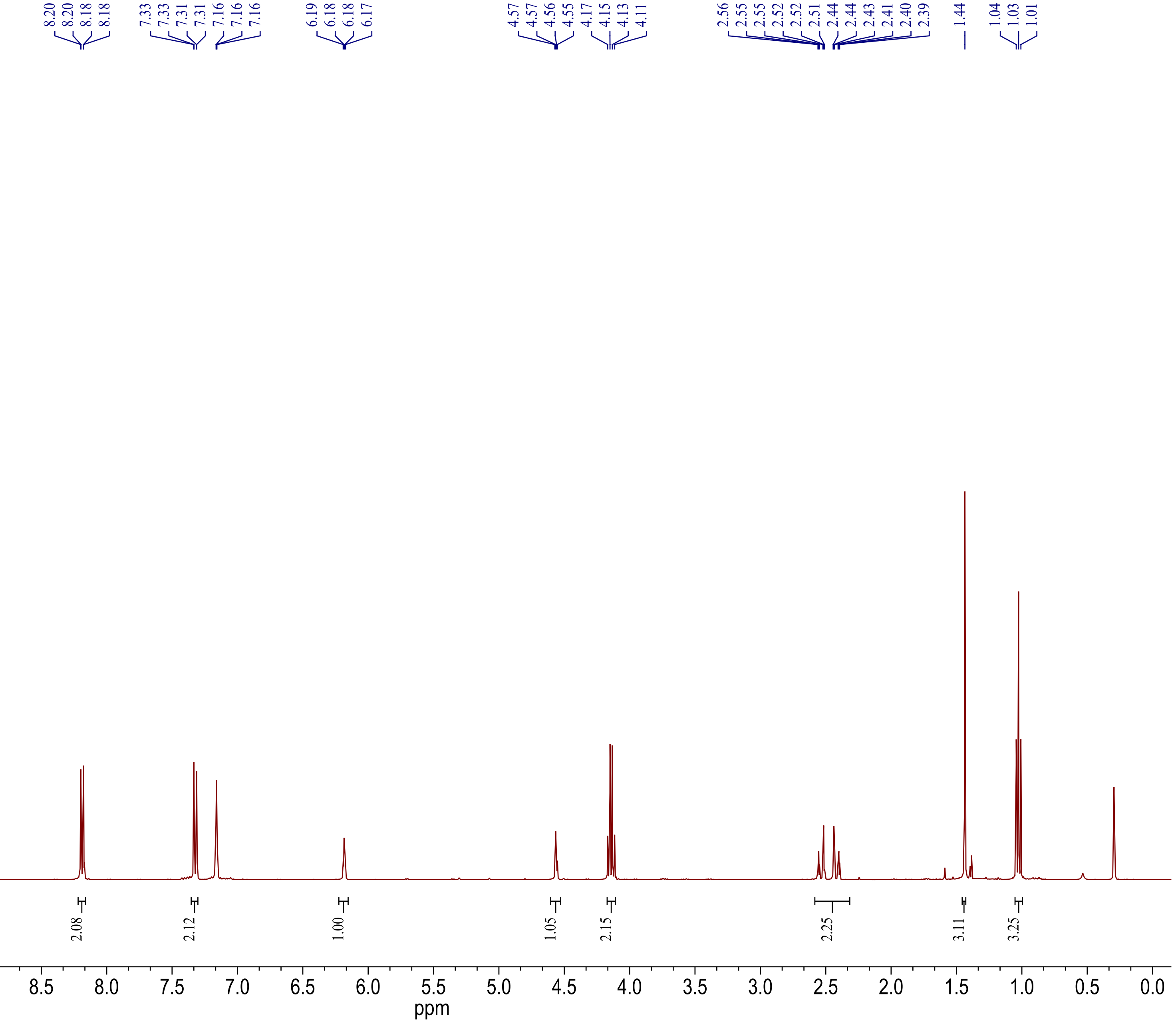

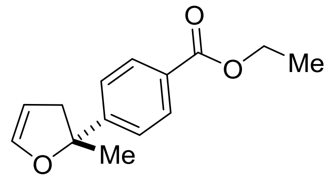

4ap
$^{13}C\{^{1}H\}$-NMR
C_6D_6, 100 MHz, 298 K

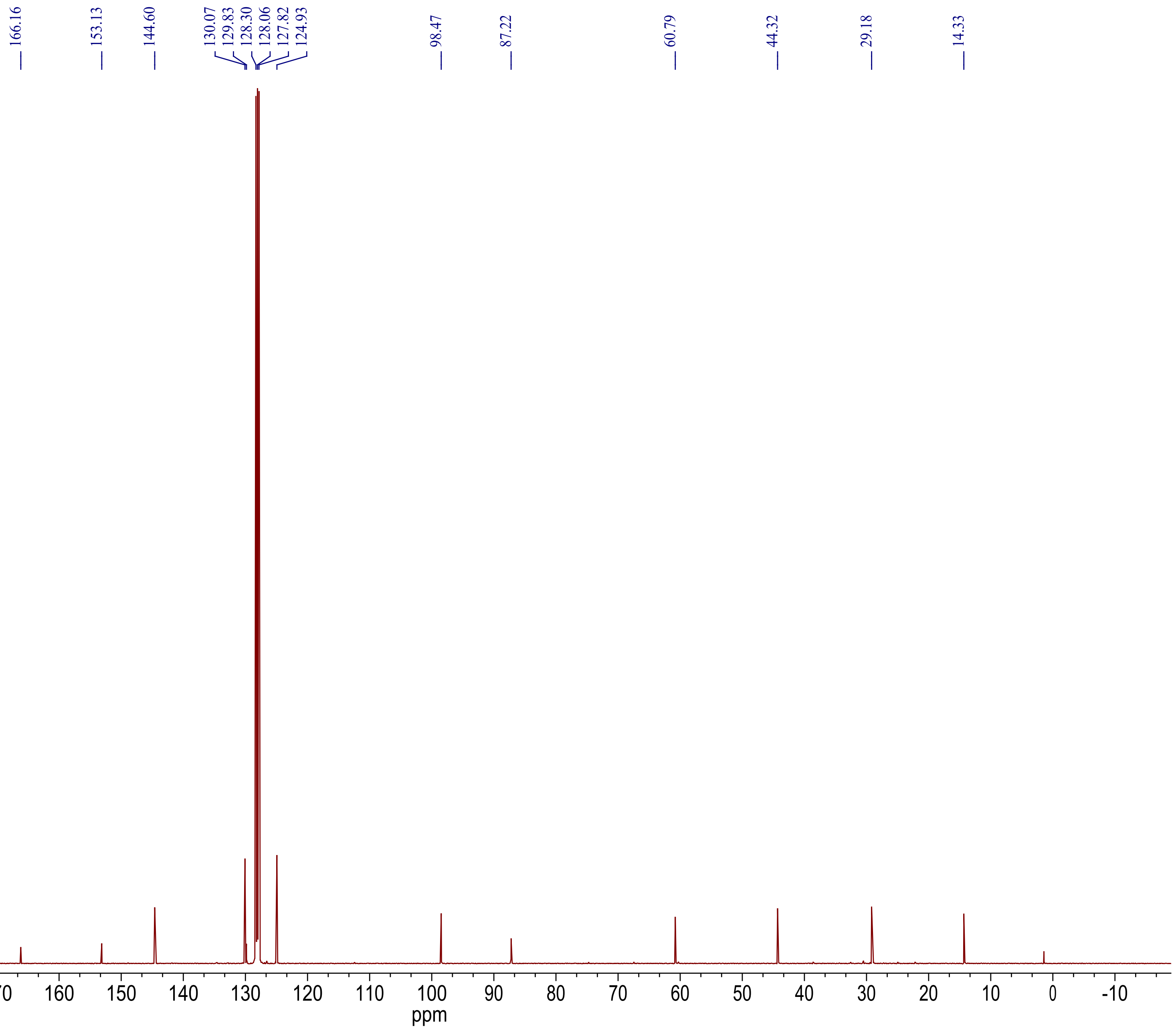

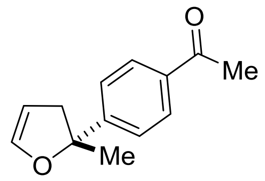

4aq
^{1}H-NMR
C_6D_6, 400 MHz, 298 K

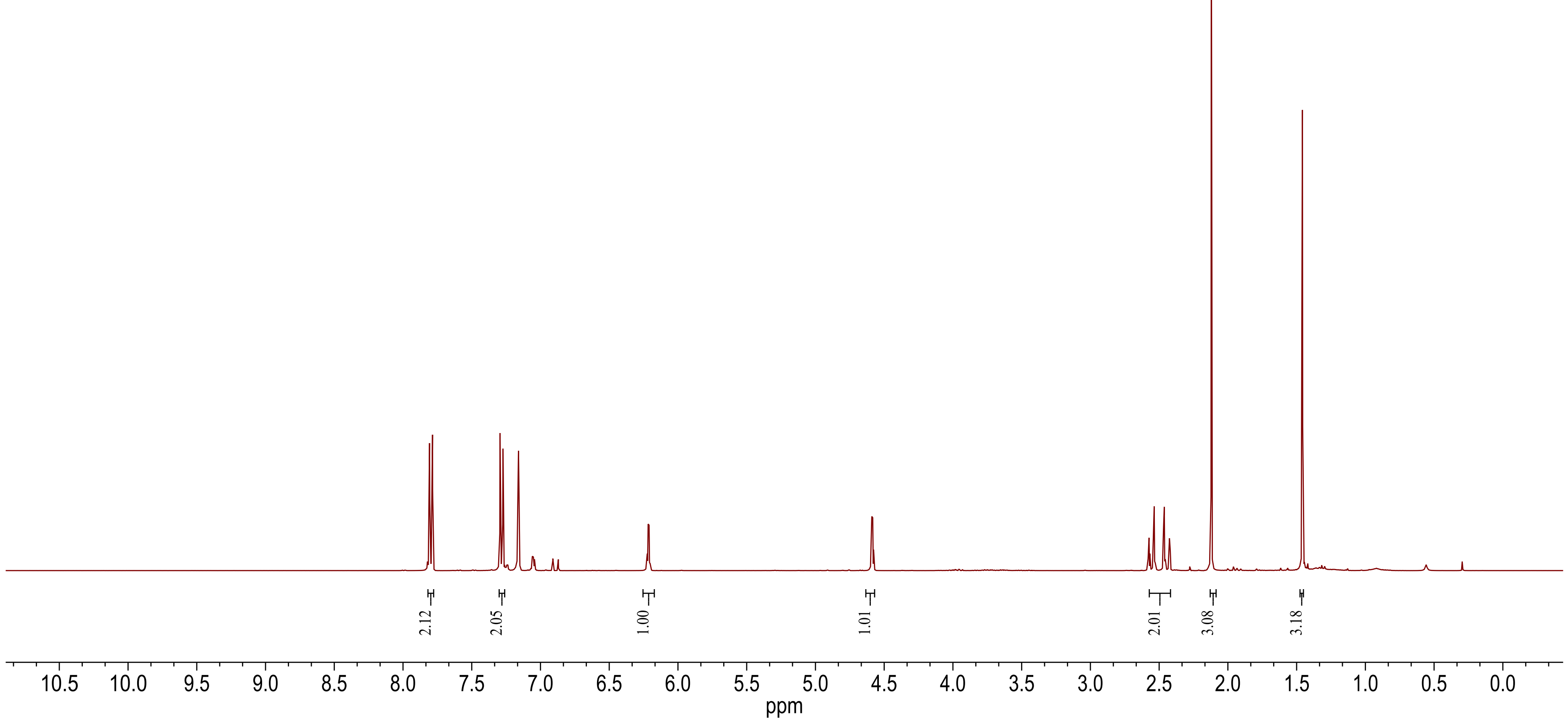

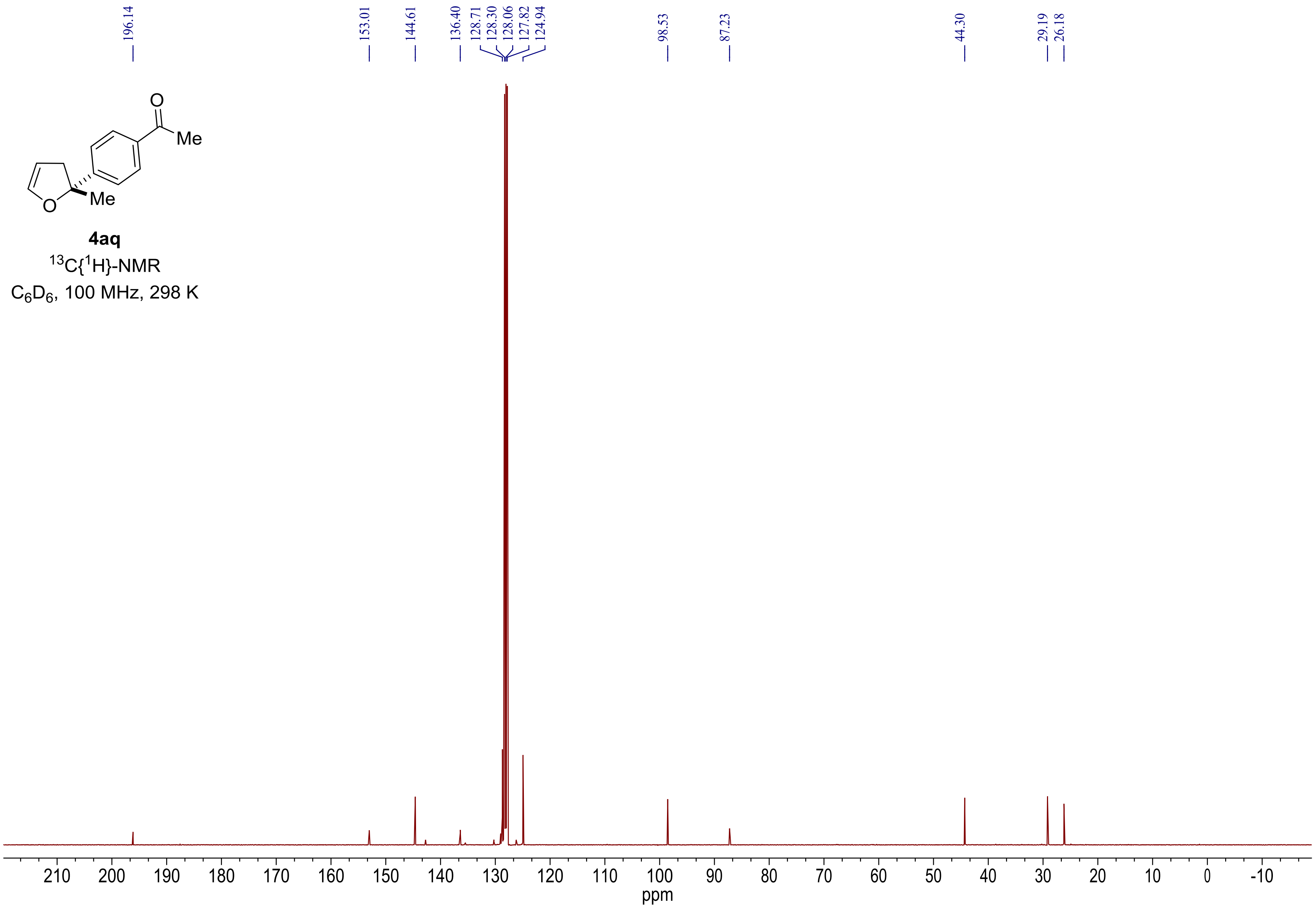
196.14
153.01
144.61
136.40
128.71
128.30
128.06
127.82
124.94
98.53
87.23
44.30
29.19
26.18
O
Me
Me
O
4aq
$^{13}C\{^1H\}$-NMR
C_6D_6, 100 MHz, 298 K
210 200 190 180 170 160 150 140 130 120 110 100 90 80 70 60 50 40 30 20 10 0 -10
ppm

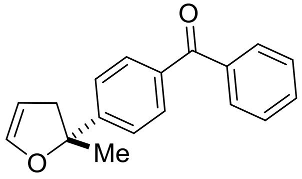

4ar

^{1}H-NMR

C_6D_6, 400 MHz, 298 K

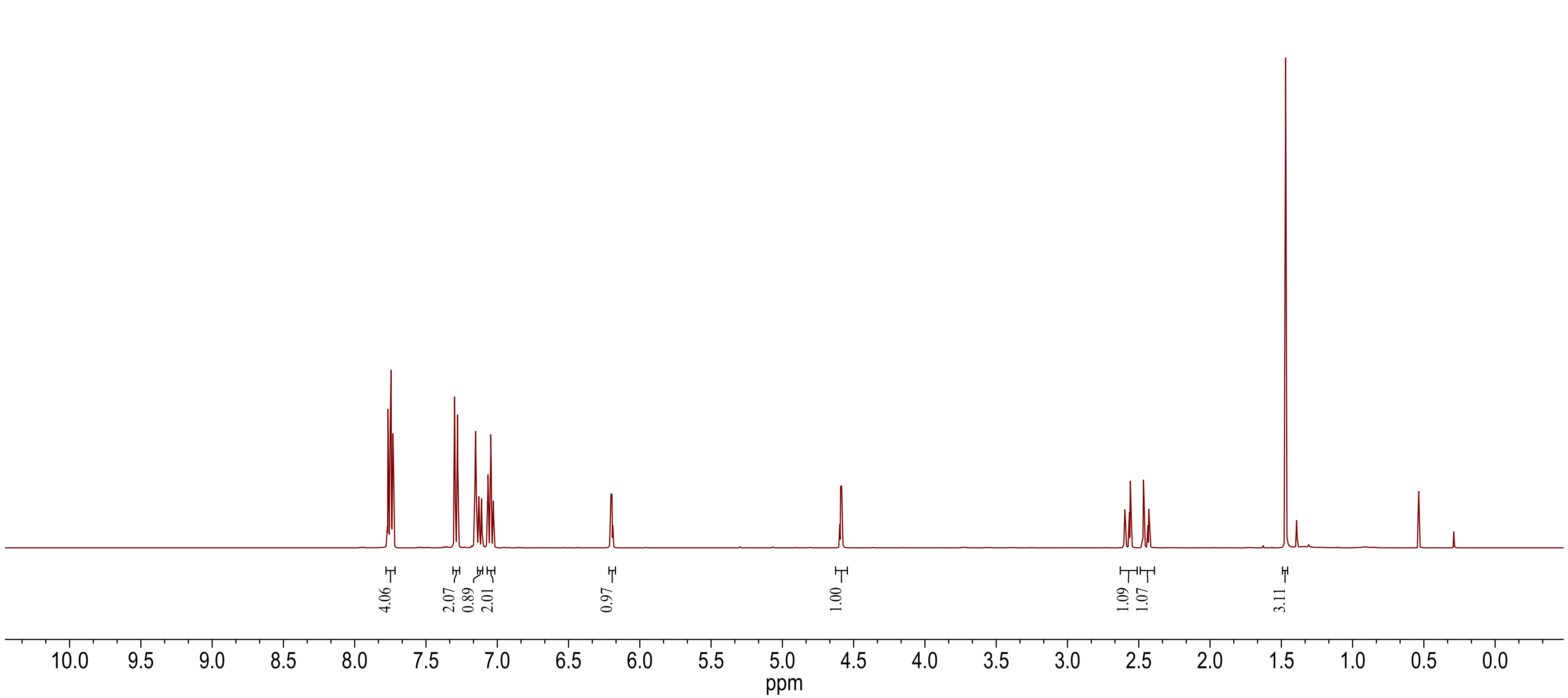

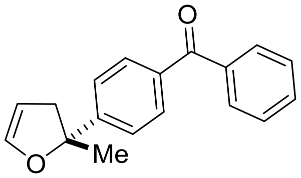

4ar

$^{13}C\{^{1}H\}$-NMR

C_6D_6, 100 MHz, 298 K

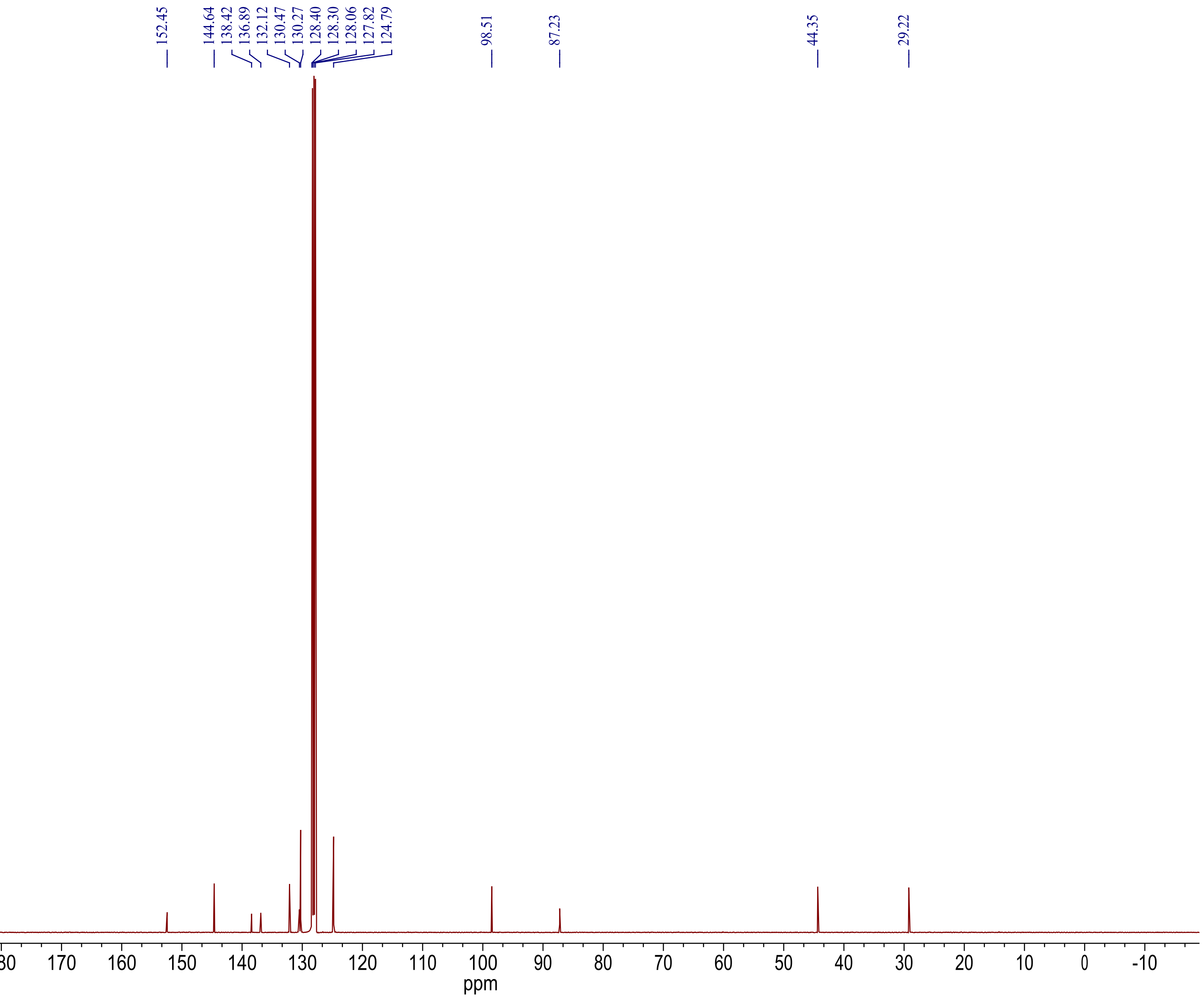

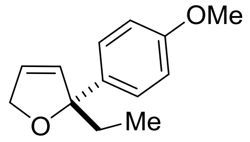

3bb

^{1}H-NMR

C_6D_6, 400 MHz, 298 K

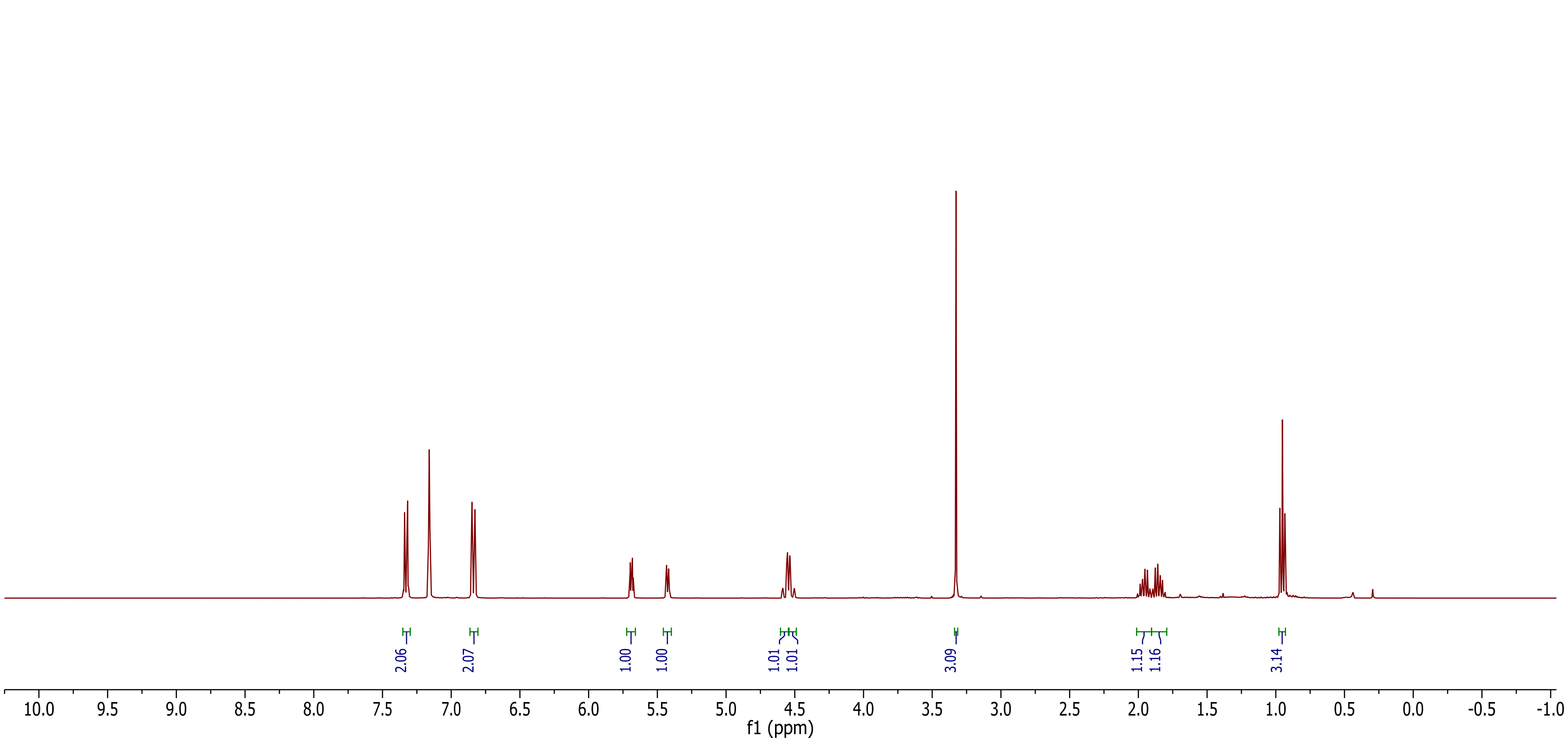

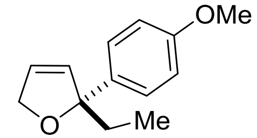

3bb

$^{13}C\{^{1}H\}$-NMR

C_6D_6, 100 MHz, 298 K

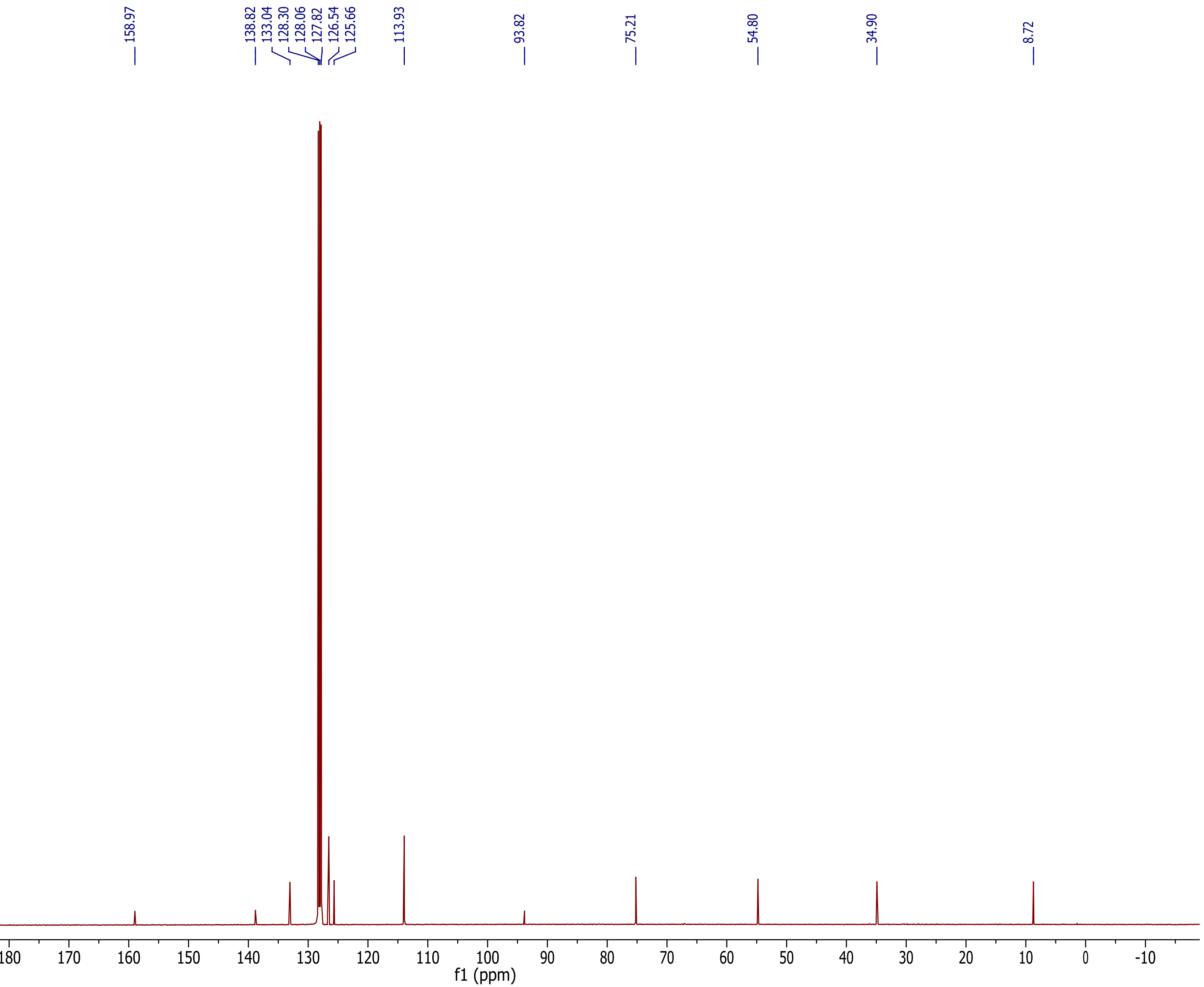

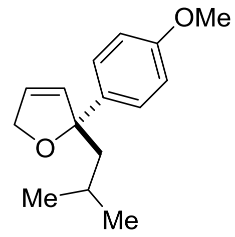

3cb
^{1}H-NMR
C_6D_6, 500 MHz, 298 K

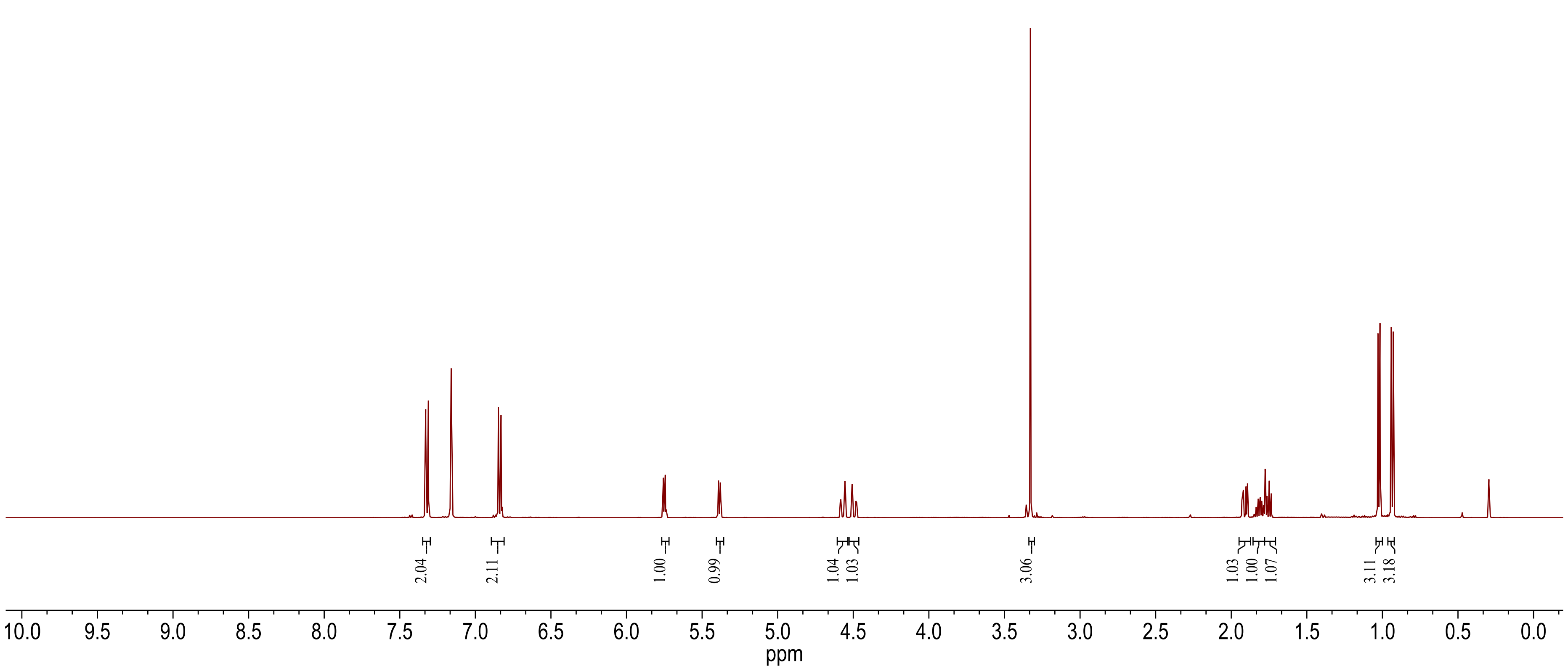

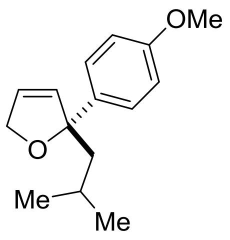

3cb

$^{13}C\{^{1}H\}$-NMR

C_6D_6, 125 MHz, 298 K

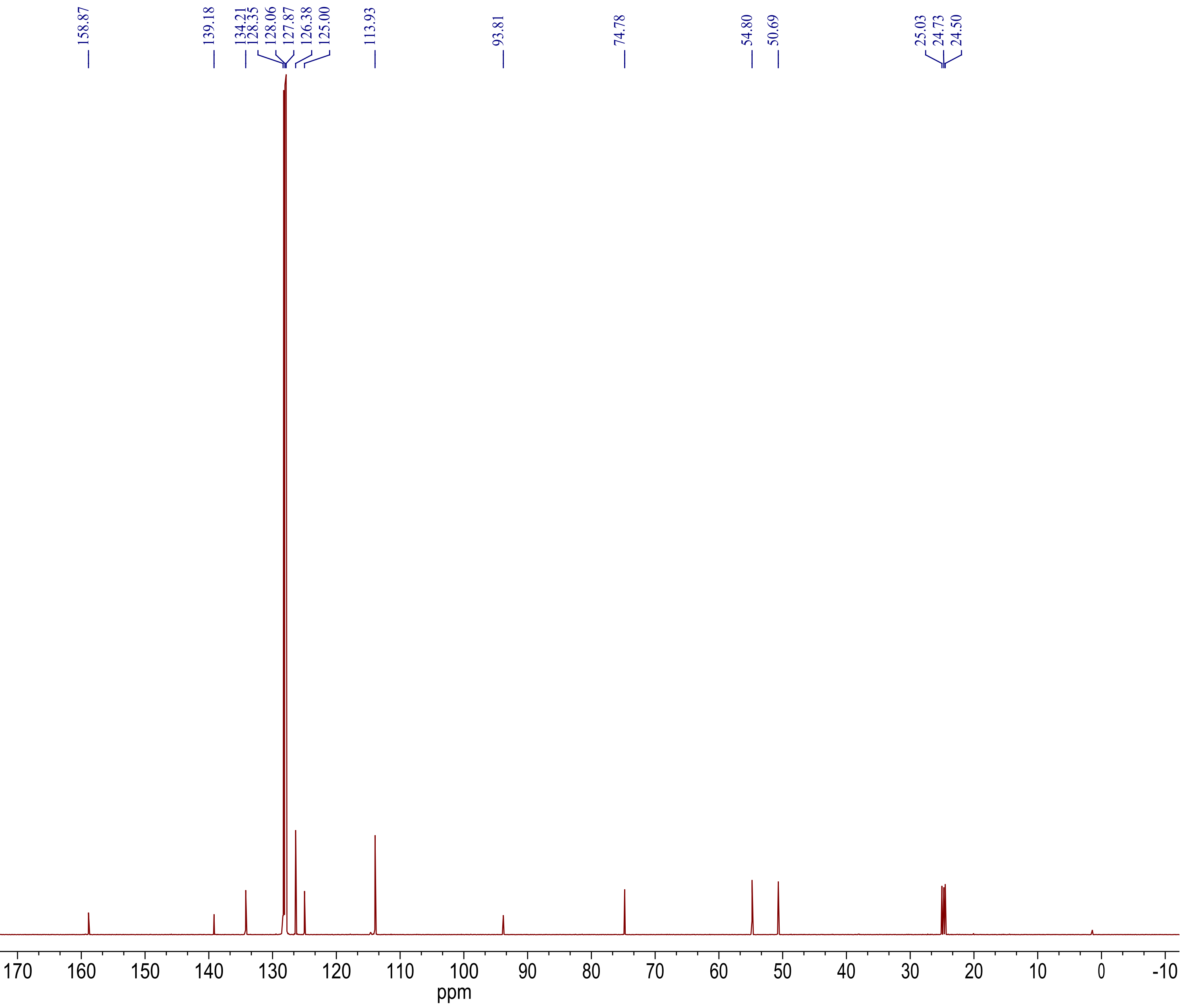

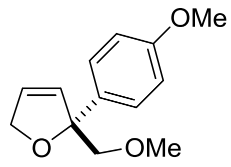

3db

^{1}H-NMR

C_6D_6, 400 MHz, 298 K

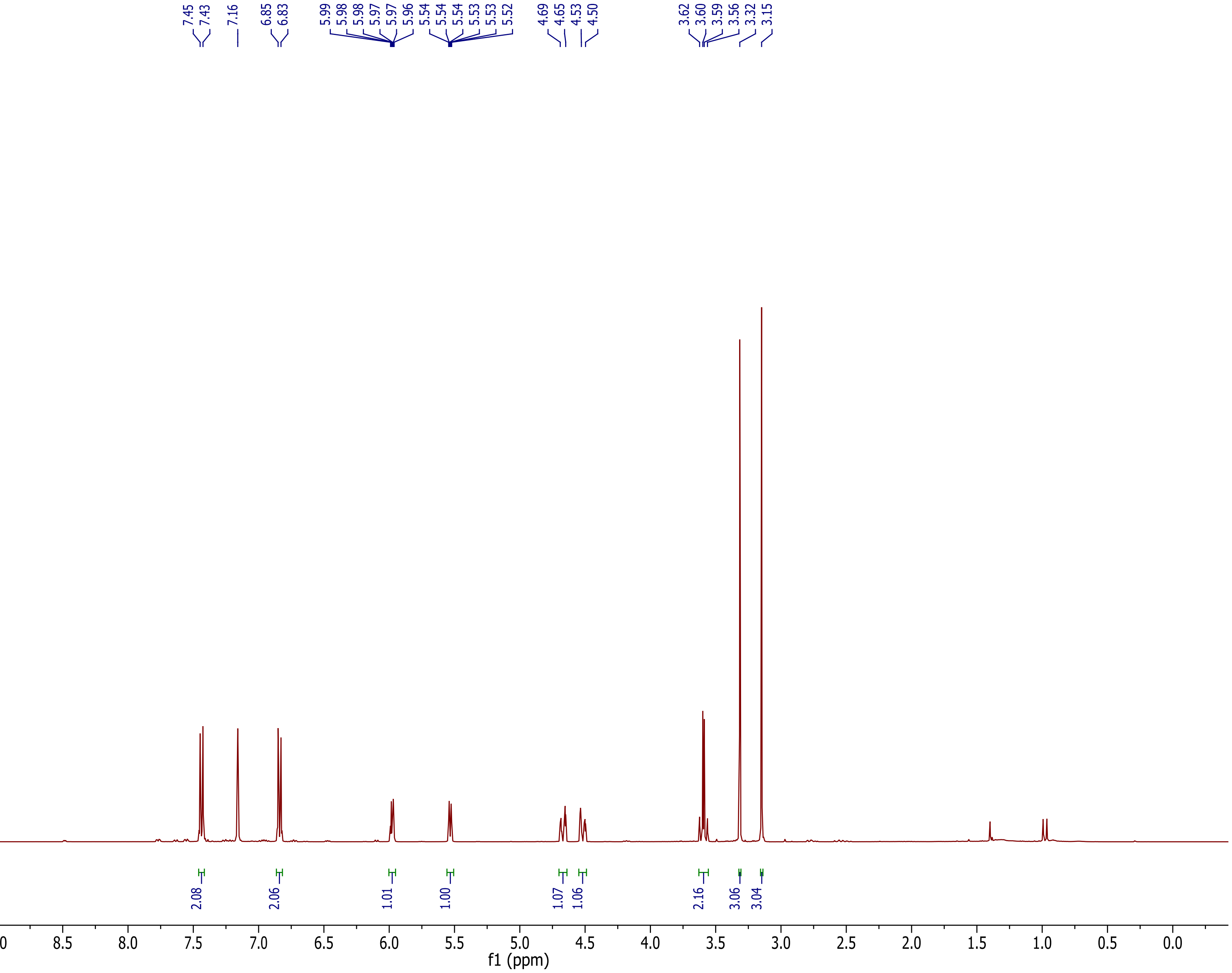

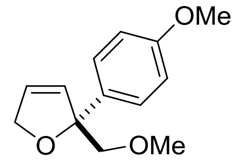

3db

$^{13}C\{^{1}H\}$-NMR

C_6D_6, 100 MHz, 298 K

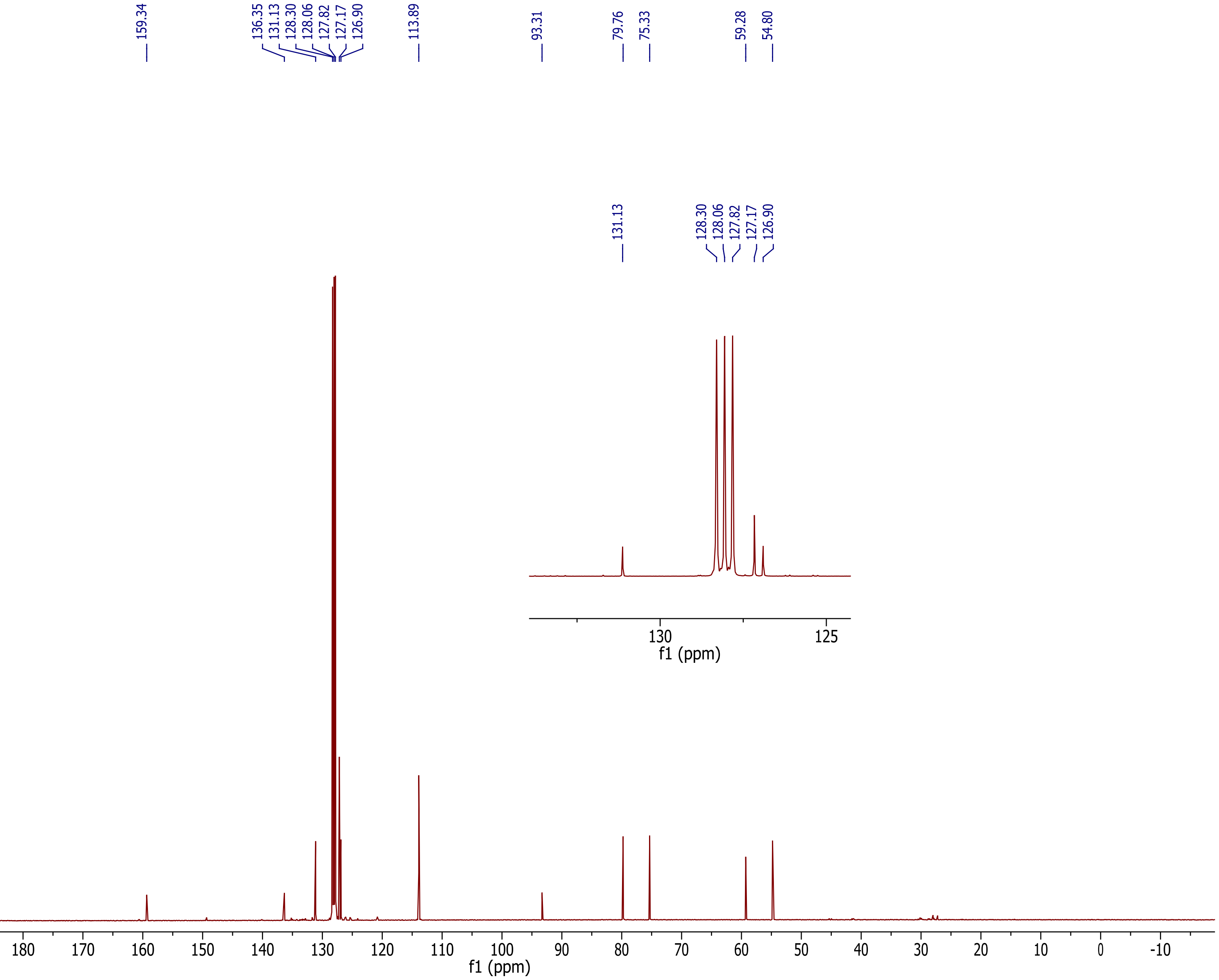

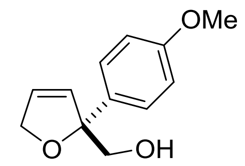

3eb
^{1}H-NMR
C_6D_6, 400 MHz, 298 K

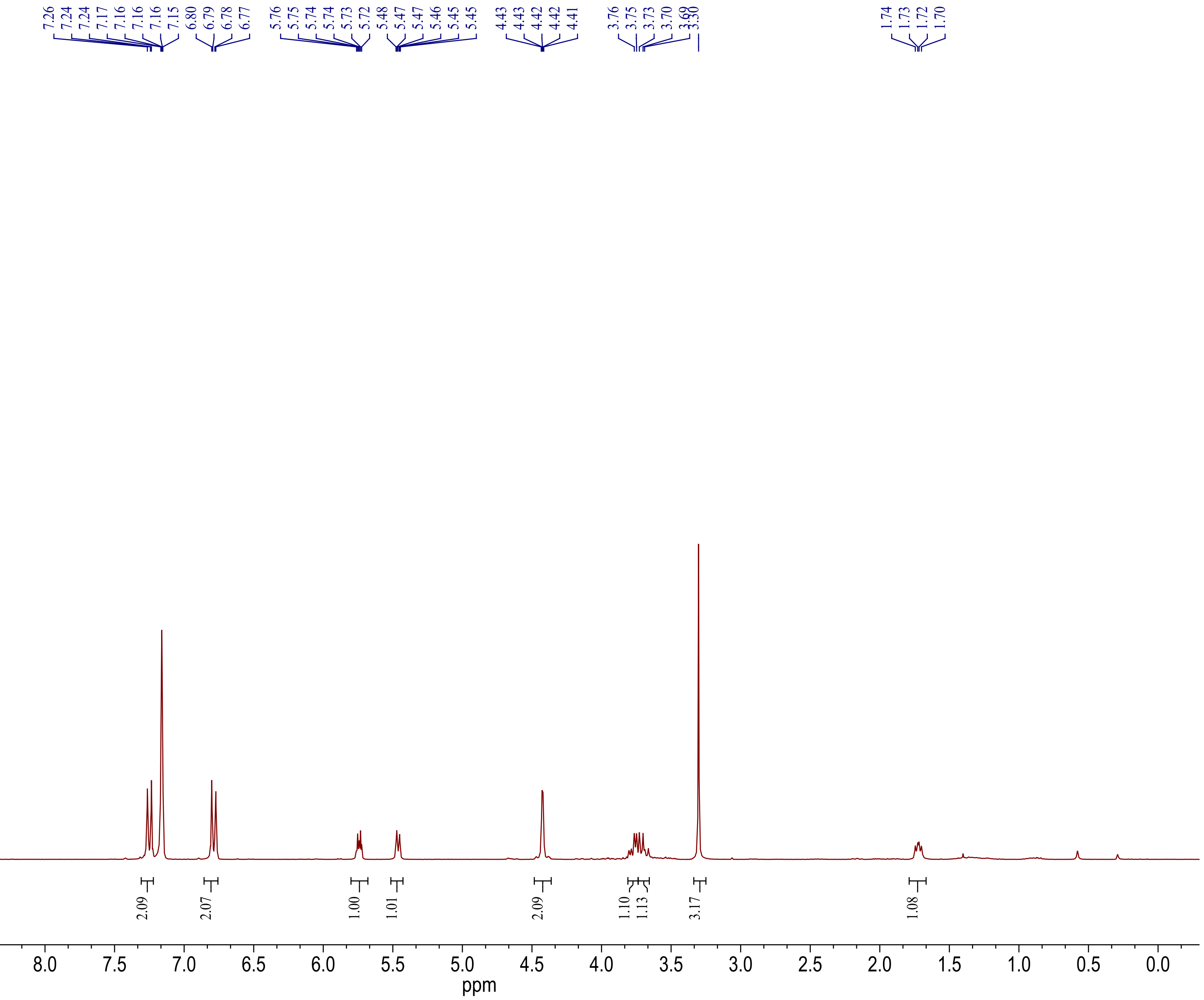

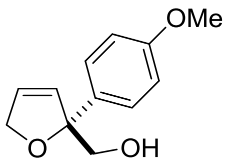

3eb

$^{13}C\{^1H\}$-NMR

C_6D_6, 100 MHz, 298 K

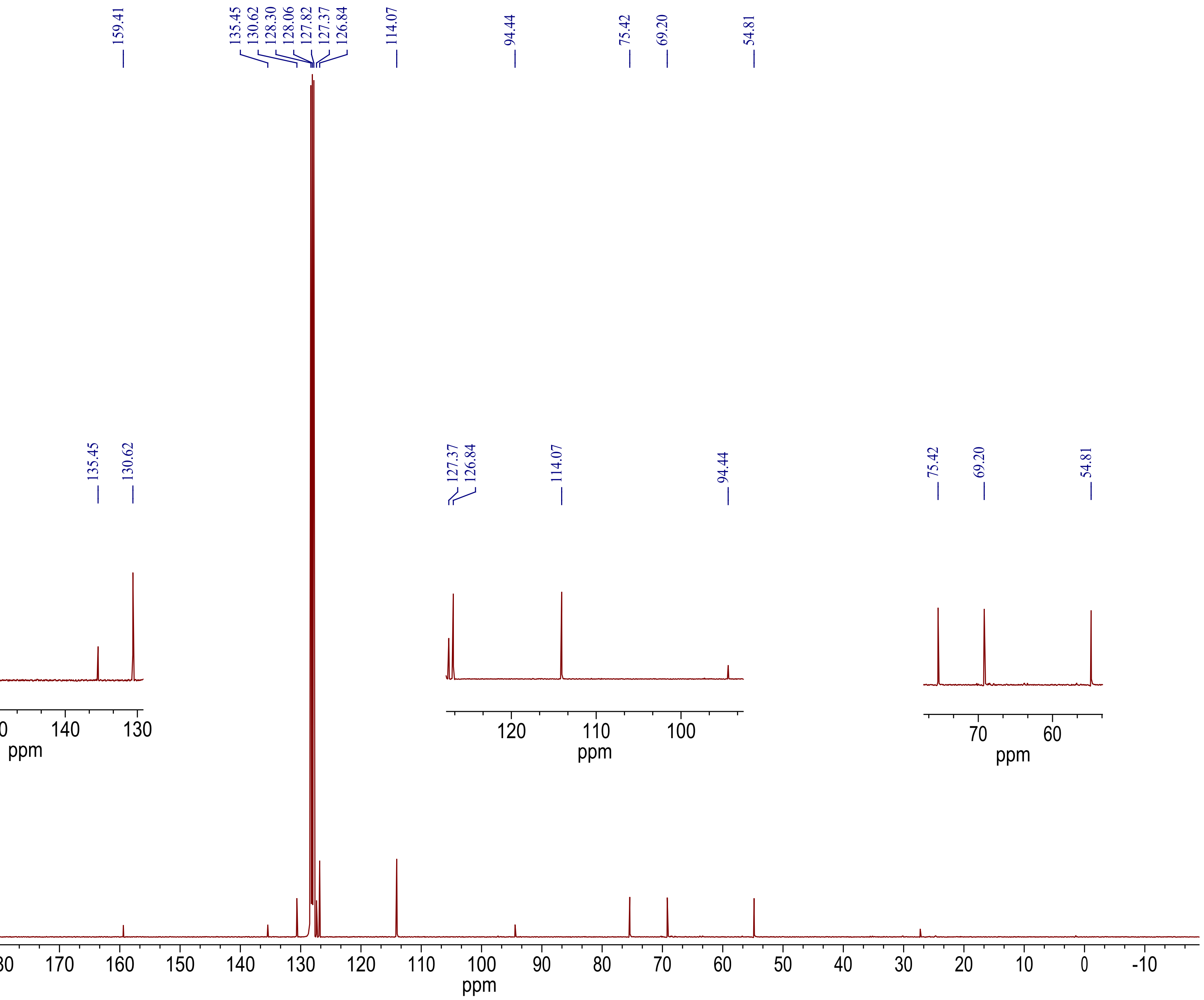

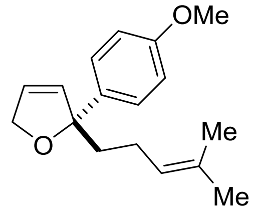

3fb
^{1}H-NMR
C_6D_6, 400 MHz, 298 K

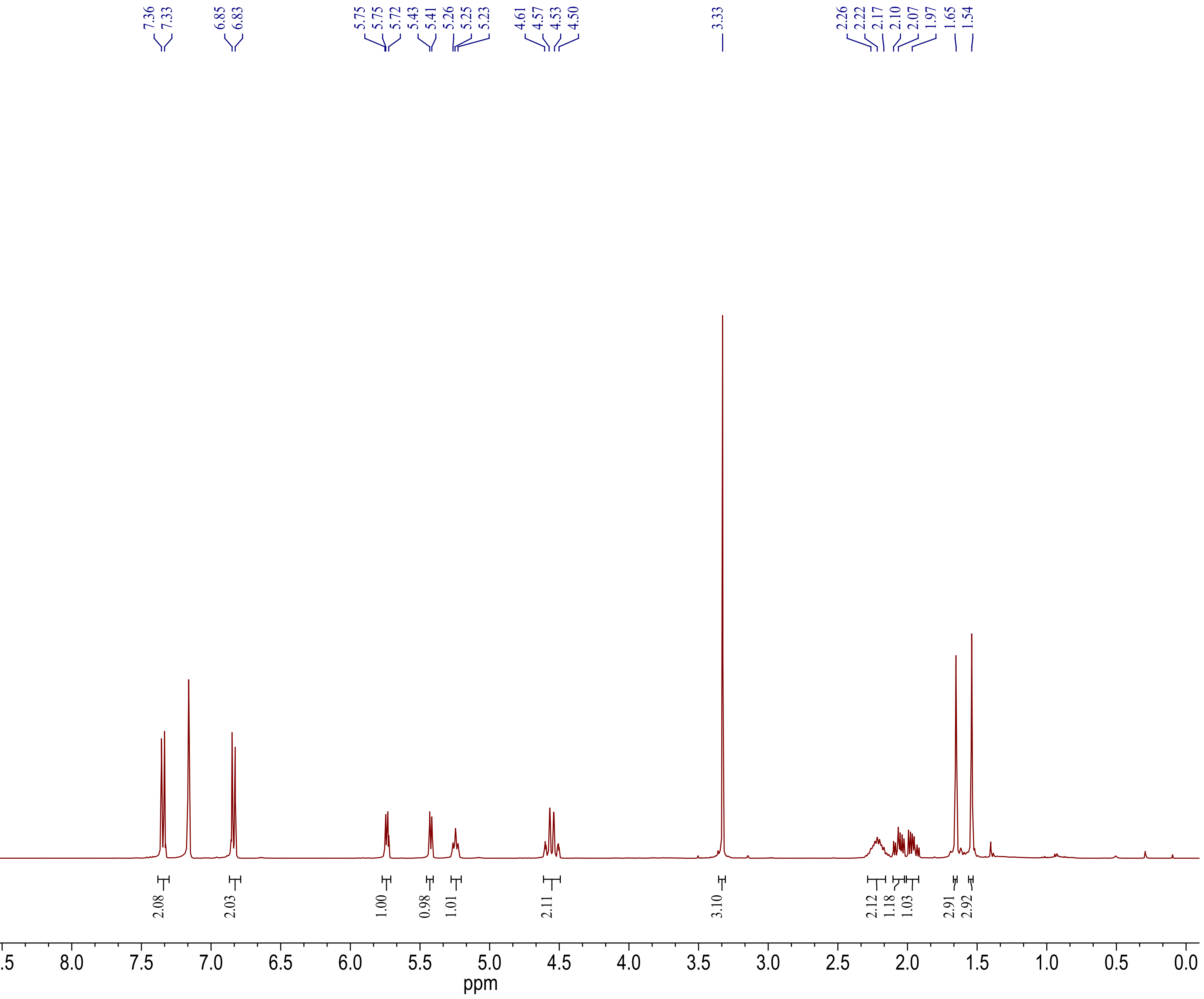

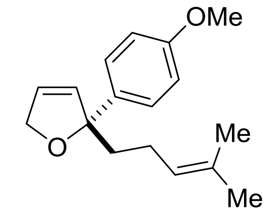

3fb

$^{13}C\{^{1}H\}$-NMR

C_6D_6, 100 MHz, 298 K

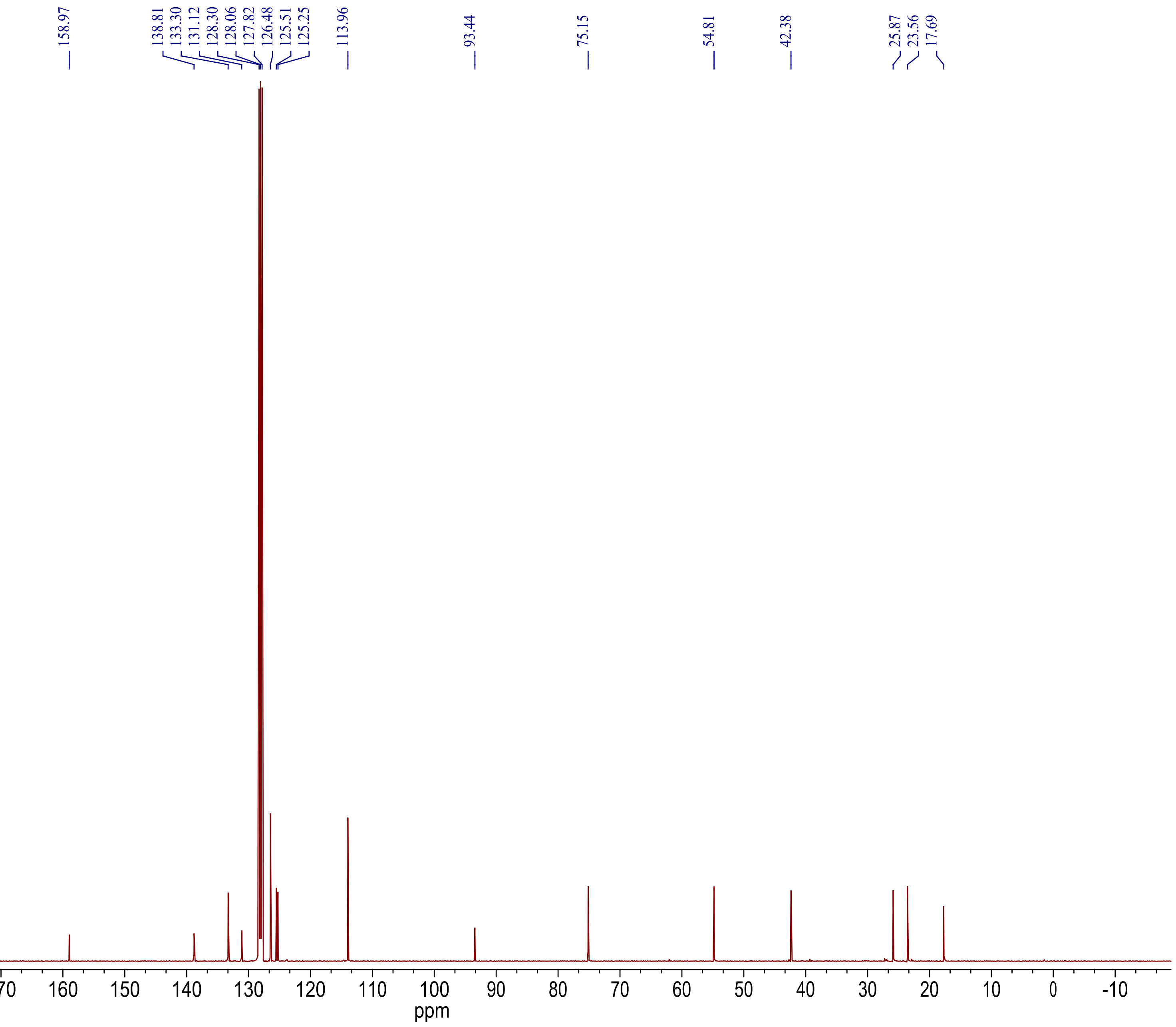

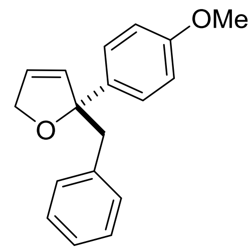

3gb

^{1}H-NMR

C_6D_6, 400 MHz, 298 K

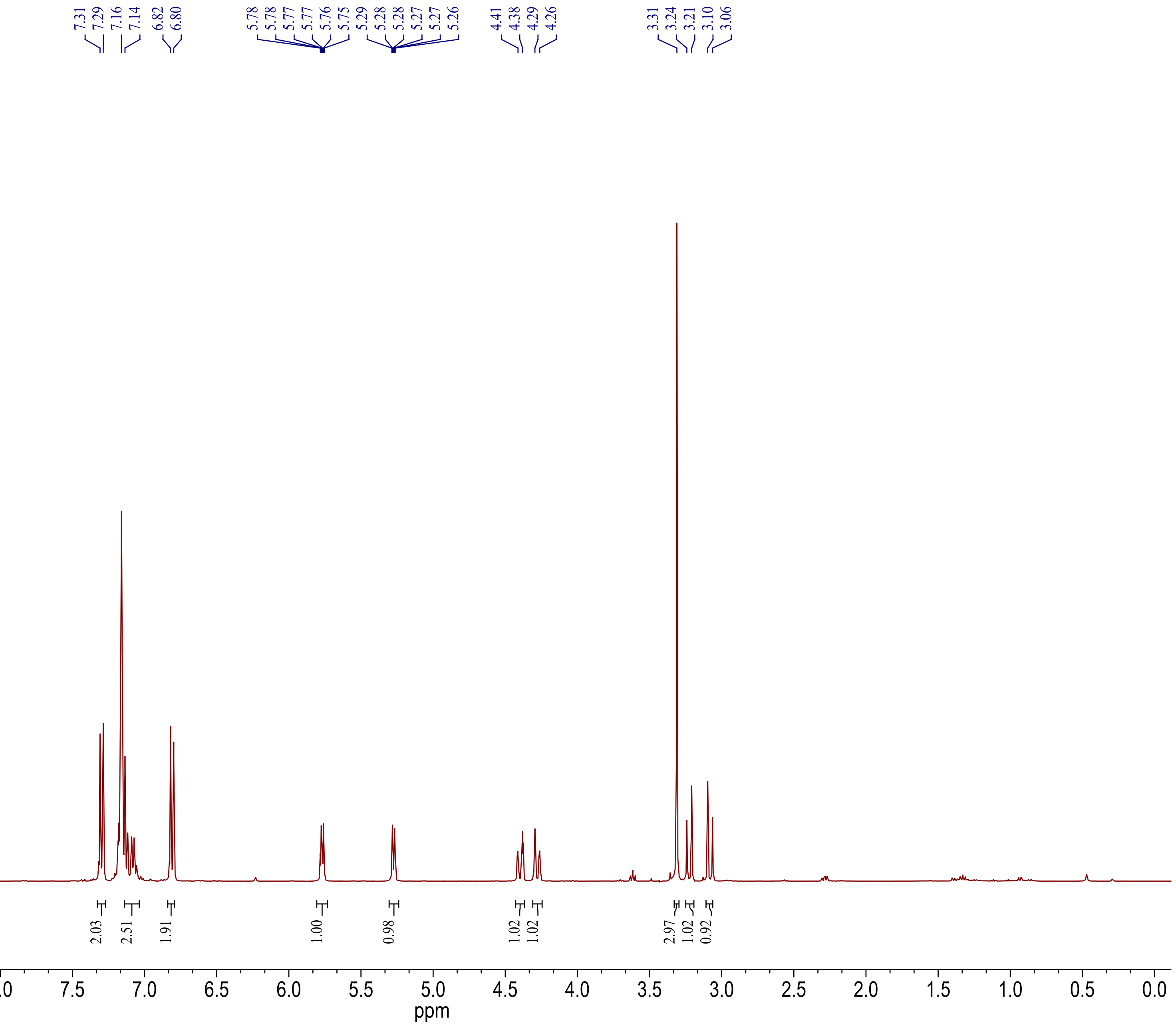

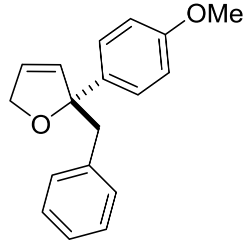

3gb
$^{13}C\{^{1}H\}$-NMR
C_6D_6, 100 MHz, 298 K

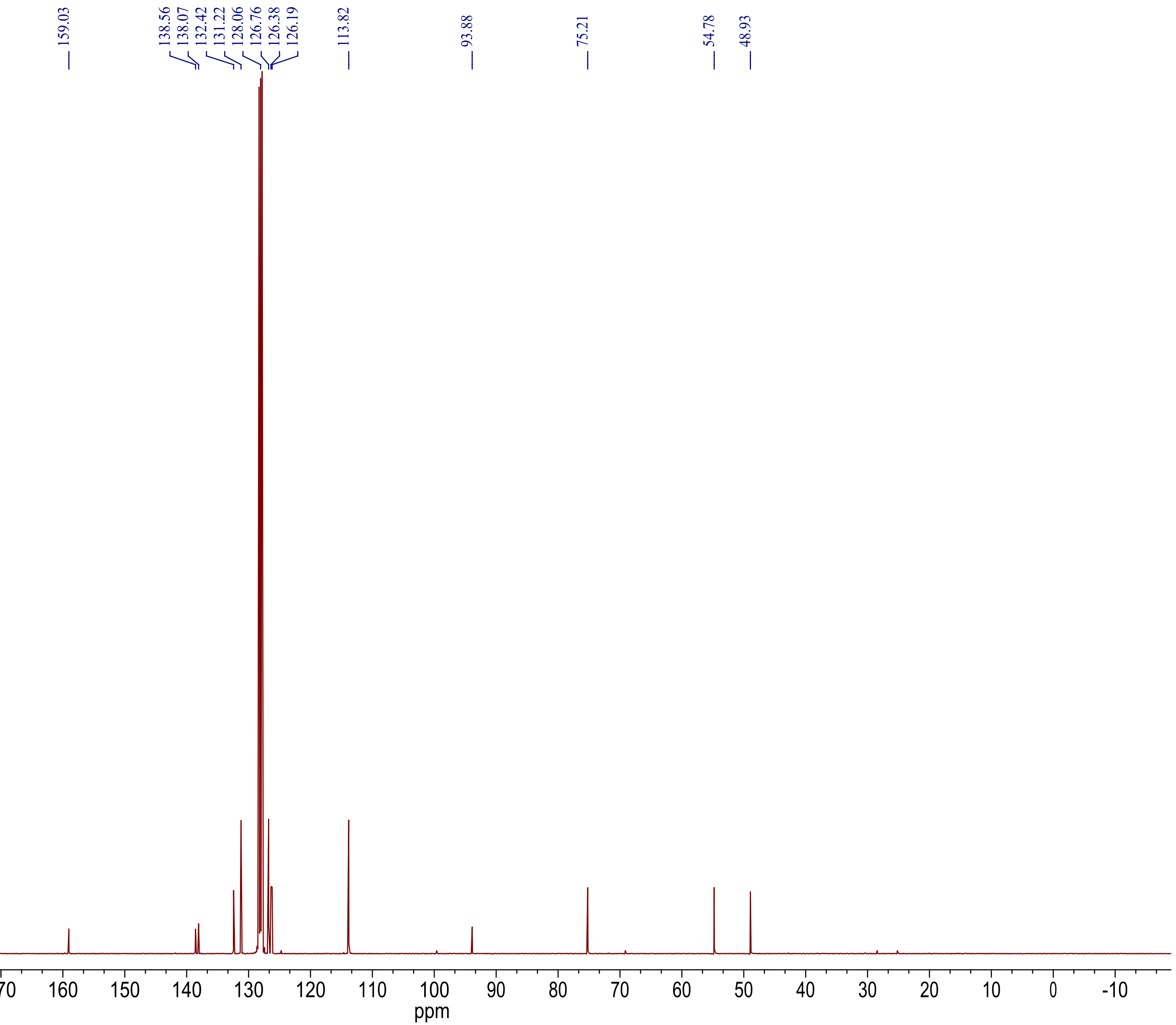

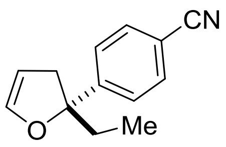

4bc

$^{13}C\{^{1}H\}$-NMR

C_6D_6, 100 MHz, 298 K

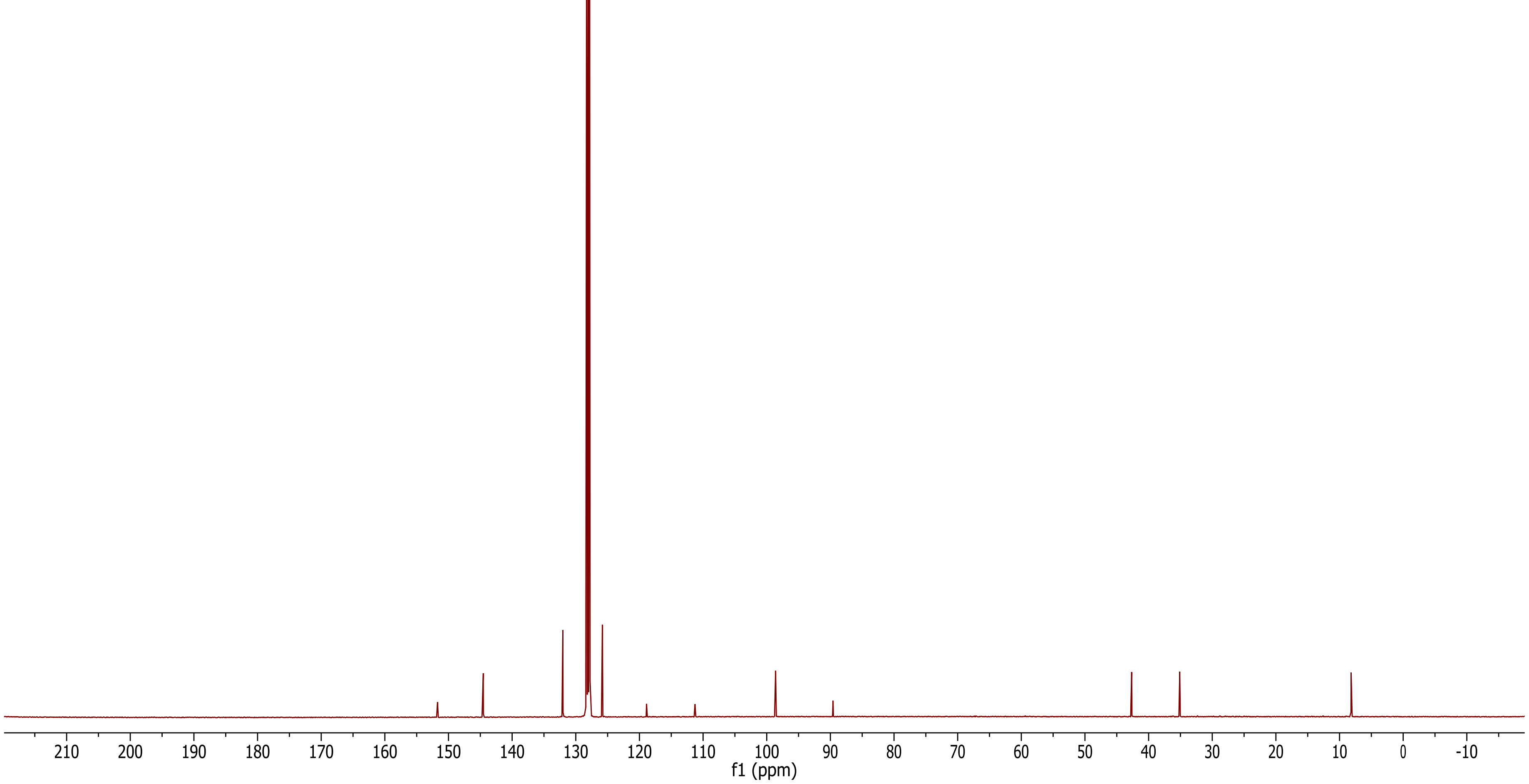

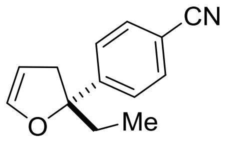

4bc
^{1}H-NMR
C_6D_6, 400 MHz, 298 K

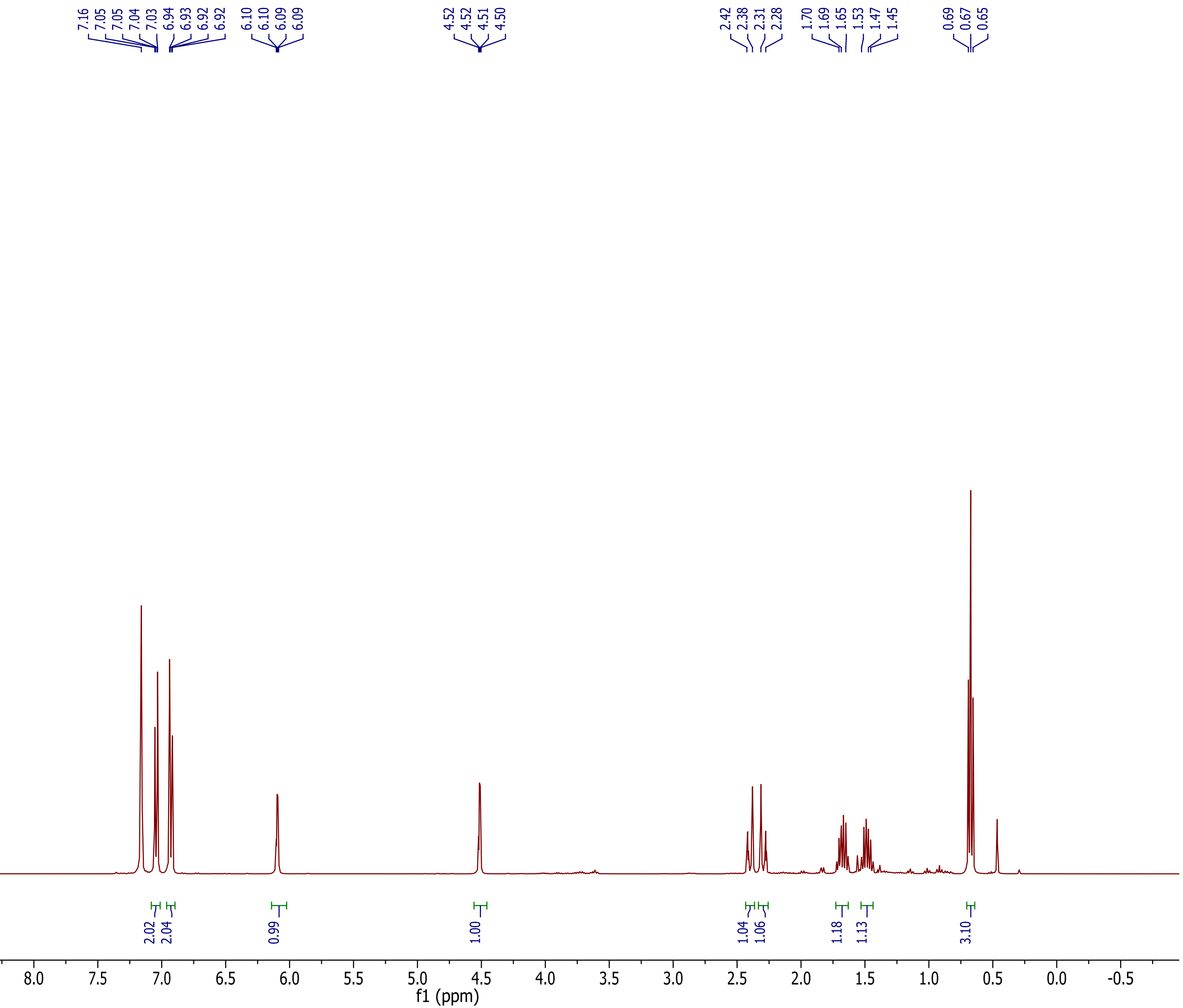

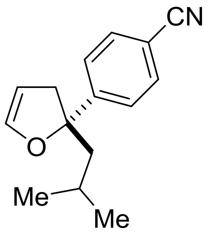

4cc

^{1}H-NMR

C_6D_6, 400 MHz, 298 K

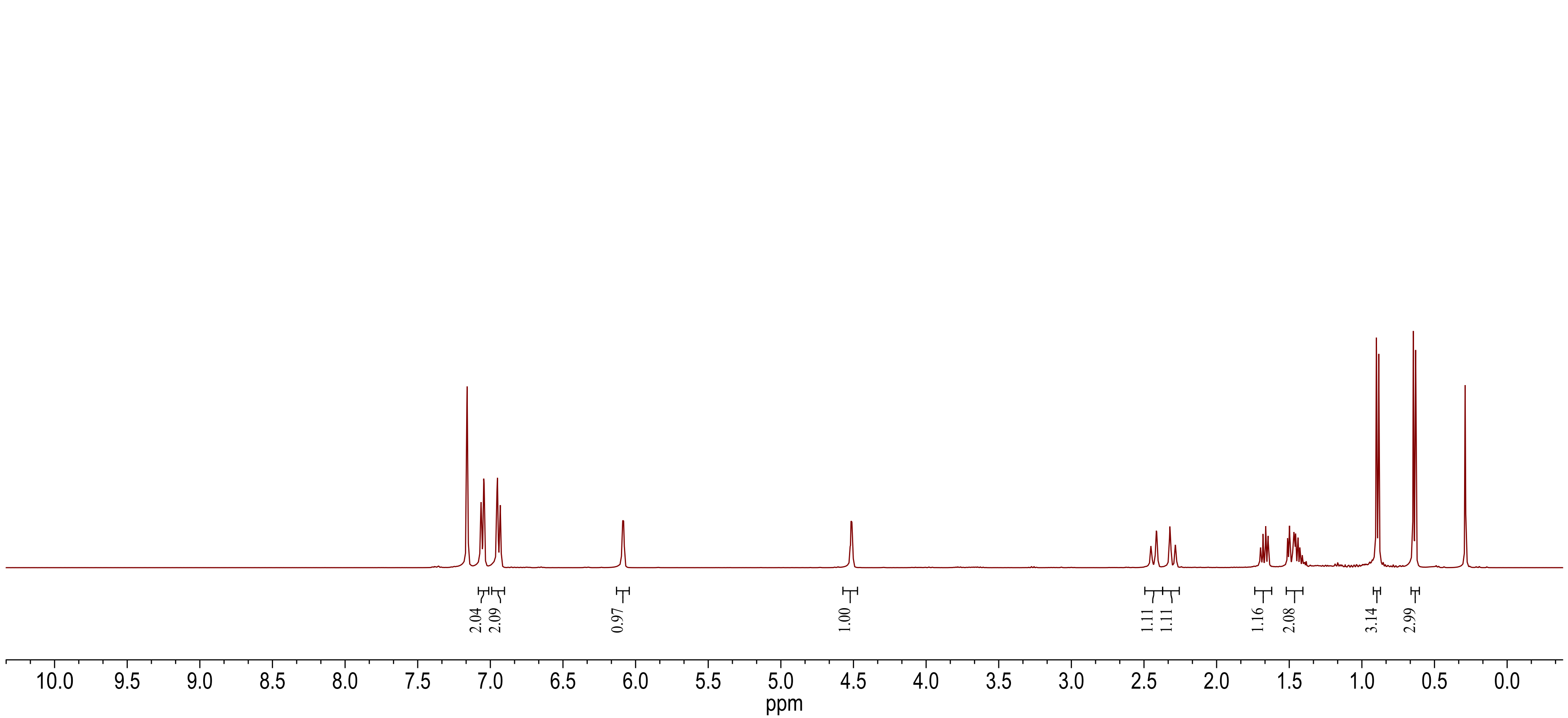

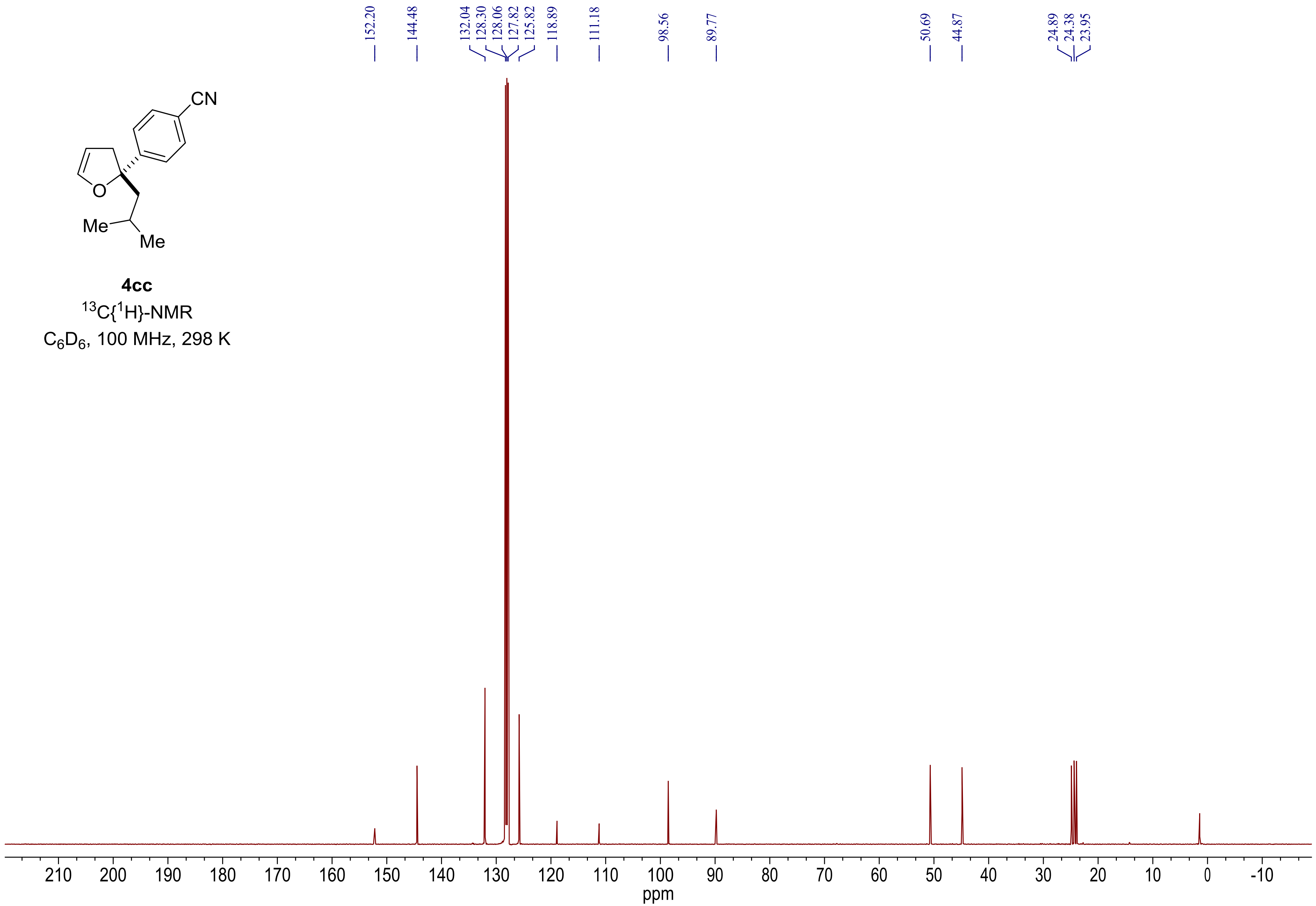

4cc

$^{13}C\{^1H\}$-NMR

C_6D_6, 100 MHz, 298 K

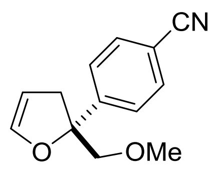

4dc

^{1}H-NMR

C_6D_6, 400 MHz, 298 K

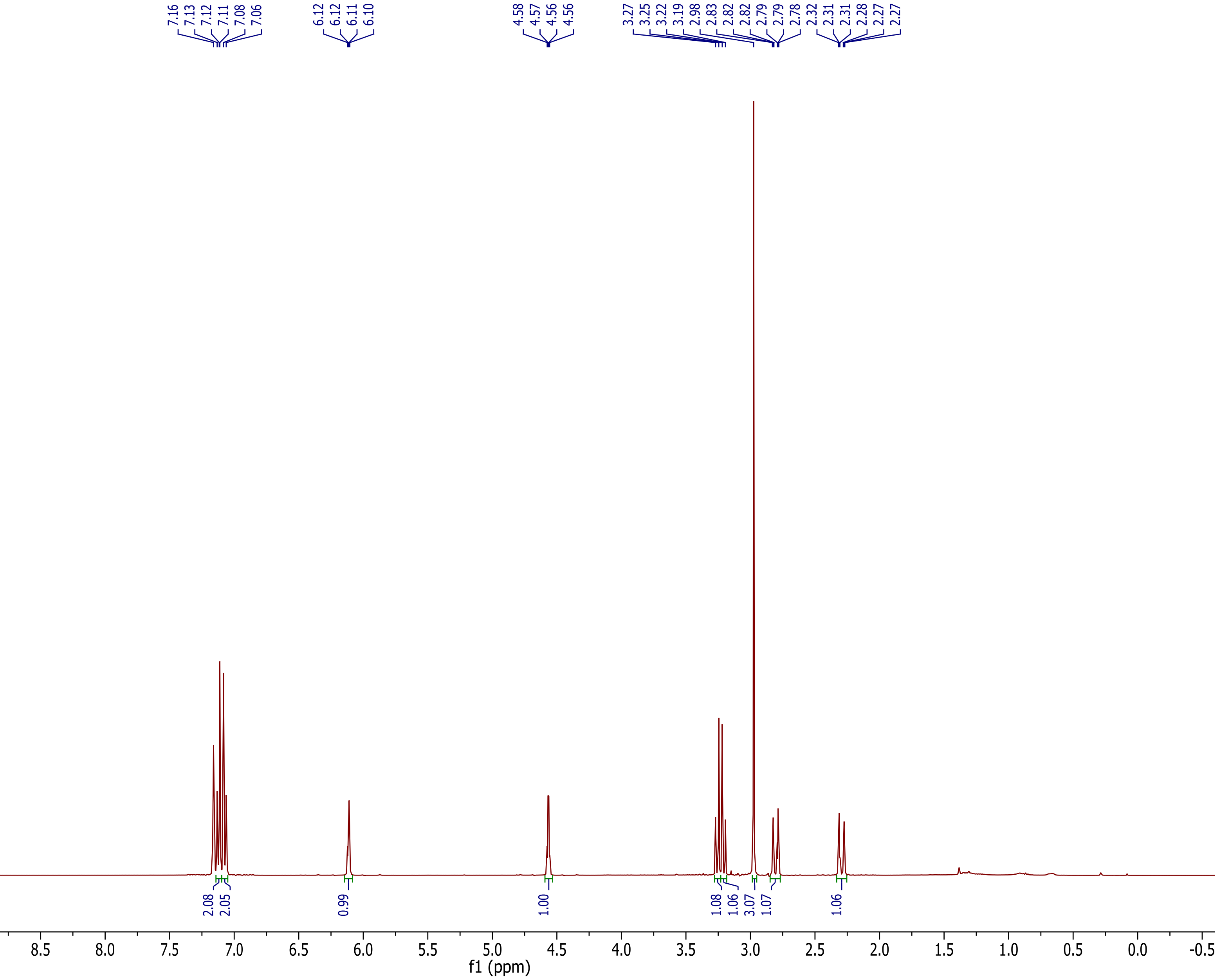

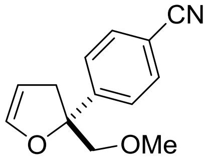

4dc
$^{13}C\{^{1}H\}$-NMR
C_6D_6, 100 MHz, 298 K

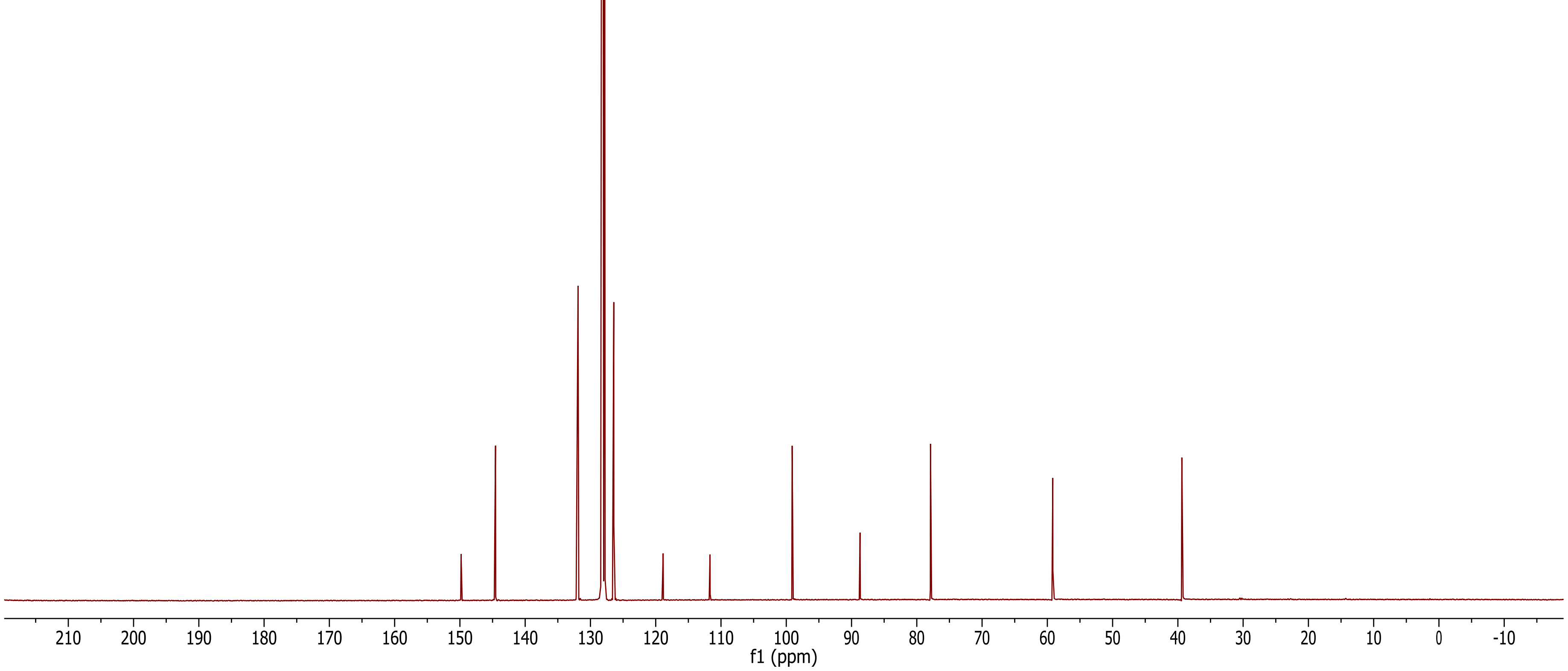

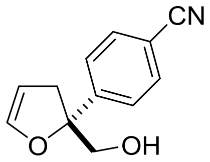

4ec
^{1}H-NMR
C_6D_6, 400 MHz, 298 K

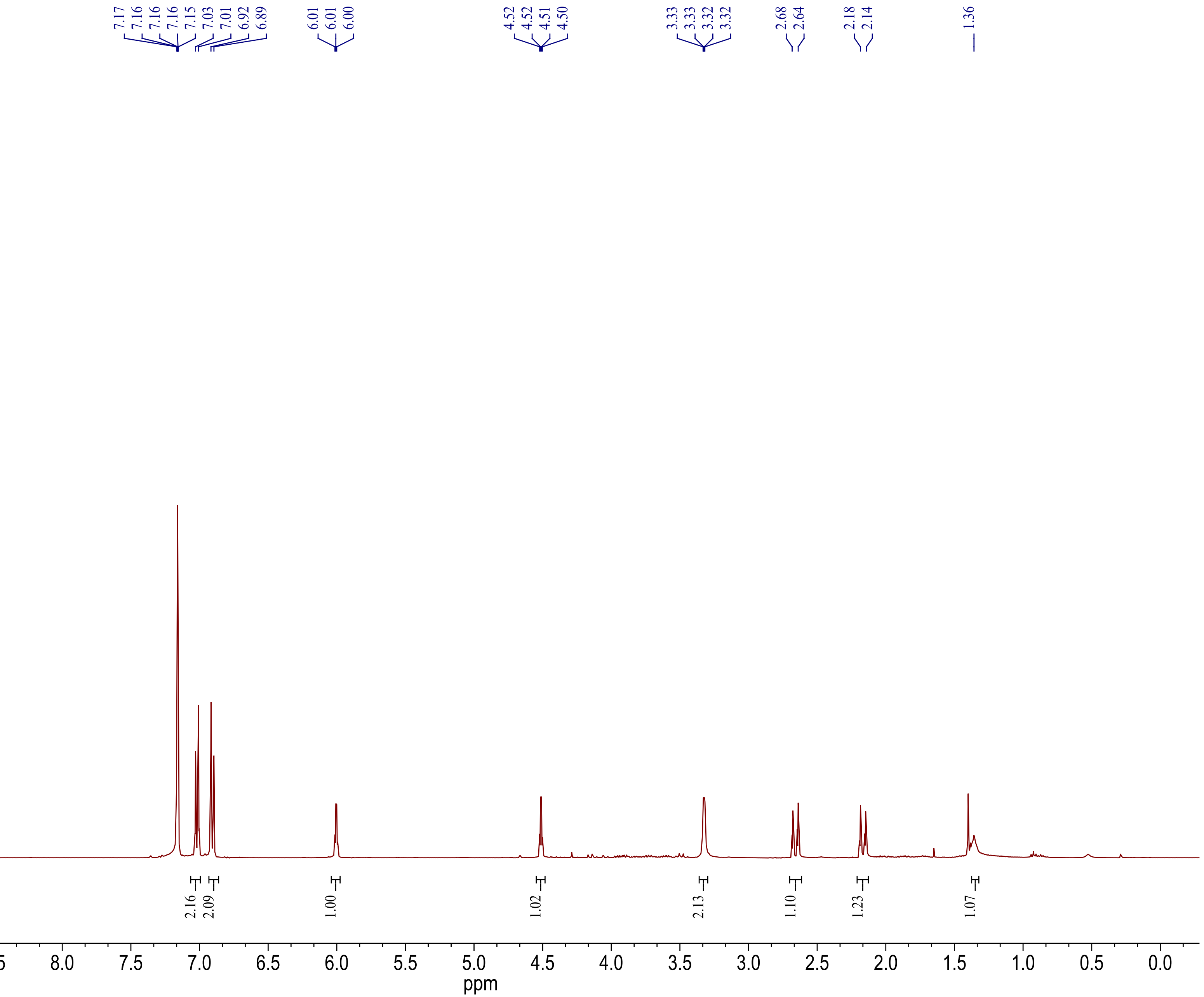

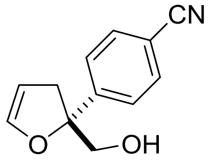

4ec
$^{13}C\{^{1}H\}$-NMR
C_6D_6, 100 MHz, 298 K

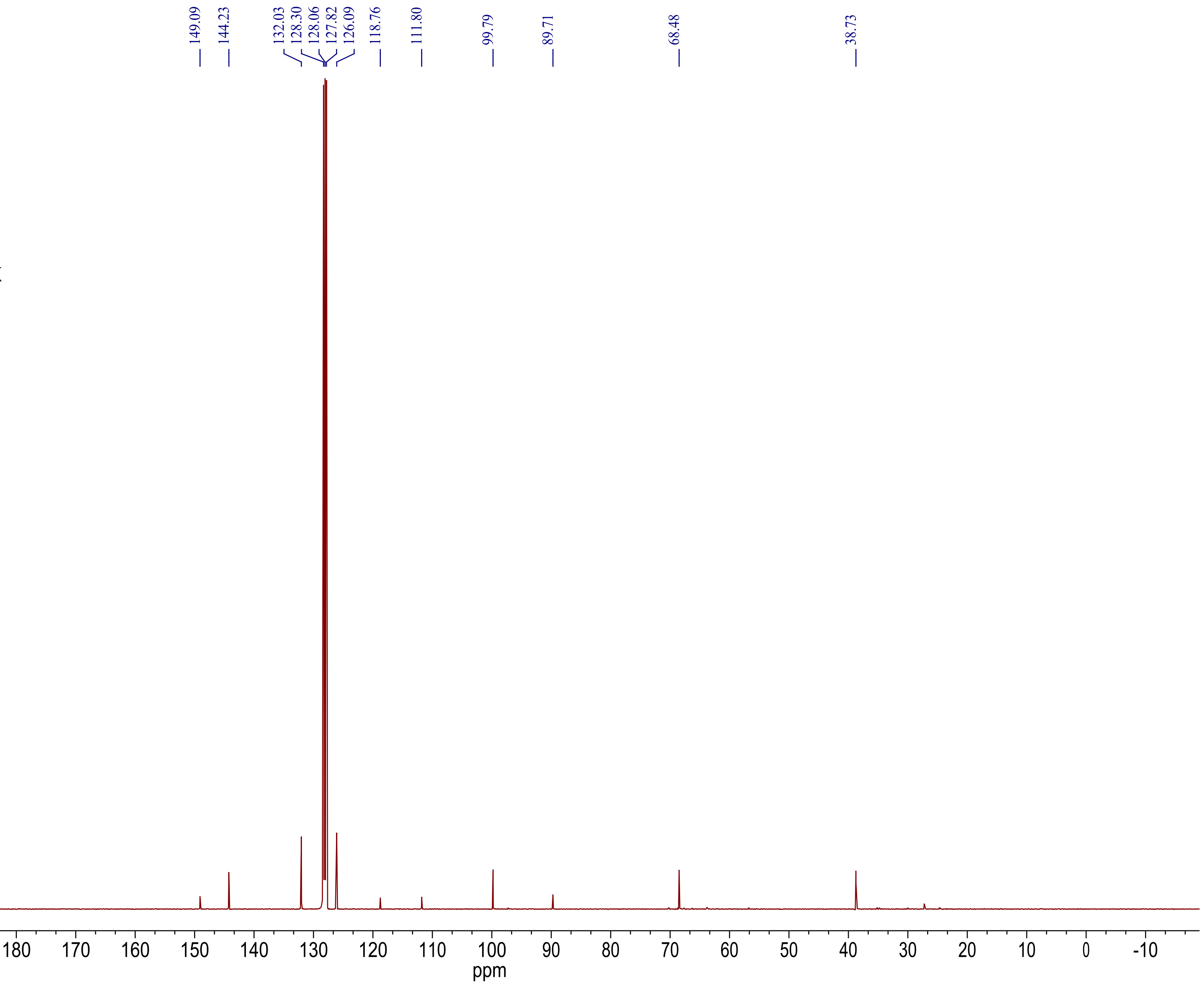

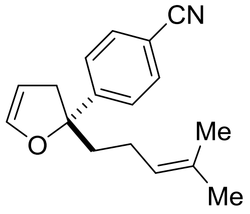

4fc

^{1}H-NMR

C_6D_6, 400 MHz, 298 K

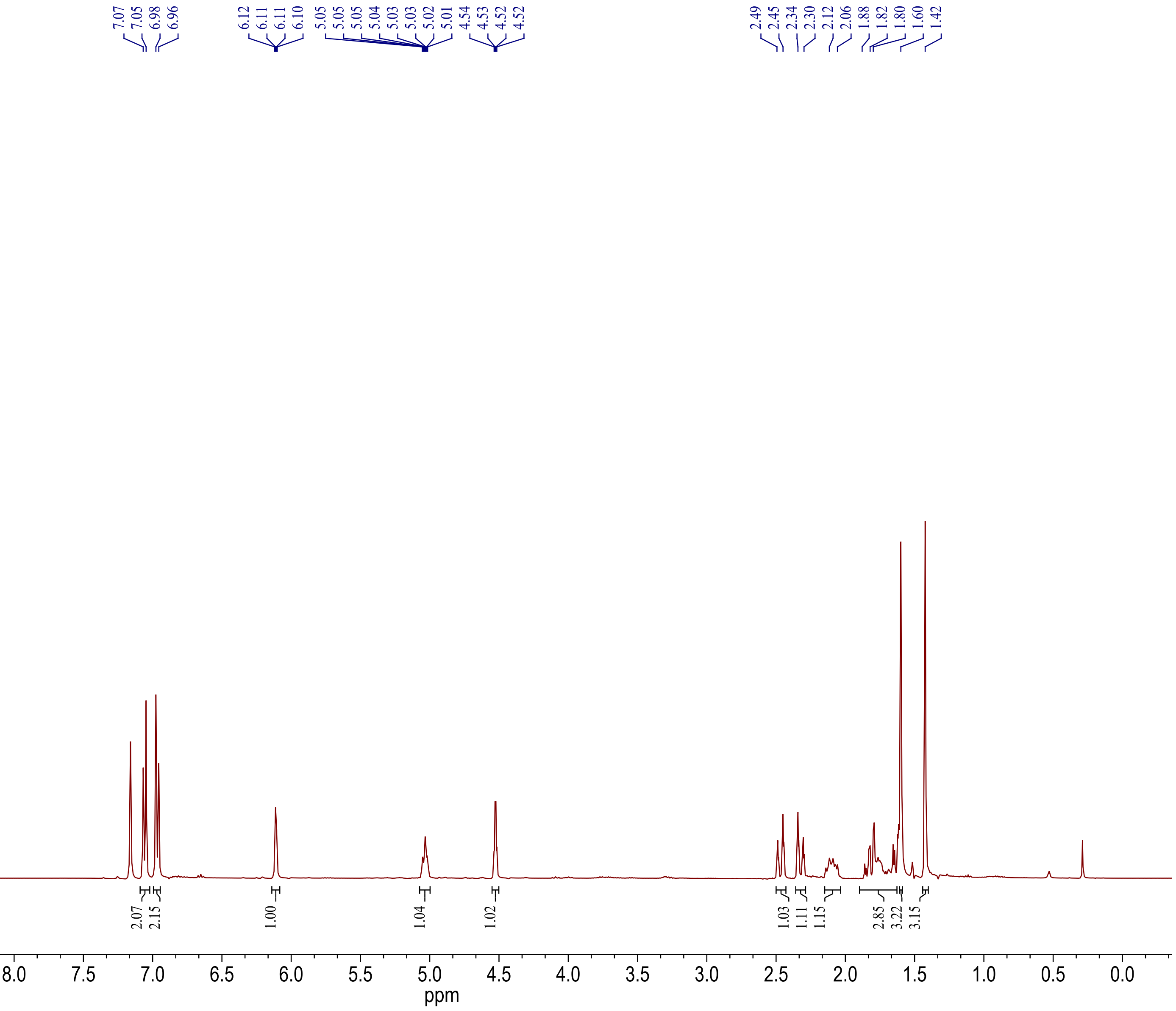

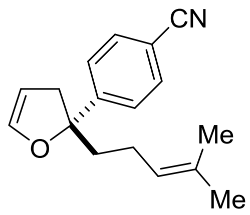

4fc

$^{13}C\{^{1}H\}$-NMR

C_6D_6, 100 MHz, 298 K

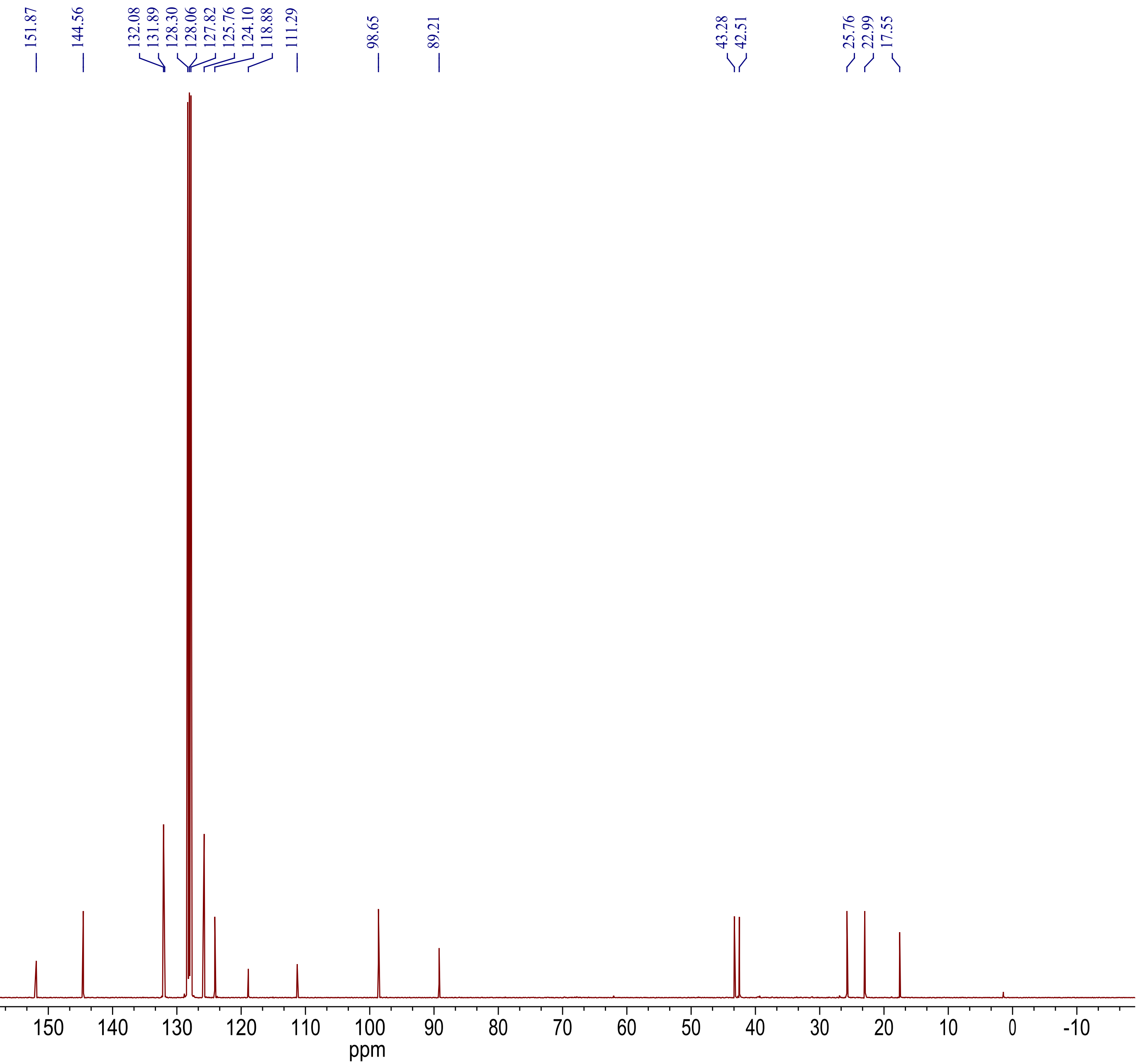

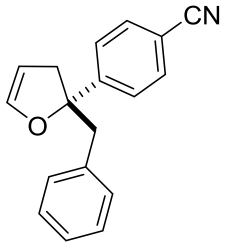

4gc
^{1}H-NMR
C_6D_6, 400 MHz, 298 K

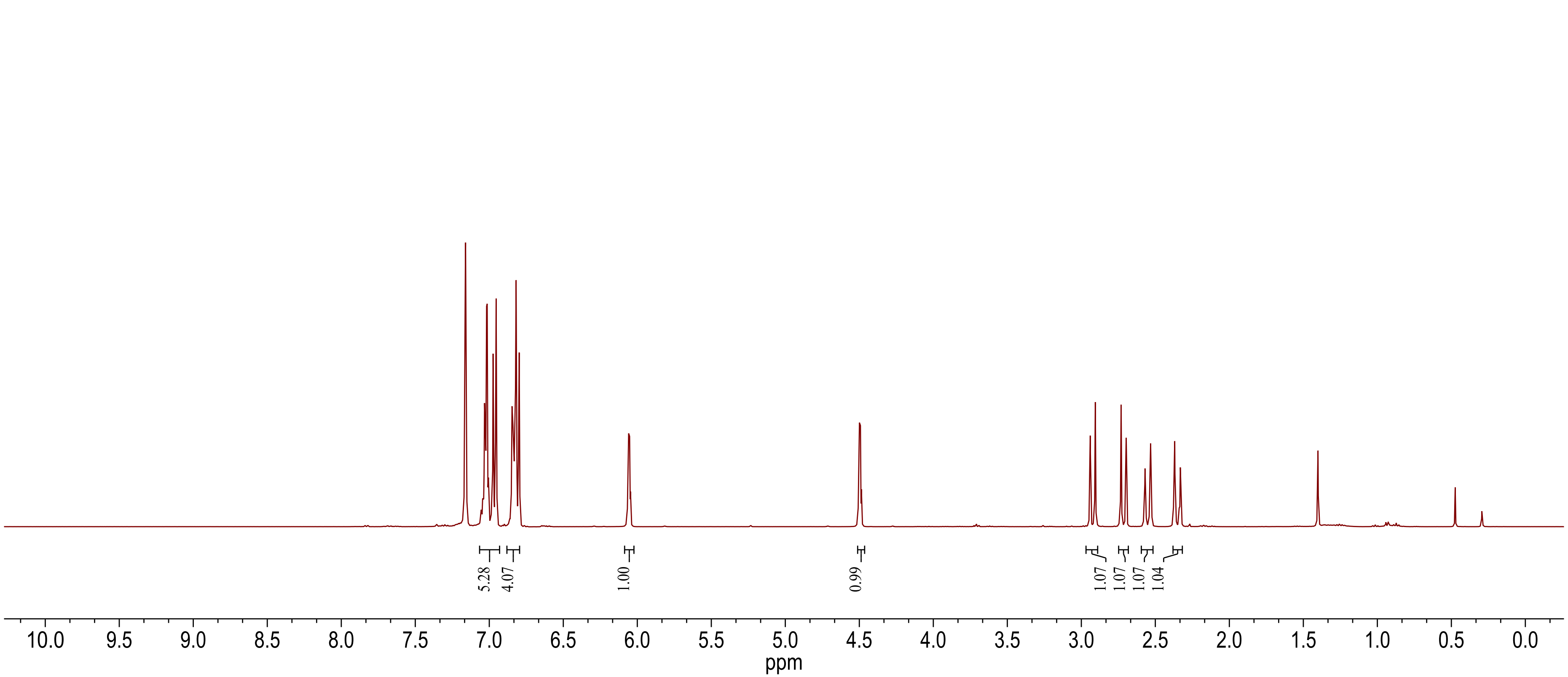

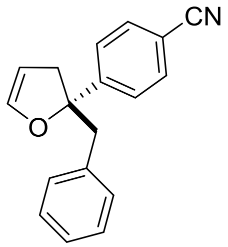

4gc
$^{13}C\{^{1}H\}$-NMR
C_6D_6, 100 MHz, 298 K

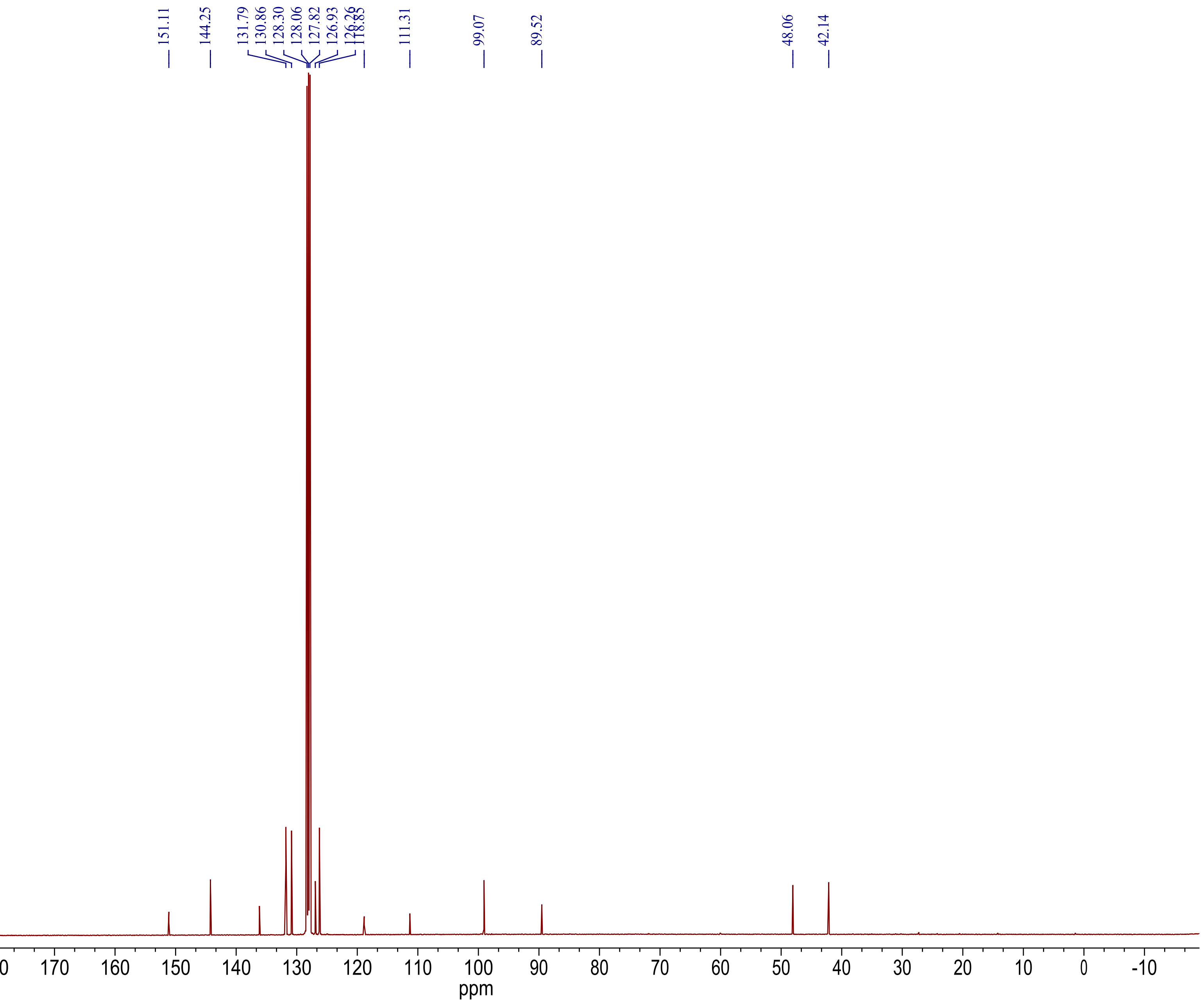

Supplement: Supplementary file 1 [file SC-006-C5SC01460C-s001.pdf]
